# Amplification and high-level expression of heat shock protein 90 marks aggressive phenotypes of human epidermal growth factor receptor 2 negative breast cancer

**DOI:** 10.1186/bcr3168

**Published:** 2012-04-17

**Authors:** Qing Cheng, Jeffrey T Chang, Joseph Geradts, Leonard M Neckers, Timothy Haystead, Neil L Spector, H Kim Lyerly

**Affiliations:** 1 Department of Surgery, Duke University Medical Center, Box 2606, 203 Research Drive, Durham, NC 27710, USA; 2 Department of Pathology, Duke University Medical Center, 3108 Meyer Ward, Durham, NC 27710, USA; 3 Department of Pharmacology & Cancer Biology, Duke University Medical Center, C118 LSRC, Durham, NC 27710, USA; 4 Department of Medicine, Duke University Medical Center, 101B MSRB, Durham, NC 27710, USA; 5 Department of Integrative Biology and Pharmacology, University of Texas Health Science Center, 6431 Fannin Street, Houston, TX 77030, USA; 6 Urologic Oncology Branch, Center for Cancer Research, National Cancer Institute, 9000 Rockville Pike Bethesda, MD 20892, USA

## Abstract

**Introduction:**

Although human epidermal growth factor receptor 2 (HER2) positive or estrogen receptor (ER) positive breast cancers are treated with clinically validated anti-HER2 or anti-estrogen therapies, intrinsic and acquired resistance to these therapies appears in a substantial proportion of breast cancer patients and new therapies are needed. Identification of additional molecular factors, especially those characterized by aggressive behavior and poor prognosis, could prioritize interventional opportunities to improve the diagnosis and treatment of breast cancer.

**Methods:**

We compiled a collection of 4,010 breast tumor gene expression data derived from 23 datasets that have been posted on the National Center for Biotechnology Information (NCBI) Gene Expression Omnibus (GEO) database. We performed a genome-scale survival analysis using Cox-regression survival analyses, and validated using Kaplan-Meier Estimates survival and Cox Proportional-Hazards Regression survival analyses. We conducted a genome-scale analysis of chromosome alteration using 481 breast cancer samples obtained from The Cancer Genome Atlas (TCGA), from which combined expression and copy number data were available. We assessed the correlation between somatic copy number alterations and gene expression using analysis of variance (ANOVA).

**Results:**

Increased expression of each of the heat shock protein (HSP) 90 isoforms, as well as HSP transcriptional factor 1 (*HSF1*), was correlated with poor prognosis in different subtypes of breast cancer. High-level expression of *HSP90AA1 *and *HSP90AB1*, two cytoplasmic HSP90 isoforms, was driven by chromosome coding region amplifications and were independent factors that led to death from breast cancer among patients with triple-negative (TNBC) and HER2-/ER+ subtypes, respectively. Furthermore, amplification of *HSF1 *was correlated with higher *HSP90AA1 *and *HSP90AB1 *mRNA expression among the breast cancer cells without amplifications of these two genes. A collection of *HSP90AA1*, *HSP90AB1 *and *HSF1 *amplifications defined a subpopulation of breast cancer with up-regulated HSP90 gene expression, and up-regulated HSP90 expression independently elevated the risk of recurrence of TNBC and poor prognosis of HER2-/ER+ breast cancer.

**Conclusions:**

Up-regulated HSP90 mRNA expression represents a confluence of genomic vulnerability that renders HER2 negative breast cancers more aggressive, resulting in poor prognosis. Targeting breast cancer with up-regulated HSP90 may potentially improve the effectiveness of clinical intervention in this disease.

## Introduction

Despite the progress that has been made in reducing mortality rates of breast cancer in the most recent time period, more than 40,000 breast cancer deaths occur in the United States annually [1]. Substantial progress in treatment requires identification of a specific set of actionable genomic abnormalities that drive or facilitate tumorigenesis, resistance to a given treatment and recurrence. Although significant amounts of gene expression profile analyses have been performed in breast cancers, assessing expression levels as the primary parameter to characterize breast cancers may be confounded by the phenotypic heterogeneity that arises as a consequence of abnormal signaling nodes and extensive biological cross-talk and redundancy. On the other hand, copy number aberrations in cancer cells can quantitatively affect gene function [2], and multiple copy number aberrations collectively regulate clinical phenotypes and cancer prognosis [3]. Analyses of chromosomal copy number aberrations (CNAs) have been proposed as a critical indicator of the possible location of aggressive cancer phenotype related genes [4,5]. Therefore, we undertook an integrative analysis of copy number and gene expression in a large population study to identify molecular factors abundant in breast cancer cells, especially in those characterized by aggressive behavior and poor prognosis, by which to prioritize interventional opportunities to transform breast cancer diagnosis, characterization, treatment and ultimately prevention.

Although a number of aberrant signaling pathways in breast cancer have been identified, heat shock protein 90 (HSP90), which is one of the most abundant proteins in mammalian cells [6], plays an important role in folding newly synthesized proteins or stabilizing and refolding denatured proteins after stress, and would influence a large number of signaling pathways. To date, more than 200 HSP90 clients have been identified, including key regulators in signal transduction and cell cycle control, steroid hormone receptors, and tyrosine and serine/threonine kinases [7-9]. HSP90 exists as multiple isoforms that include HSP90AA1 (an inducible form) and HSP90AB1 (a constitutive form) in cytoplasm, HSP90B1 in endoplasmic reticulum and TRAP1 in mitochondria [10]. However, unlike HSP90AA1 and HSP90AB1, the client proteins selectively interacting with HSP90B1 or TRAP1 chaperones have yet to be defined.

HSP90 contains an N-domain ATP binding site and its ATPase activity is necessary for all of its cellular functions [11]. *In vivo *Hsp90 does not function alone but acts in concert with co-chaperones such as Sba1/p23 and Cdc37[8]. Interactions with co-chaperones are thought to be important to direct Hsp90 function for specific physiological processes such as regulation of cell cycle progression, apoptotic responses, or kinase-mediated signaling cascades [10]. The protein is regulated both at the expression level and through posttranslational modifications such as phosphorylation, acetylation and methylation. These processes control its ATPase activity, and its ability to interact with its clients and co-chaperones, as well as its degradation [6,7]. In addition, HSP90 has a higher affinity for amino--terminal ligands in cancer cells, compared with the HSP90 in normal cells[12].

In breast cancer, HSP90 is required for the stabilization of many proteins in pathways that play key roles in cancer growth and survival, such as estrogen receptor (ER), progesterone receptor (PR), essential components of HER2 signaling (HER2, AKT, c-SRC, RAF and HIF-1α), and EGFR [9,13]. For example, HER2 is among the most sensitive client proteins of HSP90 [14,15], and HSP90 inhibition mediates degradation of HER2, as well as PI3K and AKT in HER2-overexpressing cancer cells[16]. Consequently, HSP90 inhibitors plus trastuzumab have significant anticancer activity in patients with HER2-positive, metastatic breast cancer previously progressing on trastuzumab[17]. Although a number of agents are in development for HER2+ and ER+ breast cancers, HSP90 inhibitors also represent therapeutic opportunities in other molecular subtypes. Triple negative breast cancer (TNBC) is defined by the clinical laboratory evaluation revealing a lack of expression of ER, PR and HER2 receptors, accounts for 10% to 20% of all breast cancer[18], and has a higher rate of distant recurrence and a poorer prognosis than other breast cancer subtypes [19,20]. Unfortunately, the lack of expression of a credentialed therapeutic target in this subtype of breast cancer limits the effective treatment options. Of interest, TNBCs often express increased EGFR protein, but in early clinical trials, response rates to EGFR inhibitors were minimal.

One potential therapeutic opportunity in tumor subtypes that do not have a known therapeutic target could include targeting Hsp90 function. Although Hsp90 protein expression was reported to be relatively low in TNBC compared to other subtypes, this early report only evaluated nine tumors [21]. More encouragingly, in pre-clinical models, TNBCs have been sensitive to Hsp90 inhibitors [22,23]. Similarly to HER2 positive tumors, TNBCs were sensitive to Hsp90 inhibition through down-regulation of components of the Ras/Raf/MARK pathway in preclinical and *in vitro *studies [23]. Being a central integrator of multiple pathways, activation of HSP90 may maintain the malignant phenotype, facilitate metastasis, and promote treatment-resistance under the stress of cancer therapy in multiple breast cancer subtypes. It has been suggested that Hsp90 up-regulation may be a sign of poor disease prognosis [24] and a recent study has demonstrated that co-expression of HSP90 and PI3K or expression of HSP90 in combination with the loss of PTEN were associated with significantly worse recurrence-free survival in patients with breast cancer [25]. However, adequately powered population studies correlating up-regulated HSP90 with prognosis in breast cancer patients have not been performed to date.

In this study, we exploited the availability of publicly available data and performed a genome scan for somatic copy number aberrations and gene expression profiling of primary breast tumors to address the general prognostic significance of gene amplification and high-level expression in breast cancer. We found that up-regulated HSP90 was one of the most significant poor prognosis factors in triple negative and HER2-/ER+ breast cancer subtypes. Our result suggested that targeting breast cancer with up-regulated HSP90 would potentially reduce the risk of lethal recurrence and distant metastasis.

## Materials and methods

### Human breast tumor samples and data collection

A total of 4,010 breast cancer gene expression profiles were collected from 23 independent data sets (GSE22093, GSE17705, GSE11121, GSE12093, GSE7390, GSE5327, GSE6532, GSE1456, GSE2034, GSE3494, GSE26639, GSE20685, GSE23720, GSE21653, GSE16446, GSE23177, GSE19615, GSE12276, GSE9195, GSE17907, GSE16391, GSE22035 and GSE5460) that were on NCBI Gene Expression Omnibus (GEO). Primary breast tumor samples were obtained before treatment and gene expression profiles were measured using Affymetrix U133A or U133 Plus 2.0 expression array. Each dataset selected for this study should have either clinical outcome data and/or HER2, ER or PR status determined by immunohistochemistry (Additional file 1). Patients' unique IDs were also collected from series matrix files (GEO) to ensure there is no redundant sample set. In addition, we successfully processed somatic copy number alterations (CNAs) of 481 breast invasive carcinoma samples that were measured using Affymetrix Genome-Wide Human SNP Array 6.0, of which gene expression profiles of the same set of primary tumor samples were also measured using Agilent Expression 244 K microarrays by The Cancer Genome Atlas Project (TCGA).

### Processing of gene expression data

**Figure 1:**
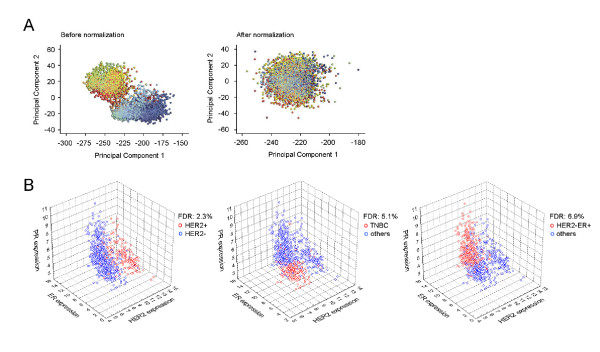
**Analysis of 4,010 breast cancer sample**. **(A) **PCA plots of before normalization and after normalization. These plots show the gene expression profiles of the samples plotted on the first two principal components. Each point represents a sample, and samples from the same data set have the same color. If there are batch effects, the samples from the same data set (the same color) will cluster together. If there are no batch effects, the colors should be mixed. **(B) **Prediction of HER2+, TNBC and HER-/ER+ breast cancer subtypes using HER2, ER and PR mRNA expression levels.

Raw Affymetrix expression CEL files from each dataset were RMA (Robust Multi-array Average) normalized independently using Expression Console Version 1.1 (Affymetrix). All data were filtered to include those probes on the HG-U133A platform. Assuming that the signal from the 69 Affymetrix control probes should be invariant, we found the structure in those probes by taking the first 15 principal components, and then removed the contribution of those patterns in the expression of genes using Bayesian Factor Regression Modeling (BFRM) [26]. A Principal Component Analysis (PCA) and Heatmap were used to confirm dataset normalization (Figure 1 and Additional file 2). By this procedure, we generated a normalized gene expression dataset compiling 4,010 breast tumor samples.

### Copy number analyses

Somatic copy number alterations (CNAs) of invasive breast cancer samples collected from 517 female patients were measured using Affymetrix Genome-Wide Human SNP Array 6.0. CEL files were available from TCGA. SNP array data from matched blood lymphocytes or matched normal tissue were also available for 494 patients. We generated a canonical genotype cluster using a data set of 799 Affymetrix Genome-Wide Human SNP 6.0 arrays that measured from normal blood lymphocytes obtained from TCGA. In total, 1,831,105 SNP and copy number markers were analyzed to construct canonical clustering positions and Log R ratio (LRR) and B allele frequency (BAF) from raw CEL files were calculated using PennCNV-Affy [27]. Matched normal samples were genotyped using Affymetrix genotyping console (version 4) and all samples were compared to ensure there was no duplication. All copy number markers and SNPs with genotype call rate higher than 90% were selected for tumor copy number analysis, and CNA calls were generated using genoCN software [28]. Genotype calls from normal tissues of the same individual were applied for genoCNA analysis, if they were available. Thirty-six samples that failed to obtain estimated parameters after 200 iterations of EM were removed from further study. All probe coordinates were mapped to the human genome assembly build 36 (hg18). In total, tumor copy number on chromosome 1-22 and chromosome X were successfully measured in 481 TCGA breast tumor samples, and normalized gene expression data from the same set of samples were downloaded from TCGA.

### Statistics analyses

We downloaded the Affymetrix U133A annotation file (hg18) from Affymetrix and removed probe sets that do not have a matched gene symbol or whose probe set's alignment did not match with gene chromosome location (pseudogenes). Using all 4,010 samples, we defined the gene expression level at each probe set as low-level expression (bottom 10% low expression value), intermediate-level expression (middle 80% expression value) and high-level expression (top 10% high expression value), and compared survival differences among those three groups using Cox-regression survival analyses. Co-efficiency was used to ensure if high-level expression was associated with poor prognosis and low-level expression was correlated with better outcome. A total of 11,761 known genes were analyzed. Statistical analyses were performed using R Project for Statistical Computing (Augasse, Austria), Matlab (Natick, MA, USA) or STATISTICA (Tulsa, OK, USA). Kaplan-Meier survival analyses on selected genes were conducted using GraphPad (La Jolla, CA, USA).

To measure the correlation between copy number aberration and gene expression, we generated copy number calls at 1,794,774 probes on chromosome 1-22 and chromosome X from all samples, including 857,551 SNPs and 937,223 CN markers. We determined copy number calls at each marker site as homozygous deletion (CN = 0), hemizygous deletion (CN = 1), normal copy number (CN = 2), low level amplification (CN = 3) and high level amplification (CN ≥4). We downloaded normalized expression data (level 2) from the TCGA database and analyzed the association between copy number and gene expression using analysis of variance (ANOVA). Associated region was defined as the region that should cover at least five consecutive SNPs or CN markers and should be longer than 10 kb. Direct correlation was defined as amplification associated with high-level expression and deletion was correlated with low-level expression.

## Results

### Analysis of 4,010 breast cancer samples

To conduct a genome wide survey for poor prognosis-associated genes in breast cancer, we compiled a collection of breast tumor gene expression data (*n *= 4,010) derived from 23 datasets that were posted on the NCBI Gene Expression Omnibus (GEO, Table 1) and normalized by Bayesian Factor Regression Modeling (BFRM) to remove technical variation (Figure 1A; Additional file 2) [26]. In addition to the raw expression data, we also obtained clinical outcome data from a subset of the samples (Additional file 1), which included data on overall survival (*n *= 1,027), recurrence-free survival (*n *= 1,372), and distant metastasis free survival (*n *= 2,187), as well as disease specific survival (event of death from breast cancer, *n *= 395).

**Table 1:** Summary of 23 data sets.

Data set	Institution	Array Platform	number of array	prognosis	IHC	**Ref**.
GSE22093	UT MD Anderson, TX, USA	HG-U133A	82		ER	[43]
GSE17705	Nuvera Biosciences, MA, USA	HG-U133A	298	dmfs	ER	[44]
GSE11121	Bayer Technology Services GmbH, Leverkusen, Germany	HG-U133A	200	dmfs		[45]
GSE12093	Veridex LLC, CA, USA	HG-U133A	136	dmfs		[46]
GSE7390	Institut Jules Bordet, Bruxelles, Belgium	HG-U133A	198	os, rfs, dmfs	ER	[47]
GSE5327	University of Chicago, IL, USA	HG-U133A	58	dmfs		[48]
GSE6532	Institut Jules Bordet, Bruxelles, Belgium	HG-U133A, HG-U133_Plus_2	414	rfs, dmfs	ER, PR	[49]
GSE1456	Karolinska Institutet, Stockholm, Sweden	HG-U133A	159	os, rfs, dmfs, Death_fromBC		[50]
GSE2034	Veridex, CA, USA	HG-U133A	286	rfs	ER	[51]
GSE3494	Genome Institute of Singapore, Singapore	HG-U133A	251	Death_fromBC	ER, PR	[52]
GSE26639	Institut Curie, Paris, France	HG-U133_Plus_2	226		HER, ER, PR	[53]
GSE20685	Koo Foundation SYS Cancer Center, Taiwan	HG-U133_Plus_2	327	os, mfs		[54]
GSE23720	Institut Paoli-Calmettes, Marseille, France	HG-U133_Plus_2	197		ER, PR	[55]
GSE21653	Institut Paoli-Calmettes, Marseille, France	HG-U133_Plus_2	266	dmfs	HER2, ER, PR	[56]
GSE16446	Institut Jules Bordet, Bruxelles, Belgium	HG-U133_Plus_2	120	os, dmfs	HER2, PR	[57]
GSE23177	Flanders Institute for Biotechnology, Leuven, Belgium	HG-U133_Plus_2	116		HER2, ER	[58]
GSE19615	Dana-Farber Cancer Institute, MA, USA	HG-U133_Plus_2	115	dmfs	HER2, ER, PR	[59]
GSE12276	Erasmus Medical Centre, Rotterdam, Netherlands	HG-U133_Plus_2	204	rfs		[60]
GSE9195	Institut Jules Bordet, Bruxelles, Belgium	HG-U133_Plus_2	77	rfs, dmfs	ER, PR	[61]
GSE17907	Institut Paoli-Calmettes, Marseille, France	HG-U133_Plus_2	55	mfs	HER2, ER, PR	[62]
GSE16391	Institut Jules Bordet, Bruxelles, Belgium	HG-U133_Plus_2	55	rfs	HER2, ER, PR	[63]
GSE22035	Centre Rene Huguenin, SAINT-CLOUD, France	HG-U133_Plus_2	43		ER	[64]
GSE5460	Dana-Farber Cancer Institute, MA, USA	HG-U133_Plus_2	127		HER2, ER	[65]

As shown in Table 1, the majority of samples lacked the molecular analysis of HER2, ER and PR expression as measured by immunohistochemistry (IHC) or fluorescent *in situ *hybridization (FISH) analysis. Nevertheless, we found significant correlations between mRNA expression level and reported HER2, ER or PR status measured by IHC (*P *< 1 × 10^-8^, Mann-Whitney U test, Additional file 3), which was consistent with previous reports that ER, HER2 and PR biochemical status was concordant with Affymetrix microarray data [29,30]. By fitting two normal distributions of mRNA expression into IHC positive and negative groups, we identified a bimodal cutoff that represents maximum likelihood of IHC status, using samples where the biochemical status of HER2 (*n *= 1,004), ER (*n *= 2,771) and PR (*n *= 1,559) was available [29], and then applied this predictive cutoff to the entire set of 4,010 samples (Additional file 4). Clinical outcomes of gene expression defined subtypes were highly concordant with IHC subtypes (Additional file 4). When mRNA expression of *HER2*, *ER *and *PR *were applied together, the over-all accuracy for HER2+, triple-negative and HER2-/ER+ was 91.7%, 91.5%, and 89.6%, respectively, comparing with the biochemical defined breast cancer subtypes (Figure 1).

### Genome-scan of copy number aberration in 481 breast cancer samples

**Figure 2:**
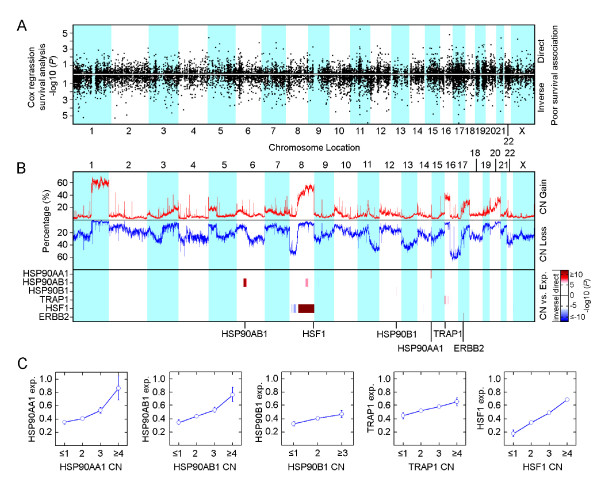
**Correlation of HSP90 expression and coding region copy number aberrations**. **(A) **Genome scans for poor prognosis associated gene. Correlation between gene expression and risk of death from breast cancer was assessed using Cox-regression survival analyses. Direct correlation is high-level expression was associated with poor survival. Inverse correlation is high-level expression was associated with better outcome. The *y *axis represents the level of significance for each expression probe set (log-transformed *P *values) at the relative genomic position on each chromosome along the *x *axis from the short-arm terminus (left) to the long-arm terminus (right). Bottom panel shows somatic CNA distribution across entire genome. **(B) **Genome scans for somatic CNA distribution and its correlation with HSP90 and HSF1 expression. Upper panel shows percentage of amplification (low-level and high-level amplification) and deletion (homozygous and hemizygous deletion) at each detected chromosome region in a group of 481 breast cancer patients. Bottom panel shows correlation between CNA and HSP90 and HSF1 mRNA expression. ERBB2 was used as positive control. Analysis of variance (ANOVA) was performed to test for association between copy numbers and gene expression. **(C) **Scatterplots of correlation between mRNA expression and copy numbers of select genes: homozygous deletion (0), hemizygous deletion (1), normal copy number (2), low level amplification (3) and high level amplification (≥4), measured by ANOVA analysis. Circles represent average levels. Vertical bars represent 0.95 confidence intervals.

Chromosomal aberrations reflect oncogene activation and loss of tumor suppressor genes. Surveys of DNA gain or loss have been considered a fertile area to search for determinants of treatment response and disease outcome in human cancer cells. In breast cancer, it has been reported that 44% to 62% of highly amplified genes were over-expressed [31,32] and at least 12% of the total variation in gene expression was directly attributed to copy number aberrations [33]. TCGA data provide a unique opportunity to enable different and potentially complementary forms of analysis of cancer phenotypes given the comprehensive nature of the datasets generated in this effort. We were particularly interested in the opportunity to link genomic copy number alterations with the observed gene expression profile and clinical data as a strategy to identify genomic determinants of poor prognosis. We therefore performed a genome-scale analysis of chromosome alteration using 481 breast cancer samples obtained from the TCGA project, from which combined expression and copy number data were available. We revealed the distribution of copy number amplifications and deletions across the entire genome (Figure 2). As expected, we observed that 23.7% of breast cancer samples had amplification (CN ≥3) on the *HER2 *coding region. Although copy number abnormalities on chromosome 1, 8, 11 and 16 are more common in studied populations (*n *= 481), we found that in most chromosome regions, both amplifications (CN ≥3) and deletions (CN ≤1) occurred in approximately 10% of analyzed samples (Figure 2).

### Identification of genes that were correlated with risk of death from breast cancer

The large cohort of 4,010 gene expression samples provided an opportunity to define a subpopulation of patients containing either extremely high or low expression levels of candidate genes and to identify genes whose high-level expression is predominant in a poor prognosis stage compared to a better prognosis stage. To determine poor prognosis-associated genes, we performed two stage analyses. In the first stage, we selected a universal cut-off and assigned each of the 4,010 samples into low, intermediate and high expression categories for each of 11,761 known genes. Then, we carried out an unbiased, genome wide Cox-regression survival analysis, comparing the prognosis difference among those three groups. By doing this, poor prognosis-associated genes should show a poor prognosis in the high expression group and a better outcome in the low expression group. In the second stage, we further assessed the poor prognosis correlation of the identified genes using gene-expression as a continuous variable and sought to correlate copy number aberrations with gene expression by measuring if amplification was correlated with high-level expression and deletion was associated with low-level expression.

Starting with the extreme, we defined the lowest 10% of expression values across the entire 4,010 samples as low-level expression and the highest 10% of expression values as high-level expression. Using death from breast cancer as the incident event, we carried out a genome wide Cox-regression survival analysis and identified 152 genes whose high-level expression was significantly associated with higher risk of death from breast cancer (*P *< 0.01, Figure 2 and Additional file 5). In addition, we assigned each of the 4,010 samples into first quartile (lowest 25%), second quartile (intermediate 50%) and third quartile (highest 25%) subgroups according to the expression levels of the 152 identified genes, and compared prognosis differences among these subgroups. Furthermore, we applied expression signal as a continuous variable to measure the distribution of the identified genes. A total of 47 of the 152 genes showed linear correlation between increased expression and poor prognosis. The highest risk of death from breast cancer was observed in patients with either top 10% or 25% higher level gene expression (*P *< 0.05, Additional file 5).

Since amplifications or deletions are likely to control the expression of genes within the corresponding region, and the correlation between copy number and expression has been recently suggested as an approach to predict the authentic molecular drivers in carcinogenesis [34], we then extended this analysis of gene expression to assess the correlation between somatic copy number alterations and gene expression using 481 invasive breast cancer samples obtained from TCGA. We found that 26 of 47 poor prognosis-associated genes showed a significant correlation between copy number aberrations and mRNA expression (*P *< 1 × 10^-8^, ANOVA, Additional file 5 and Additional file 6). To support this modeling, we analyzed the expression of HER2, a well known oncogene associated with poor prognosis based on increased copy number and high gene expression. As expected, high-level expression of *HER2 *was driven by coding region amplification and was significantly associated with poor prognosis (Additional file 5). Importantly, we found both cytoplasmic HSP90 isoforms, *HSP90AA1 *and *HSP90AB1*, were among the most significant factors that led to higher risk of death from breast cancer, indicating that HSP90 plays an important role in modulating poor prognosis phenotypes in breast cancer (Additional file 5).

### Increasing expression of HSP90 was correlated with poor prognosis of breast cancer

To address the extent to which HSP90 is a prognostic factor in breast cancer, we analyzed the correlation between HSP90 expression and clinical disease outcomes, such as survival, recurrence, and metastasis, in different subtypes of breast cancer. Other HSP90 isoforms, such as *HSP90B1 *and *TRAP1*, may affect treatment responses in specific subtypes of breast cancer and this effect could be largely diluted in the analysis of a heterologous population. Therefore, *HSP90B1 *and *TRAP1*, as well as HSP transcriptional factor 1 (*HSF1*), were also included.

We assessed the correlation between mRNA expression and poor prognosis in different breast cancer subtypes using Cox-regression survival analysis and compared survival differences between high-level expression (top 10% or 25%) and low-level expression groups using Kaplan-Meier Estimated survival analysis. To elucidate if high-level expression of HSP90 isoforms were truly independent prognostic factors, we conducted Cox Proportional-Hazards Regression (COXPH) survival analyses to quantify the weight of the hazard ratios associated with high expression and their significance when considered alongside other clinical variables, such as size, grade, nodal status, age, HER2, ER and PR, in the whole cohort and in the relevant subtype of cancer.

We found that high-level expression of *HSP90AA1 *independently led to higher risk of death from breast cancer in TNBC, while *HSP90AB1 *caused poor survival among patients with the HER2-/ER+ breast cancer subtype through increased risk of distant metastasis (Table 2 and Additional file 7). High-level expression of *HSP90AB1 *was an independent factor affecting disease-specific survival (death from breast cancer) and over-all survival of breast cancer (Table 2). In addition to these findings, we found that a higher risk of recurrence in HER2+ and HER2-/ER+ breast cancer subtypes was significantly correlated with increased expression of *HSP90AA1 *and *HSP90B1*; and increasing expression of *HSP90AA1 *and *HSP90AB1 *were significantly associated with a higher chance of distant metastasis in patients with HER2-/ER+ tumor (Additional file 7).

**Table 2:** Prognosis of HSP90AA1 and HSP90AB1 in different subtypes of breast cancer.

Subtype	Gene	Cox-regression analysis		Kaplan-Meier survival analysis					COXPH survival analysis		
				High 25% vs. others		High 10% vs. others			High 10% vs. others		
		*P*-value	n	*P*-value	HR(95%CI)	*P*-value	HR(95%CI)	n	*P*-value	*P*-adjusted	n
All samples	HSP90AA1	0.0020	395	0.0499	1.75(1.00-3.06)	0.0241	2.81 (1.15-6.90)	395	0.0193	0.3320	225
(dss)	HSP90AB1	0.0136		0.0404	1.72(1.02-2.90)	0.0011	3.69 (1.68-8.07)		0.0022	0.0008	
All samples	HSP90AA1	0.0081	1072	0.0384	1.39(1.02-1.89)	0.0002	2.55(1.55-4.21)	1072	0.0048	0.1069	421
(os)	HSP90AB1	0.0175		0.0401	1.36(1.01-1.83)	0.0024	2.06(1.29-3.28)		0.0010	0.0022	
HER2+	HSP90AA1	0.7459	194	0.694	0.89(0.50-1.60)	0.1523	2.07(0.76-5.61)	194	0.4364	0.2703	63
(os)	HSP90AB1	0.5693		0.6728	1.15(0.59-2.24)	0.3733	1.76(0.51-6.13)		4.90E-08	0.1839	
HER2-ER+	HSP90AA1	0.1057	506	0.0706	1.52(0.97-2.39)	0.0563	1.92(0.98-3.75)	506	0.1593	0.5829	228
(os)	HSP90AB1	0.0015		0.0918	1.44(0.94-2.20)	0.0005	3.04 (1.63-5.68)		1.53E-05	0.0004	
TNBC	HSP90AA1	0.0049	282	0.0302	2.07(1.07-3.98)	< 0.0001	16.9(4.66-60.9)	282	0.0079	0.0394	105
(os)	HSP90AB1	0.1328		0.0483	1.82(1.00-3.30)	0.2936	1.83 0.59-5.66)		0.4344	0.9968	

Cox-regression survival analysis was performed using gene expression signal as continuing variable. CI, confidence interval; Dss, disease specific survival (death from breast cancer); HR, Hazard Ratio; n: number of samples; os, over-all survival.

Among patients with TNBC, higher expression of HSP90 isoforms (*HSP90AA1*, *HSP90AB1, HSP90B1 *and *TRAP1*) was correlated with higher risk of recurrence. However, these significant interactions were not observed after adjusted multiple clinical availables. This might be affected by the fact that the entire set of clinical variables were only available in a small proportion of the samples. It also indicated that a single HSP90 isoform might only have a slight influence on disease outcome, such that when several interactions occur together, the combined effect becomes clinically significant. Nevertheless, high-level expression of *HSF1 *was an independent factor for recurrence in TNBC (Additional file 7).

### Amplifications of *HSP90AA1*, *HSP90AB1 *and *HSF1 *collectively defined a subpopulation of breast cancer samples with up-regulated HSP90 gene expression

We found a significant association between gene expression and copy number aberrations in *HSP90AA1*, *HSP90AB1, TRAP1 *and *HSF1 *(*P *< 1 × 10^-8^, ANOVA; Figure 2) and a trend for significant correlation in *HSP90B1 *(*P *< 1 × 10^-5^, ANOVA; Figure 2), indicating that high-level expression of HSP90 and HSF1 was driven by gene amplification. Although hemizygous deletion of HSP90 isoforms and *HSF1 *were found in 4.37% to 18.09% of breast cancer samples, homozygous deletion was uncommon. Only 1 of 481 (2%) breast cancer samples had two allele deletions on the *TRAP1 *coding region, and no patients carried a homozygous deletion of other HSP90 isoforms and HSF1, suggesting that loss of expression of HSP90 is a rare event in breast cancer.

**Figure 3:**
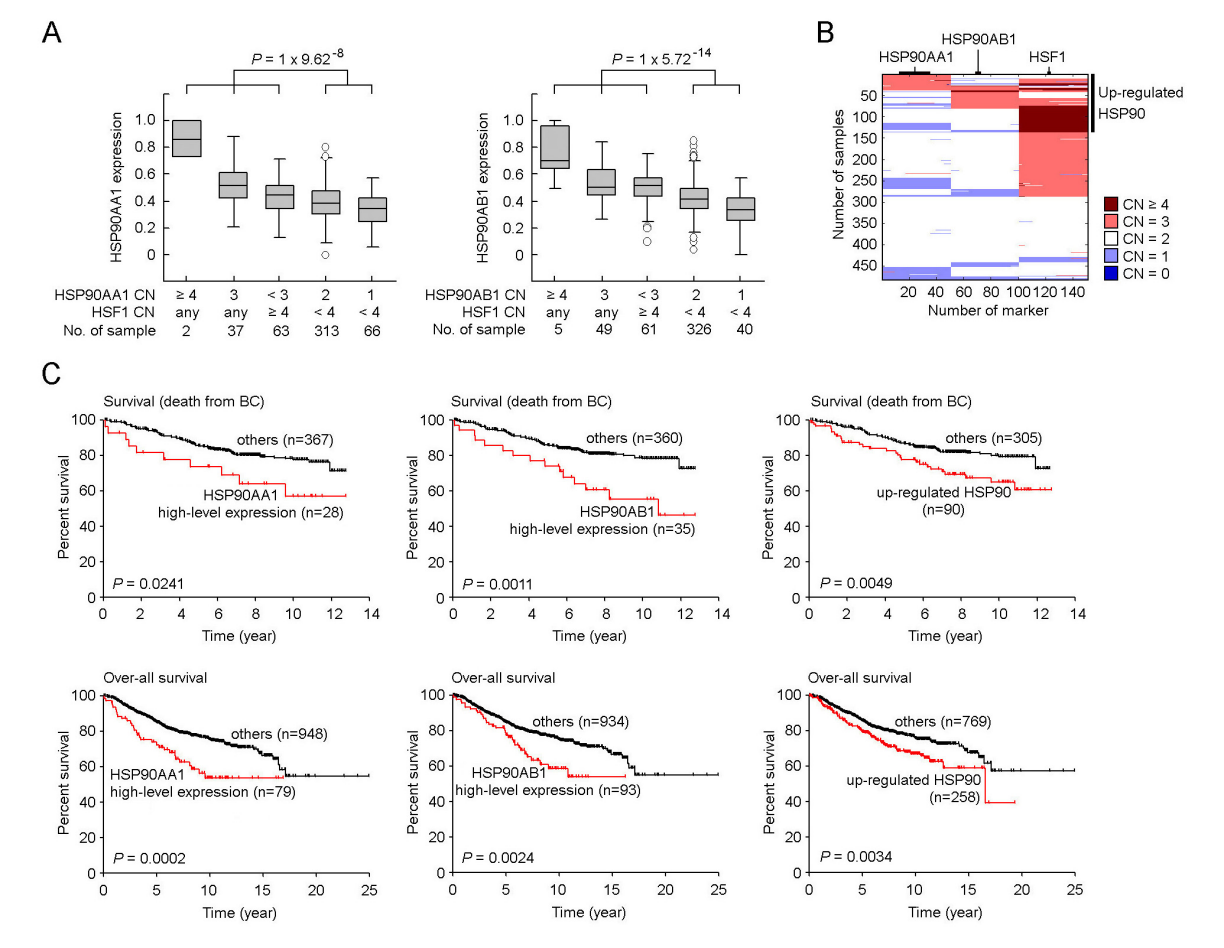
**Prognosis of up-regulated HSP90**. **(A) **Correlation between *HSP90AA1*, *HSP90AB1 *and *HSF1 *copy number aberrations and *HSP90AA1 *and *HSP90AB1 *expression. Differences between up-regulated HSP90 and others were assessed using the exact Mann-Whitney U test. Boxes represent the 25% to 75% quartiles, lines *in the boxes *represent the median level, whiskers represent the non-outlier range, and circles represent the outliers. **(B) **Distribution of *HSP90AA1*, *HSP90AB1 *and *HSF1 *copy number aberrations across 481 TCGA samples. **(C) **Prognosis of high-level expression of *HSP90AA1 *or *HSP90AB1*, and up-regulated HSP90. Kaplan-Meier estimates of disease specific survival (event of death from breast cancer) in 395 breast cancer patients (number of events, *n *= 83) and over-all survival in 1,027 breast cancer patients (number of events, *n *= 248). *P *values were calculated using log-rank Mantel-cox test. Tick marks indicate patients whose data were censored by the time of last follow-up.

We observed that 8% of breast cancer samples carried amplifications (both high-level and low-level amplifications, CN ≥3) of *HSP90AA1*, leading to a higher expression of *HSP90AA1*, compared with samples without *HSP90AA1 *amplifications (*P *= 7.67 × 10^-8^, *n *= 481, Mann-Whitney U Test; Figure 3A). Similarly, amplifications (CN ≥3) of *HSP90AB1 *were found in 11% of the population, and were correlated with significantly higher expression of *HSP90AB1 *(*P *= 1.02 × 10^-8^, *n *= 481, Mann-Whitney U Test, Figure 3A). Although amplification (CN ≥3) of *HSF1 *coding regions was a common event in the studied samples (54.1%), high-level amplification (CN ≥4) of *HSF1 *was found in 16% of the population, in which 75% of the samples did not have a co-amplification of either *HSP90AA1 *or *HSP90AB1 *(Figure 3B). Among the samples without amplifications of *HSP90AA1 *or *HSP90AB1*, high-level amplification of *HSF1 *was significantly correlated with higher expression of *HSP90AA1 *(*P *= 0.0052, *n *= 422, Mann-Whitney U Test) and *HSP90AB1 *(*P *= 4.5 × 10^-7^, *n *= 428, Mann-Whitney U Test), respectively (Figure 3A). Furthermore, amplification of *HSP90AA1 *and/or high-level amplification of *HSF1 *collectively represents a group of breast cancer samples with up-regulated *HSP90AA1 *mRNA expression (*P *= 9.62 × 10^-8^, *n *= 481, Mann-Whitney U Test, Figure 3A). Up-regulated *HSP90AB1 *mRNA expression was also seen in samples with amplification of *HSP90AB1 *and/or high-level amplification of *HSF1 *(*P *= 5.72 × 10^-14^, *n *= 481, Mann-Whitney U Test, Figure 3A).

On the other hand, we found that amplification of *HSP90AA1 *and *HSP90AB1 *was a predominant genomic feature of the highest 10% of *HSP90AA1 *(*P *= 0.0001, *n *= 481, Fisher's exact Test) and *HSP90AB1 *(*P *= 2.71 × 10^-6^, *n *= 481, Fisher's exact Test) expressing tumors. High-level amplification of *HSF1 *(CN ≥4) was significantly enriched in the samples with the highest 20% of *HSF1 *(*P *= 3.30 × 10^-10^, *n *= 481, Fisher's exact Test) expressing tumors. When samples with the highest 10% of *HSP90AA1 *and/or highest 10% of *HSP90AB1 *expressing tumors were combined with the highest 20% of *HSF1 *expressing tumors, this collective set of samples clearly captured the subpopulation of amplified HSP90 (*P *= 3.99 × 10^-25^, *n *= 481, Fisher's exact Test). Because high expression of *HSP90AA1, HSP90AB1 *and *HSF1 *was driven by amplification, and high-level amplification of *HSF1 *was associated with higher expression of HSP90 in un-amplified HSP90 samples, we defined up-regulated HSP90 as a collection of samples with the top 10% high expression value of *HSP90AA1 *and/or *HSP90AB1*, and the top 20% higher expression of *HSF1*. Using these definitions, up-regulated HSP90 accounted for 31% of the breast cancer population (Additional file 1) and up-regulated HSP90 was significantly correlated with higher expression of all HSP90 isoforms (*P *< 1 × 10^-8^, Mann-Whitney U test, Additional file 8).

### Up-regulated HSP90 was independently correlated with poor prognosis in HER2 negative breast cancer subtypes

To investigate the correlation of up-regulated HSP90 and poor breast cancer prognosis, we performed a univariate Kaplan-Meier survival analysis and a multivariate Cox Proportional-Hazards Regression (COXPH) survival analysis using other poor clinical outcome-associated clinical cofactors, such as tumor size, grade, nodal status, age, HER2, ER and PRstatus, as co-variants. We found that up-regulated HSP90 was significantly associated with a higher risk of death from breast cancer (*P *= 0.0049, *n *= 395, Figure 3B) and poor overall survival in a subset of 1,027 patients in which overall survival data were available (*P *= 0.0034, log-rank Mantel-cox test, Figure 3C). This poor prognosis phenotype was independent of clinical cofactors (*P *= 0.0062, *n *= 421, COXPH test, Table 3 and Additional file 9). Furthermore, we found that up-regulated HSP90 was significantly associated with a higher risk of recurrence and distant metastasis in TNBC and breast cancer with the HER2-/ER+ phenotype (Additional file 10). Up-regulated HSP90 was an independent factor that led to higher risk of death from breast cancer in the HER2-/ER+ breast cancer subtype (*P *= 0.0042, *n *= 421, COXPH test, Table 3), with a trend of significantly higher risk of distant metastasis in this subtype (Table 3). Particularly, up-regulated HSP90 independently increased risk of recurrence in TNBC (*P *= 0.0101, *n *= 421, COXPH test, Table 3; Additional file 9), and more than 70% of TNBC patients with up-regulated HSP90 had disease recurrence within eight years after initial treatment (Additional file 10).

**Table 3:** Prognosis of up-regulated HSP90 in different subtypes of breast cancer.

Subtype	Event phenotype	Kaplan-Meier survival analysis			COXPH survival analysis			
		*P*-value	HR (95%CI)	n	*P*-value	Co-efficiency	*P*-adjusted	n
All samples	Death	0.0034	1.57 (1.16-2.12)	1072	0.0007	0.5714	0.0062	421
HER2+	Death	0.3118	1.40 (0.73-2.71)	194	0.2564	0.7433	0.1405	63
	Recurrence	0.475	0.87 (0.58-1.29)	204	0.9528	-0.2160	0.6705	72
	Distant metastasis	0.2292	0.77 (0.50-1.18)	347	0.5383	-0.6461	0.2330	90
HER2-/ER+	Death	0.0148	1.71 (1.11-2.63)	506	0.0003	0.8373	0.0042	228
	Recurrence	0.0183	1.31 (1.05-1.65)	832	0.1790	0.2077	0.3054	361
	Distant metastasis	0.0002	1.65 (1.27-2.15)	1223	0.0098	0.4050	0.0705	415
TNBC	Death	0.0604	1.76 (0.98-3.19)	282	0.5693	0.2586	0.5869	105
	Recurrence	0.0002	2.29 (1.49-3.52)	285	0.0008	0.9924	0.0101	122
	Distant metastasis	0.0195	1.60 (1.08-2.37)	516	0.6722	-0.0323	0.9390	158

CI: confidence interval; HR: hazard ratio; n: number of samples

## Discussion

The phenotypic heterogeneity of cancer arises as a consequence of numerous genetic abnormalities (such as somatic mutations and chromosomal aberrations) acquired during tumor development and results in the formation of a disease that is enormously complex and highly variable between patients. An ability to dissect this heterogeneity will facilitate a deeper understanding of the relevance of these alterations for disease phenotypes by which to develop rational therapeutic strategies that can be matched with the characteristics of the individual patient's tumor. In fact, this has already been achieved in some instances of breast cancer where HER2-positive tumors are treated with trastuzumab or lapatinib, and ER-positive tumors are treated with anti-hormonal therapy. To identify additional molecular characteristics for a more effective treatment of breast cancer, an approach to rapidly and efficiently leverage available breast cancer genomic data and correlate both genetic and clinical features and outcomes is urgently needed.

Gene expression profiling has become a major tool for the study of breast cancer and substantial amounts of data are available from public databases. To date, microarray data from more than 6,000 primary breast cancer samples have been posted on the Gene Expression Omnibus (GEO) database. To capture the complexity of breast cancer heterogeneity and pinpoint molecular factors that can be therapeutically targeted, we compiled a large collection of breast tumor gene expression data (*n *= 4,010) derived from 23 datasets that were published from October 2005 to February 2011, including subsets of samples in which clinical prognosis data were available. We identified a series of genes whose high-level expression increased the risk of death from breast cancer, which may be exploited to improve the effectiveness of clinical intervention in this disease. We found that HSP90AA1 and HSP90AB1, two cytoplasmic HSP90 isoforms, were among the most significant factors of poor prognosis in different breast cancer subtypes. As one of the most abundant proteins in malignant cells and a key factor that stabilizes oncoproteins involved in cancer growth and survival, our results suggest that increased HSP90 expression may play an important role in promoting aggressive breast cancer phenotypes. Furthermore, we found that highly expressed *HSP90AA1*, *HSP90AB1 *and *HSF1 *were driven by somatic amplifications, which collectively were found in approximately 30% of tumors, which we classified as up-regulated HSP90. We revealed that up-regulated HSP90 was significantly associated with risk of death from breast cancer among patients with HER2-/ER+ breast cancer, and greatly increased the chance of disease recurrence in TNBC, and these interactions were independent of clinical variables.

Perhaps the most significant challenge presented by the complexity of breast cancer is the ability to design and develop therapeutic regimens that can match the characteristics of the individual patient's tumor -- to achieve the goal of personalized cancer treatment. In addition to the well credentialed or previously described genes HER2 and GRB7, we found additional factors associated with an increased risk of death from breast cancer, such as CUTL1 [35], CTTN [36] and GINS2 [37] that have been previously linked with poor prognosis of breast cancer. This reflects the nature of cancer heterogeneity in which multiple mutations and alterations generate the cancer phenotype. The development of therapeutic strategies that can completely and precisely match the complexity of breast cancer with equally complex combinations of regimens will be clinically challenging, particularly considering the need to utilize combinations of drugs that must be shown to be safe when combined together. A more practical approach would prioritize the more universal molecular factors associated with aggressive behavior and poor prognosis, upon which more general therapeutic regimens can be developed for use in combinations. Previous reports have indicated that high expression of HSP90, assessed by protein expression analysis, is associated with a poor overall prognosis in breast cancer patients [24]. High HSP90 expression was associated with high expression of HER2 and ER, large tumors, high nuclear grade, and lymph node involvement [9]. Our results demonstrated that up-regulation of multiple isoforms of HSP90 in primary breast cancer were independent poor prognosis factors, indicating that HSP90 targeted therapies in combination with cytotoxic chemotherapies or other targeted agents, may improve diagnosis and treatment of highly aggressive breast cancers.

Because HSP90 is a key component of oncogenic signaling, an increasing number of candidate HSP90 inhibitors have been developed and evaluated, both in preclinical models and in clinical trials. Although HSP90 inhibitors have exhibited clinical activity in the treatment of breast and other cancers, targeting HSP90 alone generally results in cytostatic rather than cytotoxic effects on tumors. In the majority of patients, disease progression occurs following cessation of treatment with an HSP90 inhibitor [8]. Our results suggest that up-regulated HSP90 might not be an independent poor prognosis factor among patients with HER2-positive breast cancer, as no statistically significant correlation was observed between poor survival and high-level expression of any HSP90 isoforms, which is consistent with the previous finding that the most common clinical response in patients with HER2-positive breast cancer who received HSP90 monotherapy is stable disease. In contrast, multiple studies using cell-based or various tumor xenograft models of breast cancer have shown a large degree of synergy by combining HSP90 inhibitors with therapies targeting HER2 (such as trastuzumab or lapatinib) [38,39]. Indeed, in animal xenograft models, tumors often do not immediately re-grow upon drug withdrawal, and often significant tumor regression can be observed[17]. In clinical trials, chronic administration of the majority of HSP90 inhibitors is well tolerated by humans, with manageable toxicity. At first glance this seems surprising given the essential role of the protein in numerous normal cellular processes; however, the apparent lack of toxicity of HSP90 inhibitors may be related to the recent realization that cancer cells are addicted to HSP90--a prime example of tumor cell non-oncogene addiction [8]. This may provide a sufficiently large therapeutic window for the safe use of HSP90 inhibitors in cancer. Additionally, there is evidence that oncogenic clients can alter the conformation of HSP90. Several inhibitors of the protein have been developed that only recognize this activated conformation [40,41] suggesting an even greater therapeutic index.

TNBC has been considered a more aggressive breast cancer subtype with a higher rate of distant recurrence and a poorer prognosis [19,20]. We found that increased expression of each of the HSP90 isoforms was correlated with a higher risk of recurrence and more than 70% of patients with up-regulated HSP90 experienced disease recurrence within eight years after initial treatment, suggesting that TNBC patients might benefit from therapies that target multiple HSP90 isoforms, such as HSP90AA1, HSP90AB1 and TRAP1. In fact, in pre-clinical models, TNBC have been sensitive to Hsp90 inhibitors [22,23]. Similar to HER2 positive tumors, TNBCs were sensitive to Hsp90 inhibition through down-regulation of components of the Ras/Raf/MAPK pathway in preclinical and *in vitro *studies [23]. Furthermore, our results demonstrated that up-regulated HSP90 was also a significant prognostic factor in HER2-/ER+ breast cancers, suggesting a broad application of HSP90 targeted therapies in the 80% of breast cancers that do not over-express HER2. In addition, other hormone receptors, such as androgen receptor, utilized HSP90, which provides a rationale for the use of HSP90 inhibitors and AR antagonist in the subset of AR+ breast cancers. Given the fact that HSP90 is one of the most abundant proteins in breast cancer cells, and HSP90 has been proposed as a potential therapeutic target for other cancers, including non-small cell lung cancer [42], our results indicate that HSP90 is an important oncogenic signaling node in breast cancer, whose high expression is associated with aggressive behavior and poor prognosis of breast cancer. Diagnostic and therapeutic strategies directed to cancer expressing high levels of HSP90 are warranted.

## Conclusions

High-level expression of two cytoplasmic HSP90 isoforms, HSP90AA1 and HSP90AB1, were predominantly driven by gene amplifications. Using clinical parameters that were associated with poor clinical outcome, such as tumor size, grade, nodal status, age, HER2, ER and RP status, we demonstrated that high-level expressions of *HSP90AA1 *and *HSP90AB1 *were independent poor prognosis factors affecting triple-negative and HER2-/ER+ breast cancer subtypes. Furthermore, up-regulated HSP90 that was defined as a collection of *HSP90AA1*, *HSP90AB1 *and *HSF1 *amplifications was one of the most significant factors that independently associated with risk of death from breast cancer, and greatly increased the incidence of recurrence and distant metastasis in triple negative and HER2-/ER+ breast cancer subtypes.

## Abbreviations

ANOVA: analysis of variance; CNAs: copy number aberrations; COXPH: Cox Proportional-Hazards Regression survival analyses; ER: estrogen receptor; GEO: Gene Expression Omnibus; HER2: human epidermal growth factor receptor 2; HSF1: HSP transcriptional factor 1; HSP90: heat shock protein 90; PR: progesterone receptor; RMA: Robust Multi-array Average; TCGA:The Cancer Genome Atlas; TNBC: triple negative breast cancer.

## Competing interests

Dr. Timothy Haystead was founding scientist of Serenex Inc, Durham NC. Dr. Timothy Haystead declares a technology has been developed for the detection of up regulated/activated Hsp90 at the protein level in tumors. This technology has been disclosed to Duke University in accordance with its patenting policies. He is a tenured Associate Professor at Duke University and receives an annual salary from this organization. All other co-authors declare no competing interests.

## Authors' contributions

QC, TH, LMN and HKL designed the study. QC and JTC contributed to the data analyses. JG, NLS and HKL provided expertise in clinical breast oncology. All authors contributed to the preparation of the manuscript. All authors have read and approved the final manuscript for publication.

## Supplementary Material

Additional file 1 **Clinical data of 4,010 breast cancer samples and expression of selected genes**. This table lists clinical data that was downloaded from NCBI GEO database, and normalized expression signal of HER2 (216836_s_at), ER (205225_at), PR (208305_at), HSP90AA1 (214328_s_at), HSP90AB1 (214359_s_at), HSP90B1 (200598_s_at) and HSF1 (213756_s_at), as well as defined up-regulated HSP90. Click here for file

Additional file 2 **Heatmaps**. These heatmaps show the expression patterns in the data before **(A) **and after **(B) **normalization. The rows contain the 1,000 genes that exhibit the highest variance in gene expression profile across the original data set. The columns contain the samples in the data sets provided. The genes and samples are in the same order in both heatmaps. Warm colors indicate high expression of the gene and cool colors indicate low expression. Click here for file

Additional file 3 **Distribution of HER2, ER and PR mRNA expression and its correlation with IHC measure molecular status**. This figure shows **(A) **histograms of HER2, ER and PR mRNA expression in 4,010 breast cancer samples and **(B) **the correlation between mRNA expression and IHC status. Differences between positive and negative groups were assessed using the exact Mann-Whitney U test. Boxes represent the 25% to 75% quartiles, lines *in the boxes *represent the median level, whiskers represent the non-outlier range, and circles represent the outliers. Click here for file

Additional file 4 **Expression defined breast cancer subtypes**. This figure shows **(A) **Bimodal selection for HER2, ER and PR cutoff according to the distribution of expression values stratified by IHC/biochemical status. **(B) **Distribution of HER2, ER and PR mRNA expression in combined dataset. **(C) **Distant metastasis-free survival analyses were stratified according to IHC/biochemical status or expression derived status using samples with available IHC/biochemical status and outcome data. Tick marks in Kaplan-Meier Estimates distant-metastasis free survival indicate patients whose data were censored by the time of last follow-up or owing to death. *P *values were calculated using log-rank Mantel-cox test. Click here for file

Additional file 5 **Breast cancer poor prognosis associated gene**. This table lists breast cancer poor prognosis -ssociated genes. Cox-regression survival analyses were performed using 395 samples in which event of death from breast cancer was available. Analysis of variance (ANOVA) was performed to test for an association between copy numbers and gene expression using 481 TCGA breast cancer samples. Click here for file

Additional file 6 **Genome scans for poor prognosis associated gene**. This figure shows the correlation between copy number aberrations and gene expression of identified genes that were associated with breast cancer poor prognosis. Upper panel shows percentage of amplification (low-level and high-level amplification) and deletion (homozygous and hemizygous deletion) at each detected chromosome region in a group of 481 breast cancer patients. Bottom panel shows correlation between CNA and mRNA expression of poor prognosis associated genes that were identified from each chromosome. Analysis of variance (ANOVA) was performed to test for association between copy numbers and gene expression. Click here for file

Additional file 7 **Prognosis of HSP90 and HSF1 in different breast cancer subtypes**. This table lists the results of survival analyses. Breast cancer subtype specific disease-specific survival (dss, event of death from breast cancer), over-all survival (os), recurrence-free survival (rfs), and distant metastasis-free survival (dmfs) were assessed using Cox-regression survival analysis, Kaplan-Meier Estimates survival analysis and Cox Proportional-Hazards (COXPH) Regression survival analysis. Click here for file

Additional file 8 **Correlation between HSP90 and HSF1 mRNA expression and up-regulated HSP90**. This figure shows HSP90 and HSF1 expression difference between samples defined as up-regulated HSP90 and not up-regulated HSP90. Differences for each pairwise comparison were assessed by the Mann-Whitney U test. Boxes represent the 25% to 75% quartiles, lines *in the boxes *represent the median level, whiskers represent the non-outlier range, and circles represent the outliers. Click here for file

Additional file 9 **Cox univariate and multivariate analyses of up-regulated HSP90**. This table lists **the **results of Cox Proportional-Hazards (COXPH) Regression survival analyses of up-regulated HSP90 using samples where the entire set of clinical data was available. Click here for file

Additional file 10 **Prognosis of up-regulated HSP90 in different breast cancer subtypes**. This figure shows Kaplan-Meier estimates curve of up-regulated HSP90 in different breast cancer subtypes. Number of recurrence events: TNBC, *n *= 142; HER2-/ER+, *n *= 331; HER2+, *n *= 112. Number of distant metastasis events: TNBC, *n *= 133; HER2-/ER+, *n *= 260; HER2+, *n *= 111. Tick marks in Kaplan-Meier estimates of recurrence-free survival and distant-metastasis free survival indicate patients whose data were censored by the time of last follow-up or owing to death. *P *values were calculated using log-rank Mantel-cox test. Click here for file
