# Supplementary material for: Amplification and high-level expression of heat shock protein 90 marks aggressive phenotypes of human epidermal growth factor receptor 2 negative breast cancer
Source: Breast Cancer Res. 2012 Apr 17;14(2):R62. doi: 10.1186/bcr3168 (PMC3446397; doi:10.1186/bcr3168)
Supplement: Additional file 1 — Clinical data of 4,010 breast cancer samples and expression of selected genes. This table lists clinical data that was downloaded from NCBI GEO database, and normalized expression signal of HER2 (216836_s_at), ER (205225_at), PR (208305_at), HSP90AA1 (214328_s_at), HSP90AB1 (214359_s_at), HSP90B1 (200598_s_at) and HSF1 (213756_s_at), as well as defined up-regulated HSP90. [file bcr3168-S1.PDF]

Additional file 1. Clinical data of 4010 breast cancer samples and expression of selected genes.

GSM	HER2 ihc	ER ihc	PR ihc	HER2 exp	ER exp	PR exp	nodal	grade	size	age	dss Ind	os year	os Ind	rfs year	rfs Ind	dmfs year	dmfs Ind	216836_s_at	205225_at	208305_at	214328_s_at	214359_s_at	200598_s_at	201391_at	213756_s_at	up-regulated HSP90
GSM107072				0	1	0		2			1	4.14	1	3.82	1			9.3149	10.6293	4.1407	12.1891	10.0517	8.7092	8.7638	5.3171	0
GSM107073				0	1	1		3			0	8.15	0	8.15	0			9.3113	11.8244	5.5700	12.1729	10.8057	9.0283	9.7757	5.9163	1
GSM107074				0	1	0		1			0	2.22	1	2.22	0			9.9733	11.6904	4.4932	12.0555	10.2988	8.7524	8.8863	5.7193	1
GSM107075				1	1	0		2			0	8.23	0	8.23	0			12.0215	10.8631	3.9548	12.6083	10.2216	8.2391	8.9384	5.2981	0
GSM107076				0	1	0					0	5.55	1	5.55	0			9.6290	10.6572	4.1288	11.6572	10.3086	8.9296	8.9599	5.1556	0
GSM107077				0	1	1		3			0	8.30	0	8.3	0			9.5244	11.8776	4.9232	12.4327	11.3351	10.1123	9.8680	5.8078	1
GSM107078				0	1	0		2			0	4.42	1	4.42	0			9.7983	11.2343	4.1640	11.5266	10.4309	8.4797	8.8546	5.2073	0
GSM107079				0	1	0		1			0	8.07	0	8.07	0			10.0095	10.3121	4.0990	12.1228	11.0191	9.2062	9.3707	4.9701	1
GSM107080				0	0	0		3			0	6.38	0	6.38	0			8.8683	6.0048	4.1956	11.6567	10.4879	7.9203	8.8907	4.7861	0
GSM107081				0	0	0		3			0	8.13	0	8.13	0			10.9904	7.7543	4.2734	12.1052	10.5106	8.4717	9.0867	4.7603	0
GSM107082				0	0	0					1	5.51	1	4.08	1			9.0298	7.1422	4.4124	11.6880	10.8661	8.7726	8.6248	5.4960	0
GSM107083				0	1	1		2			0	8.11	0	8.11	0			9.7628	9.6295	6.3372	12.4139	10.7523	8.8266	9.5805	5.2938	0
GSM107084				0	1	0		3			1	5.47	1	3.47	1			8.7370	10.1806	4.3926	12.6040	9.1880	7.7280	9.8054	4.9927	0
GSM107085				0	1	1		3			0	6.82	0	0.56	1			9.8052	12.1247	4.8572	11.8230	10.4257	7.6244	9.1505	4.8957	0
GSM107086				0	1	0		2			1	6.25	1	5.53	1			9.7583	9.8203	4.7023	12.3649	9.6243	7.7977	9.4812	4.9342	0
GSM107087				0	1	0		2			0	7.71	0	7.71	0			8.5926	10.4664	4.2961	12.5067	10.8618	8.8492	9.0488	5.2197	0
GSM107088				0	1	1		2			1	6.22	1	4.44	1			9.3708	11.2772	6.6506	12.9120	10.4570	8.6913	9.6773	5.4483	1
GSM107089				0	1	1		3			1	3.06	1	1.39	1			8.8798	9.7651	5.8407	12.1973	10.3588	8.0387	8.9772	4.9524	0
GSM107090				0	0	0		3			0	5.87	1	5.87	0			8.6930	8.8021	4.3314	11.9366	9.0886	7.3781	8.9970	4.9160	0
GSM107091				0	1	1		2			0	7.86	0	7.86	0			10.5020	12.3160	5.6716	12.2563	10.3005	8.5547	8.8181	4.6465	0
GSM107092				0	1	1		2			1	3.85	1	2.92	1			9.4207	11.9441	5.4727	12.7421	9.6625	6.4475	9.2707	4.6924	0
GSM107093				0	1	1		3			1	2.65	1	1.09	1			9.5000	11.9168	5.0374	12.7784	11.0457	9.0237	9.1249	4.6464	1
GSM107094				1	1	0		3			1	5.63	1	4.36	1			12.7881	10.9881	4.0834	12.5085	11.2491	9.9734	9.8893	5.0286	1
GSM107095				0	1	1		2			0	7.61	0	7.61	0			9.4383	11.1887	5.0992	12.8285	9.9608	7.4317	10.0134	4.9861	1
GSM107096				0	1	1		1			0	7.55	0	7.55	0			9.6353	10.7870	6.9911	12.2545	10.5993	7.5877	9.3908	5.0889	0
GSM107097				0	1	1		2			1	3.18	1	1.19	1			9.6521	10.9875	5.1060	11.9990	9.5811	8.0870	9.2089	5.1666	0
GSM107098				0	1	0		1			0	8.03	0	8.03	0			9.8239	9.9459	4.5469	12.1385	10.4868	8.6701	8.9739	4.6402	0
GSM107099				0	1	0		2			0	7.31	0	5.16	1			9.1836	11.1924	3.9648	12.9076	10.0844	7.9394	9.5532	5.2712	1
GSM107100				1	0	0		3			0	7.65	0	7.65	0			12.4705	7.9180	4.5306	12.4504	10.5221	7.5266	9.1658	5.3434	0
GSM107101				0	0	0		2			0	7.38	0	7.38	0			9.3206	6.5733	4.1282	11.7027	9.7234	7.1325	8.5648	5.2182	0
GSM107102				0	0	0		3			0	7.29	0	7.29	0			10.5288	5.8427	4.2553	12.2418	10.3078	8.1877	8.9853	5.0662	0
GSM107103				0	1	0		2			0	8.03	0	8.03	0			9.7351	11.5822	4.3171	11.4102	10.1395	8.6739	8.8868	4.6213	0
GSM107104				0	1	1		1			0	8.03	0	8.03	0			10.1192	11.3229	7.1784	11.9283	10.5179	8.8392	9.2120	4.8054	0
GSM107105				0	1	0		1			0	7.93	0	6.65	1			9.9047	11.6235	4.1025	12.3852	10.5567	8.0140	8.9435	4.6018	0
GSM107106				0	1	0		1			0	7.73	0	7.73	0			9.1465	10.0414	4.8005	11.9049	9.5202	9.5468	8.7761	4.9660	0
GSM107107				1	1	1		1			0	8.20	0	8.2	0			11.5367	11.0767	5.3046	12.0816	10.9350	8.6000	9.3951	5.8203	1
GSM107108				0	1	1		3			0	7.59	0	7.59	0			9.5223	11.6056	6.1671	12.1956	9.7643	8.2899	8.8793	4.5608	0
GSM107109				1	0	0		3			0	7.81	0	7.81	0			12.3039	7.0664	4.2697	12.2002	10.2876	8.6203	8.9782	4.9161	0
GSM107110				0	1	1					0	8.48	0	8.48	0			9.1194	11.9987	4.8277	12.8243	10.6780	8.7159	9.4211	5.4288	1
GSM107111				0	0	0		1			0	7.95	0	7.95	0			9.6816	8.7179	4.2867	11.5223	9.8795	8.4564	8.4767	4.9779	0
GSM107112				0	1	0		2			0	2.24	1	2.24	0			9.6299	10.3704	4.7535	11.6672	10.0683	7.7518	8.6024	4.6671	0
GSM107113				0	1	0		3			0	7.28	0	7.28	0			9.4666	10.7188	4.5271	12.7200	10.7690	8.5814	9.2816	5.4829	0
GSM107114				0	1	1		2			0	7.96	0	7.96	0			10.4406	9.8534	5.4261	12.5218	10.9686	9.5610	8.9249	5.2983	1
GSM107115				0	0	0		3			0	6.86	0	6.86	0			8.9924	6.7666	4.6272	12.4732	10.4931	8.1996	8.1631	5.3176	0
GSM107117				0	1	0		2			0	7.03	0	7.03	0			10.4717	10.7130	3.6041	12.2408	10.6309	8.4288	8.7996	4.9323	0
GSM107118				0	1	0		2			0	8.46	0	8.46	0			9.7405	9.6690	3.7152	12.3498	9.3642	8.3233	9.1143	4.8938	0
GSM107119				0	1	1		2			0	7.84	0	7.84	0			9.5304	11.0910	5.8387	11.8782	9.5543	8.4101	8.4844	5.0743	0
GSM107120				0	1	0		2			0	6.13	0	6.13	0			9.1983	10.6922	3.6335	12.0369	10.0871	8.6490	8.6980	5.7956	1
GSM107121				0	1	1		3			0	7.21	0	7.21	0			8.3098	10.2337	4.8660	11.8132	9.9890	8.8126	8.7397	4.6792	0
GSM107122				0	0	0		2			0	8.13	0	8.13	0			9.1826	8.1237	3.9391	12.2398	10.4190	8.5549	8.9712	5.0336	0
GSM107123				1	1	0					0	7.65	0	7.65	0			12.2178	13.1106	4.6384	12.1318	10.8608	9.2117	8.9002	5.7911	1
GSM107124				0	1	0		2			0	7.07	0	7.07	0			8.7165	9.5762	4.6693	11.8431	10.4663	9.0276	8.7310	4.6521	0
GSM107125				1	0	0		2			1	4.72	1	3.99	1			12.7580	8.2031	4.3573	12.0969	10.5353	9.1336	8.4149	4.8038	0
GSM107126				0	1	1		1			0	7.55	0	7.55	0			10.5582	11.7576	7.0716	12.2940	11.0887	9.1839	9.9005	5.7321	1
GSM107127				0	1	1		1			0	7.75	0	7.75	0			9.3630	12.6060	6.9486	11.7838	10.4077	8.6947	8.8612	4.9529	0
GSM107128				0	1	0		2			0	8.08	0	8.08	0			9.7032	11.3554	4.1198	11.9278	10.0603	8.8147	8.8997	4.8985	0
GSM107129				0	0	0		3			1	2.38	1	1.55	1			8.3285	7.8742	4.3691	11.9387	10.4929	8.8622	8.1237	4.9177	0
GSM107130				0	1	1					0	8.40	0	8.4	0			9.8842	12.3387	5.2180	12.0442	9.8023	8.3543	8.7326	5.0083	0
GSM107131				0	0	0		3			0	7.54	0	7.54	0			8.5321	8.9065	4.8050	11.8863	10.3379	8.8540	8.6553	4.6286	0
GSM107132				0	1	1		1			0	7.28	0	7.28	0			10.1719	10.8647	6.2586	11.6513	10.2231	8.4818	8.8559	4.7458	0
GSM107133				0	1	0		2			0	7.73	0	7.73	0			9.6826	12.4101	4.4689	11.5834	9.9498	8.2974	9.3332	4.6855	0
GSM107134				1	1	0		3			1	3.00	1	1.85	1			12.6732	11.9697	4.7704	12.5319	10.6509	8.3531	9.0816	4.6660	0
GSM107135				0	0	0		3			0	6.31	0	6.31	0			8.9508	7.4695	4.4432	12.0940	10.1041	9.0961	8.6919	4.7777	0
GSM107136				0	1	0		2			0	8.30	0	8.3	0			9.4771	10.3561	4.2542	12.0961	9.9387	8.8806	8.7380	4.7572	0
GSM107137				0	1	1		2			0	7.07	0	7.07	0			10.5908	11.0038	5.9298	11.9391	11.0718	8.9257	8.8533	5.3239	1
GSM107138				0	1	0		3			1	6.40	1	3.15	1			10.7671	10.1153	4.0712	12.3036	11.4210	8.2895	9.0125	5.2127	1
GSM107139				0	1	0		1			0	8.15	0	8.15	0			10.2887	9.8955	4.1784	11.8992	10.4738	8.3732	8.9406	4.8432	0
GSM107140				0	0	0					0	8.24	0	8.24	0			9.1439	6.1445	4.7386	12.2119	9.8940	8.5631	8.6497	4.1900	0
GSM107141				0	0	0		3			0	6.02	0	6.02	0			9.0325	7.2242	4.1297	12.5206	10.6369	8.6154	9.5699	5.3126	0
GSM107142				0	1	1		3			0	5.85	0	5.85	0			10.9559	10.3723	5.7401	11.8351	10.0357	8.1720	8.8626	5.3717	0
GSM107143				0	1	1		2			0	5.96	0	5	1			9.3098	10.6125	5.2075	12.1883	10.5732	8.6799	9.4254	4.9109	0
GSM107144				1	0	0					0	5.81	0	5.81	0			11.7814	9.2088	4.0610	12.3203	11.0073	9.0674	9.2408	4.8730	1
GSM107145				0	1	1		2			0	5.86	0	5.86	0			8.9526	10.1950	5.6534	11.9479	10.5133	9.0613	8.9731	4.9486	0
GSM107146				0	1	0					0	5.75	0	5.75	0			10.4570	9.6842	3.8126	12.3302	10.0980	8.7756	9.3782	4.9489	0
GSM107147				0	1	0		1			0	5.71	0	5.71	0			10.5191	9.9612	3.9555	12.4972	11.0682	8.8760	9.2008	4.9746	1
GSM107148				0	1	0		2			0	5.63	0	5.63	0			10.6064	10.2926	3.7815	11.8512	10.1533	10.6712	8.6175	5.2017	0
GSM107149				0	1	1		2			0	5.68	0	5.68	0			10.2611	11.5564	8.7588	12.2858	10.7585	9.3052	9.4458	5.2091	0
GSM107150				0	1	0		2			0	7.99	0	7.99	0			10.0115	10.5973	4.5791	12.1453	10.8004	9.0312	9.0295	5.2777	0
GSM107151				0	1	1		3			0	5.56	0	5.56	0			10.4453	13.1113	5.1629	12.8560	11.3219	9.1682	9.4884	5.4340	1
GSM107152				0	1	1		3			1	4.61	1	1.61	1			9.5884	11.7174	4.8577	12.0109	10.7335	8.4533	8.6462	5.4669	0
GSM107153				0	1	0		1			0	5.69	0	5.69	0			9.9998	11.0620	4.7696	12.1318	10.5313	9.2355	9.1642	5.7321	1
GSM107154				0	1	0					0	5.79	1	5.79	0			10.0411	12.2177	4.7216	12.0427	9.5977	8.5360	8.8881	5.0384	0
GSM107155				0	1	0		2			1	1.73	1	1.04	1			9.5917	11.7882	4.6557	12.1601	10.2709	8.8672	8.7508	4.8336	0
GSM107156				0	1	0		2			0	8.33	0	8.33	0			9.1056	9.6686	4.3838	12.3370	10.8399	8.6419	8.7797	4.9072	0
GSM107157				0	1	1		3			0	6.03	0	6.03	0			10.1053	12.5200	4.9675	12.2807	10.0806	9.4583	8.7760	4.7805	0
GSM107158				0	1	1		1			0	4.51	1	4.51	0			7.9995	11.2366	4.8975	11.5281	10.3964	9.3400	8.9498	5.2295	0
GSM107159				0	1	0		2			0	5.86	0	5.86	0			9.7635	9.6810	4.3743	12.0376	10.0702	9.1234	8.8604	4.6457	0
GSM107160				0	1	0		1			0	5.56	0	4.3	1			8.6430	10.9579	4.3368	12.4532	10.5438	9.0808	8.6632	5.3555	0
GSM107161				0	1	1		3			0	7.92	0	7.92	0			8.8027	11.3628	6.1534	11.5615	10.5249	8.3368	8.9679	5.4618	0
GSM107162				0	1	1		2			0	6.52	0	6.52	0			8.6362	11.2912	6.4499	11.9697	9.5113	7.4010	9.1316	4.6365	0
GSM107163				0	0	1		3			0	7.42	0	7.42	0			10.2313	9.2691	5.0276	12.4242	8.9766	7.4671	10.3201	6.3981	1
GSM107164				0	1	0					0	6.46	0	6.46	0			9.3982	10.2672	3.9192	12.0954	10.1604	7.7775	9.0149	5.0272	0
GSM107165				0	0	1		2			0	8.43	0	8.43	0			8.8845	9.3374	5.1695	12.1137	10.0052	9.0751	9.0594	4.9299	0
GSM107166				0	0	0		2			1	6.72	1	6.24	1			7.4608	6.3794	4.3387	12.3117	9.6030	9.3635	9.2343	5.4251	0
GSM107167				0	1	1		2			0	7.28	0	3.89	1			9.3912	9.8245	5.9056	11.6614	9.8117	8.5336	8.9882	4.9017	0
GSM107168				0	1	1		3			0	7.96	0	4.27	1			9.2620	9.9725	5.6518	11.8959	10.3066	8.9126	9.5401	5.4702	0
GSM107169				0	0	0		2			0	7.59	0	7.59	0			9.8530	9.3371	4.4949	12.2909	10.3172	8.9247	9.2462	5.5641	0
GSM107170				0	0	0		2			1	1.21	1	0.23	1			9.2555	8.2830	4.2499	12.2389	9.3395	8.8946	8.9885	5.0164	0
GSM107171				0	1	1		2			0	7.51	0	7.51	0			9.4287	10.4040	5.8934	12.2804	9.6233	8.8523	9.9815	5.2119	0
GSM107172				0	1	0		2			0	7.67	0	7.67	0			8.7332	10.2020	4.6004	11.8734	10.0560	9.2720	8.8417	5.0737	0

GSM107173	0	1	0	1	0	8.30	0	8.3	0			9.9167	10.8183	3.8305	12.0289	10.0768	8.6155	8.6889	5.0458	0
GSM107174	0	1	0	2	0	7.78	0	7.78	0			9.6096	9.7652	4.4124	11.6689	10.0707	8.5986	9.1941	4.8872	0
GSM107175	0	0	0	3	0	7.89	0	7.89	0			9.1381	8.3597	4.5629	12.0872	9.9796	9.2504	8.8345	4.9208	0
GSM107176	0	1	0	1	0	7.44	0	7.44	0			10.1826	9.8079	4.0830	12.2247	10.2160	9.7178	8.7585	5.0874	0
GSM107177	0	1	0	1	0	5.95	0	5.95	0			10.5757	10.6798	3.9261	11.4923	10.2192	8.6859	8.8842	5.5654	0
GSM107178	0	1	0	2	1	2.83	1	2.78	1			10.9761	12.9345	4.7555	12.3699	10.8917	9.0324	8.7728	5.6429	1
GSM107179	0	1	0	2	0	6.26	0	6.26	0			9.5578	13.1233	4.4604	12.1253	11.1822	8.2063	9.3641	5.3386	1
GSM107180	0	1	1	1	0	8.32	0	8.32	0			10.6550	12.3092	5.1457	12.3054	11.0914	8.6056	9.2082	5.3866	1
GSM107181	1	1	0	3	1	4.83	1	3.86	1			12.4805	10.5466	4.6882	12.2280	10.2194	9.0862	9.2734	5.3359	0
GSM107182	0	1	0		0	6.17	0	6.17	0			9.6478	10.6943	4.4462	11.9391	10.1088	8.3845	8.6951	4.9379	0
GSM107183	0	0	0	3	0	8.13	0	8.13	0			8.4318	7.2078	4.7444	12.4214	10.2028	9.2896	9.0312	5.1502	0
GSM107184	0	1	0	1	0	5.95	0	5.95	0			9.9130	10.0448	3.8445	12.1115	10.0758	8.6467	8.8607	4.9056	0
GSM107185	0	0	0	2	0	5.98	0	5.98	0			9.8075	8.6957	3.5231	12.0372	10.5735	8.4876	8.9179	5.0495	0
GSM107186	0	0	0	2	0	6.05	0	6.05	0			7.5390	5.9169	3.8795	11.9189	9.8058	8.8074	8.6072	4.8207	0
GSM107187	0	1	0	1	0	6.76	0	6.76	0			8.9974	10.8562	4.4550	12.1744	9.4006	8.8261	8.3481	4.8349	0
GSM107188	0	1	0	2	0	8.32	0	8.32	0			10.4409	9.7819	3.7047	11.6237	10.2262	8.8254	8.6640	4.8610	0
GSM107189	1	1	1	2	0	6.55	0	6.55	0			11.5428	9.9710	5.0293	12.2864	9.7049	7.6646	9.2681	5.2720	0
GSM107190	0	1	0	1	0	7.94	0	7.94	0			9.4488	10.0680	4.3149	12.0411	9.9895	8.3858	8.6473	4.8221	0
GSM107191	0	0	0	3	0	6.55	0	6.55	0			10.1938	7.7195	4.4570	12.5287	10.8950	9.8785	8.9035	5.5332	0
GSM107192	1	1	1	3	0	7.05	0	7.05	0			11.2231	11.5852	5.1807	12.4250	10.5358	9.2565	9.1184	5.5139	0
GSM107193	1	0	0	3	0	6.40	0	6.4	0			12.1905	7.6112	4.4943	12.4336	10.1755	9.4789	9.0567	6.4904	1
GSM107194	0	1	0	3	0	7.40	0	7.4	0			10.4454	11.4482	4.5298	12.3626	11.0363	9.4842	9.2592	6.2522	1
GSM107195	0	0	0	3	1	1.72	1	1.12	1			9.0963	7.6417	4.7370	12.1124	10.3808	8.7024	9.2687	6.0662	1
GSM107196	0	0	0	3	0	7.71	0	6.44	1			9.4361	8.1371	4.5436	11.4522	10.1325	8.8564	9.3034	6.1223	1
GSM107197	1	1	0	3	0	5.69	0	5.69	0			11.7530	10.6322	4.6655	11.9592	9.8770	9.1759	9.0859	4.7270	0
GSM107198	0	0	0	2	0	7.67	0	7.67	0			8.6132	6.7615	4.5088	12.0445	10.1191	8.8796	9.3314	4.8397	0
GSM107199	0	1	1	3	1	4.79	1	3.92	1			9.7260	12.3642	5.3331	12.1671	10.9107	9.2376	8.8196	5.7682	1
GSM107200	0	1	1	3	0	7.78	0	7.78	0			9.5531	11.3089	8.9168	11.9937	9.7798	9.3837	9.1135	5.2962	0
GSM107201	0	1	0	3	0	7.50	0	7.5	0			9.8504	11.3401	4.2678	12.3729	10.2972	10.2738	9.6082	5.1101	0
GSM107202	0	1	1	1	0	7.36	0	7.36	0			8.9891	11.7621	6.0804	11.9522	10.0124	9.9923	9.2676	5.3265	0
GSM107203	0	1	1	3	0	7.73	0	7.73	0			9.9565	11.9757	5.9305	11.9202	9.3140	9.9607	8.6532	4.9622	0
GSM107204	0	0	0	3	1	1.14	1	1.12	1			8.7431	9.2757	4.3625	12.7593	11.0557	8.9194	9.7190	5.2722	1
GSM107205	1	0	0	2	1	1.67	1	1.3	1			13.1139	9.3042	4.7324	12.0938	10.1040	9.3455	8.9663	4.6245	0
GSM107206	0	0	0		1	3.84	1	1.46	1			10.3970	7.3673	4.5266	12.3696	10.0495	9.4087	9.8865	5.1498	0
GSM107207	0	0	0	2	0	8.47	0	8.47	0			9.8186	9.0589	3.8701	11.9040	9.8059	8.3088	8.8433	4.6056	0
GSM107208	1	0	0	3	0	8.03	0	8.03	0			12.0296	8.5141	3.8036	12.2199	9.6105	8.6798	8.5963	4.7021	0
GSM107209	0	0	0	1	0	7.99	0	7.99	0			9.6349	8.6008	3.9525	11.8442	9.8180	8.2897	8.9305	5.0116	0
GSM107210	0	1	0	3	1	2.80	1	0.76	1			9.4585	10.3254	4.5947	12.1602	10.1897	8.6764	9.4649	4.9879	0
GSM107211	1	0	0	3	0	7.63	0	7.63	0			12.5909	9.2216	3.6871	12.2412	10.0926	8.4432	8.7099	5.1425	0
GSM107212	0	0	0	2	0	7.84	0	7.84	0			9.3666	8.6525	4.6083	12.3070	10.5986	8.3730	8.9517	5.2797	0
GSM107213	0	0	0	2	0	7.75	0	7.75	0			8.9851	9.3289	3.7103	11.7623	8.9998	7.7680	8.5416	4.9213	0
GSM107214	0	0	0	1	0	7.67	0	7.67	0			10.2247	9.3176	3.3870	11.8466	10.1310	8.6598	9.4941	5.0267	0
GSM107215	0	0	0	3	0	2.28	1	1.26	1			9.0784	5.7167	3.5401	12.4942	10.5063	8.3488	9.4226	4.8979	0
GSM107216	0	0	0	3	0	6.61	0	6.61	0			11.0291	8.8209	4.4058	11.7355	9.5462	6.8141	8.5210	4.9902	0
GSM107217	0	0	0	3	0	7.19	0	7.19	0			8.3658	5.6113	3.8495	11.9414	10.6707	7.8431	8.8521	5.3432	0
GSM107218	0	0	0	3	0	6.44	0	6.44	0			8.1109	7.4698	4.4537	11.8187	9.9816	8.0975	8.2013	4.7255	0
GSM107219	0	0	0	3	1	4.41	1	0.9	1			9.3185	5.7884	3.9868	12.0289	9.9253	8.4231	8.7094	5.0033	0
GSM107220	0	1	0	3	0	6.32	0	6.32	0			9.7992	10.0031	3.9979	12.1033	10.4846	8.9393	9.0398	5.2101	0
GSM107221	0	0	0	2	0	8.36	0	8.36	0			9.7704	8.1177	3.8590	11.8154	9.6643	8.2457	8.5912	4.9745	0
GSM107222	0	1	1	2	0	8.49	0	8.49	0			9.5412	11.1965	4.8490	11.9536	9.9481	7.5198	8.8250	4.8616	0
GSM107223	0	0	0	3	0	0.18	1	0.18	0			10.0812	8.7472	3.3157	12.4636	10.4451	7.9485	9.0430	4.4787	0
GSM107224	0	1	1	3	0	5.94	0	5.94	0			9.7017	12.5869	7.2137	11.7448	10.2696	8.1457	8.9056	4.9181	0
GSM107225	0	0	0	3	0	8.32	0	8.32	0			8.7213	8.8289	3.5850	11.8900	9.9195	8.0599	8.9986	5.0553	0
GSM107226	0	1	0	3	0	5.98	0	1.34	1			9.8739	11.4969	4.1433	12.7080	10.3646	8.8168	9.5348	4.9880	0
GSM107227	0	1	1	3	0	6.02	0	0.67	1			9.4044	11.6001	5.4735	11.9430	10.0012	8.9770	9.4088	5.1879	0
GSM107228	1	1	0	3	1	3.07	1	2.79	1			12.8477	10.3172	3.8428	12.2882	9.6750	8.6714	8.5809	4.8336	0
GSM107229	0	0	0	3	1	4.87	1	1.38	1			9.1402	7.5606	4.0315	12.0136	10.9574	8.7738	10.3666	6.0101	1
GSM107230	0	1	1	1	0	5.62	0	5.62	0			10.0045	11.1207	5.1544	11.9792	10.2418	8.4236	8.6205	5.2093	0
GSM107231	0	0	0	2	0	0.70	1	0.7	0			10.8970	8.9823	4.5428	12.1483	10.5140	7.7544	9.1001	5.0040	0
GSM120649	0	0	1							11.34	0	8.3222	7.4061	4.8672	12.3454	10.8361	9.6751	8.5067	5.0163	0
GSM120651	0	0	0							7.36	0	9.2430	8.6390	4.7280	12.4809	10.3356	9.6434	8.7920	5.0067	0
GSM120652	1	0	0							1.02	1	13.3253	8.1413	4.7569	12.7816	10.3792	7.6592	8.7957	4.6625	0
GSM120653	0	0	0							8.00	0	10.8866	8.7975	4.6779	12.4792	10.4313	10.2180	8.9541	5.6954	1
GSM120654	0	0	1							8.11	0	10.8139	9.1160	5.5555	12.1485	10.3978	8.9062	8.7195	5.4749	0
GSM120655	0	0	1							0.84	1	8.5106	8.1934	4.8400	12.3766	10.2357	9.0929	9.5019	4.9326	0
GSM120656	0	0	0							1.87	1	8.9968	6.7379	4.4327	11.3049	9.5423	8.9956	8.8531	5.3348	0
GSM120657	0	0	1							5.09	0	8.7330	7.7095	5.1120	12.7161	8.7693	9.4309	9.6970	5.1620	0
GSM120658	0	0	1							10.22	0	9.2031	8.2951	5.3013	12.5574	10.5993	8.5489	10.1085	5.2985	0
GSM120659	0	1	1							8.76	0	9.6825	12.2472	5.3306	12.3843	10.3902	9.1487	8.9689	5.3315	0
GSM120660	0	0	0							9.70	0	8.7560	7.0894	4.7536	13.0303	10.3032	9.0126	8.6678	5.1415	1
GSM120661	0	0	1							7.21	0	9.7944	7.4650	5.3712	13.5621	10.2438	8.3362	8.7299	5.5830	1
GSM120662	0	1	1							5.73	1	10.1144	11.3218	4.9026	12.7475	10.3371	7.8173	8.9031	5.1246	0
GSM120663	0	1	1							2.41	1	9.2393	9.7256	5.3822	12.3368	10.2152	8.4080	8.8650	5.2779	0
GSM120664	1	0	1							6.93	0	13.8339	7.9092	4.8743	12.8367	10.3137	8.8799	8.8399	4.9469	1
GSM120665	1	1	1							13.04	0	13.1445	10.3798	5.3353	12.8474	10.2076	9.0845	8.3751	5.3153	1
GSM120666	0	0	1							7.29	0	10.7110	8.4160	5.3129	12.2063	11.1096	8.8238	8.8189	5.2583	1
GSM120667	0	1	0							5.94	0	8.7023	12.0147	4.5043	12.2907	10.8869	8.4682	8.7353	4.9441	0
GSM120668	0	0	1							0.60	1	8.6687	8.7084	4.8821	12.2875	10.9033	8.1430	8.9642	5.1407	0
GSM120669	0	1	0							8.02	0	9.1678	9.6631	4.4279	12.7220	10.2184	8.6111	9.0556	4.7421	0
GSM120670	0	0	0							5.88	0	9.9429	8.8895	3.9620	12.9167	9.9814	9.6680	8.7428	4.8556	1
GSM120671	0	0	0							1.97	1	8.3757	7.6784	4.5729	12.9439	10.2588	9.1894	8.7998	4.9645	1
GSM120672	0	0	0							10.64	0	8.1989	8.6226	4.6088	12.3760	10.6943	8.8950	9.2468	5.0904	0
GSM120673	1	0	0							5.36	0	13.8452	8.8159	4.7941	12.5848	10.3252	8.9931	8.4142	5.5245	0
GSM120674	0	0	1							7.19	0	10.7723	7.7100	4.8888	13.0007	10.2209	9.5212	8.7645	5.3272	1
GSM120675	0	0	0							9.95	0	8.9851	8.1851	4.7027	12.0052	10.5522	8.5234	8.1671	4.9445	0
GSM120676	1	0	1							6.14	0	12.6297	8.6701	5.0513	12.6542	10.8408	9.2770	8.8430	5.1400	0
GSM120677	0	1	1							5.70	0	9.8596	9.7284	4.9858	12.5760	9.8144	9.5290	9.1054	5.3472	0
GSM120678	1	1	1							8.87	0	13.2296	9.5517	5.1117	12.7284	11.2749	9.5635	8.9724	4.9675	1
GSM120679	0	0	0							3.47	1	8.6979	7.6609	4.5800	12.6615	9.6954	8.9400	8.7033	4.9015	0
GSM120680	0	0	0							8.61	0	9.0685	6.4576	4.7925	12.7379	11.1197	9.0173	8.8794	5.0346	1
GSM120681	1	0	0							9.59	0	12.3504	7.3128	4.4873	12.1633	11.3430	9.3972	9.0088	5.2562	1
GSM120682	0	0	0							6.36	0	8.7896	5.6788	4.3071	12.6031	8.8922	9.6911	9.3783	5.7017	1
GSM120683	0	0	0							10.57	0	9.0206	6.7674	4.2597	13.2974	10.5073	9.2682	8.5054	5.3359	1
GSM120684	0	0	0							10.93	0	9.7556	7.5127	4.5379	12.2150	10.8000	8.4348	9.6406	4.8519	0
GSM120685	1	0	1							7.33	0	11.4017	8.6834	4.8911	12.3605	10.7677	8.7049	8.6868	5.3102	0
GSM120686	0	0	0							6.74	0	9.3669	3.5665	3.3380	14.2688	10.1779	10.8240	10.0371	6.2680	1
GSM120687	0	0	1							0.77	1	9.6238	8.3558	5.0650	12.7133	10.4602	8.2045	8.3792	4.8610	0
GSM120688	0	0	1							10.84	0	9.8145	8.0030	4.9192	12.3640	11.0273	8.3007	8.7852	4.9905	1
GSM120689	0	0	0							5.00	0	9.6081	9.2351	4.1295	12.7295	10.5928	9.0906	8.4990	5.1365	0
GSM120690	0	0	1							7.32	0	8.6281	8.1172	4.9785	12.4960	10.6061	8.3210	8.9455	5.1762	0
GSM120691	0	0	1							8.57	0	8.5472	7.9537	4.8619	12.7268	10.0781	8.1075	8.7142	4.9189	0
GSM120692	0	0	0							7.39	0	9.3874	7.1740	4.3987	12.4246	10.7254	8.6973	9.1004	4.8712	0
GSM120693	1	0	0							5.22	0	12.8462	8.5649	4.4264	12.4498	10.4079	8.9476	8.5401	5.4280	0
GSM120694	1	0	1							7.18	0	13.2985	8.3665	5.3888	12.4743	9.7409	8.5382	9.3159	5.0813	0
GSM120695	0	0	0							7.49	0	9.8966	7.4609	4.7712	12.4454	10.2584	8.3710	8.5497	4.9498	0

GSM120696			0	0	1				12.63	0	9.2114	8.5095	4.8392	12.8249	11.6018	8.9381	8.1014	5.4942	1
GSM120697			0	1	1				9.29	0	9.3249	10.6086	7.3376	12.3000	10.3370	8.6057	8.5766	5.3055	0
GSM120698			0	0	1				5.12	0	8.3738	8.5161	5.1278	12.3649	10.4836	9.2910	8.9909	5.0711	0
GSM120699			0	0	1				6.64	0	9.4534	8.0095	5.0729	12.4819	10.8737	9.3836	9.6604	5.4404	0
GSM120700			1	1	1				6.06	0	13.4938	12.2017	5.5943	12.1051	10.1024	8.2220	8.5207	5.5101	0
GSM120701			0	0	1				5.75	0	9.2195	9.1154	5.0738	11.8902	10.9504	9.5157	9.0333	5.4954	1
GSM120702			0	0	1				0.36	1	8.3451	7.7321	4.9339	12.9139	10.7263	8.3965	9.0166	5.0342	1
GSM120703			0	0	0				7.60	0	9.8432	9.2512	4.6853	11.8225	10.6848	9.8455	7.8339	5.0913	0
GSM120704			0	1	1				7.60	0	10.2580	10.8478	5.1333	12.6026	10.4434	9.3111	8.6343	5.2255	0
GSM120705			0	1	1				6.91	0	9.4427	9.5201	5.1329	11.7888	10.6801	8.2131	8.9437	5.3376	0
GSM120706			0	0	1				8.51	0	9.0975	9.1327	5.0975	12.1987	10.3998	8.5842	7.7278	5.4081	0
GSM120707			0	1	1				1.80	1	10.2902	11.8657	5.2393	11.7956	10.0691	8.5961	8.7772	5.2477	0
GSM124994	1	0	1	0	0	1	3	2.3			12.9497	7.7866	3.7275	12.6687	10.7717	10.5336	8.7042	6.6781	1
GSM124995	0	0	0	0	0	0	3	0.9			8.9405	7.4437	4.0219	12.2946	10.0382	10.1623	8.7729	4.5837	0
GSM124996	1	0	1	0	0	0	3	2.5			13.2593	8.3493	4.4374	12.6558	10.5332	9.0788	8.7537	5.4334	0
GSM124997	0	0	0	0	0	0	3	3.5			8.2895	6.8782	4.4999	12.4649	10.7473	9.5117	9.0436	6.1789	1
GSM124998	0	0	0	0	0	1	3	2.1			7.4765	7.1532	4.2983	12.6538	10.8820	10.1114	9.8262	5.3431	0
GSM124999	1	0	1	0	0	1	3	2.5			13.3813	6.8442	3.8878	12.5578	10.3125	10.3205	9.1057	4.9056	0
GSM125000	0	0	0	0	0	1	3	1.2			8.3206	6.8306	3.8430	12.3071	11.3181	8.9414	8.9830	5.6662	1
GSM125001	1	0	1	0	0	0	3	2.1			13.0075	9.2497	4.7963	12.3933	10.6651	10.3838	9.0812	4.2857	0
GSM125002	0	0	0	0	0	1	2	2.5			9.7202	8.6439	4.2553	12.7553	10.2242	9.8766	8.9285	4.4838	0
GSM125003	0	0	0	0	0	1	3	3.3			8.5128	8.3306	4.2517	12.3032	9.8250	9.7141	9.7971	5.0800	0
GSM125004	0	0	0	0	0	0	3	2.5			8.4384	7.2346	4.4147	12.5749	10.4441	10.4854	10.1770	5.5697	1
GSM125005	0	0	0	0	0	1	3	3			7.9611	8.1985	3.9535	12.8079	11.2225	9.3818	9.6708	6.7757	1
GSM125006	0	0	0	0	0	0	2	1.8			8.3250	7.5079	4.1669	12.4809	9.7208	9.8685	8.2140	5.0514	0
GSM125007	0	0	0	0	0	1	3	4			8.3998	8.8148	3.6921	12.4146	10.8431	9.0222	9.4094	6.3340	1
GSM125008	0	0	0	1	1	0	3	2.1			9.6295	12.8107	5.3482	12.3294	10.1825	9.8157	9.4455	4.4936	0
GSM125009	0	0	0	0	0	0	3	1			9.0044	8.2675	4.3761	12.1047	9.6196	9.2922	10.1498	5.4997	0
GSM125010	0	0	0	0	0	0	3	0.9			8.2763	6.9444	4.3967	12.6073	9.1878	8.5100	8.1894	4.9592	0
GSM125011	0	0	0	0	0	0	3	2.2			8.4722	8.1458	4.1284	12.6855	9.5824	9.4688	8.8489	4.8112	0
GSM125012	0	0	0	0	0	0	3	1			8.3605	6.6069	3.8160	12.0259	9.2288	9.0491	8.4920	4.4501	0
GSM125013	1	0	1	0	0	1	3	3.5			12.6475	8.2307	4.5236	12.4652	9.8695	8.4554	8.7089	6.1785	1
GSM125014	0	0	0	0	0	0	3	2.5			8.3474	7.4574	4.6401	11.9771	9.2107	9.5582	7.4637	4.5898	0
GSM125015	0	0	0	1	0	0	2	2.6			9.5512	13.3794	4.2121	12.2496	10.2678	9.5275	8.7511	6.3597	1
GSM125016	0	0	0	0	0	0	3	3			8.4336	7.3867	4.3930	12.5967	10.5388	8.9805	9.0583	5.9531	1
GSM125017	0	0	0	0	0	0	3	1.3			9.7875	6.5904	3.8121	12.3630	9.7724	9.5945	8.8479	4.8246	0
GSM125018	0	0	0	0	0	0	3	1.4			7.8100	5.5666	3.7742	13.0054	9.7276	9.1454	8.8123	5.5675	1
GSM125019	0	0	0	0	0	0	3	1.7			8.5530	6.9537	3.9499	11.0737	10.1442	8.1881	8.4942	4.9353	0
GSM125020	0	0	0	0	0	0	3	2.3			7.4060	5.8198	4.4529	12.2318	11.1158	11.1998	8.7358	4.6533	1
GSM125021	1	0	1	0	0	1	3	3.4			13.4012	7.3334	4.0681	12.6604	9.9267	8.9745	8.7670	6.5124	1
GSM125022	0	0	0	0	0	0	3	1.5			7.9196	9.1008	3.3787	12.4843	10.2847	9.1553	8.8518	5.4777	0
GSM125023	0	0	0	0	0	0	3	2.5			8.5565	7.8343	4.1681	12.0395	10.1579	9.8346	8.7579	6.3157	1
GSM125024	0	0	0	0	0	0	3	2.8			9.2995	7.5069	4.1777	12.0813	10.1106	8.2529	9.9282	5.9172	1
GSM125025	0	0	0	0	0	0	3	2.2			8.3383	7.0194	3.8072	12.6032	11.3547	8.6055	8.6484	5.4491	1
GSM125026	0	1	0	1	1	0	1	3.5			8.7854	10.9033	6.5508	12.1927	9.3043	9.5444	8.5933	4.3706	0
GSM125027	1	1	0	1	1	0	3	1			11.1077	10.8130	4.9927	12.5781	10.4283	10.5607	8.5881	5.0183	0
GSM125028	0	1	0	1	0	0	1	1.7			10.2145	11.4773	3.7651	12.8469	10.6261	9.1746	9.4490	4.3267	1
GSM125029	1	1	1	0	0	1	3	1.4			12.4540	9.2884	3.9016	12.5496	9.2595	9.9121	8.5008	4.8477	0
GSM125030	0	1	0	1	0	1	2	2.6			10.3723	10.8390	4.7372	12.4001	10.0516	9.4716	9.2722	4.5802	0
GSM125031	0	1	0	1	1	0	2	0.9			9.7299	9.8648	6.5570	13.0161	10.6918	10.1308	8.6932	5.6464	1
GSM125032	0	1	0	1	0	0	1	0.8			10.1395	9.5924	4.3201	12.4738	9.8881	9.3097	8.4442	5.1980	0
GSM125033	1	1	1	1	0	0	2	2.2			11.8193	11.8672	4.1544	12.4290	10.1375	8.9797	8.7379	5.1357	0
GSM125034	1	0	0	0	0	0	3	1.8			8.2608	6.7950	4.0239	12.2523	10.6150	8.9873	8.5755	6.9921	1
GSM125035	0	1	0	1	1	1	2	2.2			9.1501	11.7271	6.5196	12.5470	10.2500	9.9493	9.3519	4.6582	0
GSM125036	1	1	0	1	0	0	3	4.2			9.3413	12.6870	4.4291	12.7463	9.8246	8.7353	9.0216	5.4010	0
GSM125037	1	0	0	0	0	0	2	2.5			10.6998	6.5004	3.9291	12.5642	10.1866	9.0151	8.7311	4.7326	0
GSM125038	0	1	0	1	1	0	2	2.3			9.4690	11.2236	4.9756	12.3005	9.6341	9.1999	8.7405	5.3674	0
GSM125039	1	1	0	1	0	1	3	2			9.5942	11.3859	4.4322	12.5982	9.8248	9.0477	8.8082	4.7817	0
GSM125040	1	1	0	1	0	1	2	1.7			10.7010	11.6558	3.7406	12.2891	10.2378	9.3820	9.0757	4.9722	0
GSM125041	0	1	0	1	0	1	2	1.1			9.4148	11.5513	4.1817	12.2652	9.9669	10.0369	9.2479	4.6482	0
GSM125042	0	1	0	1	1	1	2	2.5			9.7138	11.6035	6.4621	12.6467	10.2150	9.1913	9.4738	4.9327	0
GSM125043	0	1	0	1	1	0	1	0.9			10.5231	11.2725	7.5829	12.2442	10.0116	9.1844	8.7725	4.8298	0
GSM125044	0	0	0	0	0	0	3	3			10.1474	6.4745	3.9858	12.4567	11.1283	9.2338	9.4118	7.4449	1
GSM125045	0	1	0	1	0	0	1	2.3			10.2192	10.2471	3.8996	12.4088	10.2032	9.4656	9.0644	5.9674	1
GSM125046	0	1	0	1	0	1	2	1.5			9.7684	12.5068	4.2446	11.6841	10.1096	8.6028	8.7053	4.7371	0
GSM125047	0	1	0	1	1	1	1	4			10.4626	11.8446	4.8212	12.5700	10.5269	9.3685	8.8976	5.1097	0
GSM125048	1	0	1	0	0	0	3	1.5			12.6735	7.0801	4.0021	12.5573	10.3668	8.9531	8.8722	5.7240	1
GSM125049	0	1	0	1	1	0	1	1.1			9.7128	12.1525	5.5531	12.8872	10.0886	9.7933	9.5711	5.7368	1
GSM125050	0	1	0	1	0	1	2	5.5			10.0103	11.6521	4.3849	12.8254	11.0840	9.5112	9.0830	5.3582	1
GSM125051	0	1	0	1	0	1	3	2.3			9.5422	12.5618	4.4760	12.8479	10.0202	9.0715	9.1541	5.0320	1
GSM125052	0	1	0	1	0	0	2	1			10.1473	11.3777	4.5692	12.0816	9.9389	8.5234	9.3482	5.3389	0
GSM125053	0	0	0	0	0	0	3	1.1			8.6303	6.9444	4.0226	12.2888	10.2341	8.4375	8.5310	4.8646	0
GSM125054	0	0	0	0	0	0	3	1.1			8.5474	5.7380	3.5616	12.4980	9.9545	9.7909	8.9316	6.0353	1
GSM125055	0	1	0	1	0	1	1	2.5			9.6351	11.2855	3.2834	12.4208	10.0689	8.3320	8.7304	5.6071	1
GSM125056	0	0	0	0	0	1	3	3			10.0728	8.6524	4.6513	12.5708	9.8954	8.0991	9.3969	4.8894	0
GSM125057	0	0	0	0	0	1	3	5.2			8.4195	7.5251	3.9465	12.7012	9.0585	9.5396	8.1226	4.7744	0
GSM125058	0	0	0	0	0	0	3	2.5			7.7007	6.5311	3.7217	12.3966	10.0548	9.0182	9.3573	5.2955	0
GSM125059	0	1	0	1	0	0	2	2.5			9.1691	10.0426	3.2960	11.8931	9.0245	9.5732	8.2788	4.3208	0
GSM125060	0	1	0	1	1	0	1	1.1			9.5665	12.1439	5.0113	12.3139	9.9508	9.5349	8.8115	4.3715	0
GSM125061	0	1	0	1	0	1	3	1.7			10.1055	12.4502	3.9156	12.3575	10.4119	9.5347	8.8392	5.2676	0
GSM125062	0	1	0	1	1	0	2	2.1			9.7197	12.8715	6.2715	12.5539	10.3917	9.1172	9.5701	5.9547	1
GSM125063	0	1	0	1	0	1	3	1.5			9.6795	12.6679	4.0417	11.5709	9.6883	8.0836	9.3129	5.0955	0
GSM125064	0	0	0	0	0	0	3	1.9			9.9612	7.1912	4.3255	12.3527	10.0626	8.7365	9.9279	6.1407	1
GSM125065	0	1	0	1	1	1	2	3.5			9.1268	12.7985	6.9921	12.4753	10.6308	9.7732	8.8760	4.5314	0
GSM125066	1	0	1	0	0	1	3	2.2			13.5084	6.4246	3.5952	12.5253	10.0226	9.3612	8.9239	6.5585	1
GSM125067	0	1	0	1	0	0	1	1.5			9.7434	12.0944	3.7732	12.0936	9.9428	8.8319	8.4349	5.1323	0
GSM125068	0	0	0	0	0	0	3	2			8.4422	7.2795	3.1671	12.1169	8.9730	9.2435	8.6838	4.9015	0
GSM125069	1	1	1	1	1	1	2	1.3			13.2429	10.4043	5.1977	12.2746	10.0812	8.8049	8.2585	4.7785	0
GSM125070	1	0	0	0	0	1	3	2.5			9.0599	5.9309	3.6428	12.4384	10.2728	8.9763	8.6049	5.0310	0
GSM125071	0	0	0	0	0	0	3	1.7			8.0537	6.0526	3.6337	11.9291	9.6526	9.2782	8.2368	5.1620	0
GSM125072	1	1	1	1	1	1	2	2.9			12.4544	10.6935	5.5065	12.6606	10.5789	8.8531	9.1620	5.4702	0
GSM125073	1	0	1	0	0	0	3	3			13.2850	7.2972	4.1241	12.3367	9.8284	8.9427	8.6415	5.2408	0
GSM125074	0	1	0	1	1	1	1	1.1			9.0339	11.0472	5.4101	12.4225	10.5029	7.3170	9.1544	4.3638	0
GSM125075	0	1	0	1	1	0	1	2			10.0931	10.9977	5.5140	11.9704	9.9816	9.0883	9.2405	5.6815	1
GSM125076	1	1	0	1	1	0	3	1.2			10.8790	12.8941	5.4170	12.1673	10.0726	9.2057	8.8270	5.2783	0
GSM125077	0	1	1	1	0	1	2	3.5			11.1827	12.1043	3.9462	12.5240	9.9956	7.5121	8.7158	4.6301	0
GSM125078	1	1	1	1	0	1	2	3.1			11.9618	11.2353	4.3390	12.3571	9.9687	8.7098	9.2190	5.1539	0
GSM125079	0	1	0	1	1	1	1	1.2			9.6784	12.0434	5.1704	12.3099	10.2809	9.1523	9.2115	5.9157	1
GSM125080	0	1	0	1	0	1	3	4.2			10.6857	13.3346	4.5582	11.8406	10.4568	8.5302	8.8481	6.0306	1
GSM125081	0	1	0	1	1	1	3	3			9.0160	13.0760	8.1568	12.2004	9.9375	9.3378	8.8727	5.2922	0
GSM125082	0	1	0	1	1	1	3	2.2			9.7592	12.1350	6.8767	12.4775	10.2403	9.3536	8.7962	4.8824	0
GSM125083	0	1	0	1	0	1	1	1.3			10.0821	11.4053	3.4805	11.8841	9.7109	8.3590	8.6605	5.0165	0
GSM125084	0	1	0	1	1	1	1	1.2			9.4481	11.4987	5.1314	13.1437	10.0785	8.6992	8.5337	4.8276	1
GSM125085	1	1	1	1	0	0	3	2			11.7106	10.2667	3.5657	12.1856	9.8604	8.9103	8.8765	4.7183	0
GSM125086	0	1	0	1	0	0	1	1.1			9.9031	11.7670	3.3644	12.3642	10.2069	9.2298	8.4427	4.8158	0

GSM125087	1	0	0	0	0	1	2	2.9						10.2164	8.2459	4.2202	12.3694	9.8571	9.2574	8.4612	5.3863	0
GSM125088	0	1	0	1	1	1	3	2.5						8.6917	12.6739	5.1215	11.9327	8.9334	7.7824	8.0708	5.1267	0
GSM125089	0	1	0	1	0	1	3	2.5						9.4686	12.5254	3.6925	13.1018	11.4365	9.5944	8.9507	5.5922	1
GSM125090	1	1	1	0	0	1	3	4.5						13.3499	6.6037	3.8210	12.7651	10.4099	8.9073	8.8975	4.6654	0
GSM125091	0	1	0	1	0	0	2	1.2						9.4055	10.3455	4.2177	12.1836	9.7668	7.5932	8.9517	4.8248	0
GSM125092	0	1	0	1	1	1	1	1.5						10.1126	11.5284	6.1316	12.3703	10.2034	8.8586	9.3986	5.0907	0
GSM125093	0	1	0	1	0	0	1	5						9.4486	10.1920	3.3951	12.2988	10.2373	7.9012	9.1296	4.9925	0
GSM125094	0	1	0	1	0	1	1	1.5						8.8732	11.2413	4.1453	12.1944	9.6405	8.7669	8.0517	5.0999	0
GSM125095	0	1	0	1	0	0	1	1.4						9.9944	10.7287	3.7566	11.9099	9.7699	8.7793	8.2762	5.5121	0
GSM125096	0	1	0	1	0	0	1	1.1						10.2982	11.5040	3.8944	12.0511	9.6045	8.8553	8.7567	5.0293	0
GSM125097	0	1	0	1	0	0	1	0.8						10.3499	11.4046	3.7910	12.1600	9.9408	8.9406	8.8191	5.2985	0
GSM125098	0	1	0	1	1	0	1	1						9.4156	10.4514	5.7996	12.3395	9.1968	8.5446	8.7342	5.1133	0
GSM125099	0	1	0	1	1	0	2	2.1						9.1811	11.4582	7.3372	12.0507	10.2835	8.6716	8.7424	5.6717	1
GSM125100	0	1	0	1	0	0	2	1.5						10.4871	12.3871	3.9156	12.8738	10.9010	8.9543	9.2174	5.2255	1
GSM125101	0	1	0	1	0	0	1	1.3						9.4163	11.3562	4.4102	12.0204	9.9100	8.7600	8.4663	5.0543	0
GSM125102	0	1	0	1	1	0	2	1.2						10.1937	10.4578	6.9442	12.2165	10.0642	9.4298	9.7937	5.6157	1
GSM125103	0	1	0	1	0	1	3	1.9						8.5251	16.1011	3.3728	14.3713	10.9904	12.0313	8.1019	5.6056	1
GSM125104	1	1	0	1	0	0	3	2.1						9.2956	13.3236	4.3160	12.4536	10.3814	9.5415	8.5305	4.5027	0
GSM125105	0	0	0	0	0	1	3	5.5						9.7486	7.1131	3.8242	12.2532	10.3946	9.0595	8.4937	5.6929	1
GSM125106	0	1	0	1	0	1	1	3.5						8.7045	11.0656	3.9498	12.5328	10.3843	9.2469	8.6766	4.9677	0
GSM125107	0	1	0	1	0	1	2	6.5						9.9147	11.4666	3.8360	12.3848	11.6170	9.3584	8.5666	6.1369	1
GSM125108	0	1	0	1	1	1	3	4.5						8.1823	11.1198	6.0528	12.2933	10.6410	9.8015	8.0707	6.3599	1
GSM125109	1	1	1	1	1	1	3	2.3						11.8104	11.4980	6.5939	12.2793	9.9134	8.8519	8.1413	4.8507	0
GSM125110	1	0	1	0	0	1	3	7						12.7734	7.0747	3.6080	12.7252	10.3490	9.8916	8.8883	4.7613	0
GSM125111	0	1	0	1	1	1	1	2.9						9.5986	12.0023	4.8395	12.2285	10.4238	9.1605	8.7928	5.0215	0
GSM125112	0	1	0	1	1	1	2	4						9.2463	12.1252	5.4524	12.6012	10.4325	8.4871	9.1206	5.2339	0
GSM125113	0	1	0	1	1	1	2	5.5						9.6768	12.4934	5.2340	12.3609	10.6095	9.1644	9.0619	5.4633	0
GSM125114	0	1	0	1	0	1	3	2.3						10.1715	14.3889	4.7389	13.1811	10.9263	8.3822	8.8587	5.9227	1
GSM125115	1	1	1	0	0	1	3	4						13.1852	8.5140	3.6992	12.9006	10.4810	9.5650	8.7917	5.0178	1
GSM125116	1	0	1	0	0	1	3	8.5						12.7373	5.7190	4.0102	12.9354	9.9606	9.6174	8.8448	5.1443	1
GSM125117	0	1	0	1	0	1	1	7						9.8905	10.7074	4.0451	12.7746	10.3383	9.4227	8.7577	5.3112	0
GSM125118	0	1	0	1	1	1	2	3.5						10.1877	12.0712	6.8835	12.7372	9.9327	9.5079	8.8336	5.3271	0
GSM125121	0	0	0	0	0	1	3	1.7						10.4195	7.1106	4.2564	12.5331	9.8510	9.1611	8.6609	5.3970	0
GSM125122	0	0	0	0	0	1	3	4.2						10.0041	9.5083	4.5264	12.7362	10.5209	9.4599	9.1955	6.0948	1
GSM150794			0	0	1	0	2	1.2	46					10.3046	8.5612	4.8798	12.9174	9.8498	9.6710	8.8609	5.2196	1
GSM150795		0	1	0	0	0		1	51					11.3117	6.9616	3.2923	12.4634	10.2530	9.4052	9.3674	5.4788	0
GSM150796		0	1	0	0	0	2	0	48					12.3668	6.3012	3.8676	12.4927	9.8940	8.7912	9.0708	4.9459	0
GSM150797		0	0	0	0	0	3	3.5	29					9.2823	7.1831	3.9569	12.3127	9.8565	8.8488	8.6529	4.9430	0
GSM150798		0	0	0	0	0		1	44					10.0280	7.7431	4.3163	12.7571	10.6614	9.9962	9.5187	5.5059	0
GSM150799		1	0	1	0	0		1.3	64					9.7602	9.6887	4.2508	12.4621	10.3672	9.9745	9.6126	5.3714	0
GSM150800		0	0	1	0	0		0	57					10.1604	10.1217	4.6732	12.9100	10.7648	9.2526	8.7335	4.9768	1
GSM150801		0	1	0	0	0	2	0	24					11.6093	7.8083	3.7093	12.3095	9.7098	8.9287	9.0024	4.9571	0
GSM150802			0	1	1									9.5043	10.6198	5.6990	12.7327	10.3153	9.3215	9.6636	5.2623	0
GSM150803			0	0	0									9.9482	7.7414	4.3000	12.5191	9.9881	9.4574	9.1497	4.6894	0
GSM150804			1	0	0									12.8808	6.0849	3.7207	12.7210	9.8532	9.5054	8.6887	5.0981	0
GSM150805			1	0	0	0								13.6160	5.3940	3.8334	12.4043	9.7695	8.7195	8.1387	4.8265	0
GSM150943		1	1	1	1	0		0	43	2.68	1	2.68	0	11.9064	10.5157	5.3685	12.8463	11.0539	9.1164	8.9162	5.2393	1
GSM150944		1	0	1	0	0		3.5	61	7.74	1	7.74	0	9.7378	9.9245	3.6229	12.7339	10.4563	9.4312	9.2524	5.2860	0
GSM150945		1	0	1	0	0		5	70	6.21	1	6.21	1	10.0819	12.5732	4.7459	12.4704	10.9293	8.6613	8.9873	5.5808	1
GSM150946		1	0	1	0	0	2	2	69	3.72	1	3.72	1	10.1154	12.0773	3.9959	13.0992	11.6381	9.7945	9.0695	5.6929	1
GSM150947		1	0	1	0	1	2	3	69	2.29	1	2.29	1	10.2020	9.7639	3.8203	13.6309	11.7328	9.2509	9.2164	6.0442	1
GSM150948		1	0	1	1	1	2	4	63	7.28	0	7.28	0	9.5838	11.5148	5.0916	12.3970	10.7224	9.3732	9.7147	5.4588	0
GSM150949		1	0	1	1		2	1	61	7.79	0	7.79	0	10.2606	11.4868	4.9822	12.7118	10.4400	9.2546	9.4752	5.3763	0
GSM150950		1	0	1	0	1	2	2.3	60	5.11	0	5.11	0	10.1402	11.8124	4.2372	12.1996	10.4390	8.6863	9.5872	6.1203	1
GSM150951		1	0	1	1	0	2	3	71	5.16	0	5.16	0	9.1479	12.3819	5.5401	12.2566	10.1107	9.1065	9.1145	5.2684	0
GSM150952		1	0	1	0	0		2.5	67	4.49	0	4.49	0	10.1294	12.3982	3.8920	12.5399	10.0519	9.3077	9.2609	4.8419	0
GSM150953		1	0	1	0	0		4.2	58	8.00	0	8.00	0	10.7531	10.5282	4.1022	11.4235	11.0131	7.9345	9.5334	5.3644	1
GSM150954		1	0	1	1	0		1.5	53	6.25	0	6.25	0	9.7371	10.1301	5.2728	12.4246	10.5724	9.4058	9.0574	5.6022	1
GSM150955		1	0	1	1	0		4	79	4.12	1	4.12	0	9.9188	10.7892	4.9636	12.5280	10.6174	8.5688	9.3543	5.4438	0
GSM150956		1	0	1	1	1	2	4	71	3.10	1	3.10	1	9.5568	11.8916	6.2839	12.4296	10.6818	9.2166	9.4090	5.2597	0
GSM150957		1	0	1	0	0		7	66	6.93	0	6.93	0	9.8716	10.7755	3.9885	12.5158	10.3186	8.6506	8.9234	5.0616	0
GSM150958		1	0	1	1	0	2	1.7	57	7.19	0	7.19	0	9.7831	10.6772	6.7361	12.5127	10.7212	8.9617	9.2768	5.1255	0
GSM150959		1	0	1	1	1	2	4	68	7.04	0	7.04	0	10.7734	12.4365	5.2374	12.4528	10.9234	9.6125	9.7656	4.9715	0
GSM150960		1	0	1	0	0		1.4	58	1.98	1	1.98	1	11.0267	11.7917	3.9983	12.8388	10.9687	8.5874	9.2815	5.4246	1
GSM150961		1	0	1	1	1	2	3	67	1.96	1	1.96	1	9.8410	11.4862	5.4001	12.4562	10.0806	9.0936	8.9613	5.6182	1
GSM150962		1	0	1	0	0	2	0	52	4.03	0	4.03	0	9.6695	12.6250	4.4393	12.2202	9.6847	9.4066	8.8375	5.2053	0
GSM150963		1	0	1	0	1	2	1.3	69	7.92	1	7.92	0	10.8195	11.0073	4.0747	12.3928	10.3670	8.4916	9.1922	5.0131	0
GSM150964		1	0	1	1	0	2	3	44	3.99	0	3.99	0	9.8255	11.8061	7.5907	12.8173	10.3167	9.8147	9.7636	4.9294	1
GSM150965		1	0	1	0	0	2	3	71	6.86	0	6.86	0	9.7938	11.8051	4.2567	12.9403	10.5991	9.6252	9.3672	5.5685	1
GSM150966		1	0	1	1	0	2	1.4	52	5.97	0	5.97	0	10.1801	9.6375	7.1162	12.5540	10.1001	7.5041	9.6209	5.2411	0
GSM150967		1	0	1	0	1		3.2	80	0.04	0			9.0399	10.6287	3.6735	13.1110	11.1892	9.0587	8.5479	5.3703	1
GSM150968		1	0	1	0	1		3	61	2.86	0	2.86	0	10.1214	11.0104	4.1161	12.6254	10.4096	8.9827	8.9867	5.1503	0
GSM150969		1	0	1	1	1	2	3	74	3.63	1	3.63	1	9.7413	11.4948	6.0451	12.8296	10.7664	10.0140	9.4461	5.1344	1
GSM150970		1	0	1	0	0	2	1.9	68	4.82	0	4.82	0	9.8330	11.9721	4.6402	12.3720	10.3311	9.1827	8.9181	5.4422	0
GSM150971		1	0	1	0	0	2	4.5	69	4.94	0	4.94	0	10.1893	11.5023	4.1122	12.7632	10.8443	8.0624	9.3819	5.5655	0
GSM150972		1	0	1	0	1	2	2.1	61	4.99	0	4.99	0	9.3282	11.7674	3.6906	12.6785	9.9037	8.4329	8.8413	5.3266	0
GSM150973		1	0	1	1	0	2	2	76	5.36	0	5.36	0	9.4710	11.9072	5.6690	12.2788	10.4316	9.4636	9.2847	5.3820	0
GSM150974		1	0	1	0	0	2	2.3	67	3.78	1	3.78	1	11.0904	10.8221	3.6825	12.2487	10.3715	9.7134	9.0172	5.6960	1
GSM150975		1	0	1	1	0	2	2.2	65	3.31	0	3.31	0	9.3886	11.6667	5.0384	12.7001	10.1658	9.2540	9.1300	5.3587	0
GSM150976		1	0	1	1	1	2	2.8	75	4.95	0	4.95	0	10.0266	12.4288	4.8406	12.3806	9.9610	8.5558	8.7870	5.4074	0
GSM150977		1	0	1	1	0	2	3	70	5.86	0	5.86	0	9.7313	12.1349	5.6781	12.5957	10.5290	9.2716	9.2379	5.2301	0
GSM150978		1	0	0	0	0	2	2.5	47	5.18	1	5.18	0	10.5482	9.3652	4.3546	12.6023	10.9846	9.1456	8.7354	5.7898	1
GSM150979		1	0	1	0	0	2	2.5	61	5.65	0	5.65	0	10.0125	10.7312	4.0984	12.1561	10.1121	9.1297	8.6488	5.0798	0
GSM150980		1	0	1	1	0	2	2.4	69	4.57	0	4.57	0	10.7254	11.0793	4.9323	12.4562	10.3315	9.4271	9.0893	5.2545	0
GSM150981		1	0	1	1	0	2	2	77	4.53	0	4.53	0	9.8769	12.5157	7.2509	12.5298	10.6717	9.5006	9.8854	5.5820	1
GSM150982		1	0	1	0	1	2	2.8	69	4.55	0			10.9845	12.2343	3.9202	13.4958	11.6341	10.5760	8.7369	5.8275	1
GSM150983		1	0	1	0	1	2	2.2	61	10.49	0	10.49	0	10.1472	10.7250	3.8863	12.9246	11.4008	8.4272	9.6754	5.5630	1
GSM150984		1	1	1	0	1	2	2	64	9.35	0	9.35	0	12.7251	11.3643	4.0408	12.2905	9.8657	9.0145	9.2015	5.5696	1
GSM150985		1	0	1	0		2	1.7	75	10.58	0	10.58	0	10.0004	11.8307	4.5590	12.4544	10.0748	8.0346	8.9057	5.1562	0
GSM150986		1	0	1	1	0	2	1.7	57	10.16	0	10.16	0	9.4140	11.6766	4.8786	12.2173	9.0407	8.8352	9.2736	5.2125	0
GSM150987		1	0	1	0	0	2	2.2	51	3.77	1	3.77	1	9.7891	10.0450	3.9332	12.2841	10.4524	9.0191	8.9449	5.1580	0
GSM150988		1	0	1	1	1		6	69	0.02	0			10.4631	12.7109	7.5530	12.5975	10.0880	9.0292	9.7476	4.9747	0
GSM150989		1	0	1	1	1	2	7	71	5.19	0	5.19	0	9.6697	14.0023	5.1024	12.1992	10.9783	8.5126	9.4672	5.0314	1
GSM150990		1	0	1	1	0		2.3	66	9.80	0	9.80	0	9.9211	11.8604	6.9756	12.6043	10.8272	9.5877	9.0257	5.7502	1
GSM150991		1	0	1	0	1	2	2	63	12.75	0	12.75	0	9.8474	11.7773	4.7989	12.3463	9.8819	8.4350	9.2305	5.0162	0
GSM150992		1	1	0	0	0	2	3	62	9.78	0	9.78	0	12.5738	7.8470	3.7836	12.5684	10.5846	9.3714	8.8467	5.3773	0
GSM150993		1	0	1	1	0		0.9	56	9.58	0	9.58	0	10.3114	12.5700	7.6749	12.3740	10.9283	10.5405	9.3736	5.4803	0
GSM150994		1	0	1	1	0	2	1.3	48	11.05	0	11.05	0	10.0858	12.3811	7.4741	12.3387	10.3105	8.3592	9.9986	5.2188	0
GSM150995		1	0	1	0	0	2	1	53	10.38	0	10.38	0	9.2595	11.3808	4.4761	12.9614	11.2418	9.5095	9.4937	5.1092	1
GSM150996		1	0	1	1	1	2	1.5	61	10.01	0	10.01	0	9.1113	12.7476	6.0796	12.7754	11.2078	9.6842	9.5054	4.8924	1
GSM150997		1	0	1	1	0	2	1.8	58	9.88	0	9.88	0	8.7729	10.5814	5.5261	12.1182	10.1311	8.6793	8.7411	4.6498	0
GSM150998		1	0	1	0	1		6	67	2.67	1	2.67	1	9.5676	10.3817	3.7566	12.7990	10.2994	8.9091	9.4952	5.6324	1
GSM150999		1	0	1	1	0		3.9	60	3.54	1	3.54	1	9.9220	11.5644	5.5339	12.0700	10.4850	9.2744	8.7903	5.1932	0
GSM151000		1	0	0	1	0		3	44	4.35	1	4.35	0	9.6083	9.0560	5.6762	12.3942	10.2522	10.6685	9.5647	5.3585	0
GSM151001		1	1	1	0	0	2	3	44	1.35	1	1.35	1	12.1305	10.5283	4.0882	12.4894	10.7115	9.3386	8.6983	4.9331	0

GSM151002	1		0	1	0	1	2	2.8	73	6.04	1	6.04	0	10.3991	10.5121	3.9114	12.4736	10.9572	9.6665	9.3371	5.5440	1
GSM151003	1		0	1	1	0	2	1.5	49	5.46	0	5.46	0	9.5423	10.4536	6.4375	12.3852	10.2201	9.8797	9.4500	4.9596	0
GSM151004	1		0	1	1		2	2	71	4.11	0	4.11	0	9.7290	11.4542	6.2597	12.6095	10.5946	9.7740	9.7575	5.1053	0
GSM151005	1		0	1	1	0		1.5	65	9.43	0	9.43	0	9.3176	10.1413	5.4233	12.1942	9.9043	10.1808	8.7776	4.5616	0
GSM151006	1		0	1	0	0	2	1.5	46	2.59	0	2.59	0	9.9667	9.6489	4.0850	12.1398	10.0342	9.6906	8.9417	4.8931	0
GSM151007	1		0	1	1	0	2	3.5	69	4.15	0	4.15	0	10.2550	11.2778	7.3223	12.0289	9.8042	9.8886	8.8441	5.5529	0
GSM151008	1		1	1	0	1	2	3	61	8.24	0	8.24	0	13.4199	9.7472	4.1617	12.9391	10.9088	8.3397	8.5424	5.3808	1
GSM151009	1		0	1	1	0	2	1.5	48	8.09	0	8.09	0	10.4414	11.0244	6.8372	12.3308	9.7567	8.2634	8.8002	4.9828	0
GSM151010	1		0	0	0	0	2	1.5	60	8.00	0	8.00	0	10.9046	9.2673	4.6624	12.5023	10.1799	8.8335	8.6427	5.0290	0
GSM151011	1		0	1	0		2	1.8	74	8.11	0	8.11	0	10.3948	11.1335	3.7755	12.1475	9.7293	8.6316	8.8786	5.2721	0
GSM151012			0	0	0									8.6838	6.2239	4.3231	12.2482	10.7614	10.2090	8.1404	4.6989	0
GSM151013	1	1	0	1	0	1	2	2.4	61	0.92	1	0.92	1	9.3496	11.5698	4.4712	11.9643	10.1782	9.6957	8.7611	4.8140	0
GSM151014	1	1	0	1	1	1	2	1.8	73	4.42	1	4.42	1	10.4602	10.5905	5.6311	12.2642	10.0235	10.2790	8.8248	4.9048	0
GSM151015			1	0	0									12.9837	5.8982	4.0569	12.6332	9.6937	10.3364	8.8951	4.6810	0
GSM151016	1	1	0	1	0	1		2.6	71	2.83	1	2.83	0	9.9960	10.2321	4.5097	12.0794	10.0534	9.3163	8.7662	4.7647	0
GSM151017	1	1	0	1	0	1	2	2	61	10.08	0	10.08	0	9.4984	10.5218	3.9179	12.1685	10.2527	9.3173	8.9294	4.8730	0
GSM151018	1	1	0	1	1	1	2	3.5	55	9.58	0	9.58	0	9.9577	11.5087	4.8602	12.6572	9.1399	9.0267	8.5992	4.7810	0
GSM151019	1	1	0	1	0	1	2	0.9	52	3.42	1	3.42	1	10.6428	12.3917	4.6245	12.9396	10.0248	9.0359	9.0998	5.0069	1
GSM151020	1	1	0	1	0	1	2	1.4	57	6.50	1	6.50	1	9.9387	11.1485	4.7642	12.1267	9.7576	8.6001	9.0462	4.8732	0
GSM151021	1	1	0	0	0	0	2	5	83	4.42	0	4.42	0	9.4521	9.0354	4.7627	11.9007	9.7435	8.9987	8.9988	4.7054	0
GSM151022	1	1	0	1	0	1	2	2.3	74	1.50	1	1.50	1	8.3607	11.2340	4.7245	12.3317	10.1388	9.4251	8.9602	4.9205	0
GSM151023	1	1	0	1	1	1	2	2.2	65	9.83	0	9.83	0	10.2948	11.9275	4.9666	11.9735	9.3261	9.6415	8.7057	4.6006	0
GSM151024	1	1	0	1	0	0	2	1.41	57	9.83	0	9.83	0	9.0568	10.2484	4.3217	12.4377	9.7693	8.9539	8.7973	4.4876	0
GSM151025	1	1	0	1	0	1	2	2.5	70	4.08	1	4.08	0	9.1966	10.1733	4.2756	12.2529	10.7418	8.9189	8.5371	4.9960	0
GSM151026	1	1	0	1	1	0	2	1.8	62	1.75	1	1.75	0	9.8397	11.7384	5.0090	12.2466	10.5019	9.0750	9.1861	4.7341	0
GSM151027	1	1	1	0	0		2	2.5	83	1.92	0	1.92	0	13.3977	9.2223	4.2991	12.4158	10.4656	9.5018	8.7485	4.7770	0
GSM151028	1	1	0	1	0	1	2	2.4	60	7.00	1	7.00	1	11.1353	11.7642	4.2493	12.6971	10.7647	9.7258	9.0403	4.6624	0
GSM151029	1	1	0	1	1	1		4.6	86	0.17	1			9.4874	12.1762	5.2708	12.2592	9.7087	9.5517	8.5868	4.7029	0
GSM151030			0	0	0									9.7398	6.1314	4.3580	12.5580	10.3607	9.2779	9.3272	4.8334	0
GSM151031	1	1	1	1	0	0	2	2.7	59	4.42	1	4.42	0	12.2780	11.5363	3.8025	12.8554	10.1819	8.6158	8.9935	5.1090	1
GSM151032			1	0	0									11.4915	6.1950	3.8220	12.1342	9.5179	8.2104	8.6641	4.6801	0
GSM151033	1	1	0	1	0	0	2	1	72	9.83	0	9.83	0	9.6569	9.8127	3.9285	12.3510	10.1228	8.7608	8.9709	4.5564	0
GSM151034	1	1	0	1	0	1	2	4	51	9.92	0	9.92	0	10.0542	9.8011	3.7694	12.1171	10.1021	8.3434	8.7342	4.6514	0
GSM151035	1		0	1	1	0	2	2.4	74	3.75	1	3.75	0	9.8502	10.9028	6.1976	12.1198	10.1728	9.9458	9.1909	5.0248	0
GSM151036	1	1	0	1	0	1	2	2.2	71	9.00	0	9.00	0	8.8833	9.6576	4.6555	12.3520	9.8775	9.5456	8.9373	4.7529	0
GSM151037	1	1	0	1	0	1	2	2.7	72	10.25	0	10.25	0	9.4147	10.8449	4.5239	12.3396	10.6313	9.2919	9.4933	5.3295	0
GSM151038	1	1	0	0	0	1	2	2.2	77	0.50	1	0.50	1	10.4112	7.5459	3.5555	12.3655	10.0495	9.1963	9.1265	4.5876	0
GSM151039	1		0	1	0	0	1	2.1	71	9.67	0	9.67	0	9.8679	12.0342	4.4809	12.5687	10.4436	9.2043	8.7280	4.6958	0
GSM151040	0	1	1	0	0	0	3	2.4	54	9.25	0	9.30	0	12.8955	6.2633	3.9210	11.8054	9.4691	9.0841	8.7111	4.5915	0
GSM151041	1	1	0	1	0	1	2	1.7	65	9.50	0	9.50	0	10.3740	10.0959	3.6642	12.5578	11.5296	8.2136	8.8099	4.7242	1
GSM151042	1	1	0	0	0	1	2	3.3	51	7.25	1	7.25	1	9.4532	9.5164	3.8437	12.7938	10.3470	9.8556	8.9105	5.1541	0
GSM151043			0	1	0									9.4315	9.7701	4.2114	12.2179	9.6974	9.3189	9.1438	4.8568	0
GSM151044	1	1	0	1	1	0	2	2.2	67	9.42	0	9.42	0	9.9228	10.5757	5.1091	12.5547	9.5240	9.5724	9.2482	4.4929	0
GSM151045	0	1	1	0	0	1	2	3.2	57	1.25	1	1.30	1	13.4855	6.6414	3.7418	12.1328	9.6264	9.5288	8.4727	4.7816	0
GSM151046	0	1	1	0	0	1	3	2.9	66	1.17	1	1.20	1	13.2251	5.9454	3.6570	12.3468	10.2618	10.0674	8.7104	4.5831	0
GSM151047	1	1	0	1	1	1	2	1.3	67	5.92	0	5.92	0	9.9540	10.4117	5.9401	12.9037	10.2341	11.3428	8.8673	4.9902	1
GSM151048	1	1	0	1	0	1	2	3.8	88	6.33	0	6.33	0	9.7620	11.3065	4.1982	11.9802	9.4325	9.3642	8.8094	4.5962	0
GSM151049	0	1	0	0	0	0	3	4.2	84	0.92	0	0.90	0	9.8599	6.0719	4.2965	12.6991	9.7033	9.9991	8.5505	4.7564	0
GSM151050	1	1	0	1	1	0	2	2.4	62	6.50	1	6.50	1	10.1973	11.1773	6.9142	12.4136	9.0913	9.1083	8.7175	4.3483	0
GSM151051	1	1	1	0	0	0	2	3.2	74	3.25	0	3.25	0	13.3357	6.1272	3.6898	12.3599	9.9144	9.3928	8.8565	4.4691	0
GSM151052	1	1	0	1	0	0	2	2.4	66	7.83	0	7.83	0	10.2172	11.0042	3.6203	12.5700	10.6282	10.2960	9.1362	5.0390	0
GSM151053	1	1	0	0	1	0	2	2.3	45	2.42	1	2.42	0	9.7268	9.0643	5.2704	12.3180	10.3589	9.4113	8.5222	4.5924	0
GSM151054	1	1	0	1	0	1	2	2.2	56	8.75	0	8.75	0	10.2720	10.8620	3.8832	12.9699	10.3606	9.1032	8.8834	5.1232	1
GSM151055	1	1	0	1	0	1	2	2.2	61	8.58	0	8.58	0	9.2331	9.5354	3.6310	12.0793	9.5046	9.1901	8.8299	4.2292	0
GSM151056	1	1	0	1	0	1	2	3.2	59	10.08	0	10.08	0	9.6259	12.2724	4.7680	12.3504	9.4951	8.1737	9.0361	4.8027	0
GSM151057	1	1	0	1	0	1	2	2.1	82	2.58	1	2.58	0	9.9229	11.5463	4.0708	13.1123	11.3979	9.5586	9.1538	5.1468	1
GSM151058	1	1	0	1	0	1	2	4.5	52	1.08	1	1.08	1	9.5925	9.9210	3.8518	12.3582	10.0104	8.0071	8.8356	4.2434	0
GSM151059	1	1	0	1	0	1	2	2.4	55	0.17	1			9.4370	10.4367	4.0910	12.1620	10.0020	7.4490	8.3218	4.7878	0
GSM151060	1	0	0	1	0	0	2	2.8	61	10.00	0	10.00	0	10.4561	11.0162	3.9034	12.1467	9.2856	8.6037	8.8908	4.8482	0
GSM151061	0	1	1	0	0	1	3	2.8	79	0.92	1	0.90	1	11.8181	4.5222	4.0127	12.5726	10.1165	8.8412	8.5280	5.3283	0
GSM151062			0	1	1									9.7850	10.4847	5.0681	12.6717	10.4498	9.1022	8.9324	4.7454	0
GSM151063	1	0	1	1	1	1	2	2	75	1.08	1	1.08	1	11.2336	11.2029	5.2042	12.3212	10.2918	9.2843	8.8218	5.1170	0
GSM151064			1	1	0									12.3966	11.0337	4.2939	12.8597	9.9362	9.0514	8.7416	4.5446	1
GSM151065	1	1	0	0	0	1	2	1.9	52	2.42	1	2.42	1	9.3419	8.5011	4.4409	12.2072	9.9210	9.6516	8.7732	4.4785	0
GSM151066	1	1	0	1	0	0	2	2.8	84	6.25	0	6.25	0	9.8637	9.5495	3.7953	12.1697	10.3919	9.2737	9.4430	5.0322	0
GSM151067	1	1	0	1	0	1	2	1.5	86	2.08	0	2.08	0	10.9822	9.9363	3.9078	12.8737	10.2772	9.3238	8.9889	5.1318	1
GSM151068	1	1	1	1	1	1	2	3.8	63	0.08	1	0.25	1	11.3233	11.4311	5.0327	12.7133	10.0595	10.5472	8.7092	5.0164	0
GSM151259	1	1	0	1	0	0		3	46	10.47	1	10.47	1	8.6235	9.9713	3.6557	12.5357	10.3393	9.0994	9.1072	5.0011	0
GSM151260	1	1	0	1	1	1		2	71	8.28	1	8.28	1	9.3223	11.1481	5.6249	13.0659	10.4681	9.7007	9.4640	5.2475	1
GSM151261	1		0	1	1	1		1.3	58	16.54	0	16.54	0	8.8771	12.4237	5.9877	12.9415	10.7565	9.6105	9.3499	4.7670	1
GSM151262	1	1	0	1	0	1	2	2	57	11.97	1	11.97	1	9.5879	11.6797	4.4230	12.7741	10.0480	9.1094	9.1455	5.1460	0
GSM151263	1	1	0	1	0	1		1.5	69	14.56	0	14.56	0	9.7446	10.8117	4.7837	12.4219	10.0551	8.3767	8.6864	4.8596	0
GSM151264	1	0	0	1	0	0	1	2	67	8.04	1	8.04	1	8.5170	12.1610	4.3489	12.4266	10.2276	9.6347	9.8083	4.7940	0
GSM151265	1	1	0	1	1	1		2.5	56	14.22	0	14.22	0	9.5505	12.5886	8.1883	12.7548	10.8309	9.8405	9.0237	4.4590	0
GSM151266	1	1	0	1	1	1	2	2	53	4.42	1	4.42	1	9.0916	12.4296	7.6474	12.5884	10.1105	8.5788	9.9388	4.8472	0
GSM151267	1	1	1	1	0	1	2	4	56	3.79	0	3.79	0	11.2922	13.2934	3.8991	12.6658	10.0366	9.8791	9.1134	4.5836	0
GSM151268	1		0	1	0	1		5.1	68	2.08	1	2.08	1	8.6206	11.4378	3.3146	12.8424	10.7493	9.6533	9.0908	4.7260	1
GSM151269	1	1	1	1	0	0	2	2	59	3.11	1	3.11	1	12.9703	11.4001	4.4902	12.2218	10.7174	8.2480	9.1643	5.2459	0
GSM151270	1	1	0	1	1	1	2	3.5	57	16.32	0	16.32	0	9.0187	10.4985	6.3040	12.4178	10.2092	8.7712	9.3134	4.6736	0
GSM151271	1	0	0	1	0	1	2	5	61	2.47	1	2.47	1	9.2333	12.7229	4.3364	12.9798	11.4124	8.4742	9.4783	5.0430	1
GSM151272	1	1	0	1	1	1	2	2.5	61	16.85	0	16.85	0	9.8499	12.6392	8.1753	12.5965	10.5200	9.2587	9.3356	4.7941	0
GSM151273	1	1	0	1	1	1	2	2.5	52	16.35	0	16.35	0	8.9697	12.5934	5.3817	13.0144	10.7939	8.8859	9.3549	4.9487	1
GSM151274	1	1	0	1	0	1	2	1.5	63	15.72	0	15.72	0	9.4812	12.0187	3.7192	12.5887	10.1418	9.8343	8.3434	5.4504	0
GSM151275	1	1	0	1	0	1	2	3.9	62	4.85	1	4.85	1	11.1426	12.3563	4.4341	12.5343	10.0305	8.9940	9.7861	5.1250	0
GSM151276	1	1	0	1	1	1	1	2.5	58	15.95	0	15.95	0	10.0345	11.6086	5.7462	12.5612	10.3746	8.5074	9.4193	4.6991	0
GSM151277	1	0	0	1	0	1	2	3	55	3.54	1	3.54	1	8.2174	12.8006	3.8299	12.7589	10.0294	9.9495	8.5488	4.9075	0
GSM151278	1	1	0	1	1	1	2	1.5	60	16.33	0	16.33	0	9.6671	11.4969	5.3691	12.5011	9.8846	9.0490	8.7815	4.5341	0
GSM151279	1	1	0	1	0	1		3.5	58	10.10	1	10.10	1	10.3054	11.8537	3.3878	12.3602	9.6566	8.6479	9.2073	4.6856	0
GSM151280	1	1	0	1	0	1	2	1.5	60	15.68	0	15.68	0	10.1346	11.8410	3.6659	13.1105	11.2308	9.4607	9.9185	4.5090	1
GSM151281	1	1	0	1	1	1	2	2.5	66	7.18	1	7.18	1	9.6490	11.9291	7.8284	13.0918	11.1109	9.4480	9.3554	5.0372	1
GSM151282	1	1	1	1	0	1	3	3.5	51	15.22	0	15.22	0	12.8034	11.0041	4.4036	12.7452	9.3022	8.8690	8.4814	4.7391	0
GSM151283	1	0	1	1	0	0		3	62	15.14	0	15.14	0	11.2278	12.4773	3.6276	12.9959	10.4461	9.5234	10.0471	4.5685	1
GSM151284	1	0	0	1	0	1	2	2	53	15.32	0	15.32	0	10.9082	10.7403	3.5342	12.8369	10.6171	9.3153	9.5422	5.2779	1
GSM151285	1	1	0	1	0	1		2	62	9.19	1	9.19	1	10.5519	11.2836	3.3485	12.5888	10.3824	8.3022	9.0238	4.8260	0
GSM151286	1	1	0	1	1	0	3	2.7	61	2.93	1	2.93	1	10.2247	11.5810	6.2055	12.5757	10.0219	8.1739	9.0920	5.0296	0
GSM151287	1	0	0	1	0	1	3	2	59	15.12	0	15.12	0	9.3422	12.8437	4.5813	11.8809	9.8492	9.7530	8.7647	4.8839	0
GSM151288	1	1	0	1	0	1	2	2.5	61	7.08	0	7.08	0	8.6054	11.8616	4.8149	12.6368	9.7979	10.2382	8.8426	4.9768	0
GSM151289	1	1	0	1	1	1	2	3	62	14.22	0	14.22	0	9.5992	12.6158	5.9976	13.2703	10.5869	9.9276	10.0123	4.9439	1
GSM151290	1	1	0	1	1	1	2	2.5	43	12.22	0	12.22	0	9.4057	10.7215	6.9100	12.6159	9.8415	9.1451	8.8495	5.0964	0
GSM151291	1	0	0	1	0	0	2	2	56	13.08	1	13.08	1	9.7951	11.7460	3.5697	12.7128	10.4810	8.9015	9.3301	4.8035	0
GSM151292	1	1	0	1	1	0		4	53	14.06	0	14.06	0	10.3394	11.0062	5.4815	12.5994	10.4167	8.6814	9.2206	5.0337	0
GSM151293	1	1	0	1	1	1		1.1	59	14.45	0	14.45	0	9.7136	12.4687	6.5282	12.6663	10.3027	8.8749	9.2363	5.2104	0
GSM151294	1	1	0	1	1	1	1	3	58	14.55	0	14.55	0	9.6612	11.9933	5.3543	12.4964	9.7258	8.7794	8.7585	4.6196	0
GSM151295	1	0	1	0	0	1		5	53	0.52	1	0.52	1	12.0701	8.7549	3.8597	12.8823	10.1229	8.8525	9.0833	4.2872	1
GSM151296	1	1	0	1	0	1	1	2.5	66	13.03	0	13.03	0	9.2513	10.9891	4.7305	13.0779	10.9297	9.1105	9.8913	4.8643	1

GSM151297	1	0	0	1	0	1	3	1.5	67			3.40	0	3.40	0	8.8832	12.3281	3.7713	12.6815	10.1951	9.4512	9.4006	4.6871	0
GSM151298	1	0	1	1	0	1	3	3	63			5.27	1	5.27	1	12.8670	11.9965	3.8133	12.8854	10.1616	9.7678	8.5770	4.5834	1
GSM151299	1	1	0	1	0	1		1.5	61			12.23	1	12.23	1	9.9925	11.7292	3.9330	12.6964	10.2390	9.2921	7.9355	5.0365	0
GSM151300	1	1	0	1	0	0	2	2	66			14.28	0	14.28	0	9.4744	12.5861	4.4394	12.3568	9.9516	8.2986	8.7119	4.6438	0
GSM151301	1	0	0	1	0	1	2	1.4	61			1.17	1	1.17	1	10.0592	12.4863	3.5961	12.8531	11.2191	8.5039	8.8143	4.6469	1
GSM151302	1	1	0	1	1	0		3	57			13.14	0	13.14	0	8.1844	13.0444	5.1033	12.7348	8.9100	9.2591	9.4690	4.8073	0
GSM151303	1	1	0	1	1	0	2	3.8	59			3.39	1	3.39	1	9.7592	11.6133	6.2502	12.6531	10.9423	8.2382	9.2708	5.1673	0
GSM151304	1	1	0	1	1	1	2	3	56			13.29	0	13.29	0	9.6059	11.6166	6.5955	12.7347	10.2018	9.0099	9.1729	4.8257	0
GSM151305	1	1	0	1	1	1	1	2	61			14.22	0	14.22	0	9.9778	11.7656	8.1803	12.5575	9.9672	8.7832	9.0360	5.4261	0
GSM151306	1	1	0	1	1	0	1	1.9	67			13.85	0	13.85	0	9.8443	11.4202	7.1993	12.3469	9.7818	8.3463	9.4829	4.6461	0
GSM151307	1	1	0	1	1	1		3	68			1.04	0	1.04	0	9.8879	11.1778	6.5014	12.8903	10.3714	8.5246	8.5349	4.5181	1
GSM151308	1	1	0	1	0	1	2	4	59			2.54	1	2.54	1	10.2157	10.4990	3.8481	12.4512	11.4696	7.3304	9.0371	4.9407	1
GSM151309	1	1	1	0	0	1	3	3	50			13.62	0	13.62	0	12.6695	7.6775	4.1254	12.7169	9.8027	9.1484	8.6179	4.6156	0
GSM151310	1	1	0	1	1	1	3	3	63			10.40	1	10.40	1	9.3213	10.2544	6.0891	12.7273	10.7360	9.8392	8.1292	5.4073	0
GSM151311	1	1	0	1	1	1	1	1.8	50			12.68	0	12.68	0	10.2874	10.3564	7.1250	12.2300	10.3195	8.0213	8.5185	4.7938	0
GSM151312	1	1	0	1	1	0	1	1.2	53			13.19	0	13.19	0	9.9230	11.8368	6.9276	12.3930	10.2676	8.4157	9.4722	5.0347	0
GSM151313	1	1	0	1	0	1	2	2.5	70			13.67	0	13.67	0	9.8016	12.2185	3.6452	12.5879	9.6626	9.2473	8.8368	5.0605	0
GSM151314	1	1	0	1	1	1	1	1.6	77			12.38	0	12.38	0	10.1736	10.7964	5.5146	12.6794	10.1442	7.4576	9.1532	5.4092	0
GSM151315	1	1	0	1	1	1	2	1.9	68			9.84	0	9.84	0	9.6157	12.8846	9.0909	12.6253	9.9751	9.0867	9.0999	5.0118	0
GSM151316	1	0	0	1	1	0	1	1.5	71			12.15	0	12.15	0	10.4026	11.8144	7.3333	12.6428	10.7495	8.1305	9.6098	5.5838	1
GSM151317	1	1	0	1	1	0	3	1.2	72			7.82	0	7.82	0	10.1600	11.1855	6.8613	12.3508	10.2771	9.3569	8.6376	4.8831	0
GSM151318	1	1	0	1	1	0	2	1.85	86			4.65	0	4.65	0	10.4206	11.5636	8.2473	12.5321	10.8153	9.1687	8.1948	4.6678	0
GSM151319	1	1	0	1	0	1	1	1.8	82			9.81	0	9.81	0	10.0605	10.5654	3.1223	12.6315	10.2094	7.7732	8.3536	5.2472	0
GSM151320	1	0	1	1	0	0	3	2.5	82			11.38	0	11.38	0	13.0151	11.5690	3.3098	12.5810	10.5960	7.1146	7.5656	4.6965	0
GSM151321	1	1	0	1	0	0	2	1.7	78			12.22	0	12.22	0	9.9407	11.9574	3.8304	12.4270	10.2093	7.7490	9.2355	4.7289	0
GSM151322	1	1	0	1	0	1		3	65			9.05	1	9.05	1	10.1464	11.7310	3.7196	12.8811	10.1243	9.8901	8.2662	5.0253	1
GSM151323	1	1	0	1	1	0	2	1.5	63			8.23	1	8.23	1	10.2840	12.0409	8.3679	12.6045	9.8838	7.7570	8.7765	5.3554	0
GSM151324	1	1	0	1	1	1	2	2	65			12.13	0	12.13	0	9.6522	11.5974	6.0845	12.8246	10.0833	9.6187	8.9770	4.8471	1
GSM151325	1	1	0	1	1	0	1	1.5	51			11.35	0	11.35	0	10.3734	11.4097	5.8373	12.6972	9.9613	9.2761	8.7816	4.6740	0
GSM151326	1	1	0	1	1	0	3	1.7	65			11.85	0	11.85	0	10.6654	12.1773	5.2307	13.0068	10.5961	8.6372	9.2609	5.2830	1
GSM151327	1	1	0	1	1	1	3	2.9	73			9.18	0	9.18	0	9.2804	11.8659	7.5788	12.6326	10.0058	8.0277	9.2163	4.6244	0
GSM151328	1	0	0	1	0	1	3	2.6	67			3.48	1	3.48	1	9.9085	11.5086	3.3143	12.8225	10.8345	9.7566	8.1816	5.1140	1
GSM151329	1	1	0	1	1	1	1	3.5	58			12.28	0	12.28	0	8.9668	11.2341	7.4500	12.6518	10.0581	9.0657	9.9491	4.9274	0
GSM151330	1	1	0	1	1	1	3	1.5	74			9.39	0	9.39	0	9.7428	12.5450	5.8320	12.6673	9.7371	8.4256	8.6957	4.7409	0
GSM151331	1	1	0	1	0	0	2	1.3	64			12.37	0	12.37	0	9.7278	10.7488	4.7400	12.6489	10.5134	8.4650	9.4021	4.9543	0
GSM151332	1	0	0	1	1	1	1	1.3	57			12.59	0	12.59	0	10.0575	11.3053	5.3873	12.6805	10.2261	9.9811	8.6705	4.9041	0
GSM151333	1	1	0	1	1	1	1	1.3	60			12.48	0	12.48	0	9.3006	11.0450	5.6474	12.9207	9.7899	9.4669	9.4274	4.7977	1
GSM151334	1	1	0	1	1	0	2	2.5	72			6.20	0	6.20	0	10.8625	11.5418	8.0507	12.2147	9.6467	9.2332	9.2935	5.0490	0
GSM151335	1	1	0	0	1	0	1	2.7	79			12.20	0	12.20	0	10.2697	8.9892	6.6961	12.9715	11.1515	10.6110	9.5606	5.5407	1
GSM151336	1	0	0	1	0	0	2	3.4	58			2.10	1	2.10	1	8.8693	12.4791	3.5867	12.8875	9.6112	8.8389	8.1921	5.1223	1
GSM151337	1	1	0	1	0	0		1.4	62			4.31	0	4.31	0	10.5299	11.3224	3.5176	11.7187	8.9950	8.3735	8.5306	5.2728	0
GSM151338	1	0	0	1	0	0	2	2	81			6.24	0	6.24	0	6.6687	12.4474	4.0000	12.6892	10.2266	9.3599	8.2176	5.0978	0
GSM151339	1	1	0	1	1	1	1	1.5	77			7.53	0	7.53	0	9.5199	12.2887	7.2930	12.7789	10.3980	9.7570	8.7086	5.0258	0
GSM151340	1	0	0	1	0	0	2	4	67			1.21	1	1.21	1	9.3297	11.2004	3.7548	12.7019	9.6650	8.8801	9.0982	4.8837	0
GSM151341	1	0	0	1	1	1	3	7.5	67			8.80	0	8.80	0	9.8497	13.4040	5.7248	12.5552	9.2422	9.0066	8.1177	5.1137	0
GSM151342	1	1	0	1	1	1	2	1.6	70			10.28	1	10.28	1	10.3915	11.7823	6.4854	12.8133	10.6951	9.3049	9.0235	5.0453	0
GSM151343	1	0	1	1	0	0	3	2.1	48			10.88	0	10.88	0	11.7390	9.7856	3.2712	13.0953	10.3621	9.5956	8.3731	4.7490	1
GSM151344	1	1	0	1	1	0	3	1.5	76			7.18	0	7.18	0	9.8806	12.8432	5.3342	12.6420	10.8648	9.2399	8.8339	5.0730	0
GSM151345	1	0	0	1	0	1	2	2.2	67			11.57	0	11.57	0	9.9328	11.2988	3.2672	13.2501	10.8143	9.4412	9.2993	5.0024	1
GSM177885	0		0	0	0	0	3	3	57	2.57	1	1.98	1	1.98	1	8.9245	5.3639	3.9259	13.0368	9.9483	8.9952	8.7849	4.7008	1
GSM177886	1		0	0	0	0	3	3	57	18.06	0	0.50	1	18.06	0	8.7758	9.3318	3.9854	12.0513	9.4904	9.3068	9.0121	4.7059	0
GSM177887	0		0	0	0	0	3	2.5	48	2.53	1	1.44	1	1.44	1	8.9121	4.4750	3.6769	12.2146	10.3684	8.5741	8.3400	4.9061	0
GSM177888	1		0	1	1	0	3	1.8	42	17.14	1	6.01	1	17.14	1	9.8847	11.4359	5.1672	11.8493	10.7634	8.4844	9.2668	4.6206	0
GSM177889	1		0	1	0	0	2	3	46	11.32	1	10.47	1	10.47	1	9.0270	9.7736	3.7121	10.4769	10.2409	8.4417	9.0223	5.6592	1
GSM177890	1		0	0	0	0	2	2	58	17.83	0	17.83	0	17.83	0	10.0269	9.0826	3.5975	12.0758	10.1916	8.7859	9.3966	4.9428	0
GSM177891	0		0	0	0	0	3	2	44	16.29	0	1.94	1	16.29	0	9.2245	7.5985	4.6921	11.5365	10.9020	8.2405	8.9464	6.7367	1
GSM177892	1		0	1	0	0	1	2.5	58	15.93	1	15.93	1	15.93	1	9.5873	10.5564	3.5897	12.1688	10.4773	8.8133	8.6545	4.9661	0
GSM177893	1		0	1	1	0	3	3	47	16.46	0	16.46	0	16.46	0	10.6814	12.3364	7.3724	12.8859	10.5332	9.0576	9.4610	4.7948	1
GSM177894	1		0	1	0	0	2	2.5	38	4.07	1	1.16	1	3.38	1	9.4597	9.9102	4.1232	12.3289	10.6030	8.7204	8.8433	4.8530	0
GSM177895	1		1	1	1	0	3	2	59	5.24	1	3.11	1	3.11	1	13.1033	12.2395	5.3991	11.9116	10.5890	8.8562	9.1572	4.9147	0
GSM177896	1		0	0	1	0	1	0.8	50	16.39	0	16.39	0	16.39	0	10.1200	8.1350	7.0852	12.7928	10.0995	9.6842	8.6628	5.1162	0
GSM177897	0		0	0	0	0	3	4.5	46	17.12	0	17.12	0	17.12	0	9.2419	5.4425	4.2626	11.9983	9.8784	8.1278	8.4507	4.6597	0
GSM177898	0		0	0	0	0	2	2	57	11.98	1	7.78	1	11.14	1	9.4888	7.0201	3.7305	12.2637	10.2481	9.7502	8.4254	4.5157	0
GSM177899	0		0	0	0	0	3	2.5	33	2.68	1	1.25	1	1.89	1	9.3697	5.6649	3.7994	13.1373	9.4198	8.8577	9.0121	4.7886	1
GSM177900	1		0	1	0	0	3	2	42	10.49	1	2.72	1	2.72	1	10.4353	10.4142	3.4461	11.6264	10.4713	8.7831	8.9544	6.2361	1
GSM177901	0		0	0	0	0	3	2	38	1.83	1	0.77	1	1.19	1	9.1937	8.3978	4.7627	12.3058	10.1264	8.9448	8.5603	4.7117	0
GSM177902	0		1	0	0	0	2	2.5	55	5.83	1	1.50	1	4.78	1	12.7767	5.6571	4.1505	12.7896	10.2609	9.1076	8.4367	5.5055	0
GSM177903	0		0	0	0	0	2	2.5	57	9.84	1	4.99	1	7.84	1	10.4518	6.0857	4.3421	12.7659	10.8250	8.8002	8.5583	4.5502	0
GSM177904	1		0	1	1	0	2	2.5	46	15.60	0	15.60	0	15.60	0	9.7894	9.7771	7.4591	12.2170	10.0084	8.3509	9.1656	5.0971	0
GSM177905	1		0	0	0	0	2	2	50	15.95	0	15.95	0	15.95	0	9.3738	8.9117	4.5806	12.5968	9.5875	9.0399	7.9663	5.0079	0
GSM177906	0		0	0	0	0	3	2	46	15.85	0	15.85	0	15.85	0	9.6377	3.5031	3.9454	12.6790	9.5358	9.0973	8.4977	4.8470	0
GSM177907	1		0	0	1	0	2	2.5	46	15.51	0	15.34	1	15.51	0	9.7753	9.2673	5.8050	12.6094	10.8396	8.6769	9.5247	4.9121	0
GSM177908	1		1	1	1	0	2	1.5	51	7.97	1	7.97	1	7.97	1	11.5924	10.5827	6.6024	12.2906	10.0836	9.1768	8.6144	5.0340	0
GSM177909	0		0	0	0	0	3	2.5	58	4.59	1	0.33	1	3.21	1	8.6722	5.3541	3.9996	12.5214	10.1259	9.1424	8.9504	5.0054	0
GSM177910	1		0	1	1	0	1	2.6	59	14.00	0	14.00	0	14.00	0	10.5496	13.2276	5.6821	13.2178	10.8257	8.8949	9.6555	4.6157	1
GSM177911	1		0	1	0	0	2	0.6	49	15.16	0	15.16	0	15.16	0	10.2133	9.7065	3.6312	12.2022	10.1918	8.6355	8.5663	4.5633	0
GSM177912	1		0	0	0	0	1	2.5	43	15.26	0	3.92	1	15.26	0	10.2591	9.0092	4.1717	12.1392	10.7341	9.6520	8.9507	4.7792	0
GSM177913	0		0	0	0	0	3	2	47	15.12	0	15.12	0	15.12	0	9.7533	6.3438	4.1996	11.5214	9.8849	8.0739	8.3346	4.7232	0
GSM177914	1		0	1	0	0	3	1.7	37	14.74	0	14.74	0	14.74	0	9.8705	9.9747	4.4689	11.8320	10.0738	9.2862	8.8162	5.4298	0
GSM177915	1		1	1	1	0	3	4	48	12.81	0	12.81	0	12.81	0	11.6968	10.8794	5.2298	12.2508	10.5383	9.2729	8.7761	5.1019	0
GSM177916	1		0	1	1	0	2	1.9	47	12.75	0	4.56	1	12.75	0	9.1185	11.8019	6.1155	12.2586	9.2827	8.2350	8.6874	4.8829	0
GSM177917	1		0	1	1	0	2	2.5	46	11.58	0	11.58	0	11.58	0	10.7498	11.3744	6.3176	12.7764	10.0863	9.7486	9.0906	5.2637	0
GSM177918	0		1	0	0	0	3	2	48	4.19	1	0.87	1	2.49	1	11.7538	6.1410	4.0154	12.3792	9.6966	9.1441	8.2848	5.0583	0
GSM177919	1		0	1	1	0	2	1.9	58	12.25	0	12.25	0	12.25	0	10.1829	10.7159	7.5081	12.6775	10.6691	9.2899	9.5009	5.3426	0
GSM177920	1		0	0	0	0	3	2	34	3.49	1	0.96	1	3.30	1	9.4273	8.8345	3.9196	12.8495	10.4739	8.7190	9.2129	5.5262	1
GSM177921	1		0	0	1	0	2	4.5	30	19.33	0	15.43	1	19.33	0	10.9004	9.3842	4.9683	12.4194	10.6738	8.6035	8.9299	5.5809	1
GSM177922	1		0	1	0	0	2	3	35	2.62	0	2.62	0	2.62	0	10.5402	10.4072	3.9275	11.5814	10.3408	8.7217	8.7142	5.2899	0
GSM177923	0		0	0	0	0	3	2.6	56	14.68	0	14.68	0	14.68	0	8.4521	6.1039	4.3121	12.1539	9.4164	9.4464	8.6587	4.8581	0
GSM177924	0		0	1	0	0	2	3.2	35	16.29	0	16.29	0	16.29	0	9.5883	11.0704	4.4888	11.8565	9.7867	8.9818	8.7237	4.7321	0
GSM177925	0		0	0	0	0	3	2.7	52	15.48	0	15.48	0	15.48	0	7.7636	6.3460	4.2578	12.0937	10.3943	9.2567	9.0568	4.9796	0
GSM177926	1		0	1	0	0	3	3	47	16.54	1	1.45	1	1.45	1	10.2997	9.7824	4.6681	12.6379	10.4647	9.1837	9.3244	5.6332	1
GSM177927	0		1	0	0	0	3	1.6	42	3.93	1	1.12	1	2.18	1	13.4489	5.7373	3.5844	12.8038	10.2318	8.7022	8.7018	5.0092	0
GSM177928	1		0	1	0	0	2	1.7	54	15.58	0	15.58	0	15.58	0	10.1382	10.3559	3.5584	11.5758	9.3277	7.4627	8.9967	4.9716	0
GSM177929	1		0	0	0	0	3	2.5	42	4.81	1	2.80	1	2.80	1	9.6083	9.1836	4.1868	11.8093	9.3157	8.5793	8.3205	4.8437	0
GSM177930	1		0	0	0	0	2	2.5	50	13.13	0	13.13	0	13.13	0	10.2202	8.3672	3.4117	12.5350	10.1734	9.0068	9.0365	5.5459	0
GSM177931	1		0	1	1	0	1	3	42	3.33	0	3.33	0	3.33	0	9.7696	9.6529	5.1870	12.3739	10.4445	8.4953	9.5660	5.2429	0
GSM177932	1		0	1	0	0	1	3.5	38	10.35	0	10.35	0	10.35	0	9.5384	10.5317	3.9728	12.4351	10.8544	8.6935	8.7782	5.9883	1
GSM177933	1		0	0	0	0	3	2.5	37	14.54	0	14.54	0	14.54	0	9.8997	9.0060	3.6381	13.0267	10.4072	9.0996	8.8925	5.6866	1
GSM177934	1		1	1	0	0	3	3	44	15.56	0	15.56	0	15.56	0	12.8514	11.2717	4.6671	12.2755	10.3083	8.1593	9.2820	5.2862	0
GSM177935	0		0	0	0	0	3	3.1	37	2.18	1	1.15	1	1.15	1	9.4811	4.8602	3.7267	12.2795	10.0012	8.0884	9.3045	5.7033	1
GSM177936	1		0	1	0	0	2	1.5	51	15.38	0	2.30	1	15.38	0	10.2772	10.3144	4.3484	12.2874	10.2353	9.2071	8.4302	5.2067	0
GSM177937	0		0	0	0	0	3	4	53	9.11	0	8.55	1	8.55	1	9.7251	7.3573	4.0458	11.8459	10.3583	9.4347	8.4195	5.1387	0
GSM177938	1		0	1	0	0	1	2.5	49	3.02	0	3.02	0	3.02	0	10.3335	11.0122	3.8799	12.3937	10.2647	8.9933	9.4663	4.7847	0
GSM177939	0		1	0	0	0	3	3	43	12.85	0	12.85	0	12.85	0	13.0680	5.2064	4.0583	12.4223	10.2631	8.5683	8.5361	4.7019	0
GSM177940	1		0	1	0	0	2	2.2	39	14.35	0	4.41	1	14.35	0	9.8436	10.5671	4.7868	12.8113	10.8063	9.1274	9.2957	5.2830	0

GSM177941	1	0	1	0	0	2	2	47	13.98	0	13.98	0	13.98	0	9.5752	10.4651	3.7442	12.2952	10.7728	8.5380	8.7724	5.7902	1
GSM177942	1	0	0	0	0	3	1.9	42	7.70	0	7.70	0	7.70	0	10.9002	9.4065	4.3363	12.8106	10.0396	9.7971	8.9986	4.8217	0
GSM177943	0	0	0	0	0	2	1.2	39	9.03	0	9.03	0	9.03	0	8.4292	7.1184	4.4633	12.1790	9.5056	8.9054	8.6166	4.9345	0
GSM177944	1	0	0	0	0	2	3	52	9.74	0	1.50	1	9.74	0	7.1791	4.7646	3.6484	11.9432	11.1597	9.0000	8.4891	5.0201	1
GSM177945	1	0	1	1	0	2	1.5	50	14.88	0	9.66	1	14.88	0	9.6428	10.0500	4.8225	12.3734	9.9394	8.9699	8.5294	5.0408	0
GSM177946	1	0	0	0	0	3	2.2	36	14.32	0	2.34	1	14.32	0	8.9325	9.4889	4.2945	12.3495	10.7381	9.4924	9.7377	6.5497	1
GSM177947	1	0	0	0	0	2	2.5	45	14.56	0	4.97	1	14.56	0	10.0407	8.0191	4.6463	12.1624	10.4527	8.9340	8.9941	5.6683	1
GSM177948	1	0	0	0	0	1	1.7	50	13.49	0	13.49	0	13.49	0	9.4086	8.2353	3.9771	12.5809	9.6819	9.2711	8.4161	5.2895	0
GSM177949	1	0	0	0	0	3	2.3	39	13.73	0	10.56	1	13.73	0	9.5151	9.0424	3.6443	12.2949	10.0174	8.9398	8.8924	4.8051	0
GSM177950	1	0	0	0	0	1	3	44	12.72	0	12.72	0	12.72	0	9.9140	9.3411	4.7612	12.6588	10.4176	10.0692	8.4712	5.1201	0
GSM177951	1	0	1	1	0	1	1.3	50	9.07	0	9.07	0	9.07	0	9.4085	9.9794	5.3765	11.9217	10.3587	8.4348	8.9615	5.2502	0
GSM177952	1	0	0	1	0	2	1.8	51	13.32	0	13.32	0	13.32	0	8.3027	8.0464	4.8716	12.2493	10.3523	9.3091	8.6889	4.8455	0
GSM177953	1	0	1	0	0	1	2	49	9.17	1	4.88	1	4.97	1	10.2643	10.4940	4.0210	12.3332	10.6325	8.6074	8.9921	4.9681	0
GSM177954	0	0	0	0	0	2	2	37	2.87	1	2.20	1	2.20	1	9.3566	4.7078	4.0406	12.1193	10.2647	8.7219	9.0192	5.3813	0
GSM177955	1	0	0	1	0	2	1.8	51	5.77	0	5.77	0	5.77	0	9.8127	9.2424	5.2793	12.0556	10.0794	9.5396	8.8651	5.0968	0
GSM177956	0	0	0	0	0	3	2	40	2.55	1	1.45	1	1.45	1	9.1291	5.9391	4.3376	12.1910	9.2810	8.1822	8.2590	4.9709	0
GSM177957	1	0	1	0	0	1	2	44	13.23	0	13.23	0	13.23	0	9.5978	9.8145	3.9142	12.8012	10.5117	10.0816	9.5228	5.1858	0
GSM177958	1	0	1	0	0	3	2	49	5.88	1	4.72	1	4.72	1	9.0326	10.4751	4.0807	11.7486	10.4311	8.4474	8.9386	6.0800	1
GSM177959	1	1	0	0	0	1	1.5	53	12.93	0	12.93	0	12.93	0	12.0128	6.7219	4.0833	12.0403	9.7161	8.3182	8.8148	4.9213	0
GSM177960	1	0	1	0	0	2	2.9	44	6.72	1	3.45	1	3.45	1	9.7959	11.3672	4.1651	13.1244	10.8581	9.3617	9.1351	5.2392	1
GSM177961	1	0	0	0	0	2	2.3	48	12.16	0	10.89	1	10.89	1	9.6365	9.4949	4.7311	12.1918	10.4183	8.5093	9.3317	5.2382	0
GSM177962	1	0	1	1	0	2	2.5	57	12.24	0	9.13	1	11.10	1	10.1617	10.5083	6.9070	10.4653	9.8542	7.8892	8.7874	5.1951	0
GSM177963	1	0	0	0	0	3	3	45	4.77	0	4.77	0	4.77	0	8.0030	5.0209	3.9738	12.5875	9.5922	8.3252	8.5867	5.4318	0
GSM177964	1	0	0	1	0	1	2	44	9.88	1	8.82	1	9.88	1	10.2182	8.9029	5.1882	12.3137	10.2539	9.3916	9.1033	4.8527	0
GSM177965	1	0	0	1	0	2	1.5	39	10.68	0	6.95	1	10.68	0	9.6209	9.4321	5.6697	12.1938	10.6369	9.1570	9.2266	5.4660	0
GSM177966	0	0	0	0	0	3	3	40	4.26	0	4.26	0	4.26	0	8.9374	4.9537	3.9135	11.7020	9.2371	9.0251	8.7278	5.4969	0
GSM177967	1	0	1	0	0	3	2	38	9.71	0	9.71	0	9.71	0	10.2930	9.5497	4.0244	12.4580	10.0722	8.6532	8.9819	5.0956	0
GSM177968	1	1	0	0	0	1	2.6	45	10.19	0	10.19	0	10.19	0	12.4197	8.2285	3.8702	12.2384	10.2792	9.1916	9.1816	4.9350	0
GSM177969	1	0	1	0	0	2	2.6	43	10.33	0	10.33	0	10.33	0	9.2952	10.4817	4.7317	12.3775	9.8508	9.0216	8.2752	4.8295	0
GSM177970	0	0	0	0	0	3	1.5	47	9.02	0	9.02	0	9.02	0	9.6333	5.9227	3.7770	11.5808	9.6867	9.1123	8.4350	5.2438	0
GSM177971	0	0	1	1	0	2	1.8	38	10.41	0	8.47	1	10.41	0	8.9252	9.7189	6.5637	12.2258	10.3618	7.9925	8.8091	5.2950	0
GSM177972	0	1	0	0	0	2	2	43	12.21	0	12.04	1	12.10	1	12.4869	7.2457	3.5786	12.5562	10.7212	8.8952	8.7538	5.3396	0
GSM177973	1	0	1	0	0	3	3.1	60	2.82	1	1.78	1	1.78	1	9.9678	11.1941	4.4516	13.2274	11.0951	8.7216	9.4497	5.2781	1
GSM177974	1	1	1	0	0	2	2	42	13.57	0	13.55	1	13.57	0	12.2794	10.3486	4.0944	12.3143	10.3004	8.4915	8.9301	5.2361	0
GSM177975	0	0	0	0	0	3	2.1	46	8.18	1	4.64	1	4.64	1	9.8465	5.0532	4.0328	12.1879	10.0905	8.3953	8.4994	5.0115	0
GSM177976	0	0	0	0	0	2	1	47	12.61	0	10.21	1	12.61	0	10.2950	7.7735	3.5088	12.4835	10.5592	9.3196	9.1568	5.2079	0
GSM177977	0	1	0	0	0	3	1	54	13.31	0	13.31	0	13.31	0	12.1952	5.4951	4.3022	12.4798	10.2937	9.4513	8.6214	5.4782	0
GSM177978	0	0	0	0	0	3	2.2	47	12.82	0	12.82	0	12.82	0	7.7622	6.4097	4.2178	12.4835	10.2336	9.0347	9.2591	4.7880	0
GSM177979	0	0	0	0	0	3	5	39	8.12	1	7.02	1	8.12	1	8.3641	5.5891	4.0254	12.8662	10.7645	9.4065	9.2801	4.9773	1
GSM177980	0	0	0	0	0	3	3	32	12.59	0	12.59	0	12.59	0	8.5171	3.5674	3.7984	12.5487	10.2408	9.5335	8.3713	4.5780	0
GSM177981	0	1	0	0	0	3	2.5	57	3.30	1	2.84	1	3.05	1	13.9357	7.3109	3.9635	12.1921	9.9432	8.4353	8.7993	5.4617	0
GSM177982	0	0	0	0	0	2	3.8	47	5.25	1	3.13	1	5.25	1	10.2644	6.5939	3.2782	12.3154	10.3068	8.4648	8.7192	5.1184	0
GSM177983	1	0	1	1	0	2	1.8	54	5.78	1	2.73	1	4.74	1	9.8505	11.1750	6.5379	12.3590	10.3788	9.0281	8.9361	5.2172	0
GSM177984	1	0	0	1	0	1	1.8	54	11.85	0	11.85	0	11.85	0	10.9444	9.1058	5.4117	12.4921	11.1558	9.0978	9.2377	5.0533	1
GSM177985	0	1	0	0	0	3	4	45	11.92	0	11.92	0	11.92	0	11.8019	3.9299	3.5248	12.2806	10.7579	8.9958	9.0994	5.3470	0
GSM177986	1	1	0	0	0	3	2.2	43	11.45	0	5.71	1	11.45	0	13.3033	8.1525	3.9549	12.9343	10.7627	8.7959	8.6943	4.9331	1
GSM177987	1	0	1	0	0	1	1	48	8.73	0	8.73	0	8.73	0	10.5197	10.1643	4.4115	12.4340	10.4344	9.5074	8.8475	5.1979	0
GSM177988	1	0	0	0	0	3	2	43	7.32	0	7.32	0	7.32	0	6.7639	4.0823	3.1851	12.0221	9.8583	8.7949	8.8762	5.2500	0
GSM177989	1	0	0	1	0	2	2	43	2.18	1	2.00	1	2.00	1	10.3519	8.1159	5.6736	12.0897	10.3903	9.3402	8.5374	5.3712	0
GSM177990	1	0	0	0	0	1	0.8	50	4.36	0	4.36	0	4.36	0	9.7430	9.4968	4.1092	12.4305	10.0198	8.9019	10.0736	4.9421	0
GSM177991	1	0	0	0	0	1	0.8	43	5.26	0	5.26	0	5.26	0	9.9953	7.3340	3.6230	12.5162	10.6291	9.0636	9.2400	5.3961	0
GSM177992	1	0	0	1	0	2	1.3	49	7.25	0	7.25	0	7.25	0	10.7280	9.2261	5.4355	11.9730	9.5345	8.4800	8.5993	4.7381	0
GSM177993	0	0	0	0	0	2	2	38	1.13	1	0.74	1	0.74	1	9.1957	3.0512	3.0676	12.2628	10.1703	8.5002	9.3204	5.1004	0
GSM177994	0	0	0	0	0	2	0.9	47	3.53	0	3.53	0	3.53	0	9.9439	5.9669	3.4793	12.0587	10.9167	8.0560	9.4065	4.5640	0
GSM177995	1	0	1	0	0	2	1	54	12.52	0	12.52	0	12.52	0	10.9828	11.4002	3.5410	11.9622	11.5340	8.9816	9.1079	5.5315	1
GSM177996	1	0	1	1	0	1	1.9	52	12.43	0	12.43	0	12.43	0	10.2696	10.4397	4.9785	12.3938	10.2886	9.6204	8.9125	5.2652	0
GSM177997	1	0	1	1	0	2	1.4	51	13.79	0	13.79	0	13.79	0	8.8683	10.7398	5.4650	11.8247	10.3884	9.5048	8.3543	4.9244	0
GSM177998	1	0	1	0	0	2	0.9	50	8.79	1	4.01	1	7.13	1	10.1012	11.1265	4.3663	12.6423	10.6836	9.3475	9.4325	5.4475	0
GSM177999	0	0	0	0	0	2	4	43	1.35	1	1.08	1	1.08	1	8.8063	6.7712	3.9560	12.2917	10.0520	8.7615	9.3653	4.4836	0
GSM178000	1	0	0	1	0	2	0.9	24	22.61	0	9.62	1	18.57	1	9.5270	9.4528	4.8940	12.0810	10.1553	9.0193	8.5696	4.8261	0
GSM178001	1	0	0	0	0	3	1.4	40	6.98	0	6.98	0	6.98	0	8.9448	5.4904	4.2470	12.8770	9.8863	9.3901	8.2232	5.1082	1
GSM178002	1	0	1	1	0	2	1	56	11.19	0	11.19	0	11.19	0	9.3809	9.7682	5.7326	11.8320	10.1201	9.0454	8.3094	5.4030	0
GSM178003	0	0	0	0	0	3	2	43	7.92	1	7.92	1	7.92	1	8.7687	7.0587	4.1128	12.2828	10.1805	9.4174	8.7552	4.6391	0
GSM178004	1	1	1	1	0		1.1	50	14.35	0	6.69	1	14.35	0	13.1215	9.9432	5.1746	12.0176	10.1667	8.6308	8.6237	4.6441	0
GSM178005	1	0	1	0	0	1	1	54	14.81	0	14.81	0	14.81	0	9.0796	10.5526	4.2486	12.3908	10.1036	9.4796	8.7770	5.2414	0
GSM178006	0	1	0	0	0	3	2.2	45	11.72	0	11.72	0	11.72	0	12.8862	5.5786	4.3921	13.2209	10.8643	10.1805	8.0961	5.1014	1
GSM178007	1	0	0	0	0	2	1.2	43	14.68	0	4.16	1	14.68	0	9.0155	8.5473	4.7963	12.1047	10.3389	9.6736	8.8621	4.7052	0
GSM178008	1	0	1	0	0	1	2	49	10.86	0	10.86	0	10.86	0	10.3862	10.7998	4.6538	11.8778	10.0453	9.0000	8.7790	5.2798	0
GSM178009	0	0	0	0	0	3	3.8	53	1.76	1	0.76	1	0.79	1	8.8464	5.9371	3.9543	12.1043	10.7413	10.1446	7.7305	4.5658	0
GSM178010	0	1	0	0	0	3	1.1	39	17.25	0	17.25	0	17.25	0	13.0639	6.3906	4.4251	12.5368	10.2035	9.7739	8.9275	4.9608	0
GSM178011	1	0	0	0	0		2.2	38	17.53	0	13.62	1	17.53	0	8.9573	8.1723	4.3436	12.3757	10.4780	8.5117	8.7857	4.9005	0
GSM178012	1	0	0	0	0	3	2	38	15.57	0	15.57	0	15.57	0	10.3133	7.3152	4.4607	12.3691	10.7322	8.8366	8.5939	5.0223	0
GSM178013	0	0	0	0	0	3	1.2	40	17.10	0	17.10	0	17.10	0	9.2750	5.8335	4.1655	12.5408	8.6440	8.5797	8.3174	4.9480	0
GSM178014	1	0	1	1	0	2	1.5	38	16.88	0	16.88	0	16.88	0	9.3412	10.2674	6.1165	12.8198	10.7191	8.9736	9.5519	5.3824	1
GSM178015	1	1	1	0	0	2	1.7	54	16.80	0	2.44	1	16.80	0	13.2773	9.8165	4.0978	12.8172	10.1603	8.8129	8.2418	4.9961	0
GSM178016	1	0	0	0	0	2	2.1	40	14.62	1	11.54	1	11.54	1	9.0619	8.2187	3.7352	11.9643	9.2569	8.0937	8.0239	5.2945	0
GSM178017	0	0	0	0	0	3	1	56	13.64	0	13.64	0	13.64	0	9.6380	6.7077	3.5043	12.5866	10.1527	10.1231	8.2689	4.4333	0
GSM178018	1	0	1	0	0	2	2	45	4.67	1	4.67	1	4.67	1	10.4141	10.6829	3.6676	12.4038	10.6846	9.0217	8.9513	6.0770	1
GSM178019	1	0	1	1	0	2	2	46	13.74	0	13.74	0	13.74	0	9.3572	10.1867	5.0936	12.4173	10.1925	9.8143	8.9281	4.8882	0
GSM178020	1	1	1	0	0	3	1.6	45	5.27	1	2.41	1	2.41	1	11.2334	9.5757	4.8186	12.4001	10.2715	9.3882	9.0551	5.0120	0
GSM178021	0	0	0	0	0	3	2.8	33	23.87	0	23.87	0	23.87	0	7.6541	8.3167	4.2286	12.1596	10.3039	8.3477	9.1274	5.0714	0
GSM178022	0	0	1	1	0	3	2.4	37	12.80	0	12.80	0	12.80	0	10.0521	10.5393	5.4406	12.1400	9.5069	8.9863	8.4307	4.5758	0
GSM178023	0	0	1	0	0	3	2.5	42	19.02	0	19.02	0	19.02	0	10.2777	9.6900	4.8075	9.9295	9.6421	9.0647	8.0913	4.9738	0
GSM178024	1	0	0	1	0	3	1.1	47	16.81	0	16.81	0	16.81	0	10.8457	8.2605	5.7369	12.5614	10.7048	8.9199	8.8631	4.9420	0
GSM178025	1	0	1	1	0	2	2	43	4.94	1	2.21	1	2.21	1	9.7844	10.9623	5.5937	12.5145	11.0009	10.3052	9.1517	5.2767	1
GSM178026	1	0	1	0	0	3	1.6	44	16.21	0	16.21	0	16.21	0	7.7998	10.4414	4.0299	13.1561	11.4819	8.8689	9.4362	5.7812	1
GSM178027	1	0	1	1	0	2	2.3	54	16.19	0	16.19	0	16.19	0	9.0370	9.9061	4.8596	12.2579	10.3204	8.8378	8.6010	5.4650	0
GSM178028	0	1	0	0	0	2	1.8	51	6.02	1	6.02	1	6.02	1	13.1749	5.9189	3.8040	12.6352	9.8630	9.5627	8.7109	5.3443	0
GSM178029	1	0	0	0	0	2	2	42	16.05	0	16.05	0	16.05	0	9.3498	9.4486	3.9931	11.8255	9.5334	8.5531	8.2726	5.0682	0
GSM178030	1	0	1	0	0	2	2.2	56	10.00	1	6.44	1	6.44	1	10.0923	11.2971	3.7914	11.9431	10.4124	9.1014	8.5195	5.0541	0
GSM178031	1	0	1	1	0	2	2.5	56	15.83	0	15.83	0	15.83	0	9.4471	10.7713	5.9401	12.0264	10.1622	8.6377	8.9135	5.3481	0
GSM178032	1	0	1	0	0	3	2.3	58	15.83	0	15.83	0	15.83	0	10.5536	9.8425	3.5609	12.2415	10.8239	9.2564	8.5385	5.0411	0
GSM178033	1	0	1	0	0	2	2.2	58	15.68	0	15.68	0	15.68	0	9.6315	11.0497	3.9792	11.8244	9.3851	8.3575	8.5423	4.7740	0
GSM178034	1	0	0	0	0	2	1.5	32	15.65	0	15.65	0	15.65	0	8.2084	6.5967	4.4101	12.1043	10.3654	8.2225	9.0691	4.9280	0
GSM178035	1	0	0	0	0	3	1.9	50	15.49	0	15.49	0	15.49	0	9.5750	7.0846	4.2235	12.2346	10.6696	9.2951	9.2648	5.0439	0
GSM178036	0	1	1	1	0	3	1.3	49	0.40	1	0.34	1	0.34	1	12.3431	10.7501	5.5988	12.1405	10.2084	8.8494	8.7804	4.8974	0
GSM178037	1	0	1	1	0	1	1.9	47	15.56	0	15.56	0	15.56	0	10.1951	9.8515	5.7124	11.9714	10.4230	8.1841	8.8008	5.5164	0
GSM178038	1	0	1	0	0	2	2.2	56	7.39	1	4.77	1	4.77	1	10.3508	12.5831	4.1310	12.7970	11.0994	8.9171	9.6386	4.7947	1
GSM178039	1	0	1	0	0	1	1	55	9.90	1	2.37	1	9.90	1	10.1468	10.9758	4.5854	12.5203	10.4838	8.9244	8.2783	5.1266	0
GSM178040	1	0	1	1	0	2	1.8	48	15.35	0	15.35	0	15.35	0	9.9388	10.5594	5.2033	12.2542	9.5145	8.8669	8.5261	4.9327	0
GSM178041	1	0	1	1	0	2	0.9	56	15.80	0	15.80	0	15.80	0	9.4643	10.4053	6.7118	12.2254	9.8457	9.5386	8.6377	5.3498	0
GSM178042	1	0	1	1	0	2	2	51	15.57	0	15.57	0	15.57	0	9.3045	10.3525	5.2673	12.0001	10.3970	9.1015	9.0578	4.8883	0
GSM178043	1	0	1	0	0	3	1.2	44	15.15	0	15.15	0	15.15	0	9.6749	9.8897	4.5814	12.2792	10.2523	8.8072	8.7542	4.9223	0
GSM178044	0	0	1	0	0	2	1.2	46	15.21	0	0.64	1	15.21	0	9.8901	9.6029	4.7689	12.1507	10.0501	9.2853	9.9686	5.2669	0
GSM178045	1	0	0	0	0	1	1.2	36	17.40	0	17.40	0	17.40	0	9.3509	8.7141	3.5315	12.5102	10.5851	8.8143	8.5791	5.0442	0

GSM178046	1		0	0	1	0	2	1.3	44	17.25	0	1.98	1	17.25	0	9.3893	9.2678	6.9384	12.3492	9.8822	8.8308	8.3892	5.2779	0
GSM178047	1		0	0	0	0	2	2.2	41	19.89	0	18.05	1	19.68	1	10.6006	9.4597	4.5059	12.1098	9.7442	8.1740	8.7713	5.3464	0
GSM178048	1		0	1	0	0	2	3	60	17.21	0	17.21	0	17.21	0	10.0115	10.8964	3.9690	12.2737	10.7752	9.4629	9.0652	5.4452	0
GSM178049	1		0	1	1	0	2	3	59	12.59	1	9.08	1	9.08	1	9.6415	11.0980	6.0716	12.2844	10.3941	9.5954	8.7021	5.5975	1
GSM178050	0		1	1	0	0	3	2.7	48	16.45	0	16.45	0	16.45	0	11.7664	10.2932	3.8194	12.4065	10.2453	9.3377	8.5437	5.0112	0
GSM178051	1		0	1	0	0	2	3	53	8.14	1	2.65	1	8.14	1	9.6888	10.1577	3.8367	12.0276	10.3777	9.0058	8.9443	5.1457	0
GSM178052	0		1	0	0	0	3	4	43	17.07	0	17.07	0	17.07	0	13.4753	4.8283	3.7530	12.4377	10.3122	8.8957	8.3468	5.1549	0
GSM178053	1		0	1	0	0	2	1.5	33	24.95	0	5.09	1	24.95	0	9.9564	10.5986	4.2194	12.0682	10.4190	8.6003	9.0051	4.6490	0
GSM178054	1		0	0	1	0	1	2.6	42	14.92	1	11.93	1	11.93	1	9.6832	8.2678	5.7385	12.1342	9.9465	8.3389	8.4172	5.3722	0
GSM178055	0		0	0	0	0	3	4	51	10.72	0	10.72	0	10.72	0	6.5723	6.5489	4.3504	12.0605	9.2433	9.6657	8.6115	4.6759	0
GSM178056	1		0	1	1	0	3	1.7	31	14.19	0	14.19	0	14.19	0	9.8534	10.7965	5.4624	11.3943	9.5464	8.5687	8.4293	5.6508	1
GSM178057	1		0	1	1	0	3	3.2	60	10.56	0	10.56	0	10.56	0	7.2305	10.0816	4.9672	12.2421	9.5672	8.8164	8.6693	5.2505	0
GSM178058	1		0	0	0	0	3	2	46	14.13	0	14.13	0	14.13	0	10.1514	8.5666	4.5193	12.2314	9.6148	8.9925	8.1582	4.9819	0
GSM178059	0		0	0	0	0	3	2.1	59	13.00	0	13.00	0	13.00	0	8.8553	5.0655	4.0932	11.6518	10.0853	8.4687	7.8696	5.3480	0
GSM178060	1		0	1	0	0	1	3.2	24	3.58	1	2.18	1	2.18	1	9.5145	9.5306	4.3871	12.2391	9.8508	8.4729	9.1093	5.6594	1
GSM178061	1		0	1	0	0	2	3	54	5.11	1	4.38	1	4.38	1	9.4661	10.7896	3.5069	12.4275	10.5840	8.3501	8.6244	5.5667	0
GSM178062	1		0	0	0	0	2	0.8	55	13.80	0	13.80	0	13.80	0	9.8199	8.6475	3.5993	12.1153	9.7666	8.7600	8.3541	4.7059	0
GSM178063	0		1	0	0	0	3	2.1	41	13.35	0	13.35	0	13.35	0	12.6423	4.0177	3.7680	12.6714	9.8987	9.8042	8.8197	5.1795	0
GSM178064	1		0	1	0	0	3	2.5	42	12.48	0	12.48	0	12.48	0	9.7809	10.0449	4.0597	12.1875	9.9796	8.1469	8.9621	5.4051	0
GSM178065	0		1	0	0	0	3	2.5	47	12.29	0	12.29	0	12.29	0	11.5738	6.1492	4.1457	12.1494	9.9550	8.9557	8.5050	5.2416	0
GSM178066	0		1	0	0	0	3	2.2	53	11.77	0	11.77	0	11.77	0	13.4678	5.0001	3.9442	12.1189	10.1343	8.4039	8.2894	4.6339	0
GSM178067	1		0	0	1	0	2	2.4	50	13.33	0	13.33	0	13.33	0	9.9253	8.7807	5.0323	12.2350	10.0606	9.0118	8.2969	5.0303	0
GSM178068	1		0	1	0	0	3	2.2	44	11.45	0	11.45	0	11.45	0	9.4194	9.8480	3.7344	12.4331	10.3809	9.0409	9.1620	5.4736	0
GSM178069	0		0	1	0	0	1	1.5	48	16.47	1	7.33	1	12.65	1	8.4864	12.5950	3.9492	11.5778	10.3710	8.6414	8.5118	5.4707	0
GSM178070	1		0	1	1	0	2	3	49	11.10	0	6.96	1	11.10	0	10.3366	9.9870	4.8692	11.6776	10.5949	9.1318	9.3528	5.9086	1
GSM178071	1		0	1	0	0	2	2.5	43	10.36	0	3.92	1	10.36	0	10.0312	10.2507	4.1476	12.2464	9.5798	9.5372	8.3283	5.1074	0
GSM178072	1		0	1	1	0	1	2	45	9.57	0	9.57	0	9.57	0	8.8361	9.8255	6.4547	11.8881	10.1711	8.9226	9.1402	4.8630	0
GSM178073	0		1	0	0	0	3	1.4	53	7.02	0	7.02	0	7.02	0	13.0663	5.2473	3.8365	11.6143	10.1091	8.7047	8.7492	5.6611	1
GSM178074	1		0	0	0	0	3	4.2	40	14.48	1	6.72	1	6.72	1	9.3490	8.6048	3.4740	12.2652	10.6465	9.7673	8.4074	5.2680	0
GSM178075	0		0	0	0	0	3	3.2	48	7.89	0	7.89	0	7.89	0	9.3925	6.8332	4.2604	11.9235	10.2182	8.5158	9.0808	4.8289	0
GSM178076	1		0	0	0	0	2	1.5	56	14.07	0	14.07	0	14.07	0	9.4296	9.4134	4.7103	11.9252	9.5886	8.8974	8.4243	4.7947	0
GSM178077	1		0	0	0	0	2	1.7	45	19.24	0	19.07	1	19.24	0	8.6703	7.6229	3.6793	12.2146	9.5870	9.9626	8.9422	5.3559	0
GSM178078	0		0	0	0	0	3	2.2	39	15.54	0	15.54	0	15.54	0	9.2692	6.1135	4.2456	11.7954	9.8112	8.5970	9.5094	5.6366	1
GSM178079	0		0	0	0	0	3	3.2	46	3.41	1	1.11	1	1.11	1	8.6705	5.4988	3.6915	12.1594	10.5181	8.6212	9.3660	5.8360	1
GSM178080	1		0	1	1	0	1	2.5	47	6.10	0	6.10	0	6.10	0	9.5377	10.2285	5.5977	12.0633	10.0513	8.4409	8.5037	5.1300	0
GSM178081	1		0	1	0	0	2	1.2	43	7.46	0	7.46	0	7.46	0	10.2581	9.8914	4.7352	11.5581	10.3218	7.7132	9.0274	5.3917	0
GSM178082	0		0	0	0	0	3	2.5	39	4.88	0	3.68	1	4.88	0	8.3595	5.8283	4.0541	10.8124	10.4135	8.7038	8.1112	6.2235	1
GSM232194	1	0	1	1	0	1		4.2	64			10.55	0	10.55	0	12.1105	11.8941	4.1655	12.3252	10.6116	8.6519	9.0063	5.9044	1
GSM232195	1	1	0	1	1	1	1	3	73			10.85	0	10.85	0	9.8152	12.6739	5.9091	11.7989	10.9978	9.0230	9.0019	5.6945	1
GSM232196	1	1	0	1	1	0	1	1.83	66			10.78	0	10.78	0	9.1901	13.3730	8.1540	12.9593	11.3326	11.5787	8.9346	5.8995	1
GSM232197	1	0	0	1	0	0	3	1.7	66			9.89	0	9.89	0	10.6407	13.1733	4.1367	12.7708	10.9736	8.4543	9.1505	5.6768	1
GSM232198	1	1	1	1	1	1	3	2.1	75			5.73	1	5.73	1	12.4548	13.2604	7.2226	12.8460	11.6488	8.3285	9.2176	5.6558	1
GSM232199	1	0	0	0	0	0	3	1.6	73			11.23	0	11.23	0	8.5679	8.2093	4.4233	12.4034	10.2194	8.4737	8.8833	5.3164	0
GSM232200	1	0	1	1	0	1	3	2.1	63			10.94	0	10.94	0	11.3025	12.2643	3.8457	12.2809	10.7615	8.1891	8.9994	5.9857	1
GSM232201	1	1	0	1	1	1	3	3.1	71			11.07	0	11.07	0	9.9213	12.0826	7.7002	12.4991	11.1857	8.7740	9.6929	6.0803	1
GSM232202	1	1	0	1	1	1	1	1.7	72			10.08	0	10.08	0	10.4963	11.9357	6.3195	12.2650	10.7327	8.7220	9.5023	5.5317	0
GSM232203	1	1	0	1	1	1	2	2.28	72			11.30	0	11.30	0	9.6048	12.0286	5.6717	12.5070	10.9338	9.4252	9.0831	5.8538	1
GSM232204	1	0	0	1	0	0		1.5	82			10.54	0	10.54	0	9.9654	9.6347	3.6008	12.6148	10.3860	9.3183	8.9333	5.5263	0
GSM232205	1	1	0	1	1	1		1.8	62			10.64	0	10.64	0	9.6650	11.5134	9.6037	12.5335	10.8435	9.1495	9.3201	5.6187	1
GSM232206	1	0	0	1	0	0	2	2.3	58			10.17	0	10.17	0	10.0395	12.2159	3.7548	12.2201	10.9490	8.4679	8.8022	5.8495	1
GSM232207	1	1	0	1	0	0	3	2.5	60			10.00	0	10.00	0	9.3829	10.9386	4.3472	12.2419	10.5742	7.7841	9.4378	5.5691	0
GSM232208	1	1	0	1	1	1	1	1.57	49			10.59	0	10.59	0	9.9932	12.3099	7.5796	12.7790	10.7288	10.1438	8.7623	5.5619	0
GSM232209	1	1	0	1	1	0	1	1.38	59			10.52	0	10.52	0	9.7355	12.3798	7.8546	12.3371	10.6432	8.5957	8.9331	5.6469	1
GSM232210	1	1	0	1	1	0	3	2.3	51			8.56	0	8.56	0	10.6617	12.2046	7.2164	12.6232	10.8621	9.0745	9.1827	5.2534	0
GSM232211	1	1	0	1	1	1	2	2.9	55			6.62	1	6.62	1	10.0424	12.2227	6.3543	12.2261	10.6607	8.5817	9.2979	6.0047	1
GSM232212	1	1	0	1	1	0	3	3	76			9.12	0	9.12	0	8.3220	12.6178	6.3541	11.7296	10.9965	9.5252	9.3081	5.6136	1
GSM232213	1	1	0	1	1	0	1	1.08	49			10.04	0	10.04	0	9.9201	11.3973	5.9304	11.8990	10.9957	8.6953	9.0610	5.5003	1
GSM232214	1	1	0	1	1	0	2	2.6	69			7.23	0	7.23	0	10.3806	13.1968	6.9504	11.8952	11.5413	8.5311	9.4873	5.9627	1
GSM232215	1	0	0	1	0	0		2.5	74			10.19	0	10.19	0	10.2624	11.2368	3.9859	12.0825	10.1767	8.7418	9.9255	5.7615	1
GSM232216	1	1	0	1	1	0	2	1.97	42			9.01	0	9.01	0	9.1631	12.4490	7.3425	11.9960	10.2336	8.7975	9.1205	5.5638	0
GSM232217	1	0	0	1	0	0	3	1.8	62			10.12	0	10.12	0	10.5770	12.4839	3.1785	12.2466	10.9883	8.0606	8.9888	6.0379	1
GSM232218	1	1	0	1	1	1	1	2.6	51			9.47	0	9.47	0	10.2097	12.0483	6.1006	12.4271	10.8357	8.8550	9.3564	5.6166	1
GSM232219	1	1	0	1	1	1	2	2	52			8.20	0	8.20	0	10.2996	12.8853	8.9668	11.1592	11.0476	9.7861	8.8753	5.5036	1
GSM232220	1	1	0	1	1	0	3	2.25	59			8.43	0	8.43	0	9.8022	14.4349	6.1596	12.4362	9.8207	9.7476	8.4103	5.3784	0
GSM232221	1	0	0	1	0	1	3	6	58			4.42	1	7.39	1	9.2281	12.9501	3.8891	12.6302	11.0056	9.3544	9.6247	6.0029	1
GSM232222	1	0	0	1	0	1	3	2.1	65			3.50	1	3.66	1	9.8892	12.5239	4.3826	11.6013	9.8540	8.2976	9.1422	5.5427	0
GSM232223	1	1	0	1	1	1	2	2.85	50			10.68	0	10.68	0	9.1479	12.1804	7.7432	12.0676	10.0483	8.6317	9.3602	5.7875	1
GSM232224	1	1	0	1	1	1	3	2	60			0.58	1	9.12	0	9.7028	12.5655	5.5744	12.2563	9.4300	7.8003	8.7271	5.3215	0
GSM232225	1	1	0	1	1	0	3	2.2	68			9.64	0	9.64	0	8.8621	12.5943	8.0607	12.4017	10.8765	8.4410	9.3710	5.7186	1
GSM232226	1	0	0	1	0	0		2.4	59			8.41	0	8.41	0	9.6252	11.8924	3.9594	12.6768	11.0197	9.6136	9.0603	5.9841	1
GSM232227	1	1	0	1	0	0	3	3	61			8.92	0	8.92	0	9.6616	12.7996	4.1394	12.2381	10.5839	8.8801	9.0773	5.3073	0
GSM232228	1	1	0	1	1	1	3	2.85	68			6.87	0	6.87	0	10.0651	13.2248	5.9410	12.5571	11.8443	9.5128	9.9120	5.6196	1
GSM232229	1	0	0	1	0	0		2.1	64			9.27	0	9.27	0	10.0690	12.3617	3.8015	12.1722	10.7197	8.8126	9.2257	5.6100	1
GSM232230	1	0	0	1	1	1	1	2.8	75			8.47	0	8.47	0	9.8623	13.0880	5.0638	12.4880	11.3155	8.9217	9.5111	6.0254	1
GSM232231	1	1	0	1	0	1		4	77			2.08	1	2.11	1	9.2226	12.4016	4.7815	12.9751	10.9470	9.0858	9.4439	5.5651	1
GSM232232	1	1	0	1	1	0		1.9	52			7.85	0	7.85	0	9.2634	13.2123	6.3860	12.4361	10.7942	9.5980	9.4403	5.7026	1
GSM232233	1	1	0	1	1	0		2	74			9.16	0	9.16	0	9.4773	11.7446	6.4041	11.8341	10.6880	8.4222	9.4300	5.2662	0
GSM232234	1	1	0	1	0	0	2	1.3	60			7.75	0	7.75	0	9.9655	11.4300	3.7266	12.4257	10.8035	8.0139	8.7938	5.7004	1
GSM232235	1	1	0	1	0	1		5	63			8.33	0	8.33	0	9.9197	10.7225	4.5562	12.4545	10.8059	9.4133	8.5795	5.7304	1
GSM232236	1	0	0	1	0	0	3	4	70			1.28	1	1.28	1	10.1573	11.7238	4.2516	12.4841	10.3671	8.8138	8.7844	5.1042	0
GSM232237	1	1	0	1	1	0		1.6	47			8.53	0	8.53	0	10.0655	11.4786	6.2660	13.0812	11.7887	8.3971	9.1399	5.6268	1
GSM232238	1	1	0	1	1	0	2	3	75			7.61	1	7.61	0	9.9241	13.5844	7.3747	11.9425	10.7399	8.7236	9.1593	6.0676	1
GSM232239	1	1	0	1	1	0	2	1.8	69			8.85	0	8.85	0	9.7533	12.9644	8.5604	11.7958	10.4427	8.6262	9.1650	5.6285	1
GSM232240	1	0	0	1	0	1	3	2.1	58			8.96	0	8.96	0	10.0091	12.2029	3.9826	12.5578	10.8071	8.3886	9.2516	5.4068	0
GSM232241	1	1	0	1	1	0	2	4	75			7.23	0	7.23	0	10.3903	12.7116	5.2518	12.3381	10.5642	8.7626	8.5896	5.4793	0
GSM232242	1	1	0	1	1	0	2	2	56			8.30	0	8.30	0	9.4934	11.2761	5.7321	12.6675	10.9830	9.0767	9.1618	5.4413	1
GSM232243	1	1	0	1	1	0		5	77			5.43	0	5.43	0	9.0223	13.1961	6.9474	12.7021	10.8414	9.3498	8.3758	5.7970	1
GSM232244	1	1	0	1	1	0	3	1.6	59			8.41	0	8.41	0	10.3797	12.7151	9.0074	12.5959	10.9202	8.8854	8.9966	5.6700	1
GSM232245	1	0	0	1	0	0	3	2.3	65			8.19	0	8.19	0	9.5615	12.9863	3.5415	12.7325	11.2953	8.7402	9.2739	4.9706	1
GSM232246	1	1	0	1	1	1		1.9	67			8.30	0	8.30	0	10.4640	13.1124	6.0788	12.2169	10.3582	8.1478	8.6885	5.5890	1
GSM232247	1	1	0	1	1	0	1	1.8	65			7.63	0	7.63	0	9.5451	12.2619	8.0100	12.3636	10.1561	6.4733	9.0752	5.1858	0
GSM232248	1	1	1	1	1	0	2	1.2	50			8.07	0	8.07	0	12.9877	11.6279	5.0759	12.0865	10.4406	9.2836	8.8379	5.5161	0
GSM232249	1	1	0	1	1	1		2	73			7.12	0	7.12	0	8.7511	12.8324	4.9666	12.5977	11.0522	9.9218	9.2966	5.8447	1
GSM232250	1	1	0	1	1	1	3	2.8	70			7.41	0	7.41	0	10.5556	11.6896	7.4156	12.9870	10.5629	8.4870	8.8466	4.8182	1
GSM232251	1	1	0	1	1	0		1.07	52			7.10	0	7.10	0	11.0227	12.4862	6.5169	11.9570	10.6151	8.9518	9.1259	5.3307	0
GSM232252	1	1	0	1	1	1	1	2.9	80			3.08	1	3.08	0	10.2371	12.0895	6.8252	12.5869	10.2707	9.0450	8.6186	5.2322	0
GSM232253	1	1	0	1	1	1	2	3.8	80			7.92	0	7.92	0	9.9408	13.1197	5.8209	11.8793	10.6603	8.9382	8.6489	5.7567	1
GSM232254	1	1	0	1	0	0	1	2.5	74			7.30	0	7.30	0	9.7651	11.4617	3.2866	12.9382	10.5569	9.4446	8.3889	5.5264	1
GSM232255	1	1	0	1	1	1	1	1.69	76			6.08	0	6.08	0	9.8407	11.5351	7.8146	12.2521	10.0404	9.3844	8.2605	5.7308	1
GSM232256	1	1	0	1	0	1	1	1.8	55			7.00	0	7.00	0	10.0430	13.1958	4.7678	12.7176	10.3880	7.9798	8.6053	5.7548	1
GSM232257	1	1	0	1	1	1		2.3	51			8.08	0	8.08	0	10.1567	11.1880	6.5617	12.8796	10.3375	9.0417	8.7782	5.0499	1
GSM232258	1	1	0	1	0	1	2	1.8	70			7.08	0	7.08	0	9.6648	12.6417	4.0721	12.9183	11.2060	10.1345	8.9649	5.8630	1
GSM232259	1	0	1	0	0	0		1.1	59			2.84	0	2.84	0	13.0022	9.3484	4.4637	12.6586	10.1195	9.1579	8.0700	5.6663	1
GSM232260	1	1	0	1	1	1	3	1.5	73			2.41	1	2.41	1	8.5560	9.8330	5.7959	12.0642	10.2801	8.9380	8.6213	5.0515	0
GSM232261	1	1	0	1	1	1	2	2.1	71			7.50	0	7.50	0	10.6147	13.1716	6.9722	13.0965	11.0462	9.4252	8.5653	5.6623	1

GSM232262	1	1	0	1	1	0		2.55	67	6.36	0	6.36	0	9.1470	13.5222	6.8941	12.0839	9.8571	9.1126	9.1144	5.3975	0
GSM232263	1	0	0	1	0	0	3	2.7	58	2.90	1	2.90	1	9.9228	11.8072	3.8360	12.4487	10.7621	9.1491	8.9738	5.2798	0
GSM232264	1	1	0	1	1	1	3	3.1	72	8.34	0	8.34	0	9.7317	13.5419	5.7206	12.6841	11.0855	9.0328	9.1551	5.8795	1
GSM232265	1	1	0	1	1	1	2	4.5	55	5.00	1	5.00	1	9.1115	13.3794	5.8038	12.2636	11.0810	9.1948	9.7656	6.0906	1
GSM232266	1	1	0	1	1	0	2	1.8	52	5.95	0	5.95	0	10.5467	13.4095	5.3041	11.8903	10.5896	8.3931	9.7910	5.8848	1
GSM232267	1	1	1	1	1	1	2	2.5	64	4.46	1	5.28	1	12.6054	13.4567	5.5878	12.6917	10.7784	7.7643	8.7712	5.3265	0
GSM232268	1	1	0	1	1	1		1.85	68	6.36	0	6.36	0	9.7368	13.1759	8.6882	12.4273	10.9500	9.3453	9.1583	6.1662	1
GSM232269	1	1	0	1	1	0	2	2	63	6.95	0	6.95	0	9.7690	13.3192	4.8612	12.5693	10.6500	8.5355	8.0731	5.5480	0
GSM232270	1	1	0	1	1	0	1	1.1	71	6.14	0	6.14	0	9.8041	11.6825	7.4577	13.0238	10.4209	10.2376	9.1266	5.6115	1
GSM282373			1	0	0	0	2	1.8				7.67	1	12.4521	5.6707	3.3916	12.3605	9.6527	9.4650	8.5846	5.4570	0
GSM282374			0	1	0	0	3	2.5				5.92	0	10.1452	11.4378	3.9163	12.3853	10.2865	9.7948	8.8390	5.6111	1
GSM282375			0	0	0	0	3	1.5				4.83	1	10.0219	8.1687	4.2304	12.3305	10.2235	9.3857	8.6263	5.3590	0
GSM282376			0	1	1	0	2	1.2				5.67	0	9.3630	9.7595	5.1373	12.5269	10.0494	9.4313	8.7757	5.2782	0
GSM282377			0	1	1	0	2	2.4				8.58	0	9.1572	10.6447	4.8498	12.5661	9.9759	8.5810	9.0382	5.1947	0
GSM282378			0	1	1	0	2	1.8				7.75	0	9.2667	11.5577	8.0181	12.1421	9.3046	8.9200	8.5856	4.8447	0
GSM282379			0	1	0	0	2	1.4				9.25	0	10.1058	11.7914	4.5772	12.2545	9.8585	8.9457	8.7341	5.2513	0
GSM282380			1	1	0	0	3	1.3				7.08	0	12.3290	9.6256	4.1831	12.3436	10.0238	8.7144	7.9740	4.6707	0
GSM282381			0	1	1	0	2	0.8				8.17	0	9.9447	12.1812	6.3005	12.7829	10.3219	9.2060	8.5328	5.1582	0
GSM282382			0	1	0	0	1	1.6				6.75	0	10.2036	10.2626	3.4385	12.0818	10.2166	8.4756	9.0006	4.9861	0
GSM282383			0	1	0	0	2	1.4				6.58	0	9.8707	10.4487	3.9430	12.6086	10.3905	9.1967	8.9797	4.7480	0
GSM282384			0	1	1	0	2	1.8				6.67	0	10.0174	11.0079	6.7402	12.7991	9.7145	9.7465	8.7668	5.2264	0
GSM282385			0	0	0	0	2	2.2				7.42	0	9.8080	8.2784	4.2850	12.3511	10.5480	9.8421	10.1527	5.2058	0
GSM282386			0	1	1	0	2	2.3				7.00	0	8.7405	11.3606	5.8210	12.6684	10.4157	9.0991	9.3483	5.1455	0
GSM282387			0	1	1	0	2	2.3				1.17	0	10.0891	11.1176	5.8375	12.3220	10.0341	9.0715	8.6540	5.2011	0
GSM282388			0	1	1	0	3	1.8				7.33	0	10.2427	10.6932	5.7037	12.3190	10.4526	9.2480	9.5393	4.6857	0
GSM282389			0	1	1	0	2	1				6.67	0	10.1204	12.7429	6.8044	12.2175	9.8290	9.4585	8.7086	4.2179	0
GSM282390			0	1	1	0	2	0.9				5.75	0	9.4932	9.6669	6.1867	12.0251	9.9223	9.2892	8.4750	5.0450	0
GSM282391			0	1	1	0	2	4.4				7.75	0	9.6488	9.6238	5.7928	12.3013	10.3577	8.3603	9.4063	4.9666	0
GSM282392			0	1	0	0	2	1.8				7.33	0	11.1027	10.7356	4.1322	12.3724	9.7867	8.7983	8.8480	5.0777	0
GSM282393			0	1	1	0	1	1.2				8.00	0	9.7877	9.5227	5.0695	12.4791	10.3202	9.6858	8.6595	5.2188	0
GSM282394			0	1	0	0	2	1.6				6.50	1	10.5008	12.4722	4.0757	12.5860	10.8256	8.6727	9.5466	5.1823	0
GSM282395			0	1	1	0	2	1.8				6.58	0	9.3698	11.0913	6.3816	12.4781	10.2752	9.5777	8.5227	4.7543	0
GSM282396			1	1	1	0	2	1.3				2.17	1	12.9152	11.3907	5.2662	12.5581	9.9556	8.9392	9.2211	4.8217	0
GSM282397			0	1	1	0	2	1.1				6.83	0	10.4744	11.7602	6.5728	12.3014	10.0478	9.8699	8.5873	4.5954	0
GSM282398			0	0	0	0	3	3.5				1.25	1	9.2023	7.0774	4.4446	12.2075	10.1681	9.1225	8.8356	4.4623	0
GSM282399			0	1	1	0	2	1.6				6.00	0	10.5279	11.5724	6.0320	12.9664	10.6118	9.1928	9.3205	5.3539	1
GSM282400			0	1	0	0	3	2.2				7.42	0	9.0864	10.6137	4.8106	12.4848	10.4807	9.1207	8.9216	4.6139	0
GSM282401			0	0	0	0	1	2.2				6.50	0	9.6505	8.8716	3.6173	12.4436	10.8540	9.5328	8.8463	5.2728	0
GSM282402			0	1	0	0	1	1.5				5.75	0	10.1369	11.2390	4.5618	13.2382	11.1812	8.8815	8.7786	4.9381	1
GSM282403			0	1	1	0	2	1.6				1.58	0	9.8519	11.6713	6.7131	12.5653	10.1648	8.9257	8.9643	4.9877	0
GSM282404			0	1	1	0	2	2.5				6.08	0	10.6648	11.4251	5.4586	12.1719	10.3303	9.0606	9.2228	5.0859	0
GSM282405			0	1	1	0	2	1.5				5.33	0	9.8873	11.4422	7.9194	12.5141	10.1508	9.0997	8.8419	4.6185	0
GSM282406			0	1	1	0	3	2.2				7.08	0	8.3673	10.7091	6.6085	12.5547	10.4561	10.5934	8.9189	4.1790	0
GSM282407			0	1	0	0	2	1.5				5.92	0	9.5411	9.9725	3.7006	12.8973	11.0930	8.4618	8.8496	4.5378	1
GSM282408			0	1	0	0	2	5.5				6.25	1	9.7389	10.4913	4.2131	12.5382	11.0836	9.6530	8.9690	5.1364	1
GSM282409			1	0	0	0	2	0.1				7.92	0	12.8683	3.9798	3.5531	12.8581	10.2787	9.9505	8.6711	4.6533	1
GSM282410			1	0	0	0	3	2.8				3.08	1	12.7551	7.0716	4.0214	12.9627	10.6463	8.8725	8.3390	4.8428	1
GSM282411			0	1	0	0	1	1.2				7.92	0	10.2929	10.2556	4.5376	12.4902	10.5642	8.7991	9.1209	4.8127	0
GSM282412			0	1	0	0	2	1.7				9.25	1	9.9473	10.4534	4.2978	12.4262	10.2600	9.0862	9.4309	4.9664	0
GSM282413			0	0	0	0	3	1.7				1.42	1	8.1731	5.5425	3.3692	12.2346	10.5874	9.3804	8.8675	5.4005	0
GSM282414			0	1	1	0	2	1.2				5.75	0	10.0119	10.8159	7.1836	12.5628	10.2215	8.6978	9.4008	5.0252	0
GSM282415			0	1	0	0	2	1.9				6.58	0	11.0216	11.0773	4.5854	12.4578	10.1318	7.7935	9.0261	5.0126	0
GSM282416			0	1	0	0	2	1.3				7.58	0	9.4574	10.4562	3.5375	12.8368	11.1948	8.2234	9.1187	5.1551	1
GSM282417			0	1	1	0	2	1.6				6.00	0	10.5315	11.3073	5.3546	12.3688	10.2317	9.5983	9.5382	5.0185	0
GSM282418			1	0	0	0	3	0.3				2.92	1	11.4617	8.3654	3.1595	12.0152	9.9393	9.4069	8.2103	5.5320	0
GSM282419			0	1	1	0	2	0.8				6.17	0	10.3287	11.1129	7.4275	12.5301	10.4360	9.3420	8.3997	5.0335	0
GSM282420			0	0	0	0	2	1.1				10.92	0	9.7504	9.2471	4.3873	12.0863	10.1111	9.0583	8.5629	5.0690	0
GSM282421			0	1	0	0	1	2.2				9.92	0	9.3284	10.1781	3.6203	12.6036	10.4956	8.4665	8.8616	4.8164	0
GSM282422			0	1	0	0	2	1.4				10.92	0	10.0799	10.5840	4.5787	12.4930	10.6753	9.8493	9.2850	4.9595	0
GSM282423			0	1	1	0	2	4				5.50	0	9.6843	10.1121	4.8594	12.3884	10.6014	8.5039	9.4186	4.3200	0
GSM282424			0	1	1	0	2	2.2				7.58	0	9.9639	11.4420	7.5460	12.2219	10.0304	8.4700	9.4977	4.8037	0
GSM282425			0	1	1	0	2	1.6				8.17	0	9.9449	10.9557	5.6940	12.3299	10.0914	8.9611	9.5507	4.7749	0
GSM282426			0	1	1	0	2	2.5				3.33	1	9.8592	10.8933	6.6473	12.1039	10.3992	8.3834	9.5693	4.8456	0
GSM282427			0	0	0	0	3	2				10.00	0	8.6927	8.3124	3.9691	12.1050	9.4357	8.2960	9.3152	4.7412	0
GSM282428			0	1	0	0	2	1.8				6.58	0	9.1265	11.6513	4.2794	12.6159	10.6201	9.1666	8.8035	5.4523	0
GSM282429			0	1	1	0	3	1.8				10.17	0	8.5515	9.6217	5.5584	12.2034	10.1106	8.7172	8.5648	4.7739	0
GSM282430			0	0	0	0	2	4				9.92	0	10.1130	8.3084	3.1214	12.6569	10.2654	8.6365	9.7714	5.1597	0
GSM282431			0	1	1	0	2	1.8				3.67	1	10.1645	9.5831	5.3485	12.0760	9.2273	9.3979	8.3992	5.6133	1
GSM282432			0	0	0	0	2	1.6				9.42	0	10.1447	8.9319	4.6105	12.3460	10.1520	9.4523	8.5742	5.2625	0
GSM282433			0	1	1	0	2	2.2				9.75	0	9.0236	10.1237	6.9058	11.9871	9.4505	8.1956	8.9468	5.0733	0
GSM282434			0	1	0	0	2	2.5				5.50	0	8.9862	10.7362	4.7410	11.9027	10.0819	8.8891	8.5589	5.2344	0
GSM282435			0	0	0	0	3	4				9.58	0	8.9696	8.1888	3.9817	12.3764	10.3525	9.0649	9.2120	5.4442	0
GSM282436			1	1	0	0	2	2.4				5.33	0	11.5402	11.6047	4.1215	12.2427	10.3329	8.9073	8.6826	4.7290	0
GSM282437			0	1	1	0	2	1.4				9.83	0	10.0840	11.4896	5.4135	12.4480	10.5051	8.5027	9.1843	4.9350	0
GSM282438			0	1	0	0	2	1.3				7.75	0	8.1029	10.2370	4.2322	12.5562	10.5648	9.4230	8.5404	4.5739	0
GSM282439			0	1	0	0	2	2.7				8.25	0	10.0809	11.0627	4.8169	12.2676	10.1789	8.6898	9.3893	4.8996	0
GSM282440			0	0	0	0	3	2.2				8.83	0	8.7247	7.5138	4.2517	12.5486	10.5781	8.9577	8.4932	4.9852	0
GSM282441			0	1	1	0	2	1.3				7.17	0	10.0537	11.2533	5.0825	12.3436	10.1560	9.2975	8.9980	5.0040	0
GSM282442			0	1	1	0	2	4				4.25	0	10.8440	11.7638	7.3956	12.5506	10.3553	8.7575	9.3630	5.2968	0
GSM282443			0	1	1	0	1	2.4				10.17	0	10.2037	10.9751	6.3861	12.2897	10.3959	8.8357	9.3685	5.2216	0
GSM282444			0	1	1	0	2	2.2				10.67	0	10.1809	11.5406	7.6704	12.2485	10.5012	9.1785	9.1663	5.0477	0
GSM282445			0	0	0	0	1	1.1				10.33	0	9.6932	9.2486	3.5116	12.0535	10.3466	8.6322	8.9939	4.6892	0
GSM282446			0	0	0	0	2	1.7				9.50	0	9.7358	4.5412	3.3352	12.2600	10.6771	9.2956	9.5742	4.9628	0
GSM282447			0	0	0	0	2	1.5				9.42	0	9.3107	8.9935	3.8269	12.3168	10.5692	9.0412	8.7410	5.1068	0
GSM282448			1	0	0	0	3	0.5				10.17	0	12.8799	7.4708	3.7091	12.3796	10.4244	9.2923	8.3566	5.2379	0
GSM282449			0	1	1	0	2	0.9				10.33	0	9.9505	10.2746	5.9808	12.5242	10.5438	8.9696	9.2492	5.4596	0
GSM282450			0	1	1	0	2	3.4				9.58	0	10.1392	12.5885	5.6107	12.2281	10.4823	8.4595	9.2279	5.0155	0
GSM282451			0	1	1	0	2	2				3.75	0	9.7954	10.6314	7.3790	12.0903	10.6764	8.7423	9.1089	4.6466	0
GSM282452			0	1	1	0	2	2				5.83	0	10.8403	10.3859	7.9029	12.3930	10.7681	8.3914	8.8602	5.0688	0
GSM282453			1	0	0	0	2	0.1				11.75	0	12.8509	8.1511	4.2031	12.4785	10.8652	8.7189	9.1104	5.2477	0
GSM282454			0	0	0	0	3	1				0.67	1	8.4585	6.7935	3.6655	12.3208	10.3714	9.3935	8.9779	5.1716	0
GSM282455			0	1	1	0	1	0.2				12.17	0	9.7511	11.2876	6.3871	12.1013	10.5834	8.5305	8.5967	4.8492	0
GSM282456			0	0	0	0	2	1.8				10.92	0	9.8183	8.7796	3.7596	12.0321	9.7863	8.8481	8.4734	5.3870	0
GSM282457			0	0	0	0	3	1.8				12.33	0	8.9823	6.6984	3.7199	12.0520	9.4280	8.7708	8.5135	4.8812	0
GSM282458			0	0	1	0	1	3				11.08	0	10.3691	8.9589	5.6302	12.3553	10.3314	9.1171	8.7509	5.4814	0
GSM282459			0	0	1	0	1	3.5				13.25	0	10.0737	7.2592	5.6936	12.3249	10.1306	9.2732	8.7574	5.0658	0
GSM282460			0	0	0	0	2	1				12.42	1	9.7401	9.3322	4.3033	12.5224	10.6338	9.1754	9.6351	5.0129	0
GSM282461			1	1	0	0	3	1.5				2.50	1	13.1142	9.6435	3.7042	12.7215	10.3703	9.4586	8.4181	5.0416	0
GSM282462			0	0	1	0	2	2				15.42	0	9.4930	9.3623	5.2243	12.1188	10.1087	8.5716	8.8956	5.1454	0
GSM282463			0	1	1	0	1	1.7				4.08	0	9.8941	10.0637	6.6497	12.5582	10.6308	9.7162	8.5687	5.2131	0
GSM282464			0	0	0	0	3	5				16.58	0	8.9734	5.5917	3.7626	12.3776	9.2608	9.5505	8.3402	5.1002	0
GSM282465			0	0	0	0	3	3.5				7.50	0	8.8134	7.5622	4.0118	11.9802	9.8111	8.1535	8.2736	4.3252	0
GSM282466			0	0	0	0	1	1.5				15.08	0	10.0738	8.1456	3.3105	12.4740	10.3097	9.4866	8.5086	5.2598	0
GSM282467			0	0	0	0	2	1				15.83	0	10.3329	7.4975	4.0846	12.4357	10.5955	9.4338	8.7989	5.1357	0
GSM282468			1	1	0	0	2	1.5				10.17	1	12.1684	10.5081	4.1482	11.7047	9.9252	8.4397	8.2967	4.7891	0

GSM282469	0	1	1	0	2	1	12.67	0	10.3655	9.8417	5.5151	12.2889	10.4647	9.2457	8.8033	5.0536	0
GSM282470	0	0	0	0	2	3.5	1.42	1	10.1908	8.3549	3.5570	12.3606	10.3165	8.9810	8.5998	5.2128	0
GSM282471	0	0	0	0	3	3.5	1.92	1	9.3844	6.0757	4.3291	12.0432	10.5899	9.4370	8.5954	4.8564	0
GSM282472	0	1	1	0	2	2.2	12.58	0	10.3009	11.7535	4.9643	12.2561	10.4676	9.0462	8.8587	4.8191	0
GSM282473	0	1	1	0	2	3.3	13.33	0	10.2830	11.2185	5.5237	12.5011	10.8735	9.5424	9.9695	4.9381	0
GSM282474	0	0	0	0	3	4	11.92	0	8.8733	8.5184	4.1358	12.0588	10.5875	7.9443	8.9902	5.0398	0
GSM282475	1	0	0	0	2	1.5	12.17	0	12.2631	8.2478	3.4595	12.2722	10.5480	8.4659	8.8746	4.9802	0
GSM282476	0	1	0	0	2	2.3	10.83	0	8.9733	11.4513	3.5587	11.4258	10.1450	8.9061	8.3245	5.2247	0
GSM282477	1	0	0	0	2	3	11.58	0	12.9270	6.3797	4.1142	12.3162	10.1825	9.2908	8.6963	4.9270	0
GSM282478	0	0	1	0	2	2.5	1.25	1	9.3419	7.5557	4.8501	12.8419	10.7038	9.2259	8.3795	5.1083	1
GSM282479	0	1	1	0	1	5	11.42	1	9.9881	10.9914	5.6259	12.8090	10.4693	9.1045	9.4457	5.1481	0
GSM282480	0	1	1	0	2	2.2	16.75	0	9.1535	11.4430	6.1061	12.1424	10.6934	8.1617	8.7049	5.3871	0
GSM282481	0	1	0	0	2	1.7	13.17	0	10.5626	9.9248	3.1371	12.5017	11.4214	9.6156	9.0406	4.9864	1
GSM282482	0	0	0	0	2	2.9	9.67	1	9.1991	5.9800	3.9002	12.3072	10.5222	9.0450	8.8179	4.8633	0
GSM282483	1	0	0	0	3	4	0.08	0	12.6323	7.8393	3.8458	12.7464	10.0482	8.8522	8.3330	4.6935	0
GSM282484	0	1	1	0	3	2.5	1.42	0	9.0975	10.5703	4.8777	12.0845	10.6027	9.1869	9.0015	4.8593	0
GSM282485	0	1	1	0	2	1.7	3.92	0	10.0289	10.4998	6.4007	12.2999	10.1280	8.5903	8.3443	5.0850	0
GSM282486	0	1	0	0	2	1.8	14.17	0	8.9076	9.8855	4.7978	12.4415	10.5675	9.0432	9.0135	4.9281	0
GSM282487	0	1	1	0	2	2.1	17.08	0	10.1731	10.8404	5.6676	12.6563	10.4875	8.9638	9.4987	5.5662	0
GSM282488	0	1	1	0	2	3.7	1.33	0	9.2818	11.5016	5.1497	12.4868	10.5341	9.1617	8.7070	5.6885	1
GSM282489	1	0	0	0	2	2.5	13.33	0	12.3423	5.1721	3.7110	12.5985	10.5981	8.8743	8.0516	5.1178	0
GSM282490	0	1	1	0	2	2.1	5.83	0	10.1125	10.4021	7.6872	12.2485	10.6262	9.4407	8.9676	5.1632	0
GSM282491	0	1	0	0	2	2.2	2.50	1	9.0917	11.1834	4.0985	12.5290	10.2359	8.7488	8.3519	4.9309	0
GSM282492	0	1	1	0	2	2.2	8.67	1	9.9359	11.3573	6.0284	12.1357	10.1301	8.5988	8.8089	4.7213	0
GSM282493	0	0	0	0	2	2.3	15.92	0	8.2801	7.6361	4.0880	12.2782	9.2423	9.0653	8.6392	4.7615	0
GSM282494	0	1	1	0	3	0.1	15.92	0	10.6085	10.0563	4.8201	12.5015	11.3300	9.1653	9.0164	5.1444	1
GSM282495	1	1	0	0	3	3.6	20.00	0	11.2906	11.8841	4.7536	12.4670	9.8821	7.9815	8.6432	4.9954	0
GSM282496	0	0	1	0	2	1.5	5.42	0	9.9736	9.4218	5.2102	12.4250	10.1551	9.7062	8.8401	4.7915	0
GSM282497	0	0	0	0	3	3	7.75	0	9.2420	8.4778	3.9629	11.5836	9.6439	8.4288	9.0670	5.2685	0
GSM282498	0	1	0	0	2	2.2	16.08	0	9.7191	10.2228	3.1804	12.4252	10.9512	8.3804	8.7310	5.1117	1
GSM282499	0	0	0	0	2	2.1	15.75	0	9.8579	9.5188	4.2894	12.0822	10.2635	8.5899	9.8076	5.0472	0
GSM282500	0	0	1	0	2	2.5	16.00	0	8.8724	9.3790	6.3312	12.2508	9.8949	8.5473	8.0790	4.9012	0
GSM282501	1	1	0	0	1	0.1	8.17	1	11.2111	9.5625	3.8285	12.6592	11.2998	9.2905	8.5505	5.3612	1
GSM282502	0	0	0	0	2	2.2	16.92	0	10.1232	7.0752	4.3608	12.0742	10.1365	8.7766	8.5346	4.9261	0
GSM282503	0	1	0	0	1	2	15.00	0	9.6105	10.3438	3.7293	12.3515	10.1114	8.4585	8.3256	4.9916	0
GSM282504	0	1	1	0	2	2.5	13.58	0	10.5225	11.1573	6.6456	12.4107	10.3509	8.6730	9.7102	4.9573	0
GSM282505	0	1	0	0	1	2	14.75	0	10.5157	9.6553	3.8598	11.9459	9.7896	8.3626	8.5981	4.8024	0
GSM282506	0	1	1	0	1	1.6	2.50	0	9.9021	10.9829	5.4614	12.2825	10.4948	8.6910	9.2589	5.4100	0
GSM282507	1	1	1	0	2	2	3.75	0	12.1864	11.0214	4.8767	12.0734	9.6164	9.0602	8.5228	4.7685	0
GSM282508	0	0	0	0	2	2.4	10.83	0	8.5753	7.1496	4.3103	12.1189	10.1884	8.7907	8.4496	4.9233	0
GSM282509	0	1	0	0	2	2	6.58	0	10.4690	10.6260	3.5767	12.1752	10.2446	9.1991	9.4377	5.1027	0
GSM282510	1	1	0	0	2	2.4	0.58	1	11.9594	10.2044	3.9678	12.5443	10.2697	8.9416	8.7333	5.3208	0
GSM282511	0	0	0	0	3	2.3	11.17	0	9.2071	9.1263	4.1927	11.9879	9.7993	8.4234	8.8835	5.5808	1
GSM282512	1	1	0	0	2	1.8	2.75	0	12.2902	9.8438	4.6935	12.0714	9.8127	8.5421	8.7905	4.7082	0
GSM282513	0	1	0	0	2	1.7	8.58	0	10.1858	11.2320	4.0911	12.0725	9.9559	8.5516	8.3080	4.8084	0
GSM282514	0	1	0	0	2	0.5	13.83	0	10.5276	9.9006	4.3743	12.4345	10.2826	9.3099	9.5395	5.3495	0
GSM282515	0	1	1	0	2	2.3	2.08	0	9.5836	11.3552	6.8393	12.3244	10.1829	9.1215	9.4997	4.6119	0
GSM282516	0	1	1	0	2	0.9	8.42	0	9.3433	9.7988	4.8556	12.3160	9.6126	9.1378	8.7936	5.0648	0
GSM282517	0	0	0	0	1	2.3	11.67	1	9.8405	9.3560	4.2934	12.1418	10.1591	8.2343	8.7537	4.9231	0
GSM282518	1	0	0	0	2	1.7	10.67	0	12.6351	5.7670	3.4034	12.2490	9.7512	8.9671	8.2436	5.0793	0
GSM282519	0	1	0	0	3	2.5	11.00	0	9.1092	10.5860	4.0689	12.5037	10.3424	9.4446	9.8641	5.5696	1
GSM282520	0	1	1	0	2	1.7	1.83	1	9.5164	11.5524	6.2569	11.8899	10.4850	8.8578	9.3024	5.1969	0
GSM282521	0	0	0	0	1	3	12.50	0	10.0412	8.6814	3.7121	12.3060	10.7945	8.7702	8.4571	5.4103	0
GSM282522	0	1	0	0	2	2.4	3.75	1	10.3005	9.8273	3.6419	12.5329	10.5738	8.4305	8.6232	5.2889	0
GSM282523	0	1	0	0	1	2.2	12.92	0	9.3860	10.3040	4.3129	12.0335	10.4397	8.2552	8.7747	4.9027	0
GSM282524	0	0	1	0	2	2	11.92	0	10.3512	9.2213	5.7063	12.3992	10.2138	8.9056	9.0467	5.3068	0
GSM282525	0	1	0	0	3	3.5	0.50	1	9.2575	12.5453	4.0542	12.0470	10.2702	8.1791	9.1156	5.1240	0
GSM282526	0	1	0	0	2	1.2	6.42	0	8.1592	11.7705	4.5771	12.5748	10.0606	8.7154	8.5082	4.9770	0
GSM282527	0	1	0	0	3	2.3	6.83	0	9.7359	11.0909	3.6497	11.6880	9.7492	9.6328	8.4072	5.1046	0
GSM282528	0	0	0	0	2	1.7	11.08	0	8.8244	5.5763	3.8129	12.2131	9.8699	8.6730	8.4557	5.1148	0
GSM282529	0	0	0	0	1	1.3	7.25	0	9.9614	9.3612	3.9921	12.0381	10.0194	8.9878	8.7087	5.2606	0
GSM282530	0	1	1	0	2	1.7	3.42	0	10.2492	10.9274	6.5128	12.3282	10.4481	8.1797	8.2380	4.8029	0
GSM282531	0	1	1	0	2	1.3	12.33	0	9.6970	9.9622	5.9088	12.1444	9.7454	8.4523	8.6544	4.8213	0
GSM282532	0	0	1	0	2	1.6	6.25	0	9.0829	8.0705	5.1643	12.1314	9.8658	8.4996	8.6712	5.1320	0
GSM282533	0	0	1	0	2	6	9.17	1	10.0962	9.4097	4.8769	12.6916	10.6122	8.8232	9.2584	5.1751	0
GSM282534	0	1	0	0	1	2.4	10.42	0	8.8370	10.3021	3.9960	12.4089	10.0054	8.4243	8.6672	4.7886	0
GSM282535	0	0	0	0	3	1.5	3.08	1	7.4637	6.8895	3.4364	12.1419	10.0650	9.2148	8.6368	4.7421	0
GSM282536	0	1	0	0	2	0.8	16.50	0	9.7123	10.9179	4.0995	12.4913	10.0226	8.7746	8.3650	4.8812	0
GSM282537	0	1	0	0	2	2.5	11.08	0	9.6149	11.8804	4.0933	12.5758	10.4603	9.9785	9.1093	4.7593	0
GSM282538	0	1	0	0	2	1.7	1.50	1	10.4136	11.2504	3.6838	12.4486	11.0579	9.1744	9.1947	5.0711	1
GSM282539	0	1	1	0	2	1.8	9.75	0	9.4221	11.0600	6.6450	12.6567	11.1729	10.0533	9.3950	5.1905	1
GSM282540	0	1	1	0	1	1	7.83	0	9.5713	10.7092	5.3573	12.2704	10.2041	8.7168	8.8917	4.4090	0
GSM282541	0	1	0	0	1	1.5	8.08	0	10.1200	9.8850	4.4203	12.3456	10.3610	8.9937	9.3438	5.2280	0
GSM282542	0	1	0	0	2	2	10.50	0	10.1705	10.7042	4.2253	12.1034	9.5342	8.9128	9.2824	5.5160	0
GSM282543	0	1	1	0	2	2	6.00	0	9.6794	11.7854	7.7415	12.1483	10.2288	8.3689	8.8066	5.0771	0
GSM282544	0	0	0	0	2	3	4.67	1	10.3441	8.9708	3.5325	12.0634	9.2000	9.0445	8.8419	5.2127	0
GSM282545	0	1	0	0	2	1.5	11.75	0	9.2737	11.7786	4.1810	12.3982	10.6035	8.8332	9.1822	4.6511	0
GSM282546	0	1	0	0	1	2	6.00	0	10.1310	10.4868	3.9790	12.3406	9.3815	9.6115	8.8268	5.6208	1
GSM282547	0	0	0	0	1	3	9.33	0	10.2829	7.9301	3.3807	12.0602	9.2144	9.2880	8.2195	5.7251	1
GSM282548	0	1	0	0	2	4	7.75	0	9.1592	10.5189	3.6092	12.5151	10.9937	8.8429	9.8875	5.3846	1
GSM282549	0	1	1	0	2	2.5	8.33	1	10.1790	10.2080	5.8331	12.1659	9.3155	8.4893	9.0763	5.6419	1
GSM282550	0	0	0	0	2	2.3	7.58	0	10.6391	8.7828	4.7110	11.7002	9.5681	8.2774	8.2825	5.8122	1
GSM282551	0	0	0	0	2	1.5	0.67	1	9.4181	5.5487	3.1261	12.4292	10.7464	8.6020	9.0300	5.0142	0
GSM282552	0	1	0	0	2	3	5.00	0	9.6763	11.6510	3.6175	11.9495	10.4447	8.2852	9.3140	4.9705	0
GSM282553	1	1	0	0	3	1.6	6.33	1	11.7405	9.5539	3.8711	12.1516	9.0980	9.1883	8.2402	5.1854	0
GSM282554	0	1	1	0	2	2.5	0.08	0	9.4662	9.7231	6.7649	11.6208	9.6172	9.4501	9.9603	5.3074	0
GSM282555	1	0	0	0	1	1.8	5.08	1	11.1738	8.7161	3.6933	12.1583	9.7025	9.9864	8.3976	5.2389	0
GSM282556	0	1	0	0	2	2	11.92	1	9.4147	10.2133	4.7359	11.9681	9.5230	9.2895	8.6833	5.2123	0
GSM282557	0	1	1	0	2	1.2	5.92	1	9.8994	10.2094	6.5749	12.4775	10.6940	8.7424	9.3799	4.6239	0
GSM282558	0	1	1	0	2	0.6	5.42	0	10.6613	9.6995	5.5378	11.8642	10.7664	9.9428	9.0075	5.5168	0
GSM282559	0	0	1	0	2	2.2	5.08	0	10.1542	9.1675	5.3879	12.2643	9.9103	9.6395	8.5868	5.4237	0
GSM282560	0	1	1	0	2	1.4	5.42	0	9.9507	10.2592	5.3507	11.9576	9.1624	9.1150	8.1618	5.3285	0
GSM282561	1	1	0	0	2	5.5	1.00	0	12.3432	10.2639	3.5442	12.2822	9.7558	9.2308	8.8285	5.5360	0
GSM282562	0	0	0	0	2	1.5	3.75	0	10.0429	8.7233	4.7247	12.3236	10.5925	9.3340	8.6633	5.5733	1
GSM282563	0	1	0	0	2	1.6	4.50	0	10.1050	10.7315	3.9413	12.2933	9.6828	8.7472	8.6585	5.0621	0
GSM282564	0	0	0	0	1	1.4	6.33	0	9.8922	7.7221	4.7692	12.3555	9.7929	9.4372	8.2528	5.2790	0
GSM282565	0	0	0	0	3	3	6.50	1	9.6804	4.6562	3.7388	12.2158	10.6806	10.5927	9.1386	5.3868	0
GSM282566	0	0	1	0	2	1.8	3.50	1	10.5000	8.1019	6.7505	11.9332	9.6314	9.1050	8.7393	5.4010	0
GSM282567	0	1	0	0	2	2.5	1.17	1	10.0068	10.4161	3.7653	12.1955	9.8407	8.9101	8.8694	5.3497	0
GSM282568	0	1	1	0	2	3	3.83	1	9.6985	10.1879	4.8300	12.7718	10.9825	8.6894	8.6511	5.4992	1
GSM282569	0	0	0	0	3	3.6	0.83	1	8.8536	6.5620	3.9692	12.6024	9.3568	9.5989	8.8888	5.1405	0
GSM282570	0	1	0	0	2	2.3	2.67	1	10.1448	12.4721	3.8668	12.9046	10.8085	8.9744	9.1217	5.1137	1
GSM282571	0	0	1	0	2	1.1	5.17	0	10.5934	7.7348	5.2152	12.4559	9.8826	9.2670	9.1081	5.2993	0
GSM282572	0	1	1	0	2	2.1	0.25	1	9.7850	13.0319	6.5923	12.3946	10.9325	9.0085	9.3875	5.3119	0
GSM305129	0	1	0				5.00	0	9.7310	11.3014	4.7620	11.6652	8.8721	8.8882	8.1249	4.7769	0

GSM305130	0	1	1	5.72	0	10.6668	10.9539	5.3625	12.6311	9.9929	9.1442	9.1332	4.8222	0
GSM305131	0	1	0	5.28	0	8.9902	11.2800	4.3758	13.2634	10.1638	7.7578	8.5380	4.6733	1
GSM305132	1	1	0	5.11	0	11.3330	13.0819	4.8175	13.0010	11.1568	9.0207	8.8934	5.5219	1
GSM305133	0	1	1	5.58	0	10.9597	11.2084	5.4652	12.5455	10.5463	9.7376	9.0580	4.5345	0
GSM305134	0	1	0	5.26	0	10.5780	11.4612	4.2331	13.0133	10.9846	9.6212	9.0900	4.7556	1
GSM305135	0	1	1	5.05	1	10.8550	13.0704	6.7358	12.5517	10.7779	9.6201	9.5317	4.8400	0
GSM305136	0	1	1	5.29	0	10.6677	12.0534	6.4620	12.7386	10.7917	8.9466	9.1708	4.8410	0
GSM305137	0	1	1	5.35	0	10.7154	10.4480	5.7624	11.9986	10.8676	8.0304	8.6228	4.4567	0
GSM305138	0	1	0	5.15	0	9.8952	10.9191	4.3306	12.8139	11.5099	9.0387	9.1763	4.7115	1
GSM305139	0	1	1	5.00	0	10.9523	12.2663	5.1429	12.3937	11.0932	8.6249	8.8277	4.8989	1
GSM305140	0	1	0	3.72	1	10.0258	10.6354	4.7872	12.4094	9.8070	9.0416	8.6729	4.5348	0
GSM305141	0	1	1	5.28	0	9.5956	10.5738	6.1732	12.6315	10.4703	7.5014	8.2760	5.1373	0
GSM305142	1	1	0	5.68	0	11.4475	10.9247	4.4095	12.8557	12.2012	7.4045	9.1022	4.7284	1
GSM305143	0	1	1	5.66	0	10.2530	12.2085	5.2457	12.5120	9.9216	8.7793	8.4907	4.5645	0
GSM305144	0	1	0	6.75	0	9.1372	10.8733	3.7340	12.1809	9.9533	9.0979	8.4742	5.2853	0
GSM305145	0	1	1	6.90	0	10.0926	11.5988	5.4867	12.4004	10.7704	7.8596	8.9454	4.9846	0
GSM305146	0	1	1	5.49	0	10.3147	13.0814	4.9881	12.2139	11.1192	9.7992	8.4566	5.0499	1
GSM305147	0	1	1	6.58	0	10.2351	12.6194	6.7286	12.4938	10.8829	8.8114	9.1764	4.8252	0
GSM305148	0	1	1	5.13	0	9.8676	12.0607	5.3805	12.7470	10.6677	9.3044	8.8187	4.6510	0
GSM305149	0	1	0	7.02	0	10.0657	11.0571	4.2786	12.3972	10.8784	8.7610	9.0227	4.9564	0
GSM305150	1	1	1	9.16	0	11.4333	11.3341	6.8938	12.6907	10.4908	8.4171	9.0895	5.0864	0
GSM305151	0	1	1	5.41	1	10.4367	12.8365	6.1846	12.3131	9.8328	9.3176	8.9447	4.9035	0
GSM305152	0	1	1	5.60	0	9.1364	10.7903	5.7625	12.0913	10.3426	9.0453	8.8034	4.9882	0
GSM305153	1	1	1	1.91	1	11.7215	12.4470	5.6385	12.7791	9.3312	9.9205	7.9361	5.3226	0
GSM305154	0	1	1	10.38	0	7.6373	11.4388	6.3677	12.7096	10.9821	9.6242	9.0178	5.5991	1
GSM305155	0	1	0	10.24	0	9.9183	10.3467	4.0709	12.2740	9.9580	8.0582	8.9253	4.9326	0
GSM305156	0	1	1	10.12	0	10.8240	12.0005	4.9480	12.1030	9.1577	10.2733	9.1226	5.7803	1
GSM305157	0	1	0	0.63	1	10.1466	10.6194	4.6196	12.5953	9.7945	8.5173	9.0368	5.3372	0
GSM305158	0	1	0	9.97	0	9.8104	11.1913	4.4893	12.9633	11.4050	9.1015	8.4864	5.0383	1
GSM305159	0	1	0	9.86	0	9.9040	10.7605	3.9447	12.5247	10.0415	8.8890	8.5481	5.1238	0
GSM305160	0	1	1	5.18	0	9.0104	12.1985	6.2668	12.7270	10.8716	9.5378	8.5609	5.2034	0
GSM305161	0	1	0	9.71	0	10.5368	10.4270	4.2474	12.4545	10.0661	9.3125	8.7420	5.0920	0
GSM305162	1	1	1	5.16	0	12.1030	11.0536	5.6742	12.6377	10.2129	9.0915	8.6514	4.9318	0
GSM305163	0	1	1	8.24	0	11.0409	10.6038	6.6007	13.1249	8.8066	8.6213	9.2876	5.0522	1
GSM305164	1	1	1	3.00	1	11.7009	13.2197	5.3020	12.8699	10.6863	8.6660	8.6451	5.1967	1
GSM305165	0	1	0	6.72	0	9.8186	11.2755	4.4508	12.4661	10.6112	8.6744	9.3214	5.2754	0
GSM305166	0	0	0	8.43	0	9.1764	7.8442	4.7603	12.3471	10.9914	8.7105	8.4778	5.3625	1
GSM305167	0	1	1	6.43	0	10.7270	11.2847	5.9103	12.1372	10.4024	9.2536	8.9754	4.9577	0
GSM305168	0	1	0	5.35	0	11.0253	11.6108	4.5084	11.7040	9.4721	9.6831	8.7993	5.2180	0
GSM305169	1	1	1	9.71	0	11.7389	12.0217	5.5069	13.9272	9.2072	8.5931	8.9075	5.3690	1
GSM305170	0	1	1	9.18	0	10.8524	11.5502	5.3080	12.9249	9.2207	9.4759	8.9109	5.5921	1
GSM305171	0	1	1	9.15	0	8.1895	12.4464	4.8518	11.5869	10.5713	9.4673	8.5896	5.0928	0
GSM305172	0	1	0	6.33	1	10.3859	12.6097	4.4006	12.3962	11.1222	8.9645	8.8915	5.4521	1
GSM305173	1	1	0	7.90	0	12.0473	10.6026	4.7331	12.4549	10.4875	9.7762	8.8988	5.1019	0
GSM305174	0	1	1	7.60	0	10.8053	10.5013	5.1800	12.8488	9.8094	9.5183	8.4329	5.0193	1
GSM305175	0	1	1	6.95	0	10.2444	12.8400	4.8401	12.7888	10.6239	9.6282	8.4593	4.5346	0
GSM305176	0	1	1	7.26	0	10.1227	12.6698	5.2233	12.6734	10.7533	9.1897	8.5685	5.0405	0
GSM305177	0	1	0	7.13	0	9.1187	11.6431	4.5411	12.6889	9.9412	8.9562	8.4952	5.0217	0
GSM305178	0	1	0	9.47	0	9.7727	10.4390	4.4801	12.5366	10.1106	9.4165	9.2352	4.5836	0
GSM305179	0	1	0	9.07	0	10.7976	11.2876	4.3670	12.6240	10.8355	10.1401	9.4246	5.4049	0
GSM305180	0	1	1	2.45	1	9.7191	11.8870	6.7902	12.4935	10.1371	9.6990	8.9558	5.3454	0
GSM305181	1	0	1	8.59	0	12.1365	9.0445	4.8753	12.0483	10.6824	8.1320	9.5389	5.6952	1
GSM305182	0	1	1	8.53	0	8.0731	10.8924	5.6271	12.1248	9.5645	9.0450	8.9277	5.4369	0
GSM305183	0	1	0	7.74	0	10.8838	10.1985	4.2876	12.2626	10.1185	8.9460	8.6275	5.0817	0
GSM305184	0	1	0	8.68	0	10.4637	11.0115	4.1303	12.1732	10.3757	9.5521	9.1664	5.0968	0
GSM305185	0	1	1	8.21	0	10.1618	11.8665	7.0732	12.4249	10.4665	10.1738	8.7821	5.3389	0
GSM305186	0	1	0	11.58	0	9.5081	10.5162	4.7887	12.4264	10.4013	9.6621	9.0194	5.0195	0
GSM305187	1	1	1	10.69	0	12.9688	10.5826	5.1121	12.5693	10.5346	9.5164	8.7194	5.1507	0
GSM305188	0	0	1	9.10	0	10.2097	8.8129	4.9098	12.4622	10.0862	10.0648	9.4663	5.1834	0
GSM305189	0	1	1	9.51	0	9.7155	10.8023	6.5014	12.3833	9.9476	9.1952	8.8747	5.0364	0
GSM305190	0	1	1	12.83	0	10.1064	11.6071	5.0907	12.5029	10.4505	9.7338	9.1355	5.0350	0
GSM305191	0	0	0	12.41	0	10.1195	8.9254	4.5941	12.2588	9.9689	9.2752	8.9152	5.1123	0
GSM305192	0	1	1	2.43	1	8.9941	13.0354	4.9171	12.8492	10.9674	9.2362	9.0035	5.0751	1
GSM305193	0	0	0	6.52	0	8.5997	9.1980	4.4662	12.3924	9.8697	9.8161	8.8823	5.2591	0
GSM305194	0	1	1	8.68	1	9.9044	11.5880	5.4144	13.3074	10.7049	10.4652	9.0404	5.4370	1
GSM305195	0	1	0	5.21	0	9.7993	11.7852	4.4055	12.2851	9.8885	9.8406	9.0500	4.9696	0
GSM305196	0	1	1	6.35	0	9.5782	12.3348	4.9302	12.2361	9.9247	9.9429	8.7111	5.3743	0
GSM305197	0	1	1	16.05	0	9.5690	11.7984	8.0849	12.4036	10.6173	9.8690	9.8433	5.1255	0
GSM305198	0	1	0	9.05	1	9.1281	11.3948	4.7917	12.7585	10.0732	9.2200	8.7575	5.3353	0
GSM305199	0	1	0	15.99	0	9.7160	11.8852	4.4592	12.5512	10.2135	9.6535	8.7375	5.0334	0
GSM305200	0	1	0	11.63	1	9.3710	11.3766	4.6310	13.2533	11.1652	9.6033	9.1406	5.2612	1
GSM305201	0	1	1	5.18	0	9.2790	11.3184	5.4175	12.3853	10.3091	9.1325	9.0740	5.5133	0
GSM305202	0	1	0	9.00	0	10.5654	11.1702	4.6099	12.4333	10.5420	8.9173	9.0701	5.2258	0
GSM305203	0	0	1	13.69	1	9.6962	9.3862	5.4918	12.5189	9.8626	9.2785	8.6028	5.0197	0
GSM305204	0	1	0	12.58	0	8.9961	11.2740	4.5188	12.6560	10.9520	10.2096	9.0811	5.0743	1
GSM305205	0	1	0	8.15	0	9.4415	10.3793	4.3708	12.7404	10.0831	8.8489	9.7709	5.1828	0
GSM305206	0	0	1	5.94	0	9.4945	8.1614	6.9141	12.3483	10.1750	8.3794	8.5205	5.2655	0
GSM305207	0	1	1	3.95	1	9.6210	10.3121	7.1638	12.2578	10.8504	8.0274	9.2949	5.5722	1
GSM305208	0	1	1	10.81	0	9.8982	10.1153	5.6855	12.7341	9.6492	7.7310	8.9071	5.0839	0
GSM305209	1	1	0	9.68	0	11.3681	10.1987	4.6189	12.6802	9.9730	9.5118	9.1688	5.6460	1
GSM305210	0	1	1	10.87	0	10.4716	12.7990	5.4590	12.5687	9.7226	8.1092	8.8705	5.1391	0
GSM305211	0	1	1	5.77	1	10.4829	10.4522	5.6060	11.9926	10.1016	8.3868	9.6448	5.4923	0
GSM305212	0	1	1	15.94	0	10.0687	11.1786	6.6547	12.5405	10.5185	10.3893	8.6930	5.3352	0
GSM305213	0	1	0	5.01	0	9.7671	11.2342	4.6435	12.2140	10.3279	9.6844	9.2483	5.1467	0
GSM305214	0	0	0	5.10	0	9.3495	8.5480	4.1234	12.0274	10.8633	9.9767	8.6844	5.1721	0
GSM305215	0	1	1	14.78	0	9.6046	10.7611	5.3632	12.8102	11.3313	9.6662	9.4343	5.4581	1
GSM305216	0	1	1	3.18	1	10.3593	11.9979	5.0387	12.8775	10.7040	10.0052	8.6949	5.4797	1
GSM305217	0	1	1	2.58	1	10.1532	11.5495	5.7554	13.0369	8.9075	10.0680	8.8384	5.1098	1
GSM305218	0	1	1	5.16	0	9.2629	11.5610	5.4136	12.1737	10.2600	9.2838	8.6078	4.9763	0
GSM305219	0	1	1	7.65	0	10.0056	10.8627	5.1412	12.4948	10.4499	9.7265	8.7698	5.3694	0
GSM305220	0	1	1	13.40	0	9.6442	10.6994	6.0378	12.4784	10.3056	9.7121	9.0703	5.2711	0
GSM305221	0	1	0	11.03	0	9.6399	9.9612	3.6801	12.2581	9.9888	9.6737	8.6467	5.2470	0
GSM305222	0	1	0	13.57	0	10.2449	10.2921	4.4580	12.5444	11.0276	9.6527	9.3454	5.4787	1
GSM305223	0	1	1	11.80	0	10.6969	11.9685	4.8616	12.6634	11.0772	10.1878	8.3684	5.8822	1
GSM305224	0	1	1	11.30	0	10.2226	12.4012	4.9987	12.2935	10.3034	9.5862	9.5916	5.1462	0
GSM305225	0	1	0	15.18	0	10.0947	10.0131	4.5680	12.3211	11.6457	9.8525	9.7837	5.6724	1
GSM305226	0	1	0	14.69	0	10.8169	9.6088	4.1507	13.1233	10.2414	10.0842	8.6007	5.0550	1
GSM305227	0	1	1	8.80	0	9.9694	10.0253	4.8537	12.0793	9.7208	8.8902	8.8225	5.5455	0
GSM305228	0	1	0	9.40	0	10.9686	10.3617	4.2902	12.1261	9.7497	7.3889	8.9873	5.5529	0
GSM305229	0	0	0	8.06	0	9.7403	8.2366	4.6517	11.9168	10.1741	9.5169	9.2433	5.1600	0
GSM305230	0	1	1	8.61	0	10.2299	10.5828	6.4211	12.4304	10.1978	9.2396	9.4546	5.3633	0
GSM305231	0	1	0	8.34	0	10.2156	11.2078	4.7029	12.4460	10.0304	9.1036	8.4778	5.3891	0
GSM305232	0	1	1	5.26	0	9.4216	10.2347	5.2721	12.3508	10.1326	9.1337	8.9193	5.9870	1
GSM305233	1	0	0	9.69	0	11.6379	9.4983	4.4580	12.7875	10.1780	10.1469	9.7094	5.5479	0
GSM305234	0	1	1	8.39	0	10.5499	11.8986	6.0645	12.2776	10.5580	9.1919	9.1777	5.7316	1

GSM305235	0	0	1			8.67	0	10.1303	9.2271	5.1222	12.4755	10.0911	8.9385	8.9431	5.2901	0
GSM305236	0	0	0			1.10	1	9.3415	8.5640	4.5768	11.7633	10.9730	8.8396	8.7318	5.7305	1
GSM305237	0	1	0			13.63	0	9.9657	10.4520	4.4918	11.9495	9.7882	9.1695	8.9178	5.5510	0
GSM305238	1	1	0			6.88	0	11.2985	11.4050	4.5290	12.4290	10.5448	8.7018	8.7663	5.1400	0
GSM305239	0	1	1			5.07	0	9.4447	11.2589	7.5224	12.6905	10.3003	9.0935	8.7358	4.9401	0
GSM305240	0	1	0			7.47	0	10.6233	11.5187	4.3903	12.9219	10.4756	8.7939	9.0562	5.0193	1
GSM305241	0	1	1			6.95	0	10.5918	11.4335	5.2534	12.4786	10.4435	8.9255	8.2591	5.0712	0
GSM305242	0	1	1			7.78	0	10.1304	11.8628	5.5261	12.3204	10.2998	10.1270	8.5029	5.8643	1
GSM305243	0	1	0			1.25	1	11.0192	10.0323	4.1154	12.5523	10.2973	8.9039	8.1788	5.1545	0
GSM305244	0	1	0			5.22	0	10.0122	12.7453	4.7948	12.3841	9.5624	8.3847	7.8184	5.3117	0
GSM305245	0	1	1			6.96	0	10.5938	11.8431	6.6768	12.3806	11.0953	9.9752	8.6928	4.9459	1
GSM305246	0	1	0			6.99	0	10.1690	10.7614	4.5872	12.5633	10.6519	9.7857	8.6439	4.7863	0
GSM305247	0	1	1			6.88	0	9.9959	11.8057	5.8295	12.7082	10.4882	9.8549	9.1791	4.8798	0
GSM305248	0	1	1			7.52	0	9.4181	11.7261	5.4440	12.2241	10.4185	9.4984	8.5331	5.2382	0
GSM305249	0	1	0			6.62	0	10.1043	10.7213	4.4625	12.5795	9.8032	10.4496	8.7909	4.9334	0
GSM305250	0	1	1			5.16	0	10.0862	12.3604	5.0075	11.8380	10.2649	9.5015	9.2072	5.1015	0
GSM305251	0	1	1			6.21	0	9.0470	11.8922	6.5543	12.4567	10.4188	10.1167	8.5744	5.4784	0
GSM305252	0	1	1			5.26	0	10.4725	11.0719	4.8596	12.5324	10.8118	9.7840	9.2535	5.1488	0
GSM305253	0	1	0			7.02	0	10.6056	11.1918	4.5088	13.1332	10.9775	10.3388	9.0015	5.2257	1
GSM305254	0	1	1			7.55	0	9.4888	9.5734	6.9825	12.5900	9.2014	8.5403	9.0922	5.4837	0
GSM305255	0	1	1			5.64	0	9.5703	11.7460	5.1566	12.4401	10.8563	9.0026	9.1828	4.8256	0
GSM305256	0	1	0			6.31	0	10.1958	12.6861	4.4521	12.4773	10.7862	9.4294	9.0362	4.9727	0
GSM305257	0	1	1			6.76	0	9.7152	12.7683	6.2926	12.4611	9.2484	9.7776	8.3986	4.7114	0
GSM305258	0	1	1			7.35	0	9.6431	13.1327	4.8412	12.7666	10.2112	9.0009	8.2915	5.1141	0
GSM305259	0	1	1			7.38	0	8.9758	12.0966	5.4216	12.3540	10.4663	9.6288	8.9473	4.7761	0
GSM305260	0	1	1			7.03	0	9.8259	11.5696	7.3920	12.5812	10.8937	9.6013	8.9107	5.3742	0
GSM305261	0	1	1			6.97	0	9.2868	11.1895	5.5519	12.5545	8.9309	9.7157	8.5671	4.7590	0
GSM305262	0	0	0			8.89	0	9.3431	7.0391	3.9371	12.8542	9.3543	8.8443	8.9781	5.9282	1
GSM305263	0	0	0			2.47	1	9.7027	6.1963	2.8446	13.4719	9.0791	8.3179	8.4995	5.9274	1
GSM305264	0	1	0			14.97	0	9.1044	10.1432	4.7230	11.7188	8.8793	8.2684	8.7930	5.3427	0
GSM308256	1	0	0	0.25	1			13.0689	4.8419	2.7677	12.3331	9.9986	8.6779	8.7441	4.9610	0
GSM308257	0	1	0	2.08	1			9.5862	10.6425	3.6959	12.3874	10.4337	9.4313	8.5286	4.8367	0
GSM308258	0	1	0	1.92	1			9.2263	10.4090	2.9480	11.4822	9.6527	9.7384	8.2522	5.1925	0
GSM308259	1	0	0	1.08	1			11.2623	5.7415	3.4101	12.0376	10.7236	8.9436	8.0733	5.1218	0
GSM308260	0	1	1	1.67	1			8.6892	10.9149	6.0273	12.0893	9.8782	9.5341	8.1660	5.1551	0
GSM308261	0	0	0	1.50	1			8.6980	5.7915	3.5980	12.0995	9.1866	8.5098	9.0924	5.5175	0
GSM308262	0	1	0	2.50	1			8.3241	11.6896	3.7062	11.5279	10.1079	9.1071	8.8082	4.8266	0
GSM308263	0	0	0	5.75	1			10.1214	9.2632	3.4851	12.9130	10.7358	9.1085	8.7341	5.0026	1
GSM308264	0	1	1	3.08	1			7.6648	12.7201	5.2919	12.6005	10.7560	9.6584	10.1904	5.1272	0
GSM308265	1	0	0	1.92	1			12.5659	7.3353	3.2094	11.7716	10.3550	9.5651	8.3059	5.1246	0
GSM308266	0	1	0	0.25	1			8.9650	10.0594	4.2448	12.1272	10.0063	9.9072	9.3415	4.8626	0
GSM308267	1	0	0	1.42	1			12.4544	7.6367	3.9648	12.0099	10.0006	9.8238	8.4431	4.8366	0
GSM308268	1	1	0	3.17	1			12.9840	12.5659	3.7412	12.2684	10.4404	9.3079	8.7683	4.7816	0
GSM308269	1	0	0	0.67	1			13.1375	5.9786	2.7506	11.9832	10.2307	9.0638	9.8165	5.0894	0
GSM308270	0	1	0	0.58	1			9.4123	13.6637	3.4463	12.3642	10.7579	9.5016	8.5235	5.3681	0
GSM308271	0	0	0	2.42	1			7.8183	6.0266	3.5655	11.7816	10.1880	9.3176	8.3556	4.8133	0
GSM308272	1	0	0	1.17	1			12.5064	9.3345	4.2781	12.4748	9.7564	9.4598	8.3895	4.8623	0
GSM308273	0	0	1	1.58	1			7.8958	8.5634	4.9887	12.4660	9.5911	9.6434	9.2225	5.3845	0
GSM308274	0	0	0	2.42	1			7.9712	8.2899	4.7475	12.0867	10.3220	9.8607	9.4282	5.0591	0
GSM308275	0	0	0	0.58	1			8.7560	9.1688	4.2796	12.1520	10.4570	8.8418	8.7546	5.2631	0
GSM308276	1	0	0	1.33	1			12.6536	7.6860	4.1169	12.7428	10.6209	9.5970	8.8182	5.0445	0
GSM308277	0	1	1	1.83	1			9.7330	12.2925	5.4615	11.9176	9.6085	9.1138	8.1979	5.9290	1
GSM308278	1	0	1	1.67	1			12.3055	8.6697	4.8857	12.5979	9.6148	9.6307	9.5269	5.0767	0
GSM308279	1	0	0	1.67	1			12.3252	8.7828	4.6773	12.5553	9.8377	9.6022	7.3760	4.9419	0
GSM308280	0	0	0	1.33	1			8.2715	7.8357	4.6720	12.5747	10.5942	9.1529	8.3117	5.1409	0
GSM308281	0	0	0	0.42	1			8.4864	6.8740	4.1894	12.6832	9.8514	9.2674	9.7330	4.9058	0
GSM308282	0	0	0	0.83	1			10.2135	5.9649	3.5685	12.0785	10.0539	9.9050	8.9676	5.0938	0
GSM308283	0	0	0	1.08	1			8.9063	8.6454	3.9969	12.2716	10.2392	9.2989	9.3282	4.7434	0
GSM308284	1	0	0	3.83	1			13.3624	7.6050	3.8683	12.2151	10.2639	8.5929	8.4061	4.6931	0
GSM308285	0	0	0	0.58	1			9.3431	4.8677	4.0556	12.6805	10.4550	9.3840	9.4719	4.9628	0
GSM308286	1	0	0	0.92	1			13.1176	8.9546	4.4085	12.5970	9.7597	9.2036	9.0429	5.3648	0
GSM308287	0	1	0	3.50	1			9.0876	10.1278	4.2567	12.2656	10.1490	8.8082	8.3886	5.2531	0
GSM308288	0	1	0	0.83	1			9.1819	10.6872	3.7664	12.0262	10.1476	8.7038	8.3386	5.2522	0
GSM308289	0	1	0	5.08	1			8.5414	12.1749	4.0363	12.0860	10.3535	8.6442	9.4043	4.7067	0
GSM308290	1	0	0	0.50	1			12.2791	8.1586	3.2221	12.3062	10.3896	9.7990	8.2080	4.9836	0
GSM308291	0	0	0	2.08	1			7.8516	7.9861	4.6254	12.5721	10.0780	8.8674	8.6600	4.9118	0
GSM308292	0	1	1	8.25	1			10.2320	12.1015	5.2210	12.7712	10.4861	9.3428	8.8513	5.2998	0
GSM308293	0	1	1	0.42	1			9.1812	12.0698	6.0635	12.6273	10.0735	9.0479	8.8606	5.1680	0
GSM308294	1	1	0	1.75	1			12.7581	11.8570	3.6544	12.6628	10.7644	9.7473	8.9229	5.0525	0
GSM308295	0	0	0	0.92	1			9.0148	4.4521	3.4667	12.0610	10.7671	9.2814	9.8891	5.2978	0
GSM308296	0	1	0	2.67	1			9.3826	13.7010	4.2040	12.4788	10.2300	9.4911	9.2422	4.8826	0
GSM308297	0	1	0	1.17	1			9.0705	12.3063	3.7197	12.8932	11.0132	9.2100	9.0815	4.8970	1
GSM308298	1	1	0	1.17	1			11.9500	13.4822	4.2632	12.6995	10.2107	9.6498	8.9913	5.0994	0
GSM308299	1	0	0	1.50	1			12.1857	9.1727	4.0863	12.7290	10.3465	9.6092	9.1541	4.6650	0
GSM308300	0	1	0	2.42	1			9.2871	12.7216	3.4275	12.1837	10.1832	9.9629	8.5569	4.8950	0
GSM308301	0	0	0	1.25	1			8.2806	7.5891	4.1376	12.5933	10.8846	10.1562	9.2193	5.4028	0
GSM308302	0	1	0	2.00	1			10.0266	10.2276	3.0410	12.0935	10.3194	8.6277	8.6024	5.0955	0
GSM308303	0	1	1	3.25	1			10.7502	11.5603	6.2660	12.2258	10.5008	9.2795	8.6130	4.5716	0
GSM308304	0	0	1	4.50	1			9.6181	9.1782	4.8876	12.0581	10.5476	8.4809	8.9763	5.0102	0
GSM308305	1	1	0	1.25	1			12.5517	13.4918	4.3408	12.7493	10.5075	9.7038	8.8681	5.7079	1
GSM308306	1	0	0	2.33	1			13.2520	7.8035	3.4975	12.5814	10.2941	9.6895	8.7789	4.7793	0
GSM308307	0	1	0	2.42	1			9.5522	10.9171	4.4545	13.0562	10.7291	10.4024	9.7738	5.1641	1
GSM308308	1	0	1	1.92	1			12.7572	8.5820	4.9669	12.6852	10.3388	9.8477	8.8131	4.5763	0
GSM308309	0	0	0	1.67	1			8.4793	8.5284	4.1166	12.6061	10.8831	9.6431	10.0627	5.2772	0
GSM308310	0	1	1	3.00	1			7.8029	13.2061	5.8092	12.6471	10.0317	9.1812	9.0759	5.0558	0
GSM308311	0	0	0	3.00	1			8.7996	7.1904	4.5961	12.3062	10.6145	9.5632	10.0983	5.0468	0
GSM308312	0	0	0	9.58	1			9.7766	7.9477	4.7406	12.5619	10.9608	8.8778	9.4204	4.5573	1
GSM308313	0	0	0	0.33	1			8.0626	5.9138	4.0372	12.9759	10.7328	9.3079	9.7560	4.6073	1
GSM308314	0	0	0	0.67	1			8.5559	7.3692	4.4658	12.6441	10.5586	9.0629	8.8757	5.5605	0
GSM308315	0	1	1	5.00	1			9.9696	13.7050	6.7712	12.7149	9.7356	10.3991	9.3852	4.5520	0
GSM308316	0	0	0	0.00	1			9.5334	6.6170	3.7479	13.1330	10.9660	9.9466	9.3007	4.9550	1
GSM308318	1	1	1	3.50	1			13.0271	9.9659	6.1039	12.7866	10.6494	8.9929	8.7654	5.9695	1
GSM308319	0	0	0	1.25	1			8.0764	7.1012	3.8628	12.6393	10.1798	9.4124	8.9135	5.4149	0
GSM308320	1	0	0	0.58	1			13.6070	7.5504	3.7902	12.6435	10.3675	9.7859	8.8314	5.8924	1
GSM308321	0	1	0	0.00	1			8.6024	12.7372	4.4368	12.9850	10.4173	9.9427	9.1914	5.7061	1
GSM308322	0	1	0	2.25	1			10.0835	13.7606	4.3530	12.5809	10.8101	9.3337	9.3000	5.4442	0
GSM308323	1	0	0	1.50	1			13.4552	7.0561	3.6163	12.4824	10.9156	8.9634	8.4028	6.0840	1
GSM308324	0	0	0	1.17	1			7.9598	6.4350	4.2979	11.8024	10.3661	8.7849	9.2702	6.2112	1
GSM308325	0	1	0	1.08	1			10.2642	13.9622	3.7808	12.6262	10.0383	8.8300	9.0141	5.0290	0
GSM308326	0	1	0	1.58	1			10.7422	12.3854	4.4086	12.1010	10.1055	9.5909	8.9157	4.8053	0
GSM308327	0	1	0	6.08	1			9.5150	12.7034	3.6564	12.1822	10.8093	8.9717	9.0736	6.2475	1
GSM308328	0	0	0	1.33	1			8.1810	5.1663	3.8654	12.6216	10.1246	9.4456	9.3360	5.2927	0
GSM308329	1	1	0	0.08	1			11.4723	11.5950	4.4580	12.9395	11.1541	9.6871	10.3363	6.6434	1
GSM308330	0	0	0	0.00	1			10.0017	6.2983	4.0053	12.6757	10.5038	9.0474	9.1848	5.2775	0
GSM308331	0	1	1	0.00	1			9.2259	11.5850	5.5300	12.6110	10.5871	9.4647	8.9371	5.1395	0

GSM308332	0	1	0	3.17	1	10.1122	11.3193	3.3416	12.6801	10.1489	9.8723	8.7525	4.6512	0
GSM308333	0	0	0	1.50	1	9.1064	7.7217	4.6002	12.4185	10.3466	8.8202	9.2651	5.0218	0
GSM308334	1	1	0	1.92	1	12.6038	11.1265	3.8999	12.5776	10.1667	9.5332	8.8640	4.4628	0
GSM308335	0	0	0	4.17	1	8.7875	7.6468	4.1710	12.0635	9.4970	9.0833	9.3285	4.2115	0
GSM308336	0	0	0	0.67	1	9.0485	7.5461	4.1323	12.1307	10.2870	9.1809	9.5644	4.9723	0
GSM308337	0	1	1	3.25	1	10.0108	13.1156	5.7619	13.0381	10.1792	9.8059	8.8816	4.6299	1
GSM308338	0	0	0	0.83	1	7.3960	7.9798	4.2534	12.4435	10.2534	10.0842	9.9076	4.7577	0
GSM308339	0	0	0	0.25	1	7.3194	4.8224	3.5556	13.0922	10.3905	9.5838	8.9638	5.2103	1
GSM308340	0	0	0	5.00	1	9.3100	7.2632	4.2033	13.2978	10.5198	10.6024	9.3759	4.8929	1
GSM308341	0	1	0	1.42	1	8.8043	9.6142	4.1654	12.2874	9.6631	8.2252	9.1944	5.3291	0
GSM308342	1	0	0	1.92	1	13.3469	7.2385	3.7108	12.8915	10.2756	8.7543	8.9471	4.5760	1
GSM308343	1	0	0	1.00	1	13.3609	9.3248	4.3306	12.8826	10.6593	9.5516	9.4688	5.1217	1
GSM308344	0	0	0	0.50	1	9.6050	7.4945	3.9538	12.6168	10.4734	10.3161	9.9289	5.9781	1
GSM308345	1	1	0	2.50	1	12.8221	11.1656	4.6045	12.4480	10.1202	9.7323	8.7864	5.6664	1
GSM308346	0	0	0	1.08	1	8.3719	6.9892	4.0712	12.1996	9.6878	9.7122	9.1081	5.1239	0
GSM308347	0	1	0	0.50	1	9.4661	14.6760	4.0988	12.5692	10.4318	9.3831	9.1578	5.6884	1
GSM308348	0	0	0	2.08	1	9.1028	7.1344	3.9596	13.0264	10.7945	9.6794	9.5591	6.0943	1
GSM308349	0	0	0	0.33	1	8.7756	8.2182	3.8969	12.4781	9.9932	9.1538	9.0678	5.3806	0
GSM308350	0	1	0	1.25	1	8.2194	11.4921	4.6642	12.1680	10.1762	8.3379	9.7498	6.5695	1
GSM308351	0	1	1	2.50	1	9.2167	13.0165	7.7828	12.5301	10.6977	9.5066	9.3178	4.9099	0
GSM308352	0	0	0	0.00	1	8.2313	9.1777	4.0851	12.5378	10.6174	9.2101	8.9873	5.6000	1
GSM308353	0	1	1	5.33	1	9.4001	12.3646	7.7366	12.5413	10.5310	8.6745	9.3987	5.6578	1
GSM308354	0	0	0	1.75	1	9.2364	8.8250	4.3410	12.6673	10.3727	9.5027	9.3590	6.7378	1
GSM308355	1	1	1	0.00	1	12.6607	13.8500	5.9817	12.1213	10.2041	8.5722	8.7135	4.9807	0
GSM308356	0	0	0	1.50	1	8.7585	5.8105	3.5681	12.9001	11.0098	10.0560	9.3787	5.4825	1
GSM308357	0	0	0	1.33	1	8.6000	7.7398	4.2801	12.7562	10.8476	9.2586	9.3028	5.9992	1
GSM308358	0	1	1	0.00	1	9.1611	12.5482	5.0288	12.6365	10.7026	8.6507	9.5082	5.5145	0
GSM308359	0	1	0	3.00	1	8.7446	13.9059	3.9616	12.7415	10.2066	9.3292	8.9602	4.7445	0
GSM308360	0	1	1	3.25	1	10.7921	12.5680	6.3176	12.9128	11.0387	8.8857	9.4395	6.0117	1
GSM308361	0	1	1	2.25	1	8.5138	12.1962	5.1034	12.4923	9.8373	9.1694	9.6961	6.8042	1
GSM308362	1	1	0	1.25	1	13.1813	9.7142	3.7838	12.6122	10.5026	9.3335	8.9281	5.6532	1
GSM308363	0	1	0	1.83	1	8.5634	12.1837	3.8121	12.1143	10.0666	8.6181	8.4955	5.2550	0
GSM308364	0	0	0	2.42	1	5.5435	7.2520	3.4218	12.5647	10.1793	10.0968	8.2087	5.1376	0
GSM308365	0	0	0	0.92	1	9.2865	7.3771	4.1054	12.3722	10.4646	9.6374	9.4417	5.9503	1
GSM308366	0	0	0	4.83	1	7.6142	7.7036	4.0719	12.2675	9.9422	9.5148	8.0989	4.4031	0
GSM308367	0	1	1	3.33	1	8.7847	15.2493	6.6287	12.2932	10.2113	9.1335	7.9932	5.6240	1
GSM308368	0	1	0	4.00	1	8.9949	11.8954	4.4007	12.6203	10.6241	9.3213	9.9723	5.6088	1
GSM308369	0	1	1	3.17	1	9.1456	12.7458	7.4082	12.8616	10.3037	9.3615	8.5516	5.4688	1
GSM308370	1	0	0	1.00	1	12.9181	7.7740	3.9968	12.5884	10.5771	9.0095	8.2107	6.6837	1
GSM308371	0	1	0	1.08	1	10.6976	11.8745	3.9068	12.8980	10.3427	9.5471	8.2534	5.0563	1
GSM308372	0	1	0	7.08	1	8.9233	12.4343	4.4168	12.6862	10.9367	8.7981	9.1684	6.2742	1
GSM308373	1	1	1	0.75	1	13.1257	10.7199	6.5992	12.3828	10.2316	9.4873	8.9709	5.1041	0
GSM308374	0	0	0	0.50	1	9.7242	6.5648	3.3917	12.7790	10.5460	9.2736	9.5346	5.1263	0
GSM308375	0	1	1	2.33	1	10.4361	13.6884	5.0949	12.6890	10.5270	8.8437	9.5788	5.5398	0
GSM308376	0	0	0	0.50	1	8.5906	6.7249	4.3020	12.5842	10.5688	9.3341	8.9060	5.2374	0
GSM308377	0	1	0	1.58	1	8.8452	9.6951	4.2260	12.3023	10.0077	8.6859	8.9306	6.4883	1
GSM308378	0	0	0	1.25	1	8.3341	9.0735	4.1168	12.6118	10.2948	9.0235	9.8007	5.3808	0
GSM308379	0	1	0	1.58	1	10.2371	12.9310	4.3979	12.9537	11.1528	10.2048	9.3703	4.9012	1
GSM308380	0	0	0	0.58	1	9.3569	6.7733	4.1809	12.3090	10.4876	9.3975	8.6002	5.3152	0
GSM308381	0	1	1	9.58	1	9.1942	10.5400	5.5629	12.1980	10.2284	9.5606	8.9006	5.3245	0
GSM308382	0	1	0	3.83	1	10.0192	9.8277	2.9951	12.2619	10.3863	9.6730	8.7156	5.2507	0
GSM308383	1	1	1	0.75	1	13.0107	10.2144	6.6801	12.2657	10.8560	10.1976	8.8190	5.0530	0
GSM308384	0	1	0	2.67	1	10.3712	9.9617	3.3092	12.1881	10.1579	9.2460	8.3678	5.4857	0
GSM308385	0	1	1	2.25	1	9.4153	11.4963	5.4131	11.8891	10.3889	9.6883	8.6310	4.8603	0
GSM308386	1	0	0	0.42	1	12.8138	6.0006	3.8314	12.4396	10.5128	9.5912	8.9118	5.1237	0
GSM308387	0	0	0	0.33	1	8.5903	6.1428	4.1443	11.9029	9.8623	9.8386	8.4888	5.1609	0
GSM308388	0	0	0	1.42	1	8.1307	5.5817	3.3015	11.8386	9.2227	9.6956	8.3118	4.7924	0
GSM308389	0	1	0	1.25	1	10.1515	10.3314	4.5835	11.9508	10.6484	10.4347	8.1495	5.2238	0
GSM308390	0	1	1	5.08	1	8.3871	10.8031	7.0682	11.9617	10.3539	9.5610	8.6779	5.3533	0
GSM308391	0	0	0	0.58	1	8.0033	9.0924	4.2404	12.3544	10.4063	8.2059	9.1145	5.0279	0
GSM308392	0	0	0	0.50	1	8.0507	6.5466	3.7207	12.3176	10.8138	8.7981	8.6546	4.7719	0
GSM308393	0	0	0	2.75	1	9.4209	7.4337	4.5571	12.8631	10.3216	9.2136	10.1356	5.4399	1
GSM308394	0	1	0	1.83	1	8.6915	10.2832	4.7435	12.7450	10.1552	9.5455	9.8149	6.3712	1
GSM308395	0	1	1	2.83	1	9.6244	11.7469	6.6037	12.9299	10.9276	9.4647	9.1230	4.7271	1
GSM308396	1	1	1	1.17	1	12.6445	12.2357	5.7803	12.8577	10.0350	9.7448	9.1908	4.8992	1
GSM308397	0	1	0	3.58	1	8.6573	12.1114	4.4814	12.5419	10.2885	9.7490	9.4385	4.8519	0
GSM308398	0	1	0	1.00	1	9.9268	9.5735	4.0978	12.3752	10.0562	9.6097	9.4235	5.1452	0
GSM308399	0	1	0	3.17	1	8.7179	11.9748	3.4978	12.3317	10.4650	9.7751	9.8432	4.6219	0
GSM308400	0	0	0	0.92	1	8.8860	7.6025	4.0074	12.2352	10.4955	9.6071	9.0996	6.4338	1
GSM308401	1	1	0	1.08	1	12.7402	10.9019	3.9595	12.9289	10.5838	9.1841	8.6464	5.3323	1
GSM308402	1	0	0	1.00	1	13.1929	7.8123	3.9529	12.3352	9.8809	9.7454	9.0088	5.0403	0
GSM308403	0	1	0	0.92	1	9.0385	12.4312	3.9745	12.5906	10.4334	9.1646	8.8822	5.1958	0
GSM308404	0	0	0	0.67	1	8.1368	5.6332	3.9517	12.5934	10.8650	9.5730	8.6705	5.0890	0
GSM308405	1	0	0	2.00	1	12.2177	8.2424	4.2038	12.6848	10.4302	9.3568	9.5620	6.0018	1
GSM308406	0	1	0	4.83	1	10.5307	12.7703	4.8040	12.3296	10.6403	9.6578	8.9042	5.0243	0
GSM308407	0	1	0	7.83	1	8.2285	10.8090	4.0533	11.9855	10.0386	9.3977	9.8339	5.5807	1
GSM308408	0	0	0	0.33	1	8.4610	6.1119	4.0113	12.5692	10.5087	9.6216	9.3958	4.2476	0
GSM308409	0	1	0	0.00	1	10.2625	12.3756	3.2208	12.9972	11.0500	9.5949	8.9115	5.0917	1
GSM308410	0	1	1	3.17	1	9.8240	11.9877	7.9801	12.3896	9.9168	9.4471	9.4587	5.0702	0
GSM308411	0	1	0	0.92	1	8.8011	9.6701	3.3805	12.1832	11.0102	9.3193	9.0788	4.9943	1
GSM308412	1	0	0	4.00	1	12.8331	8.7797	3.6732	11.9220	9.7058	8.8817	7.5071	4.9219	0
GSM308413	0	1	1	4.08	1	10.2955	14.6595	6.4602	11.6945	10.3142	10.7158	9.1477	6.5859	1
GSM308414	0	1	1	8.08	1	9.3872	13.0514	5.8765	12.4076	10.2384	9.8813	9.2237	6.4028	1
GSM308415	0	1	0	4.17	1	9.9583	13.5400	4.5587	12.0980	10.4143	9.3653	9.2562	5.3054	0
GSM308416	0	1	1	4.42	1	9.8208	13.1656	7.5149	12.4958	10.5639	9.9753	8.5416	4.6993	0
GSM308417	1	1	1	1.92	1	13.0130	15.1836	8.3624	12.5790	10.4846	9.7514	9.3702	5.7139	1
GSM308418	0	1	0	2.08	1	9.0413	13.2626	3.6406	12.7245	10.2585	10.5731	8.2116	5.2581	0
GSM308419	1	1	0	1.83	1	11.7039	13.9750	4.3239	12.4265	9.8695	9.3739	9.2759	5.1632	0
GSM308420	0	1	1	3.00	1	9.4756	12.4536	6.7487	12.9664	10.5155	10.2656	9.5418	5.1696	1
GSM308421	1	1	0	2.92	1	11.3052	12.6273	3.0795	12.7755	10.8092	10.3341	9.1258	7.1994	1
GSM308422	0	1	0	1.33	1	10.3273	12.8477	4.2320	12.5972	10.9133	9.9176	9.1927	5.3612	0
GSM308423	0	1	0	2.92	1	9.7674	14.6378	4.0533	12.5030	10.6866	8.9210	8.7889	5.7782	1
GSM308424	0	1	0	1.92	1	10.3533	13.8751	4.5017	12.8237	11.4524	10.1061	9.1451	5.5385	1
GSM308425	1	1	0	1.17	1	11.5009	13.3350	4.0687	12.5473	10.1303	9.1135	9.1129	6.3824	1
GSM308426	0	1	1	0.75	1	11.0607	14.7454	4.8204	12.5396	10.4070	9.8062	8.9727	5.5143	0
GSM308427	0	1	0	3.50	1	9.5798	14.1687	3.9516	12.1389	10.7052	9.4043	8.2059	6.0960	1
GSM308428	0	1	1	1.42	1	10.0986	13.8713	5.0068	12.6092	10.2751	9.7989	8.8880	5.7608	1
GSM308429	0	1	0	0.42	1	9.8824	12.8608	3.3247	12.6028	11.1212	9.4740	9.5925	5.2368	1
GSM308430	0	1	0	4.58	1	8.9400	10.8156	4.4639	12.4217	10.1718	10.3531	8.9724	5.6292	1
GSM308431	0	1	0	1.92	1	9.6048	10.5055	3.5945	12.4836	10.8070	10.0359	8.8731	4.7142	0
GSM308432	0	1	1	3.33	1	9.4195	13.4886	8.6140	12.1635	10.1438	9.4579	9.5615	5.3664	0
GSM308433	1	1	1	3.67	1	13.0534	11.8427	5.4403	12.4338	10.2225	9.7871	8.0443	5.3286	0
GSM308434	1	1	1	3.92	1	11.8085	11.5741	8.5727	13.0459	10.8736	10.6382	9.0153	5.7662	1
GSM308435	0	1	1	2.17	1	8.7603	12.0469	7.6139	12.2764	9.3236	8.4050	9.1311	5.0099	0
GSM308436	0	1	1	1.75	1	10.0083	13.3341	9.2183	12.0195	9.5863	9.3188	8.8950	6.0941	1

GSM308437		0	1	1		2.92	1	9.5838	14.0097	7.4741	12.9058	10.0523	10.6832	9.1155	5.3059	1
GSM308438		0	0	0		2.92	1	9.9403	9.4531	3.8064	12.8070	10.5114	10.4769	8.7342	5.7267	1
GSM308439		0	1	1		0.33	1	8.2725	12.8755	8.2595	12.3370	10.1611	9.5098	9.0858	6.4875	1
GSM308440		1	1	1		0.42	1	11.8791	12.0609	5.2607	12.9711	10.5738	9.5672	9.3074	7.3035	1
GSM308441		0	1	1		3.50	1	9.1330	10.6142	5.5051	12.6503	10.1308	9.5954	9.4524	6.0181	1
GSM308442		0	1	1		1.50	1	9.9318	12.1726	5.5388	12.4909	10.5388	9.9889	9.1695	5.5703	1
GSM308443		0	0	0		1.17	1	7.6121	8.3624	4.2152	12.4687	10.1296	9.4825	9.1073	5.9119	1
GSM308444		0	1	0		1.33	1	8.9626	13.6328	3.8998	12.0148	9.6163	9.5715	8.5413	5.3472	0
GSM308445		0	1	1		2.50	1	10.3522	12.7153	6.6796	12.3094	10.3037	9.7443	9.6106	5.4097	0
GSM308446		0	1	0		3.50	1	10.4989	12.5663	3.5311	11.7791	9.4601	8.8532	8.7561	6.4845	1
GSM308447		0	1	1		2.00	1	8.7962	12.3626	5.0903	12.5746	9.9746	9.3157	9.2820	6.2351	1
GSM308448		0	1	0		2.08	1	10.2316	13.9908	4.1838	12.3831	9.7279	9.3839	8.6475	6.1928	1
GSM308449		0	0	0		3.92	1	9.8512	9.2431	4.4675	12.1496	10.1630	9.3251	8.5842	5.8675	1
GSM308450		0	1	0		2.75	1	9.3985	14.1062	4.3752	12.4484	9.8582	9.8449	9.1567	7.0520	1
GSM308451		0	1	1		2.42	1	9.8673	14.1798	9.1158	12.4668	10.4307	9.2894	9.8261	6.2413	1
GSM308452		0	1	1		2.08	1	9.6859	14.6935	8.5151	12.8941	10.3399	10.2410	9.3801	6.2559	1
GSM308453		0	1	1		6.92	1	10.2514	14.7181	9.1253	12.1723	10.6657	9.6257	8.7385	6.9888	1
GSM308454		0	1	1		6.08	1	9.8399	14.0863	7.8437	12.4767	10.8805	9.9768	9.1607	6.4960	1
GSM308455		0	1	0		2.58	1	10.3114	13.1646	3.9159	12.2984	9.9626	9.1420	8.8129	5.4675	0
GSM308456		0	1	0		2.33	1	9.5560	12.3205	3.8900	12.6897	11.2633	9.9366	8.8221	6.4613	1
GSM308457		1	1	0		1.50	1	11.2296	12.1947	3.0340	13.3256	11.3911	9.8456	8.7642	6.3919	1
GSM308458		0	1	1		0.75	1	9.9306	14.0502	6.3248	13.0768	10.5508	9.3889	9.0807	4.3976	1
GSM308459		0	1	0		0.58	1	9.6558	13.6191	3.6444	12.7532	10.2101	9.3441	9.4145	4.5285	0
GSM308460		0	1	1		8.92	1	9.4335	12.3424	6.2547	11.9805	9.9968	9.2538	9.0637	4.6026	0
GSM36777	1	0	1	1	0	6.58	0	9.7881	11.5668	5.0829	12.3505	10.1512	9.1913	9.0405	5.4661	0
GSM36778	1	0	1	0	0	4.17	1	10.2105	9.8801	4.2656	12.8125	9.7670	9.6026	9.0249	6.1009	1
GSM36779	1	0	1	1	0	11.00	0	9.8545	11.0922	5.0012	12.0557	10.8916	8.9318	8.6736	5.9233	1
GSM36780	0	1	0	0	0	7.00	0	12.9169	7.0420	4.7023	12.1584	10.0708	9.6613	8.4638	5.1708	0
GSM36781	1	1	0	1	0	12.25	0	12.2205	7.9806	4.8963	11.6507	9.6632	8.2937	8.2911	5.0928	0
GSM36782	1	0	1	1	0	5.50	0	9.6770	11.4000	5.3202	12.1686	10.6365	7.8692	8.5960	4.8695	0
GSM36783	1	0	1	1	0	4.33	0	10.6340	12.3625	7.6596	12.4603	11.3658	9.1932	8.7663	5.1929	1
GSM36784	1	0	1	0	0	4.75	1	10.5763	11.4620	4.7821	12.4550	10.9288	8.1266	8.5746	4.6519	0
GSM36785	1	0	1	0	0	4.75	0	9.1491	12.1060	3.8538	12.2783	9.7764	8.9536	8.4652	4.9781	0
GSM36786	1	1	1	1	0	5.50	0	12.3155	9.6284	5.9061	12.7827	11.4799	9.8304	8.8659	5.5922	1
GSM36787	1	0	1	0	0	6.00	0	9.8029	11.4421	4.6874	12.6446	11.4214	7.8915	9.1836	5.3369	1
GSM36788	0	0	0	1	0	6.58	0	8.2130	7.3328	5.1136	12.4208	10.9802	9.8332	9.1316	5.6857	1
GSM36789	1	0	1	1	0	4.25	1	9.3819	11.7068	5.0935	12.7251	9.5449	9.4471	8.4413	5.0317	0
GSM36790	1	0	1	1	0	8.92	0	9.5672	12.3121	5.9162	12.5498	10.6999	10.3517	9.2747	5.1919	0
GSM36791	0	0	1	1	0	6.58	0	10.2173	11.0530	5.4338	12.1087	10.5242	7.8938	8.7716	4.7495	0
GSM36792	1	0	1	0	0	5.92	1	9.9980	11.0193	4.6685	12.3844	10.7608	7.5992	8.5735	5.2875	0
GSM36793	0	0	0	0	0	8.42	0	8.9128	7.0186	3.8916	12.1976	10.1656	8.5788	8.1992	5.2543	0
GSM36794	1	0	0	0	0	7.25	0	8.2951	8.0391	4.4904	12.0400	9.4340	8.9226	8.3983	5.3983	0
GSM36795	0	0	0	0	0	7.33	0	9.2740	7.1785	4.2613	12.4821	11.0970	9.7744	9.0193	5.5152	1
GSM36796	1	0	1	1	0	9.83	0	8.7923	10.6391	5.6307	11.9815	9.9823	8.1029	8.7224	5.2835	0
GSM36797	0	0	0	0	0	0.75	1	8.6293	6.1045	4.4752	12.2086	9.4449	8.0719	8.8236	5.4098	0
GSM36798	0	0	0	0	0	8.83	0	8.3488	7.4564	4.6332	12.2510	9.8234	8.2619	8.7266	5.5120	0
GSM36799	1	1	1	0	0	6.25	0	11.7210	10.8305	4.0955	12.6491	11.2661	9.2731	8.6174	5.2664	1
GSM36800	0	0	1	1	0	3.08	1	10.1951	12.8210	6.4172	12.1182	10.4441	9.4216	8.6991	5.2591	0
GSM36801	1	1	1	0	0	10.42	0	11.3276	11.0369	4.4098	12.0814	9.8356	9.0109	8.3798	5.7477	1
GSM36802	1	0	1	1	0	7.67	0	9.9938	10.1235	6.4832	12.3830	10.9257	9.5454	9.3529	5.6424	1
GSM36803	1	0	1	0	0	7.75	0	10.2757	11.4882	4.5712	12.2235	10.1365	10.7704	8.9473	5.7725	1
GSM36804	1	0	1	1	0	7.33	0	9.9845	9.8811	6.1728	12.2915	10.3808	10.7043	8.4316	5.3276	0
GSM36805	1	0	1	1	0	8.33	0	10.6122	11.9973	6.4637	12.3722	10.7421	9.0870	9.1211	5.4904	0
GSM36806	1	0	1	1	0	7.67	0	10.2786	11.1238	6.6980	12.1449	9.9436	9.1876	8.9780	5.2355	0
GSM36807	1	0	0	1	0	7.33	0	9.7466	8.5846	5.1927	12.4089	11.0150	9.0430	8.8659	4.9728	1
GSM36808	0	0	1	0	0	4.83	0	10.1334	9.8936	4.1962	12.6917	10.4165	7.9408	9.1462	5.0172	0
GSM36809	0	0	0	0	0	4.67	0	7.4446	6.8293	4.3827	11.9705	10.5801	8.1677	8.1689	4.9906	0
GSM36810	1	0	1	1	0	8.75	0	10.0023	11.0019	5.4919	13.2777	11.6940	8.0747	8.7828	5.4950	1
GSM36811	1	0	1	1	0	5.17	1	9.4792	10.5591	5.9721	13.1075	9.8172	10.8079	9.0705	6.0012	1
GSM36812	0	0	1	1	0	6.67	0	9.0656	9.6624	5.4176	12.2733	10.4824	9.2877	8.7686	5.3035	0
GSM36813	1	0	1	0	0	4.08	1	9.5449	10.6673	4.5569	12.9978	9.9546	7.7013	9.4735	5.2845	1
GSM36814	1	1	0	1	0	5.08	1	12.1863	9.3060	5.6457	12.3726	9.8355	9.1144	8.8512	6.1908	1
GSM36815	1	1	1	0	0	6.00	1	12.5593	9.7783	3.8742	12.6304	10.3998	10.1973	9.3220	6.1230	1
GSM36816	0	0	0	0	0	8.17	0	7.6265	8.3028	4.7209	12.1309	10.4912	9.9261	8.5677	5.5984	1
GSM36817	1	0	1	1	0	9.42	0	8.0077	9.9626	5.4359	12.2821	10.4167	10.6382	9.3815	5.0667	0
GSM36818	1	0	1	1	0	5.17	1	8.7629	11.0421	5.7618	12.0786	9.9260	8.5777	8.3449	5.1725	0
GSM36819	1	0	1	1	0	10.92	0	9.2234	10.7575	6.6090	12.1991	10.9881	8.2655	8.9370	5.3998	1
GSM36820	1	0	1	0	0	11.75	0	9.1428	10.3945	4.7695	11.8589	10.6668	8.9518	9.5919	5.4703	0
GSM36821	1	0	1	1	0	7.33	0	9.0995	11.2920	6.4484	12.5134	10.0279	9.0519	8.7004	5.5702	1
GSM36822	0	0	0	0	0	4.17	0	9.3530	6.3030	3.9700	12.1957	11.4967	8.7787	8.7668	5.1045	1
GSM36823	1	0	1	0	0	9.92	0	9.8862	9.9330	4.4495	12.2754	10.6556	9.7802	9.1217	5.8673	1
GSM36824	1	0	0	0	0	7.17	0	9.0725	7.3246	4.6323	12.2401	11.1675	9.1812	9.1845	5.3476	1
GSM36825	1	0	1	1	0	8.25	0	9.7257	10.5518	5.4605	12.2752	10.7904	9.5924	8.7621	5.3967	0
GSM36826	1	0	1	0	0	4.83	1	10.1130	10.9007	4.2878	12.3356	10.6827	8.7773	8.2754	5.2778	0
GSM36827	0	0	0	1	0	13.00	0	8.7837	8.4145	5.2722	12.0838	10.0158	8.8449	8.8331	4.9303	0
GSM36828	0	0	0	0	0	6.33	0	9.3581	6.2651	4.7969	12.4593	10.0700	10.6040	8.6882	5.7385	1
GSM36829	1	0	1	0	0	13.08	0	9.5103	11.7597	4.4960	12.7560	9.8677	8.6746	9.1402	4.9952	0
GSM36830	1	0	1	0	0	9.75	0	9.9793	11.4086	3.6791	12.1671	10.9564	8.8016	8.6578	5.2576	1
GSM36831	1	0	0	0	0	7.25	0	9.8419	8.5867	3.8145	12.0969	10.3091	9.0544	8.8922	5.1642	0
GSM36832	1	0	1	1	0	9.92	0	9.8846	11.0392	5.3127	12.0957	11.2693	9.4385	9.1615	5.9024	1
GSM36833	0	1	0	0	0	4.50	0	12.9529	8.0492	4.3160	12.2156	10.3776	9.3225	8.3570	5.2605	0
GSM36834	1	1	1	0	0	9.08	0	13.0001	11.6344	4.3332	11.8917	10.0576	7.8935	9.2044	5.6143	1
GSM36835	0	0	0	0	0	1.17	1	9.1086	7.0080	4.1634	12.2656	10.1007	8.5961	8.7042	5.6641	1
GSM36836	1	0	0	0	0	8.25	0	10.3091	8.6487	4.3520	13.0512	11.3824	8.2278	9.3540	5.6851	1
GSM36837	1	0	1	0	0	11.42	0	9.4473	10.0994	4.0530	12.5188	10.7711	8.2100	9.1888	5.8393	1
GSM36838	1	0	0	1	0	2.83	1	9.8152	9.0532	4.9987	12.9381	10.3957	7.8324	8.9027	5.8286	1
GSM36839	1	0	1	1	0	2.67	1	10.3582	10.0172	5.4700	12.4306	9.7749	8.5803	8.6202	5.5950	1
GSM36840	1	0	1	0	0	7.17	0	8.8009	11.0352	4.3617	12.5090	10.5042	7.3916	8.8784	5.1218	0
GSM36841	1	0	1	1	0	11.17	0	9.4775	11.0758	5.1528	13.1471	11.1222	8.7525	8.6789	5.5647	1
GSM36842	1	1	1	1	0	6.75	0	13.3194	11.0358	6.5021	12.7331	10.0877	8.2675	8.6543	5.7735	1
GSM36843	1	0	1	1	0	6.42	0	9.9364	12.6648	7.4392	12.5333	11.6427	9.0228	9.7023	5.2720	1
GSM36844	1	0	1	1	0	10.08	0	8.7934	11.1974	4.9826	12.1964	11.4078	9.3489	9.7330	5.4808	1
GSM36845	1	0	1	0	0	4.50	0	10.3269	11.2726	4.7705	12.5780	11.2063	8.6259	8.8827	5.1102	1
GSM36846	0	0	0	1	0	8.58	0	8.6378	7.5470	4.9815	12.5058	10.1223	7.3621	9.6357	5.4906	0
GSM36847	0	0	0	0	0	7.08	0	8.8158	7.2729	4.2675	12.2793	12.1400	9.4024	9.1408	4.6351	1
GSM36848	1	0	1	0	0	8.75	0	10.3888	9.7732	4.5900	12.4943	9.0941	10.3123	9.0517	5.6421	1
GSM36849	1	0	1	0	0	9.17	0	10.0827	12.0138	4.3960	12.1500	10.9349	7.8380	8.7076	5.3985	0
GSM36850	1	0	1	1	0	10.08	0	10.0546	10.1135	4.8210	12.9585	8.9537	9.6009	9.3161	5.7655	1
GSM36851	1	1	0	0	0	8.33	0	11.8646	7.1719	3.9755	12.4205	10.3995	9.1237	8.9897	4.9806	0
GSM36852	1	1	1	1	0	8.92	0	12.0816	10.9755	4.9556	12.3755	10.4633	8.1824	8.7457	5.0692	0
GSM36853	1	0	1	0	0	8.75	0	9.4782	11.1071	4.2473	12.4577	10.4299	9.7723	9.2578	5.5314	0
GSM36854	0	0	1	0	0	8.75	0	7.4220	11.4649	4.6656	11.9609	10.2846	8.4222	8.7638	5.3426	0
GSM36855	0	0	0	0	0	10.67	0	8.7824	6.1118	4.1612	12.1530	9.6306	8.7244	8.2877	5.4466	0
GSM36856	1	1	1	1	0	7.17	0	11.2281	10.2089	7.1823	12.3611	11.2890	10.4570	9.6164	5.2972	1
GSM36857	1	0	1	1	0	7.92	0	9.8181	11.6462	5.0251	12.4247	10.7012	8.9632	8.6786	5.3189	0

GSM36858	1	1	1	0	0	1.17	1	12.5819	10.6760	4.4953	12.5915	9.2634	9.1007	9.0498	5.4934	0
GSM36859	1	0	1	1	0	10.83	0	9.7758	10.6918	5.3344	12.1189	9.5139	8.6092	9.0327	5.1363	0
GSM36860	1	1	0	1	0	2.50	1	12.2094	9.1086	5.3494	12.4520	10.2148	9.2128	8.8262	5.1664	0
GSM36861	1	0	1	1	0	12.92	0	10.5475	11.7428	6.2843	12.2335	9.8950	8.0629	8.8431	5.0732	0
GSM36862	0	0	0	0	0	2.08	1	8.5317	7.0489	4.5797	11.7537	10.1833	8.3735	8.4428	5.2648	0
GSM36863	0	0	1	0	0	8.17	0	9.3117	10.3448	4.7544	11.9030	9.9884	9.6011	8.4386	5.5676	0
GSM36864	1	0	1	0	0	7.00	0	9.5783	10.9462	4.6671	12.4290	11.0046	8.7707	9.1950	5.0341	1
GSM36865	0	0	0	0	0	8.58	0	8.9157	8.4227	4.3778	12.1951	10.1095	8.1801	8.3331	5.1431	0
GSM36866	1	0	1	1	0	8.92	0	10.0856	12.9137	5.7465	12.2805	10.4933	9.0834	8.7787	5.3694	0
GSM36867	1	0	1	1	0	9.00	0	10.1761	12.3435	6.5711	12.0483	10.2658	9.2737	8.4818	5.5702	1
GSM36868	1	0	1	1	0	10.25	0	9.3928	10.8155	5.5277	11.9746	9.8772	9.9599	9.3123	5.4567	0
GSM36869	1	0	1	0	0	10.50	0	9.8580	11.4805	3.8998	12.3020	11.0303	9.3887	8.9173	4.8293	1
GSM36870	1	1	1	0	0	2.50	1	11.3428	11.3825	4.5820	11.9359	10.0062	8.6367	8.9703	5.8706	1
GSM36871	1	0	1	1	0	7.00	0	9.6238	11.1097	4.8620	12.0782	10.2433	7.7207	9.0444	5.3427	0
GSM36872	1	1	1	0	0	0.58	1	12.4515	11.5405	4.0847	12.0990	9.7846	7.8751	8.6076	5.1002	0
GSM36873	1	0	1	1	0	8.33	0	9.1227	11.3467	5.3946	12.0090	10.1324	8.4217	8.6494	5.3401	0
GSM36874	1	0	1	1	0	2.50	1	9.6101	11.2552	5.0679	12.5579	10.7290	9.2056	8.8088	5.5750	1
GSM36875	0	0	0	0	0	0.58	1	8.2949	4.7487	4.3512	12.0936	9.4840	9.5276	9.1342	5.7306	1
GSM36876	0	0	0	0	0	11.08	0	9.2993	6.1488	4.5617	12.3224	9.9256	10.5647	9.3105	5.8871	1
GSM36877	1	0	1	0	0	3.58	1	8.6618	10.7914	4.5507	12.5710	10.8882	9.9955	9.3669	5.1077	0
GSM36878	1	0	1	1	0	8.17	0	9.7427	11.3910	4.9427	12.7307	10.9985	9.8480	9.4306	5.3378	1
GSM36879	0	1	0	0	0	3.25	1	12.7058	8.2743	4.2728	12.0738	10.5946	8.6865	8.4720	5.3222	0
GSM36880	1	0	1	1	0	8.08	0	9.2379	10.9122	5.0117	12.0223	10.3793	8.9604	9.2504	5.5188	0
GSM36881	1	0	1	1	0	0.92	1	9.8834	12.1948	5.4143	12.4515	10.5349	9.1555	8.4377	5.6439	1
GSM36882	1	0	1	1	0	8.25	0	9.8264	10.7024	5.0981	12.2195	10.2773	8.2440	9.2073	5.4755	0
GSM36883	1	0	0	0	0	7.33	0	9.3808	8.6316	4.1719	12.3263	10.4576	9.1134	8.7250	5.1159	0
GSM36884	1	1	1	0	0	10.75	0	12.3328	10.7036	4.7957	12.5098	10.6635	8.9521	8.9236	5.3314	0
GSM36885	1	0	0	1	0	3.33	1	9.4230	9.5048	6.0960	12.1351	10.2785	9.7404	9.1252	4.9863	0
GSM36886	0	0	1	0	0	7.83	0	8.6950	11.1141	4.7776	12.2228	9.5050	9.9320	8.5484	5.0065	0
GSM36887	1	0	1	0	0	9.00	0	9.0314	12.2108	4.7820	12.0199	11.2276	9.1787	8.7299	5.4971	1
GSM36888	1	0	1	1	0	2.33	1	8.8783	12.7416	4.9257	12.1884	10.6914	10.3872	9.2763	5.8925	1
GSM36889	1	0	1	0	0	9.00	0	7.7924	9.5691	4.4957	12.2683	9.9856	9.0137	8.5809	4.7387	0
GSM36890	1	0	0	0	0	9.67	0	9.1419	9.2523	4.3325	12.3783	10.4227	8.9685	8.7738	5.1479	0
GSM36891	0	0	0	0	0	7.25	0	9.5528	5.7717	4.3699	11.9958	10.6556	9.1411	9.1614	5.5754	1
GSM36892	1	0	1	1	0	9.17	0	10.6910	10.5226	5.0637	12.0795	11.3159	9.6439	9.0825	5.6993	1
GSM36893	1	0	1	0	0	10.33	0	10.1086	12.1676	4.6237	12.3420	11.1596	10.1756	9.1389	5.4116	1
GSM36894	1	1	1	0	0	9.08	0	12.1669	11.0356	4.7473	12.0874	10.6636	8.6312	8.5212	5.2231	0
GSM36895	1	0	1	0	0	8.00	0	9.9274	12.3826	4.3100	12.4119	11.3276	8.5330	8.3301	5.0151	1
GSM36896	1	0	1	1	0	8.50	0	10.5513	12.0146	5.2867	12.1971	10.9722	9.6464	8.9806	5.5416	1
GSM36897	1	0	1	0	0	2.08	1	10.2422	9.8070	4.5370	12.8083	10.0003	9.2603	9.0389	6.4144	1
GSM36898	1	0	1	1	0	0.58	1	9.7974	12.9395	5.1182	12.3391	10.2635	8.6246	8.9698	4.6170	0
GSM36899	1	0	1	0	0	14.08	0	9.9586	11.4624	4.4466	12.2417	11.2728	10.9411	9.3616	5.5761	1
GSM36900	1	0	0	1	0	9.08	0	9.4459	6.8763	5.0206	12.7422	9.2485	10.0968	10.0899	5.6934	1
GSM36901	1	0	0	0	0	8.42	0	9.0883	8.2095	4.5124	11.5594	9.8733	9.0480	8.2125	5.4314	0
GSM36902	1	0	0	0	0	2.33	1	8.6886	6.3496	3.6910	12.3284	10.7700	9.2429	8.2038	5.2210	0
GSM36903	1	0	0	0	0	0.67	1	8.5108	9.4818	4.1596	11.8399	9.5082	10.2308	8.6615	5.7481	1
GSM36904	0	1	0	0	0	8.17	0	12.9926	8.1998	4.0470	12.5483	11.1958	9.1175	8.7268	5.2022	1
GSM36905	0	0	0	0	0	1.42	1	6.4085	5.4949	3.7993	11.8870	10.2851	9.4859	8.7970	5.6492	1
GSM36906	0	0	0	0	0	8.42	0	9.5064	7.9052	4.5992	12.3329	10.8397	9.1600	9.1759	4.7583	0
GSM36907	1	0	1	1	0	7.17	0	10.1003	11.9726	6.5456	11.8863	10.0114	9.1132	8.9313	5.1740	0
GSM36908	1	0	1	1	0	1.25	1	9.9591	11.8457	5.6701	11.7130	10.6068	10.1347	8.7313	5.5889	1
GSM36909	0	0	0	0	0	7.92	0	8.1309	7.8664	4.3604	11.8072	9.7533	8.7661	8.3570	4.8915	0
GSM36910	1	0	1	1	0	10.42	0	9.2139	10.3340	5.6732	12.7150	10.8318	9.0537	9.5846	5.2287	0
GSM36911	1	0	1	0	0	6.67	1	9.4061	10.9512	3.5144	12.2456	11.0351	8.6187	9.0603	5.2995	1
GSM36912	0	0	0	0	0	8.08	0	7.7128	6.9948	4.1456	12.3721	10.4122	8.7086	8.8498	5.2894	0
GSM36913	1	0	1	0	0	9.08	0	9.7348	12.4472	4.6377	12.4058	10.4531	9.0773	9.1328	4.9184	0
GSM36914	1	0	1	0	0	7.00	0	8.6200	11.4894	4.5526	12.2246	10.8208	8.6623	8.5697	5.3920	0
GSM36915	0	1	0	0	0	8.08	0	12.4804	7.3260	4.5036	12.5444	11.2367	9.3714	8.8938	5.0544	1
GSM36916	1	0	1	1	0	10.42	0	9.4839	10.3232	5.3809	12.3662	10.6758	9.1313	8.8598	5.6301	1
GSM36917	1	0	1	0	0	12.00	0	9.6514	11.1288	4.7328	12.0658	10.3876	8.6995	9.1250	5.7557	1
GSM36918	0	0	0	0	0	0.92	1	9.0720	8.8423	4.2834	12.0923	11.6053	8.9501	9.3355	5.6560	1
GSM36919	1	0	1	1	0	12.75	0	9.5961	11.1290	6.7166	12.2885	10.7999	8.0570	9.7984	5.3961	0
GSM36920	1	1	1	1	0	1.67	1	11.5145	10.5213	5.1747	12.1501	10.7219	9.6013	8.5309	5.1078	0
GSM36921	1	0	1	1	0	9.00	0	10.6515	10.1239	5.7127	12.1604	10.3811	9.1103	9.4301	5.7427	1
GSM36922	1	0	1	0	0	9.42	0	9.6397	11.9407	3.6480	12.1657	10.7194	9.1586	8.6697	5.2434	0
GSM36923	0	0	0	0	0	1.92	1	9.1721	5.7335	4.0479	12.2216	10.8530	8.6482	8.4605	5.1008	0
GSM36924	1	0	1	0	0	2.42	1	9.3657	11.0840	4.5021	12.0038	10.6066	9.8957	8.8239	4.9234	0
GSM36925	1	0	1	0	0	8.67	0	10.2934	10.5881	4.4728	12.3650	10.4705	9.6169	8.8461	4.8468	0
GSM36926	0	1	0	0	0	2.00	1	12.0658	7.5131	4.2389	12.4158	10.5823	8.8636	9.3577	4.8444	0
GSM36927	1	0	0	1	0	0.67	1	9.5945	9.1656	4.9167	11.4137	10.0657	9.6776	8.7638	5.0330	0
GSM36928	1	0	1	0	0	1.92	1	10.1240	11.2633	4.2590	11.8234	10.4510	9.8002	8.6360	5.3543	0
GSM36929	1	0	1	1	0	7.75	0	11.0556	11.0180	5.0698	12.7535	11.0303	9.3298	9.3258	5.2796	1
GSM36930	1	1	1	0	0	7.00	0	12.5749	11.9518	4.5151	12.3410	11.2823	8.2321	8.6245	5.5146	1
GSM36931	1	0	0	0	0	3.08	1	9.3144	7.5970	4.6466	12.1028	10.7994	8.8665	8.6935	5.2093	0
GSM36932	1	0	0	0	0	8.00	0	9.3547	9.1479	4.0784	12.2970	10.4642	7.4110	8.5225	5.0973	0
GSM36933	1	0	1	1	0	9.17	0	8.9528	11.5394	5.1098	12.3463	9.8774	9.3367	8.3411	5.3766	0
GSM36934	1	1	1	1	0	14.25	0	11.8767	12.6572	5.1541	11.9734	10.8758	7.9516	9.3562	5.4102	0
GSM36935	0	0	0	0	0	9.50	0	7.6367	7.2158	4.3973	12.1094	10.7498	8.3973	8.8982	5.1476	0
GSM36936	1	0	0	0	0	9.08	0	9.7675	9.4297	3.8381	12.3848	10.1107	8.5618	8.3621	4.6675	0
GSM36937	0	0	0	0	0	0.92	1	9.3167	8.0084	4.7178	11.9044	10.8838	9.9310	8.9359	4.9102	0
GSM36938	1	0	1	0	0	9.00	0	9.6541	10.8033	4.6790	12.1386	10.6202	10.2683	9.0940	5.0182	0
GSM36939	1	0	1	1	0	1.17	1	8.9985	12.4546	5.8679	12.0277	10.0535	9.4930	9.4134	5.1352	0
GSM36940	0	0	0	0	0	11.17	0	8.1604	6.4159	4.7181	11.9218	10.5786	10.9160	9.4654	5.5111	0
GSM36941	0	0	0	1	0	0.75	1	10.0166	7.5383	5.2018	12.2234	11.9447	9.8083	9.3009	5.3932	1
GSM36942	1	0	1	1	0	12.67	0	9.7291	9.5726	5.8914	12.3806	9.7649	9.6692	9.3003	5.5406	0
GSM36943	1	0	1	0	0	1.58	1	9.4833	10.3454	3.9119	12.6636	11.4528	9.3227	8.8285	5.0005	1
GSM36944	1	0	1	1	0	9.33	0	9.5017	9.7574	5.9944	12.1640	9.8109	9.5251	9.0656	5.3973	0
GSM36945	1	0	1	1	0	7.67	0	9.7914	11.4598	4.9283	12.5063	10.8958	8.7391	8.7747	5.0645	0
GSM36946	1	0	1	1	0	2.17	1	10.3312	11.8889	5.3077	12.3609	11.4993	8.7947	9.2763	5.1722	1
GSM36947	1	0	1	0	0	1.33	1	9.6300	12.2205	4.6328	12.1811	11.8470	8.5540	9.5145	5.5441	1
GSM36948	1	0	1	0	0	7.25	0	10.3310	12.1392	4.5310	12.2120	10.3429	8.9859	9.2759	5.4702	0
GSM36949	0	0	0	1	0	0.50	1	7.8709	8.1567	4.9175	12.2988	11.4162	8.7638	9.4330	5.1687	1
GSM36950	1	0	1	1	0	2.33	1	9.9937	12.1021	5.8624	12.3059	9.7803	9.4628	9.4213	5.1903	0
GSM36951	1	0	1	1	0	8.67	0	10.4081	12.6936	6.4647	12.7794	11.0278	8.8169	8.3464	5.0416	1
GSM36952	0	0	0	0	0	1.08	1	8.5408	7.2232	4.2415	11.9541	10.1919	8.5422	8.4856	4.8773	0
GSM36953	0	1	0	1	0	7.00	0	12.6725	7.8112	4.9439	12.3211	10.5930	8.8554	8.9611	5.7869	1
GSM36954	1	0	1	1	0	1.58	1	10.4009	13.4375	5.1459	12.6786	10.3375	7.4354	8.6882	5.1483	0
GSM36955	0	0	0	0	0	2.08	1	9.2361	7.7648	4.4085	12.2397	10.5436	9.2867	9.0762	5.1843	0
GSM36956	1	0	1	1	0	2.67	1	9.9366	11.3116	5.9819	12.8758	10.2586	9.4711	9.4783	5.5289	1
GSM36957	1	0	1	0	0	3.92	1	9.7913	10.8190	4.3159	12.5705	10.1218	9.3956	8.7462	5.5298	0
GSM36958	1	0	1	1	0	9.25	0	8.8898	10.4485	5.4251	12.0352	10.9718	9.6581	9.0151	5.2939	1
GSM36959	0	0	0	0	0	10.00	0	9.0725	6.2021	4.5149	11.8498	10.7052	10.0359	8.9898	5.6020	1
GSM36960	1	0	0	0	0	4.25	1	9.2373	7.1333	4.7069	12.1728	10.1168	10.0611	8.3525	5.0004	0
GSM36961	0	0	0	0	0	12.75	0	10.6765	7.0451	4.3821	12.1636	9.9074	8.2453	8.4582	5.3902	0
GSM36962	1	0	1	1	0	9.50	0	9.1898	10.8560	7.1996	11.9290	10.8128	9.6204	9.4518	5.0415	0

GSM36963		1		1	1	1	0			5.00	0	11.6632	11.4546	5.4147	12.1855	11.1365	8.7080	9.0923	5.6382	1
GSM36964		0		1	0	0	0			1.00	1	12.9144	7.6111	4.2944	11.8653	10.1819	9.8797	9.0065	5.0981	0
GSM36965		1		0	0	1	0			11.17	0	8.0346	9.3199	5.5370	11.9059	10.5348	9.3642	9.1849	5.5018	0
GSM36966		0		0	0	0	0			7.50	0	9.4795	5.3751	4.1393	12.0935	10.4111	9.7130	9.1733	5.6291	1
GSM36967		1		0	1	0	0			1.25	1	9.1650	10.0502	3.4756	12.2347	9.2598	9.2398	8.6083	5.4177	0
GSM36968		0		1	0	0	0			12.67	0	12.8403	6.2243	3.9241	12.0983	10.2994	8.9718	8.9822	5.2239	0
GSM36969		0		0	0	0	0			2.67	1	8.7457	6.9042	4.2633	12.1221	9.8782	9.1432	9.1805	5.2153	0
GSM36970		1		0	1	0	0			9.67	0	9.8026	10.1636	4.1006	12.8265	10.8860	9.3784	8.6675	5.1887	1
GSM36971		1		0	1	0	0			1.58	1	8.9979	10.5345	4.5238	12.0941	10.0112	9.3888	8.6400	5.4946	0
GSM36972		1		0	1	0	0			3.08	1	10.0347	12.1767	4.3456	12.1615	9.8332	8.6881	8.8386	5.1533	0
GSM36973		1		0	1	1	0			2.08	1	9.8874	11.4781	5.9112	12.4352	10.7765	9.4874	8.7548	5.2754	0
GSM36974		1		0	1	0	0			1.92	1	10.2685	13.9467	4.3886	12.3575	10.8288	8.8896	8.9322	5.1197	0
GSM36975		1		1	0	0	0			9.17	0	12.7925	8.0721	4.4507	12.2186	10.6103	8.2781	8.6758	5.1793	0
GSM36976		1		0	1	1	0			3.17	1	9.3139	12.3193	5.6237	12.6052	10.8253	9.5225	9.1138	5.3116	0
GSM36977		0		0	0	0	0			10.33	0	9.3636	8.7351	4.8130	12.3279	10.3380	7.8462	8.3852	5.2483	0
GSM36978		0		1	0	0	0			12.33	0	13.0302	7.6418	4.2835	12.5426	11.3008	8.8043	8.5675	5.1084	1
GSM36979		1		0	1	1	0			8.92	0	10.2135	11.9247	5.0509	12.5139	11.5700	8.5117	8.8175	5.3226	1
GSM36980		1		0	1	1	0			10.17	0	9.1424	12.6716	5.4168	11.5759	10.6854	9.1017	8.7980	5.2209	0
GSM36981		0		0	0	1	0			7.25	0	8.8278	8.2048	5.0739	12.5029	10.3513	9.6607	7.8452	5.1109	0
GSM36982		1		0	1	0	0			7.67	0	9.6670	9.7053	4.4073	12.4641	10.8850	9.3571	9.1281	4.9126	0
GSM36983		1		0	1	1	0			6.25	1	8.8994	11.6880	5.1396	11.8202	10.1316	8.4876	8.3643	5.2043	0
GSM36984		1		0	1	0	0			11.50	0	9.6378	13.2928	4.3714	11.7261	11.4212	8.8164	8.4204	5.5181	1
GSM36985		1		0	1	1	0			4.58	1	9.3188	10.6503	4.9474	12.6402	11.5433	8.6070	9.7853	5.6056	1
GSM36986		1		0	1	0	0			6.42	1	9.3647	10.5999	4.0461	12.7253	11.1906	8.3094	8.2069	5.0971	1
GSM36987		1		0	1	0	0			8.08	0	9.5347	10.7279	4.6535	11.8928	10.3567	8.7783	9.0142	5.6769	1
GSM36988		1		0	1	1	0			8.00	0	10.1226	12.2629	5.1271	12.0383	10.8216	8.1727	8.9363	5.2399	0
GSM36989		1		0	1	0	0			0.92	1	9.2348	10.1754	3.7224	12.1958	9.9331	8.5053	8.5175	5.3859	0
GSM36990		1		0	1	1	0			11.92	0	9.6586	10.7978	4.8544	12.2309	10.6718	8.9141	8.7725	5.2947	0
GSM36991		0		0	0	0	0			9.50	0	9.2144	5.9285	3.9633	12.0551	10.4091	9.3719	8.4938	5.2315	0
GSM36992		1		1	0	0	0			8.67	0	13.1206	7.2291	3.7168	12.5059	9.9677	8.9044	8.6007	5.1970	0
GSM36993		1		0	1	0	0			8.17	0	9.5085	10.7181	3.7133	11.8339	10.2104	8.1395	8.8900	5.4394	0
GSM36994		1		0	1	0	0			0.17	1	9.6156	10.5210	4.6077	12.1239	9.8301	9.0285	8.8165	5.5704	1
GSM36995		1		1	1	1	0			9.42	0	12.4675	11.4698	5.3965	12.1471	10.3619	8.2681	8.4563	5.3186	0
GSM36996		1		1	0	1	0			2.00	1	12.1710	8.9021	5.1554	12.6496	10.7915	9.3268	9.0513	5.4984	0
GSM36997		1		0	0	0	0			3.67	1	9.8202	9.4984	4.4016	13.0841	9.3803	9.2201	9.6534	5.7693	1
GSM36998		1		0	1	1	0			0.42	1	10.0808	11.1557	6.2906	12.3333	10.1121	8.7879	8.4486	4.9397	0
GSM36999		1		0	1	0	0			3.08	1	9.4838	10.4824	4.1686	12.6122	10.8098	9.1106	8.8860	5.3046	0
GSM37000		1		0	0	0	0			11.42	0	9.3791	8.2493	4.7230	12.1649	10.2387	8.4272	8.9049	5.4372	0
GSM37001		1		1	1	0	0			2.75	1	13.2683	9.9233	4.5586	12.6089	11.2362	9.1868	8.7012	5.0491	1
GSM37002		0		0	0	0	0			1.33	1	8.7781	5.9791	4.3161	12.3061	10.2748	8.7226	8.6375	5.1738	0
GSM37003		1		0	1	0	0			1.58	1	9.4990	12.0005	4.7758	12.3700	11.0231	7.9623	8.8201	5.3028	1
GSM37004		1		0	1	0	0			2.92	1	9.7257	12.2158	3.8119	12.1269	10.4651	8.6480	8.5273	5.3511	0
GSM37005		1		0	1	1	0			4.67	1	10.5471	11.9825	6.3245	12.3385	9.3799	7.8219	9.7986	5.2116	0
GSM37006		1		0	1	1	0			5.92	1	9.2440	11.3515	4.9509	12.7212	10.7505	7.5296	9.3486	5.5062	0
GSM37007		1		0	1	0	0			4.92	1	9.3655	12.4932	4.1817	12.3201	10.9099	7.6662	8.7395	5.2452	0
GSM37008		1		0	1	0	0			5.83	1	8.3393	11.2557	4.1783	12.4007	11.1565	7.2392	9.0344	5.3752	1
GSM37009		1		0	1	0	0			10.17	0	9.0173	11.8188	4.6380	12.6440	10.0120	8.3424	8.7625	4.9499	0
GSM37010		1		0	1	1	0			8.42	0	9.9508	12.3592	6.6064	12.4845	11.3391	9.1065	9.1956	5.1266	1
GSM37011		1		0	1	1	0			5.00	1	10.1749	12.7329	6.0922	12.6464	11.2790	8.3741	8.6252	5.1468	1
GSM37012		1		1	1	1	0			11.00	0	11.1735	11.2867	5.0971	11.9883	10.5720	8.1408	8.9506	5.2070	0
GSM37013		1		0	1	0	0			5.50	1	8.8665	11.0736	4.1498	11.8324	10.7827	7.6764	8.4434	5.0402	0
GSM37014		1		0	1	0	0			11.92	0	9.9026	12.3337	4.3660	12.0997	10.1197	8.5044	8.7386	5.2697	0
GSM37015		1		0	1	0	0			10.17	0	10.4330	11.0191	4.1213	12.4520	10.9808	8.3160	8.5046	5.0429	1
GSM37016		0		1	0	0	0			9.00	0	12.6264	6.6822	4.4961	12.4029	10.2359	8.8106	8.2337	5.2549	0
GSM37017		0		0	0	0	0			9.00	0	9.8094	7.4579	4.2703	12.1658	10.9057	10.1666	9.3709	5.0719	0
GSM37018		1		0	1	0	0			1.67	1	10.0411	12.6150	4.5860	12.1419	10.6099	9.2886	9.9432	5.2213	0
GSM37019		1		1	0	0	0			12.17	0	11.9421	9.3311	4.5598	12.2495	10.8813	8.7760	8.8721	5.5015	0
GSM37020		0		1	0	0	0			4.00	1	13.0497	8.2864	4.4463	12.1175	9.9322	8.8256	8.1510	5.1154	0
GSM37021		0		0	0	0	0			9.08	0	8.3146	6.0545	4.0939	12.1921	10.3425	9.6517	9.3610	5.4820	0
GSM37022		0		0	0	0	0			2.50	1	8.6403	6.6662	4.2395	11.8897	10.4892	10.0438	8.7228	5.7019	1
GSM37023		0		1	0	0	0			1.17	1	12.9928	7.5311	4.2480	12.4792	9.7295	8.8943	8.4794	5.3216	0
GSM37024		1		0	1	1	0			7.92	0	10.0933	10.0347	6.2492	12.1573	10.0079	8.9764	8.5375	5.2927	0
GSM37025		1		1	1	1	0			8.92	0	11.2472	12.0718	5.2056	12.4414	11.1185	8.5740	9.0775	5.6054	1
GSM37026		1		0	1	0	0			3.00	1	10.0874	9.7197	4.0795	12.4292	10.7215	9.5538	8.5946	5.5257	0
GSM37027		1		0	1	0	0			1.42	1	9.4731	11.4527	4.2637	12.4285	11.2544	8.8421	8.8996	4.8138	1
GSM37028		1		0	1	1	0			3.25	1	9.4851	12.6715	8.1394	12.7373	11.2219	8.6590	9.6356	4.8875	1
GSM37029		1		0	1	1	0			3.92	1	10.2307	13.0817	5.0219	12.7405	10.7165	7.6146	8.4996	4.9589	0
GSM37030		1		0	1	0	0			3.67	1	9.9718	11.1128	3.4618	12.0895	10.9501	7.9061	9.1886	4.7483	1
GSM37031		1		1	1	0	0			1.50	1	12.5503	11.4830	4.6943	12.3885	9.8844	8.2396	8.5417	5.3594	0
GSM37032		1		0	1	0	0			7.17	0	9.9657	12.4126	4.6409	12.2462	10.6005	8.0178	8.8481	5.1758	0
GSM37033		1		1	1	1	0			13.42	0	11.8086	11.3262	6.8385	12.3512	11.2977	8.3272	9.3196	5.2856	1
GSM37034		0		1	0	1	0			7.33	0	12.0763	8.1140	4.9722	12.1793	10.4480	9.8232	8.6693	5.3198	0
GSM37035		1		0	1	1	0			1.17	1	9.9758	11.9673	4.8629	12.6915	11.6400	8.2504	9.1168	5.0423	1
GSM37036		1		1	0	0	0			1.25	1	13.1555	9.3994	3.6373	12.6592	10.4810	8.8985	8.8419	5.0402	0
GSM37037		1		0	1	0	0			1.58	1	10.6215	11.5947	4.0552	12.9529	11.3174	9.7764	9.1273	5.4815	1
GSM37038		1		0	1	1	0			5.00	1	10.1008	12.9922	5.9131	11.8645	10.4319	7.5319	9.2008	5.3748	0
GSM37039		1		0	1	0	0			4.00	1	9.8460	11.8031	4.2998	11.8085	10.4981	8.2282	8.8486	4.9629	0
GSM37040		0		0	0	1	0			0.50	1	8.4589	7.3722	4.8408	12.0974	11.5511	8.3997	8.7693	5.0961	1
GSM37041		1		0	1	1	0			0.75	1	10.4110	9.6704	5.1034	12.4990	11.0860	8.0746	9.0457	4.8292	1
GSM37042		0		1	0	1	0			1.17	1	13.1618	8.3373	4.8269	12.3435	10.5180	9.9114	8.1076	5.0107	0
GSM37043		0		0	0	0	0			13.42	0	8.0185	6.2136	4.5199	12.2165	11.0211	9.2291	8.7942	5.0926	1
GSM37044		1		0	0	0	0			9.33	0	10.0850	7.0628	4.1530	12.4323	9.4395	7.9355	8.5656	5.2449	0
GSM37045		0		0	0	0	0			10.25	0	8.6005	7.4813	4.4258	12.4539	10.1626	8.5748	9.0031	5.1729	0
GSM37046		1		0	1	0	0			7.17	0	10.1103	11.7658	4.3666	12.0342	10.5046	9.4552	8.8639	4.8203	0
GSM37047		1		0	0	1	0			9.00	0	9.4887	7.7132	4.8417	12.3120	10.2945	7.6285	8.4601	4.7860	0
GSM37048		0		0	0	0	0			7.25	0	8.4696	6.6939	3.7587	12.3440	10.0699	8.3877	8.5219	5.3755	0
GSM37049		0		1	0	0	0			2.42	1	11.5208	9.4942	4.1004	12.3145	9.8306	8.4777	8.9422	5.6072	1
GSM37050		0		0	0	0	0			1.50	1	7.6683	6.7745	4.3353	11.9830	10.1262	8.6669	8.6598	4.9194	0
GSM37051		1		0	0	0	0			1.50	1	8.4596	6.8237	4.4482	12.4484	10.9813	8.4273	8.7951	4.8861	1
GSM37052		0		1	0	0	0			2.67	1	11.3677	7.7361	4.5949	12.5594	10.0369	8.0774	7.8634	4.7637	0
GSM37053		0		0	0	1	0			2.75	1	8.8972	7.8916	4.8895	12.3789	9.6150	9.1615	8.7936	4.8153	0
GSM37054		0		1	0	0	0			5.50	0	13.6099	7.3993	4.3310	13.1642	11.2458	8.6472	8.7377	4.9951	1
GSM37055		0		1	0	1	0			4.92	0	13.2022	9.0663	5.8530	12.5346	9.9292	8.8186	8.3234	5.3282	0
GSM37056		0		0	0	0	0			6.83	0	10.2139	8.6039	4.1244	12.2781	10.0286	8.1043	8.9198	5.6647	1
GSM37057		1		0	1	1	0			4.92	0	9.1568	12.4054	5.0096	12.0087	10.2601	8.4427	8.4472	5.0500	0
GSM37058		1		0	1	0	0			5.33	1	9.4452	11.8514	4.5545	12.4744	11.4071	8.6178	8.3821	5.3877	1
GSM37059		1		0	1	0	0			6.92	0	9.6354	11.5286	4.3005	12.4839	11.0731	9.0688	9.2102	5.3804	1
GSM37060		1		1	1	1	0			6.75	0	12.1797	10.6961	5.0506	12.5437	10.4860	8.3942	9.3646	5.5740	1
GSM37061		0		0	1	1	0			8.75	0	10.0170	10.9714	4.9409	13.0729	11.5458	9.2479	9.7207	5.0221	1
GSM37062		1		0	1	1	0			4.50	0	8.8988	10.9976	5.2376	12.5925	10.9574	8.3465	9.3728	5.3699	1
GSM411235	0	1	1	0	1	0	1	2	54	2.11	1	10.2744	10.3370	4.0224	12.6384	10.7871	9.0470	8.7096	4.7879	0
GSM411236	0	1	0	0	1	1	0	3	65	3.32	0	8.8328	12.9950	5.4178	12.4592	10.4669	8.5160	9.7410	5.1620	0
GSM411237	NA	1	1	0	1	0	1	2	71	5.18	1	9.1460	12.1412	4.4719	13.0209	11.2882	9.4020	10.1794	6.3368	1
GSM411238	0	1	1	0	1	1	0	2	65	4.53	0	9.7185	12.4475	8.5065	12.2644	10.0502	9.3322	9.4402	5.0221	0
GSM411239	0	1	1	0	1	1	1	2	56	5.49	0	9.5272	10.3743	5.8451	12.3093	10.0973	9.6351	8.7750	5.2845	0

GSM411240	NA	1	1	1	1	1	1	2	52			2.12	1			11.2123	11.2202	7.2531	12.0555	9.8883	8.9380	8.6863	4.9650	0
GSM411241	0	1	0	0	1	0	1	3	70			1.20	0			10.2932	12.0757	3.1508	13.1452	11.3127	9.7882	9.8313	6.1875	1
GSM411242	0	1	0	0	1	1	1	2	51			4.97	0			9.4234	10.5529	6.2974	12.3130	9.9957	9.0624	8.8101	5.7470	1
GSM411243	0	1	1	0	1	1	0	2	66			5.03	1			9.4016	9.7528	5.0129	12.5785	10.3507	9.8898	9.0484	5.8493	1
GSM411244	0	1	1	0	1	0	1	2	72			2.07	0			8.6995	11.6876	4.4675	12.2196	10.7785	9.0707	10.4987	6.5617	1
GSM411245	0	1	1	0	1	1	0	2	58			2.32	0			10.3516	11.0941	5.8170	11.7231	10.5056	8.7165	9.2199	6.0734	1
GSM411246	0	1	1	0	1	1	1	2	52			2.08	0			9.3392	10.1757	8.3394	12.7406	10.2902	9.6640	8.9017	5.3232	0
GSM411247	1	1	1	1	1	0	1	3	67			0.93	0			11.3413	11.8375	3.8264	12.4789	10.9858	9.4227	8.5797	5.6707	1
GSM411248	0	1	1	0	0	0	0	2	69			2.18	0			9.7213	8.5757	4.7640	12.1602	10.2084	10.0193	8.8454	5.6194	1
GSM411249	NA	1	1	0	1	0	0	2	78			4.86	0			9.8562	12.6406	4.3727	11.8884	9.8932	8.4684	8.4097	4.1442	0
GSM411250	0	1	1	0	1	1	1	2	67			4.66	1			9.6834	12.0674	6.8114	12.2667	10.1062	9.3829	9.0183	5.2334	0
GSM411251	0	1	1	0	1	1	0	2	56			4.62	0			9.8371	11.9307	6.2771	12.7704	10.1837	9.5167	9.3938	5.2482	0
GSM411252	0	1	0	0	1	0	1	2	52			1.98	0			8.9324	10.5142	4.2862	12.5200	10.3990	9.1125	8.7772	4.8045	0
GSM411253	0	1	0	0	1	0	0	3	74			2.10	1			10.1315	12.5110	3.6847	12.5968	11.2045	9.4575	9.0647	5.2108	1
GSM411254	0	1	0	0	1	0	1	1	63			4.21	1			9.8895	11.7296	3.9703	12.1618	9.8067	9.3945	8.6662	5.1514	0
GSM411255	0	1	0	0	1	0	1	3	58			2.13	0			9.0826	12.3784	3.6394	11.7683	10.0409	8.9290	8.4486	4.8725	0
GSM411256	0	1	1	0	1	1	0	2	68			2.08	1			9.7540	12.6151	6.5870	12.2472	9.8272	9.6756	8.8763	5.2872	0
GSM411257	0	1	0	0	1	1	1	2	58			2.29	1			9.0269	11.8629	4.9036	12.2292	9.8730	9.5037	8.6742	5.0265	0
GSM411258	0	1	1	0	1	1	0	2	76			4.10	0			9.6298	11.6407	6.4615	11.9560	9.4087	9.4253	8.3252	5.0669	0
GSM411259	0	1	1	0	1	0	0	1	57			4.06	0			9.6787	10.6476	4.6529	12.4123	10.2879	9.0103	8.7562	5.5918	1
GSM411260	0	1	1	0	1	0	1	2	59			2.12	1			10.0263	10.0051	4.5726	12.2908	10.4111	9.8177	8.7641	5.7064	1
GSM411261	0	1	1	0	1	1	1	2	60			4.08	0			9.8567	13.4093	6.4881	12.3594	10.3105	8.8609	9.9918	5.5569	0
GSM411262	NA	1	1	0	1	1	1	3	56			2.15	1			9.3001	12.7366	7.5115	11.9960	10.5322	9.6584	8.7294	5.5336	0
GSM411263	NA	1	1	0	1	1	1	2	59			2.11	0			9.5364	12.0838	5.8059	11.7682	10.3264	9.5441	9.4056	5.4568	0
GSM411264	1	1	1	1	1	0	1	3	46			2.08	0			12.6242	10.2275	4.1382	12.6233	10.5561	9.7287	8.7071	5.1307	0
GSM411265	0	1	1	0	1	1	0	3	53			3.98	1			9.9927	13.0074	5.8920	12.3895	10.2319	9.1901	9.4165	5.9570	1
GSM411266	0	1	1	0	1	1	0	3	68			1.08	0			10.2528	12.2094	6.0859	12.0131	11.0483	8.5070	9.0558	4.8089	1
GSM411267	NA	1	0	0	1	1	1	3	55			2.28	1			8.7603	11.6592	4.8692	12.3629	10.6377	9.3973	8.8993	5.1783	0
GSM411268	0	1	1	0	1	0	0	3	53			1.58	0			8.2656	10.8744	4.0579	12.2335	9.8794	9.2707	9.1678	4.9141	0
GSM411269	NA	1	1	0	1	1	1	2	58			3.70	0			9.4619	10.6729	4.8522	12.4563	10.5360	9.6787	9.2365	5.1921	0
GSM411270	0	1	1	0	1	1	1	2	54			2.08	0			9.9856	11.2845	5.0853	12.4634	10.6439	9.0363	8.8925	5.1126	0
GSM411271	0	1	1	0	1	0	1	3	70			3.33	1			9.6953	11.7659	4.5051	12.6707	10.7964	8.6850	9.1464	5.6717	1
GSM411272	0	1	1	0	1	0	1	3	62			2.18	1			10.3190	12.7158	3.5855	12.5129	11.0964	9.4742	8.8708	6.0370	1
GSM411273	0	1	0	0	1	0	0	2	56			1.13	1			10.6386	10.0939	3.2351	12.4821	10.8283	9.3394	9.5283	5.5164	0
GSM411274	0	1	1	0	1	1	0	2	69			2.16	0			9.6896	11.6663	5.3199	12.1126	10.0113	9.4297	9.1063	5.4156	0
GSM411275	0	1	1	0	1	0	1	2	69			2.11	0			9.5222	11.8116	3.7863	12.2206	9.7732	9.2097	8.8199	5.0302	0
GSM411276	0	1	1	0	1	0	1	3	56			2.09	1			10.0210	11.8901	3.2221	12.5489	10.3636	9.3824	9.4033	5.2929	0
GSM411277	NA	1	1	0	1	0	1	2	53			3.39	1			9.4403	11.6409	4.4888	12.1951	10.2380	9.1100	8.3986	4.9835	0
GSM411278	0	1	1	0	1	1	1	2	71			3.58	0			9.1790	13.0683	9.4980	12.7246	10.9640	10.2108	9.8327	5.7786	1
GSM411279	0	1	1	0	1	1	0	3	76			3.50	0			9.5830	12.6917	4.9616	12.3683	10.7244	9.4561	9.1163	6.0751	1
GSM411280	0	1	1	0	1	1	1	3	67			2.08	0			9.5321	11.9359	5.7134	12.3236	9.9885	8.7925	8.6072	4.8255	0
GSM411281	NA	1	1	0	0	0	0	2	74			2.11	1			10.0413	8.3683	4.3837	12.3319	10.5173	8.9798	8.4466	5.2448	0
GSM411282	0	1	1	0	0	1	1	2	57			3.42	1			9.5776	9.4126	4.9631	12.5564	10.2600	9.9738	8.9753	5.1546	0
GSM411283	1	1	1	1	1	1	0	2	60			2.09	0			12.6009	11.7324	4.9991	12.5373	10.1695	9.6857	8.4346	5.5771	1
GSM411284	0	1	0	0	1	1	1	2	65			5.01	1			9.9066	12.2607	7.8513	12.7366	11.1468	10.2414	9.9275	5.5486	1
GSM411285	0	1	1	0	1	1	0	2	41			2.11	0			9.7105	11.7435	7.7430	12.6940	10.8972	8.9843	9.7525	5.7898	1
GSM411286	0	1	1	0	1	1	1	2	46			4.69	1			8.8398	9.7564	6.6534	12.2491	9.9691	9.9777	8.4244	5.0821	0
GSM411287	0	1	0	0	1	0	0	2	65			4.29	0			9.4889	9.8066	4.0767	11.8815	9.9065	9.0894	8.3822	5.0747	0
GSM411288	0	1	0	0	1	0	1	3	46			4.06	1			9.9800	11.4517	3.8234	12.2503	9.4381	9.0452	8.7620	5.9783	1
GSM411289	NA	1	0	1	1	0	0	3	66			3.58	0			12.6950	11.1321	3.4129	12.7196	11.3234	9.5999	8.5327	5.2078	1
GSM411290	0			0	0	0	0	3								8.6601	8.2457	4.1092	12.2811	10.0342	8.3727	8.6036	4.8428	0
GSM411291	NA			1	0	0	1	3								13.0829	6.2194	3.9944	12.5034	9.5481	10.0984	8.4844	5.5800	1
GSM411292	1		0	1	0	0	1	3		2.80	0			2.80	0	12.9494	6.5083	3.5814	12.0560	9.5744	9.9381	9.4306	4.6361	0
GSM411293	1		0	1	0	1	1	3		1.85	0			1.85	0	11.5533	7.8965	4.8284	12.4888	10.6198	8.5887	9.2975	5.3817	0
GSM411294	1		0	1	0	0	0	3		2.32	0			2.32	0	12.9012	6.7834	3.3394	12.3930	10.8791	9.7816	9.3271	7.7600	1
GSM411295	1		0	1	0	0	1	3		5.08	0			5.08	0	13.2413	7.2554	3.7137	12.3059	10.4124	8.5877	9.4165	4.7473	0
GSM411296	0			0	0	0	1	3								9.3539	4.9369	3.7466	12.3754	10.4423	9.8596	9.4606	6.3124	1
GSM411297	0		0	0	0	0	0	3								9.6868	7.9302	4.4333	12.3075	10.4653	9.8574	9.5289	4.6282	0
GSM411298	1		0	1	0	0	1	3		3.85	0			3.85	0	13.4727	8.1939	4.2541	12.4778	10.7037	8.5443	10.4700	5.4689	0
GSM411299	1		0	1	0	0	1	3		4.71	0			4.71	0	13.8006	7.8116	4.3167	12.9374	11.4571	9.3801	9.8677	5.5458	1
GSM411300	1		0	1	0	0	0	3		2.96	0			2.96	0	13.4104	6.7807	3.6609	12.3206	9.8652	8.4801	8.8746	4.6599	0
GSM411301	1		0	1	0	1	0	3		2.70	0			2.70	0	12.1114	9.0412	5.1110	12.4504	10.4111	10.0036	9.3946	4.5182	0
GSM411302	1		0	1	0	0	0	2		4.72	0			4.72	0	13.9287	7.8810	3.8676	12.0841	10.4251	9.3542	8.6524	5.2364	0
GSM411303	0		0	0	0	0	1	3		0.88	1			0.88	1	9.3683	6.6693	4.0873	11.7585	10.6495	10.2701	9.5128	5.6009	1
GSM411304	1		0	1	1	0	1	2		1.44	0			1.03	1	13.4016	9.6450	3.6584	12.8010	10.4209	9.2007	8.7140	6.2623	1
GSM411305	1		0	1	0	0	1	3		2.18	0			2.18	0	13.3404	7.6860	3.8245	12.4037	9.9797	8.9211	8.5120	5.7019	1
GSM411306	0		0	0	1	1	1	3		4.73	0			4.73	0	9.7426	10.8521	4.9525	11.9611	10.1198	9.0753	9.9798	5.9036	1
GSM411307	1		0	1	0	0	1	3		3.18	0			3.18	0	13.5201	9.1691	4.2604	12.0741	10.0573	8.8242	8.7579	4.7567	0
GSM411308	0		0	0	0	0	0	3		2.12	0			2.12	0	8.9978	6.5954	3.8437	12.4136	9.9681	9.7221	9.4258	6.3875	1
GSM411309	0		0	0	0	1	1	3		2.35	1			0.80	1	9.5475	7.9577	5.1591	12.3815	11.1907	9.0231	8.7003	6.0291	1
GSM411310	0		0	0	0	0	1	3		3.70	0			3.70	0	8.8291	7.3733	4.5762	12.0516	10.5375	9.9994	8.3423	4.9994	0
GSM411311	0		0	0	0	0	1	3		3.66	0			3.66	0	8.6462	8.7436	4.4684	12.0758	10.3961	9.0984	8.9657	4.2030	0
GSM411312	0		0	0	0	0	1	3		2.11	0			2.11	0	9.0264	8.5749	3.7271	12.1807	9.4683	9.5540	9.1832	6.5433	1
GSM411313	0		0	0	0	0	0	3		3.15	0			3.15	0	9.6163	6.1084	3.5060	12.1668	9.9513	8.6217	8.5123	4.9155	0
GSM411314	0		0	0	0	0	1	2		3.45	0			3.45	0	9.3004	8.2594	3.7676	12.2115	10.0709	9.2381	8.3229	4.2396	0
GSM411315	0		0	0	0	0	0	2								10.4215	9.0967	4.2754	13.0497	11.0642	9.5324	9.2367	4.7525	1
GSM411316	0		0	1	0	0	1			5.83	0			1.52	1	12.9000	8.2566	4.4399	12.4429	10.1981	9.2286	8.5388	4.7745	0
GSM411317	0		0	0	0	0	1	3		1.27	1			1.27	1	10.2233	7.9736	4.0807	12.1103	10.2457	9.2431	9.1369	4.5839	0
GSM411318	0		0	0	0	0	0	3		2.05	0			2.05	0	9.0370	6.4197	3.7470	12.1261	9.6460	8.6088	9.7597	7.0298	1
GSM411319	0		0	0	0	0	1	3		3.21	0			1.59	1	9.2036	8.4364	3.6774	12.3134	10.6812	9.7038	8.9950	6.0749	1
GSM411320	0		0	0	0	0	0	2		3.77	0			3.77	0	9.4467	7.5178	4.5162	12.3396	10.4142	8.7156	9.4387	4.9488	0
GSM411321	1		0	1	0	0	1	3		2.79	0			2.79	0	12.1231	7.1182	3.0856	12.4857	10.2561	9.9713	8.6091	4.7779	0
GSM411322	0		0	0	0	0	1	3		2.13	0			2.13	0	8.3768	6.1547	3.8222	12.5919	9.3472	9.3478	7.6087	5.5145	0
GSM411323	0		0	0	0	0	1	2		4.85	0			4.85	0	9.2682	9.3887	3.6165	12.6460	10.9979	9.8672	8.9178	4.9472	1
GSM411324	0		0	0	1	0	1	3		3.72	0			3.72	0	8.5074	9.6557	4.0277	12.3678	11.0012	9.4646	9.2233	5.5698	1
GSM411325	0		0	0	0	0	1	2		1.99	0			1.99	0	8.8552	6.8175	3.8906	11.7914	9.4813	8.6842	7.8849	6.0747	1
GSM411326	0		0	0	0	0	1	3		1.95	0			1.95	0	8.9071	8.3097	3.8785	12.4887	10.7440	9.3780	9.9051	6.4359	1
GSM411327	0		0	0	0	0	0	3								8.4082	7.2847	4.3725	12.4877	10.3811	8.5255	9.6767	5.2540	0
GSM411328	0		0	0	0	0	0	3		2.58	0			2.58	0	8.7785	8.2733	3.9457	12.2187	9.9761	9.8113	9.0690	5.4741	0
GSM411329	0		0	0	0	0	1	3		2.15	0			0.68	1	10.3408	8.2509	3.4706	12.3714	10.3734	9.1729	9.0685	6.4732	1
GSM411330	1		0	1	0	0	0	2		4.35	0			4.35	0	13.2346	6.5709	3.3355	12.7307	10.4247	9.7340	8.4774	4.9190	0
GSM411331	0		0	0	0	0	0	3		3.32	0			1.72	1	9.0727	6.8545	3.5494	12.8697	10.5665	9.7271	9.2830	5.5420	1
GSM411332	0		0	0	0	1	1	3		1.90	0			1.90	0	8.6108	8.8390	5.0043	12.6479	9.9912	8.3631	9.1078	6.4365	1
GSM411333	0		0	0	0	0	0	3		2.85	0			2.85	0	8.6256	6.5171	3.0978	12.2689	10.1792	9.6716	9.1983	5.3136	0
GSM411334	0		0	0	0	0	0	3		2.66	1			1.05	1	8.8739	7.1065	3.9173	12.1468	10.4770	9.4607	9.2521	5.8318	1
GSM411335	0		0	0	0	0	0	3		2.50	0			2.50	0	8.7945	7.5917	4.0049	12.6733	9.0285	8.8007	9.0787	4.3924	0
GSM411336	0		0	0	1	0	0			5.79	0			5.79	0	9.5373	9.7949	4.3538	12.1322	10.2585	9.3709	9.7771	4.9742	0
GSM411337	0		0	0	0	0	0	3		5.29	1			3.87	1	8.7943	7.0652	4.3455	12.3325	9.9025	9.8030	8.4867	4.6879	0
GSM411338	1		0	1	0	0	0	3		2.76	0			2.76	0	14.1312	6.3564	3.7664	12.6317	10.4279	9.7011	8.8535	4.5505	0
GSM411339	0		0	0	0	0	1	2		2.01	1			2.01	1	8.5886	7.0533	3.8957	11.9730	10.3338	9.3003	8.9300	5.9072	1
GSM411340	0		0	0	0	0	1	3								8.2778	6.2467	3.0404	12.1320	9.3254	8.9756	8.6378	5.3041	0
GSM411341	0		0	0	0	0	0	2		4.60	0			4.60	0	8.7237	7.0105	4.0793	12.1327	9.8039	9.0241	8.4959	4.5619	0
GSM411342	0		0	0	0	0	0	3		3.74	0			3.74	0	8.2252	9.3356	4.8086	12.4132	10.5158	8.4603	9.9260	4.8146	0
GSM411343	0		0	0	0	0	0	2		2.62	0			2.62	0	9.1910	8.3338	4.5116	12.0187	10.1842	9.5605	8.5625	5.1282	0
GSM411344	0		0	0	0	0	1	3		4.31	0			4.31	0	8.8011	9.2287	4.4496	12.2114	10.8854	9.1096	9.8423	4.8796	0

GSM411345	0		0	0	0	0	1	1	1.64	0	1.64	0	8.6109	7.5244	4.1731	12.6982	10.9271	9.2568	9.4105	5.5496	0
GSM411346	0		0	0	0	0	0	3	3.85	0	3.85	0	9.0405	9.0960	4.7220	12.5085	10.2323	9.1474	8.6797	5.2733	0
GSM411347	0		0	0	0	0	0	3	1.18	1	0.61	1	9.0265	6.0903	3.5860	12.6973	9.8857	9.1989	10.0385	5.6447	1
GSM411348	0		0	0	0	0	1	3	3.86	0	3.86	0	8.8649	8.1898	4.6392	12.3987	10.9098	10.9389	9.1481	5.4662	0
GSM411349	0		0	1	0	0	0	2	3.14	1	3.14	1	11.7572	9.0200	4.0763	12.2094	10.3290	8.9373	9.0669	4.2183	0
GSM411350	0		0	0	0	0	0	3					9.4155	9.1301	4.6983	12.1842	10.5175	9.0174	9.0602	6.2323	1
GSM411351	1		0	1	0	0	1	3	5.99	0	0.70	1	13.9328	8.3764	4.2699	12.3067	10.2341	8.5523	8.9294	5.0589	0
GSM411352	0		0	0	0	0	0	3	1.83	0	1.83	0	9.3436	9.4729	3.8521	12.1354	9.8709	8.3956	9.0720	6.1143	1
GSM411353	0		0	0	0	0	0	3	5.04	0	5.04	0	9.6882	8.0313	4.6753	12.2522	10.6021	9.8124	10.0670	5.0120	0
GSM411354	0		0	0	0	0	1	3	0.86	1	0.86	1	8.4288	5.4810	3.8458	12.9003	11.2741	8.6924	9.8789	5.6206	1
GSM411355	0		0	0	0	0	0	3	5.02	1	5.02	1	8.7577	9.2648	4.2214	12.5519	10.8619	8.7780	9.0849	4.1756	0
GSM411356	0		0	0	0	0	0	2	5.92	0	5.92	0	9.2891	8.0213	3.7935	12.2890	10.8102	9.5261	9.4098	4.7188	0
GSM411357	0		0	0	0	0	1	3	1.08	0	1.08	0	8.5835	6.3306	3.3519	11.9704	9.9356	9.7798	9.0813	5.7788	1
GSM411358	1		0	1	0	0	0	2	2.05	1	0.94	1	12.0308	8.0752	4.0720	12.2292	10.8668	9.2436	9.7488	5.0648	0
GSM411359	0		0	0	0	0	1	3	3.81	0	3.81	0	9.1060	7.4294	4.0452	12.2499	11.0469	8.7480	8.1943	6.0169	1
GSM411360	0		0	0	0	0	1	3					9.8219	8.0402	4.6694	12.6660	10.2938	8.5885	9.0534	4.9198	0
GSM411361	0		0	0	0	0	0	3	1.73	1	1.04	1	8.7238	8.3020	3.9478	12.7023	10.8928	9.9081	9.2852	5.5158	0
GSM411362	1		0	0	0	0	0	2	4.18	0	2.94	1	10.1697	7.8292	4.6820	12.4588	9.9969	9.3260	8.5391	5.5045	0
GSM411363	1		0	1	0	0	0	3	2.36	0	2.36	0	14.0371	4.1183	3.5477	12.6452	9.3630	10.2069	9.5104	6.0031	1
GSM411364	1			0	0	0	0	3					9.3391	9.0951	4.3439	12.6601	10.1637	9.5741	9.4272	5.0815	0
GSM411365	1		0	1	0	0	0	3	4.51	0	4.51	0	11.5800	8.0108	3.5974	12.3222	9.8460	9.1159	8.9018	5.1914	0
GSM411366	1		0	1	0	0	1	3	2.72	0	2.72	0	13.4819	8.2792	4.3221	12.4217	9.8958	8.8663	8.6297	4.9069	0
GSM411367	1			1	1	1	1						11.6981	13.8736	5.4429	12.2934	10.7937	9.5174	9.0351	4.9465	0
GSM411368	0		0	0	0	0	1	3	1.75	0	1.75	0	8.2661	7.2558	4.2750	12.5620	10.0524	8.9544	9.4301	4.4496	0
GSM411369	NA		0	1	0	0	1	3	5.67	0	5.67	0	13.3894	7.6723	3.6501	12.4357	10.5331	10.5996	8.6380	5.4970	0
GSM411370	NA		0	0	0	0	0	3	4.58	0	4.58	0	9.4764	6.1618	3.9161	12.2077	10.3367	8.7940	8.5327	5.1983	0
GSM411371	NA		0	0	0	0	1	3	5.07	0	5.07	0	9.0582	7.8329	4.2194	12.4790	11.1610	9.1951	9.9386	5.3005	1
GSM411372	NA		0	0	0	0	1	3	2.63	0	2.63	0	9.6703	7.7721	3.7793	12.1182	9.9267	9.2284	8.4288	5.5798	1
GSM411373	NA		0	0	0	0	0	3	1.50	1	0.68	1	8.1046	7.8804	4.5949	12.7023	10.3641	8.9306	8.7104	4.5633	0
GSM411374	NA		0	0	0	0	1	3	2.02	0	0.44	1	8.9110	8.7690	4.2622	12.3320	10.2069	9.3914	8.6724	4.7739	0
GSM411375	NA		0	0	0	0	0	3	2.81	0	2.81	0	9.5248	4.2241	2.6621	11.9988	10.1093	9.8825	8.7796	5.6392	1
GSM411376	NA		0	0	0	0	1	3	2.52	0	2.52	0	10.6837	7.0524	4.0026	11.8594	10.3141	9.8485	9.4957	5.4773	0
GSM411377	NA		0	0	1	1	1	3	1.99	0	1.53	1	9.3270	10.1309	5.0500	12.6685	10.8595	8.3349	9.3679	6.0031	1
GSM411378	NA		0	0	0	0	0	3	3.59	0	3.59	0	9.7537	7.6313	4.1901	11.9104	9.9930	8.7718	8.7261	4.8072	0
GSM411379	NA		0	0	0	0	0	3	1.23	0	1.23	0	9.4858	6.6020	3.7786	12.4127	9.5660	8.9016	8.5721	4.6156	0
GSM411380	NA		0	0	0	0	0	3	2.25	0	2.25	0	9.2348	6.1274	3.9408	12.4737	10.1739	8.9360	8.4805	5.6123	1
GSM411381	NA		0	0	0	0	1	3	3.66	0	3.66	0	9.1468	6.0363	3.3694	12.3281	10.7399	9.9021	9.6893	5.0843	0
GSM411382	NA		0	0	0	0	0	3	3.31	0	3.31	0	9.0389	7.7586	3.7246	12.2209	10.2712	9.3052	9.0464	4.7254	0
GSM411383	NA		0	0	0	0	1	2	3.78	0	3.78	0	9.9202	8.3636	3.6916	12.4503	10.0858	10.1021	8.5782	4.6853	0
GSM411384	NA		0	0	0	0	1	3	3.08	0	3.08	0	9.5825	7.0491	3.5439	12.1499	10.4454	9.8867	8.3918	4.6487	0
GSM411385	NA		0	0	0	0	0	1	2.58	0	2.58	0	9.8063	5.8964	3.3989	12.7331	11.0038	10.1118	8.8109	4.8502	1
GSM411386	NA		0	0	0	0	1	3	2.68	0	2.68	0	8.5622	7.4037	3.5405	12.4533	10.2184	9.9626	9.0615	4.6223	0
GSM411387	NA		0	0	0	0	1	3	0.98	1	0.17	1	9.2964	5.8699	3.7856	12.6089	9.2918	8.6076	8.5756	4.3703	0
GSM411388	NA		0	0	0	0	0	2	1.76	0	1.76	0	9.4939	8.4467	3.9579	12.0523	9.9362	8.3295	8.1814	4.7927	0
GSM411389	NA		0	0	0	0	1	3	1.62	1	1.61	1	8.3159	7.2990	4.2549	12.0881	9.4169	9.2559	7.7304	4.6828	0
GSM411390	NA		0	1	0	0	1	3	5.62	0	5.62	0	13.0556	7.8821	4.6414	12.3863	10.1372	8.4056	10.1256	4.6545	0
GSM411391	NA		0	0	0	0	0		5.63	0	5.63	0	10.7476	7.2844	4.4232	12.5666	10.6938	10.1893	10.2236	5.3495	0
GSM411392	NA		0	0	0	0	1		0.38	0	0.38	0	8.3426	4.6019	3.0642	12.2917	10.3804	10.3442	8.8863	5.1007	0
GSM411393	1		1	1	0	0	1	2	2.65	0	2.65	0	13.2138	8.8705	3.5405	12.4902	10.0278	9.3569	8.0214	4.8214	0
GSM411394	0		1	0	0	1	0	3	5.47	0	5.47	0	10.0109	7.0260	5.0296	12.5833	11.5056	9.6334	9.9012	4.7059	1
GSM411395	1		1	1	0	0	1	2	5.47	0	5.47	0	13.5988	6.3557	3.9692	12.9123	10.9118	10.3549	9.3913	5.6648	1
GSM411396	0		1	0	0	0	1	3	3.31	0	3.31	0	10.6627	5.9674	3.3852	12.8197	9.8789	9.4407	9.6878	5.4023	1
GSM411397	0		1	0	0	1	1	3	4.88	0	4.88	0	9.1908	9.3167	4.8880	12.3949	11.1540	8.8806	9.9947	5.2640	1
GSM411398	0		1	0	0	0	0	3	3.10	0	3.10	0	8.2892	5.6995	3.3128	12.3982	10.2039	9.0144	9.3706	5.1161	0
GSM411399	0			0	1	0	0	3					9.7683	13.4711	4.6057	12.3138	9.7075	8.6426	8.7047	4.8696	0
GSM411400	0		1	0	1	0	1	3	4.79	0	4.79	0	8.5225	9.5955	4.3088	12.4977	10.9815	10.2629	10.0140	5.2116	1
GSM411401	0		1	0	0	0	0	3	5.53	0	5.53	0	8.9336	7.9742	4.5804	12.3218	10.2615	9.3615	9.1862	4.8311	0
GSM411402	1		1	1	0	0	0	2	5.51	0	5.51	0	11.2845	6.0178	3.8138	12.2202	10.8243	9.6024	10.1325	5.4509	0
GSM411403	1		1	0	0	0	1	3	1.74	0	1.74	0	9.6619	8.1427	3.5297	12.3912	10.2012	8.7773	9.1332	6.5559	1
GSM411404	1		1	1	0	0	1	3	3.58	0	3.58	0	11.6692	9.4531	3.6852	12.5961	10.2760	8.5554	8.6788	5.1491	0
GSM411405	1		1	1	0	0	1		5.72	0	5.72	0	13.9486	6.9463	3.9486	12.3145	10.6165	10.3887	9.0985	5.5664	0
GSM411406	1		1	1	1	0	1	3	3.10	0	3.10	0	12.8628	9.8517	3.5861	11.9533	10.0488	9.7281	8.6735	5.5767	1
GSM411407	1		1	1	0	1	0	3	1.70	0	1.70	0	13.1719	8.9544	4.9264	12.6911	10.5841	9.3160	9.1704	5.3025	0
GSM411408	NA		1	0	0	0	0	3	3.58	0	3.58	0	8.5659	7.5321	3.4773	12.2105	9.8860	8.6632	8.8188	4.5964	0
GSM411409	NA		1	0	0	0	1	3					8.2234	7.3167	3.8092	12.1813	9.2300	9.4600	8.3727	4.4373	0
GSM441624		1		0	0	0	0				7.58	0	9.5194	9.0096	4.4766	12.6741	10.7304	8.6187	8.9634	5.8179	1
GSM441625		1		1	1	1	0				2.68	0	11.4476	9.7885	4.9319	12.8276	10.4256	8.8211	8.4531	5.1612	1
GSM441626		1		0	0	0	0				7.74	0	9.1330	8.5285	3.2394	12.5415	10.3256	9.0692	8.8187	5.4172	0
GSM441627		1		0	1	0	0				6.21	1	9.5092	11.8407	4.5404	12.3754	10.5272	8.5417	8.6385	5.5323	0
GSM441628		1		0	1	0	0				3.72	1	9.5896	11.3672	3.9866	13.0335	11.5299	9.2498	8.7842	5.7597	1
GSM441629		1		0	0	0	1				2.29	1	9.7304	8.6284	3.6153	13.4659	11.6492	8.9490	9.0462	5.9258	1
GSM441630		1		0	1	1	0				7.36	0	9.4848	10.4072	7.0163	12.6273	10.5788	8.2903	9.1584	5.6125	1
GSM441631		1		0	1	0	1				7.28	0	8.9803	10.5330	4.2792	12.2540	10.1884	8.9875	9.2823	5.4959	0
GSM441632		1		0	1	0	0				5.58	0	8.9106	10.5653	3.6989	12.0401	10.0773	9.2528	9.3224	5.3009	0
GSM441633		1		0	1	0					7.79	0	9.7630	10.4745	4.4009	12.4953	10.2190	8.8252	9.1050	5.2303	0
GSM441634		1		0	1	0	1				5.11	0	9.6701	11.6025	4.0898	12.1248	10.2940	8.3242	9.2011	5.9768	1
GSM441635		1		0	1	1	0				5.16	0	8.5857	12.2634	5.2373	12.0015	9.5563	8.6064	8.8114	5.3156	0
GSM441636		1		0	1	0	0				4.49	0	9.6694	11.7854	3.9544	12.3185	9.7228	8.7526	8.8646	5.0256	0
GSM441637		1		0	0	0	0				8.00	0	10.2998	9.4160	3.6950	10.9597	10.9974	7.2722	9.1723	5.5480	1
GSM441638		1		0	0	0	0				6.25	0	9.3806	8.7823	4.5787	12.2984	10.1998	8.6072	8.9315	5.4740	0
GSM441639		1		0	1	0	0				4.12	0	9.5301	9.5290	4.3639	12.5105	10.4986	7.9391	8.9803	5.6564	1
GSM441640		1		0	0	0	0				6.93	0	9.2001	9.1893	3.8171	12.2945	10.0316	8.3661	8.5366	5.1555	0
GSM441641		1		0	1	1	0				7.19	0	9.1110	9.6603	6.1985	12.3321	10.2746	8.3657	8.9253	5.1681	0
GSM441642		1		0	1	0	1				7.04	0	10.1536	11.8767	4.7954	12.3923	10.5377	9.2105	9.3440	4.8698	0
GSM441643		1		0	1	0	0				1.98	1	10.6045	10.5057	3.3933	12.5945	10.7017	7.9146	8.8765	5.5578	0
GSM441644		1		0	1	1	1				1.96	1	9.3946	11.1362	4.9599	12.3487	9.7605	8.5427	8.6260	5.4958	0
GSM441645		1		0	1	0	0				4.03	0	9.1261	12.0974	4.3589	12.0608	9.7500	8.9700	8.5259	5.3326	0
GSM441646		1		0	1	0	1				7.92	0	10.3562	10.2522	3.7618	12.3151	10.1559	7.8635	8.8903	5.1504	0
GSM441647		1		0	1	1	0				3.99	0	9.3260	11.1837	7.2904	12.6970	10.2374	9.2336	9.5105	4.9931	0
GSM441648		1		0	0	0	0				3.73	0	9.9511	9.1010	4.7095	12.3395	10.4539	9.9471	8.2862	5.4251	0
GSM441649		1		0	1	0	0				6.86	0	9.2325	11.4302	4.1119	12.9373	10.4124	9.3981	9.0476	5.4563	1
GSM441650		1		0	1	1	0				7.01	0	9.5109	11.8731	5.3435	12.3756	10.4151	9.3097	9.3408	4.9702	0
GSM441651		1		0	1	0	0				5.74	0	9.1874	11.5459	4.7490	12.9624	10.9982	8.7134	9.3557	5.1426	1
GSM441652		1		0	0	1	0				5.97	0	9.7136	9.0121	6.9731	12.4214	10.1651	7.0752	9.3410	5.4384	0
GSM441653		1		0	0	1	0				5.00	0	9.3445	9.4446	5.7611	12.3378	10.3966	8.2826	8.6278	5.2926	0
GSM441654		1		0	1	0	1				2.86	0	9.5503	9.9148	3.9406	12.4927	10.1820	8.5613	8.6614	5.3443	0
GSM441655		1		0	1	0	1				1.35	0	9.8297	10.3424	4.4487	12.6826	9.8094	8.6287	9.1723	5.4491	0
GSM441656		1		0	1	0	1				2.60	0	9.6563	12.7465	4.4153	12.1431	10.6268	8.0933	10.4456	5.2277	0
GSM441657		1		0	1	1	1				3.63	1	9.2621	10.5583	5.2513	12.6304	10.4103	9.4190	9.1069	5.3391	0
GSM441658		1		0	1	0	0				4.82	0	9.4232	10.8412	3.9193	12.2076	10.4715	8.7413	8.5020	5.5200	0
GSM441659		1		0	1	0	0				4.94	0	9.7518	12.6955	4.1323	12.7588	10.7985	9.0009	9.0447	5.4832	0
GSM441660		1		0	1	0	1				4.99	0	8.8193	10.8540	3.5949	12.5920	9.6100	7.9174	8.3549	5.3097	0
GSM441661		1		0	1	1	0				5.36	0	9.0309	11.5742	5.4983	12.2653	10.4270	9.1201	8.9687	5.3040	0
GSM441662		1		0	1	0	0				4.95	0	10.2304	12.3821	4.3711	12.6077	10.3826	8.4143	8.6998	5.8230	1
GSM441663		1		0	1	0	0				3.78	1	10.6268	9.8782	3.6545	12.0215	10.3662	9.3916	8.8496	5.6938	1

GSM441664	1	0	1	0	0	3.31	0	8.8139	11.0345	4.7579	12.5255	10.0719	9.1712	8.7372	5.2650	0
GSM441665	1	0	1	0	1	4.95	0	9.3808	11.7591	4.6640	12.1969	9.7261	8.2133	8.3388	5.4397	0
GSM441666	1	0	1	1	0	5.86	0	9.2725	11.6036	5.3168	12.5671	10.3523	8.7698	8.8979	5.5372	0
GSM441667	1	0	0	0	0	5.16	0	9.7000	9.1566	4.3411	12.3422	10.3197	8.6249	8.8963	5.3564	0
GSM441668	1	0	0	0	0	5.18	0	10.0443	8.4506	4.0240	12.4251	10.6582	8.7843	8.4833	5.7047	1
GSM441669	1	0	1	0	0	5.65	0	9.5021	9.6679	3.6716	11.8727	9.9388	8.6517	8.3473	5.4544	0
GSM441670	1	0	1	0	0	5.48	0	8.7440	10.1616	3.7450	12.4237	10.6397	8.2536	8.6866	5.4429	0
GSM441671	1	0	1	0	0	4.57	0	10.2945	9.8511	4.3520	12.2381	10.1295	8.8754	8.7060	5.3001	0
GSM441672	1	1	1	0	1	1.74	1	11.9013	11.6413	4.3036	12.1871	10.5481	8.7044	8.7665	5.4465	0
GSM441673	1	0	1	0		4.89	0	8.9632	10.1716	3.9516	12.3207	10.7008	9.4858	8.5777	5.3468	0
GSM441674	1	0	1	0	1	2.53	0	9.3759	9.7991	4.4232	12.4487	10.0945	8.5857	8.6574	5.3890	0
GSM441675	1	0	1	0	1	0.71	0	8.7997	12.1614	4.2461	12.6005	10.7539	8.7548	8.6520	5.5696	1
GSM441676	1	0	1	1	0	4.53	0	9.3839	12.2998	7.4052	12.4630	10.5798	9.0715	8.6869	5.5807	1
GSM441677	1	0	1	0	0	0.50	1	8.2267	11.6429	4.6174	12.3265	9.9574	8.8986	8.6402	5.3719	0
GSM441678	1	0	1	1	0	2.97	0	9.1829	11.5228	8.2555	12.5369	10.2847	8.8503	8.7416	5.5596	0
GSM441679	1	0	1	0		2.33	0	9.8680	11.1066	4.4430	12.5117	10.4778	8.6333	9.6226	4.9171	0
GSM441680	1	0	1	0	1	0.62	0	10.5395	10.3384	4.0624	12.6248	10.7769	8.5330	8.8542	5.1660	0
GSM441681	1	0	1	0	1	4.55	0	10.4197	11.3421	3.8655	13.2662	11.5515	10.0855	8.3931	5.9866	1
GSM441682	1	0	1	0	0	3.88	0	9.9766	9.8173	3.5882	12.6894	10.7219	9.0887	8.7249	5.2430	0
GSM441683	1	0	1	1	1	4.49	0	9.8253	11.9141	7.0817	12.7470	10.1284	9.3058	8.2178	5.6637	1
GSM441684	1	0	0	0	0	9.88	0	10.9694	7.4973	3.6722	12.3531	10.0971	8.7638	8.0817	5.2304	0
GSM441685	1	0	1	0	1	10.49	0	9.6838	9.5905	3.5706	12.8331	11.3691	7.8674	9.4013	5.7279	1
GSM441686	1	1	1	0	1	9.35	0	12.5332	10.8509	3.9660	12.0805	9.7576	8.3984	8.8463	5.6317	1
GSM441687	1	0	1	0		10.58	0	9.5649	10.7799	4.1826	12.1791	9.6499	7.3613	8.5800	5.1820	0
GSM441688	1	0	1	0	0	10.16	0	8.9688	11.3758	4.7262	11.9931	9.2654	8.0861	9.0685	5.4903	0
GSM441689	1	0	0	0	0	3.77	1	9.1008	8.6925	3.4712	12.0902	10.3773	8.6187	8.6097	5.4434	0
GSM441690	1	0	1	1	0	4.53	1	8.8384	10.0614	5.1929	12.0955	9.6411	7.8128	9.2252	5.1272	0
GSM441691	1	0	1	0	1	5.67	1	10.2042	12.1994	4.3881	12.3246	10.2947	8.4178	8.9173	5.1738	0
GSM441692	1	0	1	1	1	7.12	1	9.2689	11.1494	7.5089	12.3663	11.2102	9.2142	9.0775	5.2375	1
GSM441693	1	0	1	0	0	5.32	0	8.6998	11.4755	4.5616	13.1391	9.9910	8.5458	9.0398	4.9965	1
GSM441694	1	0	1	0	1	5.19	0	9.1452	13.6350	4.6988	12.0230	11.1614	8.1110	9.0345	5.2283	1
GSM441695	1	0	1	1	0	9.80	0	9.3636	11.2176	6.7973	12.3841	10.6261	8.8914	8.7769	5.7601	1
GSM441696	1	0	1	0	1	12.75	0	9.3765	11.0491	4.5593	12.2407	9.6411	8.0859	8.8271	5.1047	0
GSM441697	1	1	0	0	0	9.78	0	12.1540	7.0277	3.7733	12.4478	10.5925	8.8574	8.5844	5.4577	0
GSM441698	1	0	1	1	0	9.58	0	9.7739	11.7825	7.2914	12.1227	10.7993	9.7965	9.3009	5.3804	0
GSM441699	1	0	1	0	0	1.55	1	10.6692	11.2904	4.0457	13.0821	11.4177	9.0327	9.2118	6.3414	1
GSM441700	1	1	1	0	0	2.57	1	12.0758	11.3323	4.6796	12.6475	10.7524	8.7325	8.5331	5.5966	1
GSM441701	1	0	0	1	0	2.74	0	9.5680	8.6591	6.2192	11.9114	10.1412	8.2175	8.8303	5.3599	0
GSM441702	1	0	1	1	1	10.29	0	9.7035	11.6284	4.9303	12.2624	11.0471	8.4019	9.0096	5.6556	1
GSM441703	1	0	1	1	0	9.41	0	9.4968	12.0654	5.3762	12.2055	10.8041	8.6714	9.3014	5.2904	0
GSM441704	1	0	1	1	0	11.05	0	9.6360	11.4882	7.2941	12.0592	9.9813	7.9345	9.7075	5.2938	0
GSM441705	1	0	1	0	1	6.50	1	9.6924	11.7869	4.0613	12.6746	11.0309	9.6048	8.8672	5.7711	1
GSM441706	1	0	1	0	0	10.38	0	8.7525	10.2166	4.1334	12.7438	11.1958	8.9611	9.1257	5.1489	1
GSM441707	1	0	1	1	0	8.59	0	8.7610	11.4063	6.4954	12.3812	10.8487	9.5001	8.7514	5.1536	0
GSM441708	1	0	1	1	1	10.01	0	8.6237	11.9478	5.5063	12.6219	11.0647	9.4162	9.1172	4.9692	1
GSM441709	1	0	1	1	0	9.88	0	8.1093	9.6782	4.9847	11.8861	10.0206	8.1425	8.4381	4.9041	0
GSM441710	1	0	0	0	1	2.67	1	9.1072	9.0956	3.5019	12.6112	10.0688	8.4630	9.3464	5.5918	1
GSM441711	1	0	1	0	0	8.10	0	9.4116	10.4427	4.5693	12.3886	10.7819	9.1432	8.9509	5.6214	1
GSM441712	1	0	1	0	1	8.88	0	9.7797	10.0324	4.3340	12.3551	10.0638	8.4917	8.6644	5.7710	1
GSM441713	1	0	1	1	1	8.81	0	10.0974	11.0056	5.6264	12.2860	10.4109	8.0372	8.4431	5.5862	1
GSM441714	1	0	0	1	0	4.35	0	8.9841	8.3493	5.3853	12.2299	10.2299	10.3599	9.4073	5.4545	0
GSM441715	1	1	1	0	0	1.35	1	11.6967	9.8844	4.0250	12.4517	10.5960	9.3277	8.3241	5.1192	0
GSM441716	1	0	1	0	1	6.04	0	9.9485	9.9536	3.6667	12.3749	10.8155	9.0981	9.0987	5.3888	0
GSM441717	1	0	1	1	0	5.46	0	9.0705	9.5641	5.8806	12.1351	10.2188	9.6530	9.2667	5.1533	0
GSM441718	1	0	1	1		4.11	0	9.2049	11.1373	5.8727	12.5443	10.1807	9.7089	9.5224	5.0314	0
GSM441719	1	0	0	1	0	9.43	0	8.7368	9.2147	5.0533	11.9600	9.7290	9.9208	8.5201	4.9155	0
GSM441720	1	0	0	0	0	2.59	0	9.4453	9.2387	4.0417	12.0816	10.1319	9.4082	8.6192	4.9559	0
GSM441721	1	0	1	1	0	4.15	0	9.6025	11.0294	7.0883	12.0251	9.7643	9.7490	8.5624	5.5236	0
GSM441722	1	1	0	0	1	8.24	0	13.1189	8.4955	3.9637	12.7679	10.2019	8.0181	8.0047	5.5235	0
GSM441723	1	0	1	1	0	8.09	0	9.8358	10.0260	6.3958	12.1589	9.3970	7.9123	8.3930	5.0870	0
GSM441724	1	0	0	0	0	8.00	0	10.6063	8.1987	4.3678	12.3275	9.9934	8.5701	8.2892	5.1820	0
GSM441725	1	0	0	0	0	1.55	0	9.8192	8.4311	4.6178	12.1575	10.3100	8.4403	8.6045	5.5287	0
GSM441726	1	0	1	0		8.11	0	9.9283	10.0021	3.6959	11.9239	9.8008	8.2263	8.5002	5.4502	0
GSM441727	1	0	1	0	1	10.80	1	10.1265	10.8397	4.3699	12.2456	10.0611	9.4043	8.7221	5.0034	0
GSM441728	1	0	1	0	1	7.69	1	9.5544	11.2498	3.7770	12.7588	10.3610	9.4668	8.8320	4.9587	0
GSM441729	1	0	1	0	0	11.70	0	10.1390	10.9892	4.7010	12.9604	10.6894	9.6061	9.0540	4.7490	1
GSM441730	1	0	1	0	0	12.28	0	10.5365	10.6223	4.2469	12.4098	9.4438	8.9283	9.0547	4.9567	0
GSM441731	1	1	1	0	0	10.83	0	11.2586	10.8758	4.7414	12.9656	10.5446	9.2625	9.1644	5.3869	1
GSM441732	1	0	1	0	1	11.01	0	10.6137	10.8217	3.9257	12.6999	10.2007	9.1051	9.3575	5.2374	0
GSM441733	1	0	1	0	1	9.46	1	9.5178	9.8558	4.2766	12.5850	9.8103	8.8936	8.7383	4.8044	0
GSM441734	1	1	1	0	0	9.70	0	13.0440	11.3751	4.0724	13.4037	10.7765	9.6653	8.8440	4.9345	1
GSM441735	1	0	1	1	1	10.94	0	10.6389	11.4564	4.8561	12.6567	9.7589	9.1614	8.8230	4.7968	0
GSM441736	1	0	1	0	1	9.97	0	10.0933	10.6378	4.6492	12.2984	9.5710	8.8282	9.0991	4.9298	0
GSM441737	1	0	1	1	0	8.52	0	9.8899	10.2191	5.3881	12.7844	10.0872	9.7570	8.6494	5.2438	0
GSM441738	1	0	1	0	0	12.19	0	9.9966	10.3754	4.4161	12.5517	10.4416	9.8851	9.3671	5.1223	0
GSM441739	1	0	0	0	1	2.23	1	9.5859	8.1857	3.8213	12.1384	9.9929	8.8594	9.0718	5.0611	0
GSM441740	1	0	0	0	0	10.97	0	8.7128	8.0340	3.8734	12.4990	9.8279	8.8553	8.7336	5.1592	0
GSM441741	1	0	0	0	0	11.91	0	9.4465	8.4534	3.5498	12.0901	9.7454	8.6829	8.7414	4.8226	0
GSM441742	1	0	0	1	1	9.41	0	10.2085	8.6193	5.9410	13.1116	10.2708	8.9164	9.0407	5.2627	1
GSM441743	1	0	1	0	0	10.65	1	10.2520	10.6716	3.8318	13.8832	10.9332	8.2333	9.5791	4.9003	1
GSM441744	1	0	1	0	0	8.46	0	10.3064	10.2792	4.7575	11.1545	9.8190	8.9850	9.1709	4.9952	0
GSM441745	1	0	1	0	0	9.77	0	9.2964	9.7379	4.6154	12.2207	9.9829	9.8202	8.4527	4.7767	0
GSM441746	1	0	1	0	0	3.58	1	10.1623	9.7989	4.4725	12.9263	10.6521	9.3938	8.7782	5.0685	1
GSM441747	1	0	1	1	1	8.39	1	8.4147	10.6972	4.9166	13.0817	10.4226	8.8509	8.8095	4.8696	1
GSM441748	1	1	1	0	1	4.19	1	13.1771	9.8345	4.0571	12.8006	9.5045	9.6191	8.0909	5.1004	0
GSM441749	1	1	0	0	0	10.45	0	11.1824	7.2514	4.2778	12.4985	9.8851	9.5639	8.5566	4.9569	0
GSM441750	1	0	1	0	1	1.99	1	8.5895	11.3933	4.7599	12.7782	9.8816	9.6424	8.4018	5.1264	0
GSM441751	1	0	1	0	0	8.21	0	9.6340	10.2929	4.3690	13.3749	10.9531	9.1884	8.6946	4.8660	1
GSM441752	1	0	1	0	0	4.35	1	9.7162	10.8787	4.1090	12.0728	9.3498	8.3502	8.7507	4.7976	0
GSM441753	1	0	1	0	0	9.45	0	9.7294	10.9472	4.2728	11.6113	8.8720	8.2245	8.6550	4.8963	0
GSM441754	1	1	0	0	1	3.15	1	13.0502	8.5890	4.4717	12.1868	8.7371	8.2543	7.9439	4.5153	0
GSM441755	1	0	1	0	0	9.40	0	9.4848	10.1780	4.1321	11.8268	9.2829	9.4331	9.4154	5.5565	0
GSM441756	1	0	1	0	0	10.89	0	9.9502	11.3117	4.2249	12.8410	10.7007	8.8198	9.0686	4.8942	1
GSM441757	1	0	1	0	0	10.51	0	10.5820	9.5641	4.1906	11.9352	10.2254	9.2417	9.1629	5.1789	0
GSM441758	1	0	1	1	1	8.73	0	9.9957	11.0242	7.4513	12.0235	10.2259	9.3093	8.8070	4.8193	0
GSM441759	1	0	1	1	0	9.48	0	9.6664	9.5668	7.1716	12.3246	10.3103	9.9954	8.9046	5.0400	0
GSM441760	1	0	1	0	1	10.10	0	10.3777	10.4196	4.4650	10.9218	9.8316	6.3681	9.1008	4.6121	0
GSM441761	1	0	1	0	0	11.03	0	10.2316	11.1007	4.2711	13.2476	10.0828	9.0814	8.8238	5.0208	1
GSM441762	1	0	1	0	1	1.31	1	9.9171	10.5702	3.8364	12.7605	10.1272	9.4521	8.7710	5.0245	0
GSM441763	1	0	1	0	0	9.99	0	9.8922	9.7311	3.7438	13.1019	11.0208	9.4661	9.1074	4.9796	1
GSM441764	1	0	1	0	1	11.15	0	9.6875	11.0429	4.3809	12.4731	10.6155	8.9619	8.8768	4.8363	0
GSM441765	1	0	0	0	1	10.47	0	9.9969	8.7517	3.9433	12.3717	10.5831	8.9343	8.8955	4.8515	0
GSM441766	1	0	1	0	0	5.43	1	10.1465	10.4146	4.1891	12.6343	9.3330	8.4329	8.5494	5.0259	0
GSM441767	1	0	0	0	1	10.52	0	9.7341	7.2881	4.3133	12.2769	9.6640	8.8856	8.5866	4.7807	0
GSM441768	1	0	1	0	0	8.59	0	10.1574	10.4962	4.5147	12.1289	10.1562	8.5439	8.7793	4.7004	0

GSM441769	1	0	1	0	0	9.77	0	10.2653	11.0006	3.8612	12.2782	10.0222	8.1342	8.9941	4.9733	0
GSM441770	1	0	1	0	0	5.68	1	10.2193	10.8820	3.9630	11.7398	10.5654	9.3428	8.6364	5.5558	0
GSM441771	1	0	0	0	1	7.13	0	9.5430	9.1224	3.7806	11.4387	9.7836	7.8836	8.7416	5.4766	0
GSM441772	1	0	0	0	1	4.78	1	9.5277	9.4446	3.8024	12.3062	9.3614	8.2528	8.7311	5.1684	0
GSM441773	1	0	0	0	0	9.78	0	9.7427	7.9367	3.7655	12.0827	9.9236	8.9844	8.7486	5.2785	0
GSM441774	1	0	1	0	1	10.74	0	9.6063	11.5990	4.5260	11.8589	10.5916	9.1409	8.6569	5.1065	0
GSM441775	1	0	1	0	1	3.50	1	9.6517	11.8577	4.6991	11.6000	9.9191	7.5320	9.5864	4.9020	0
GSM441776	1	0	1	0	1	9.85	0	9.6971	10.9340	4.2512	12.7073	10.5577	8.7450	9.1279	5.0362	0
GSM441777	1	0	1	0	0	9.93	0	10.7926	10.2298	3.5906	12.6920	10.8910	9.9617	9.3552	6.1204	1
GSM441778	1	0	1	0	0	5.87	0	9.8305	11.0496	4.6564	13.2830	10.5502	10.5070	9.3919	5.8541	1
GSM441779	1	0	1	1	0	9.52	0	9.9164	11.1055	7.8049	12.9443	10.1301	10.4583	8.5628	5.5490	1
GSM441780	1	0	1	0	0	7.37	0	8.6421	11.5089	4.3185	11.9914	9.7029	10.1996	8.9129	5.3856	0
GSM441781	1	0	0	0	0	9.40	0	9.0998	9.0961	4.2101	12.4079	9.9007	9.7204	8.7938	5.6197	1
GSM441782	1	0	1	0	1	8.53	1	9.9903	10.7233	4.2880	12.3711	10.6716	9.8934	9.1754	5.6804	1
GSM441783	1	0	1	0	1	10.33	0	9.8596	10.2616	3.6888	12.1974	9.7232	10.1797	8.7484	5.4640	0
GSM441784	1	0	0	0	0	9.45	0	10.0277	8.9455	3.5079	10.8306	9.3498	8.5482	8.8807	5.3409	0
GSM441785	1	0	1	1	1	9.43	1	9.9687	10.5479	5.5925	12.4378	10.4737	9.5895	8.9787	5.4303	0
GSM441786	1	0	0	0	1	10.54	0	10.0179	9.3995	3.8731	12.4501	10.3849	9.4993	8.8933	5.6289	1
GSM441787	1	0	1	1	0	10.27	0	8.9609	10.7247	6.0369	11.9197	9.8214	9.4567	8.8983	5.8510	1
GSM441788	1	0	1	1	0	10.11	0	9.7060	10.8973	5.1019	12.3051	9.8177	9.4948	9.0284	5.4120	0
GSM441789	1	0	1	1	0	8.82	0	9.0860	9.6966	5.9337	10.3411	9.9041	8.0168	9.5700	5.5128	0
GSM441790	1	0	1	1	0	8.93	0	9.4728	9.7328	5.0559	12.0735	9.1456	9.6734	8.8194	5.0720	0
GSM441791	1	0	0	0	0	9.32	0	9.8693	8.6794	3.6108	11.7932	9.5238	9.4146	8.6099	5.2022	0
GSM441792	1	0	1	0	0	10.37	0	10.6375	10.5962	4.4386	9.9954	9.0125	7.4913	8.9456	4.9382	0
GSM441793	1	0	1	0	0	10.32	0	9.0360	10.7549	4.0663	12.3929	9.6281	10.1125	8.6529	5.3521	0
GSM441794	1	0	0	0	0	10.30	0	10.9180	8.9027	3.6908	12.4153	10.2283	8.9365	8.9866	5.5859	1
GSM441795	1	0	1	0	0	10.22	0	9.8025	9.9828	3.8398	12.0109	9.1430	9.2063	8.8174	5.3081	0
GSM441796	1	0	1	0	0	8.46	0	9.7775	9.9864	4.4081	12.1489	10.0452	8.8702	8.9832	5.1193	0
GSM441797	1	0	0	0	0	9.82	0	9.1060	7.9389	3.5544	12.2244	9.9599	9.3697	8.6466	5.1248	0
GSM441798	1	0	0	0	0	9.34	0	10.3376	7.9561	4.0007	12.5469	10.3269	9.3228	8.8306	5.4529	0
GSM441799	1	0	1	0	0	8.14	0	9.9939	10.1269	3.7195	11.9721	8.9429	9.4796	8.5300	5.4017	0
GSM441800	1	0	0	0	0	7.35	0	9.5899	9.0922	4.3692	13.2738	11.0821	9.6865	9.1145	5.8749	1
GSM441801	1	0	0	0	1	8.15	0	9.2677	5.7555	3.2188	12.4198	9.8930	9.6036	9.2956	5.4779	0
GSM441802	1	0	0	0	0	9.89	0	9.5234	6.3788	3.4717	12.1435	9.2104	9.3650	8.3772	5.1873	0
GSM441803	1	0	1	0	0	9.68	0	10.0715	9.5347	3.7724	12.1465	9.6126	9.1282	8.5746	5.4892	0
GSM441804	1	0	0	1	1	9.39	0	9.4203	9.1334	5.2797	12.7935	9.9519	9.6827	8.9917	5.1468	0
GSM441805	1	0	1	0	0	9.36	0	8.7095	10.4807	4.1404	12.5677	10.6886	9.0142	8.8247	6.0025	1
GSM441806	1	0	0	0	0	9.21	0	10.0195	9.3713	3.5272	10.9362	9.4785	8.8355	8.4359	5.3480	0
GSM441807	1	0	0	0	1	9.10	0	9.3090	7.2246	3.5925	10.8557	9.6436	8.9596	9.9883	5.1274	0
GSM441808	1	0	1	0	1	9.26	1	10.0528	9.6729	3.6936	12.4208	10.1194	9.1726	8.7270	5.2370	0
GSM441809	1	0	0	0	0	2.55	1	9.5240	5.1196	3.5356	11.2219	10.3241	9.3480	8.6003	5.5752	1
GSM441810	1	0	0	0	0	7.13	0	10.5374	9.2179	3.4962	12.3843	9.6649	9.3828	8.6535	5.6248	1
GSM441811	1	0	1	1	0	9.57	0	9.8426	9.6402	6.0427	11.7463	9.6965	9.3993	9.1411	5.4665	0
GSM441812	1	0	0	0	0	9.19	1	9.5019	8.6734	3.6971	10.9857	9.1748	8.6596	8.4870	5.1796	0
GSM441813	1	0	1	1	1	8.11	0	9.9820	9.5634	5.0854	10.0146	8.1797	7.6464	8.4561	4.9351	0
GSM441814	1	0	1	0	0	7.85	0	8.9036	9.9369	4.2664	10.5309	9.8548	9.3991	8.7451	4.9407	0
GSM441815	1	0	1	0	0	8.53	0	9.5342	10.1747	4.0338	13.0069	10.6682	9.4382	9.0615	5.3873	1
GSM441816	1	0	0	0	0	8.11	1	9.2821	7.1524	3.2833	12.1660	9.5751	9.1640	8.6549	5.4192	0
GSM441817	1	0	0	0	1	8.01	0	9.9737	9.4868	3.7198	12.6455	10.1500	9.0783	8.9138	5.4503	0
GSM441818	1	0	0	0	0	7.14	1	9.9186	9.3265	4.2776	13.0321	10.5362	9.3773	9.1387	5.5557	1
GSM441819	1	0	0	0	0	8.79	0	9.6039	9.4751	3.4845	11.4683	9.8279	9.7467	9.3345	5.2388	0
GSM441820	1	0	1	0	0	8.44	0	9.3914	9.9165	4.4258	12.3299	9.7199	9.6757	9.3834	5.1106	0
GSM441821	1	0	1	0	1	1.48	1	10.3061	9.7894	4.0904	12.2605	9.5142	9.7754	8.9355	5.4219	0
GSM441822	1	0	1	0	0	8.45	0	10.5050	9.8293	3.4400	12.0827	9.5539	9.9746	7.9128	5.4452	0
GSM441823	1	0	1	0	1	10.62	1	9.4786	10.1927	4.5027	12.7231	11.4462	8.5115	9.2190	5.2112	1
GSM441824	1	0	1	0	0	3.50	1	10.5617	9.8352	4.1806	13.0765	10.1630	8.6384	9.0553	5.3563	1
GSM441825	1	0	0	0	1	4.66	1	9.9962	8.5135	4.4451	12.2218	10.5734	9.0508	8.8339	5.2743	0
GSM441826	1	1	1	0	1	15.22	0	11.4754	10.7061	4.2434	12.5422	9.9596	8.6481	9.0914	4.9413	0
GSM441827	1	0	0	0	0	14.43	0	9.3333	7.5267	4.0758	11.1734	7.8045	7.7099	8.5070	4.7787	0
GSM441828	1	0	1	0	1	13.93	0	10.3375	11.6079	4.4023	12.6139	10.7123	8.3904	8.9683	5.3399	0
GSM441829	1	0	1	1	1	6.58	1	9.9924	12.8307	5.1487	12.5748	10.5518	10.3832	8.4138	5.0122	0
GSM441830	1	0	1	1	1	5.23	1	9.5480	10.1952	5.1758	13.1483	10.6144	8.1325	8.6952	5.2594	1
GSM441831	1	0	1	1		12.61	0	9.5160	10.9790	6.4770	11.8795	10.2590	8.9507	9.0764	5.3374	0
GSM441832	1	0	1	0		11.73	0	10.4307	10.0096	4.8121	12.7423	10.7083	8.4046	8.7163	4.9908	0
GSM441833	1	0	0	1	1	12.04	0	8.7125	8.3829	5.1114	12.2953	10.3334	8.6684	8.6835	4.9585	0
GSM441834	1	0	1	0	0	8.08	1	9.4832	11.6542	4.3102	12.9867	10.2691	9.8888	8.7087	5.0300	1
GSM441835	1	0	1	0	0	11.64	0	9.8925	10.0961	3.7476	12.6732	10.1272	8.8683	8.8858	4.8417	0
GSM441836	1	0	1	0	0	12.21	1	10.1524	9.9610	3.7578	11.8212	9.5006	8.8743	8.4789	5.1789	0
GSM441837	1	0	0	1	0	11.94	0	10.7448	9.2664	5.0354	12.6066	10.1210	8.2529	9.2903	5.1326	0
GSM441838	1	0	1	1	1	7.47	1	10.5744	11.3800	4.9456	12.3903	10.1178	8.7317	8.8737	4.9910	0
GSM441839	1	0	0	0	0	7.48	0	9.1863	7.1398	4.5216	12.3756	9.9382	9.7868	8.1419	4.8920	0
GSM441840	1	0	1	0	0	10.73	1	9.6652	11.2721	4.2858	12.5975	9.8300	8.9554	8.9227	5.1743	0
GSM441841	1	0	1	0	0	10.93	0	9.6674	9.6139	3.7749	12.3172	9.4672	8.8372	8.9473	4.7518	0
GSM441842	1	0	1	1	1	10.39	0	9.1270	9.6967	4.9051	13.0406	10.1478	8.7718	9.1793	4.6039	1
GSM441843	1	0	0	0	0	11.28	0	8.8862	9.4807	4.5152	11.9373	10.4153	9.4529	8.3251	4.7573	0
GSM441844	1	0	1	1	0	9.48	0	9.3304	12.0365	7.2208	12.5642	10.2394	8.9051	9.2588	4.5975	0
GSM441845	1	0	1	0	0	10.10	0	9.3364	11.9590	4.1603	13.0055	10.5482	9.2640	8.7778	4.8950	1
GSM441846	1	0	0	0	0	9.33	0	10.5471	6.4706	3.7969	12.8702	11.1865	9.2936	9.0001	4.7376	1
GSM441847	1	0	0	0	1	9.08	0	8.7741	5.8558	4.1644	13.0928	10.4245	9.3252	8.3836	4.9960	1
GSM441848	1	0	1	1	1	8.78	0	9.9414	13.0732	5.3009	12.4894	10.5784	9.0112	8.9778	4.6404	0
GSM441849	1	0	1	0	1	4.72	1	9.3148	11.6152	4.6695	13.0116	10.0750	9.4635	8.3339	5.3030	1
GSM441850	1	0	1	1	1	7.24	1	9.7452	11.6011	6.1896	12.9057	10.8648	9.1255	8.9760	5.6030	1
GSM441851	1	0	1	0	1	9.44	0	10.3809	10.5759	4.5685	12.9960	11.7966	9.4205	8.9990	5.5484	1
GSM441852	1	0	0	0	0	8.18	0	10.6579	9.4747	3.3950	12.8899	11.3986	9.6730	8.8564	5.8738	1
GSM441853	1	0	0	0	0	7.83	0	9.8530	9.1604	3.7940	12.4915	10.7757	8.6556	8.8453	5.4984	0
GSM441854	1	0	1	0	1	6.75	0	10.1718	10.7524	4.5687	12.4736	9.7196	8.7375	8.6592	5.2018	0
GSM441855	1	0	1	1	1	3.53	1	10.0690	11.7065	4.8878	12.5056	10.6380	9.7770	8.7401	5.0521	0
GSM441856	1	0	1	0		4.00	1	10.0416	11.9829	4.6398	11.8804	10.1966	8.8259	8.4115	5.0963	0
GSM441857	1	0	0	0	0	6.29	0	9.8821	9.4768	4.0275	12.6000	10.3285	9.7209	8.4146	5.0139	0
GSM441858	1	0	1	1	0	4.62	0	10.1430	12.3420	6.1979	12.7787	10.0554	9.7308	8.9749	5.0533	0
GSM441859	1	1	0	0		4.60	1	11.7643	8.6575	3.7726	12.3161	10.0121	9.4855	8.9851	5.3502	0
GSM441860	1	0	1	0	0	6.08	0	9.3220	9.6170	4.2123	12.0193	10.2385	9.6767	8.7320	4.9420	0
GSM441861	1	0	1	0	0	5.41	0	10.1094	10.4389	3.9789	12.5058	10.6000	9.3740	8.6155	4.9595	0
GSM441862	1	0	1	1	0	4.57	0	8.4878	10.3899	6.3886	12.4891	10.2154	10.1771	9.0785	5.4291	0
GSM441863	1	0	0	0	0	5.48	0	9.7579	8.8218	3.9119	12.0740	10.0446	9.5207	8.4793	5.0334	0
GSM441864	1	0	1	0	1	3.56	1	10.7499	9.8394	4.3779	12.1071	9.3851	9.5376	8.3513	5.0632	0
GSM441865	1	0	1	1	1	5.48	0	9.7922	10.7357	6.1507	12.5069	10.1056	9.4126	8.8725	4.8680	0
GSM441866	1	0	0	0	0	1.74	1	10.7277	6.4525	4.0959	12.3435	10.6304	9.8480	8.2911	4.9546	0
GSM441867	1	0	1	0	0	2.03	1	10.0372	11.5677	4.5028	12.2598	9.5252	9.4107	8.5752	4.8673	0
GSM441868	1	0	1	0	1	8.41	1	10.7093	11.0289	3.7761	13.0222	10.9049	9.0289	9.2634	5.7187	1
GSM441869	1	0	1	0	1	3.58	1	9.7313	11.0488	4.1426	12.6131	10.3981	9.0224	8.6788	5.1334	0
GSM441870	1	0	1	0	1	4.20	0	9.7139	11.1796	4.5757	12.4982	10.1106	8.7481	8.5818	4.9836	0
GSM441871	1	0	1	0	1	5.30	0	8.8508	11.7125	4.6841	12.3035	9.9011	9.0586	8.4558	4.7185	0
GSM441872	1	0	1	0	1	2.80	0	10.2554	11.6990	4.4653	12.1171	10.1910	9.0788	8.4952	5.3061	0
GSM441873	1	0	1	1	1	11.97	0	10.1765	9.8720	5.8880	11.7640	9.6368	9.1281	8.9894	5.3978	0

GSM441874		1		0	1	0	0				6.98	1	9.2338	10.5791	3.9983	12.3718	10.6256	9.4109	8.9708	5.7683	1
GSM441875		1		0	1	0	1				3.01	1	9.7633	12.6141	4.5179	12.4644	10.1157	9.7606	8.6824	5.1425	0
GSM441876		1		0	1	0	1				1.56	0	9.1981	11.3981	3.8887	11.9136	10.3836	10.7208	8.4328	5.4586	0
GSM441877		1		0	1	0	0				0.50	0	9.7427	11.7472	4.7683	12.3792	10.8740	9.6006	8.5609	5.2192	0
GSM441878		1		0	1	0	1				10.48	0	9.9489	9.6191	4.0317	11.9519	9.5530	10.2581	8.6515	5.1410	0
GSM441879		1		0	0	0	0				3.93	0	9.9841	8.5501	4.3058	12.1463	9.5442	9.2683	9.3920	5.1551	0
GSM441880		1		0	0	0	1				2.35	0	9.7399	9.0083	4.2511	11.7417	8.5842	9.1908	9.1008	5.3194	0
GSM441881		1		1	1	0	1				2.13	1	12.6914	10.6325	4.2511	12.2104	9.4482	9.5513	8.1680	4.9759	0
GSM441882		1		0	1	0	0				2.12	0	9.9700	9.7839	4.5668	11.6483	9.9828	9.7015	8.5342	5.5792	1
GSM441883		1		0	0	0	1				1.45	1	9.7647	7.8943	4.1222	11.8953	10.1549	9.5361	8.8383	5.0384	0
GSM441884		1		0	1	0					12.33	1	9.9797	10.0764	3.8083	11.2656	9.2313	9.3851	8.9923	5.1829	0
GSM441885		1		0	1	0	1				1.22	1	10.2780	10.1430	4.4576	11.2143	8.4606	10.8718	8.6534	5.3615	0
GSM441886		1		0	1	0	1				8.34	0	9.3314	10.5595	3.8162	12.0050	10.1775	9.4276	8.4333	5.1494	0
GSM441887		1		0	1	0	1				3.42	1	10.1075	11.4711	4.2820	12.1882	10.1621	8.9501	8.9722	4.8553	0
GSM441888		1		0	1	0	1				12.17	0	8.3301	11.2138	4.6592	13.0097	9.8207	9.4953	9.1414	4.7592	1
GSM441889		1		0	1	0	0				16.09	0	8.7467	12.7638	4.6395	12.0215	9.7399	9.8086	8.3027	5.0268	0
GSM441890		1		0	1	0	0				12.98	0	9.2817	11.1767	4.1397	12.5987	10.2911	9.6386	8.8503	4.8612	0
GSM441891		1		0	1	0	1				10.14	0	9.6411	11.3047	4.3373	12.4914	9.7069	8.8149	8.7122	4.7137	0
GSM441892		1		0	0	0	0				14.08	0	10.1234	7.8147	4.0814	11.8371	9.4119	6.4525	8.6990	5.0580	0
GSM441893		1		0	0	0	0				13.73	0	10.5417	8.4677	4.4789	11.3842	9.5341	8.1645	8.6996	5.0609	0
GSM441894		1		0	1	1	1				14.11	0	8.9201	10.3838	5.3895	12.0592	9.4905	9.0468	8.4136	4.8117	0
GSM441895		1		0	1	0	0				13.80	0	11.0575	10.6782	4.0207	11.6606	9.3394	9.4316	8.7744	5.1519	0
GSM441896		1		1	0	0	1				3.71	1	12.4441	9.1358	4.3858	12.5969	10.5287	10.3943	8.9266	5.4145	0
GSM441897		1		0	1	0	1				15.13	0	10.7476	11.2210	4.3883	12.3837	9.5665	9.5837	8.3609	5.0876	0
GSM441898		1		0	1	0	1				14.27	0	9.2963	10.9220	4.2370	12.9240	10.4507	10.0961	9.0468	5.3599	1
GSM441899		1		0	1	0	0				14.19	0	10.1394	10.5470	4.3835	12.4003	9.7391	9.1071	8.6621	5.1014	0
GSM441900		1		0	1	0	0				14.42	0	9.0788	9.6295	3.9931	11.3082	8.8225	9.0395	8.8020	5.3161	0
GSM441901		1		0	1	0	0				11.43	0	9.3441	11.3752	4.4269	12.6796	10.4836	9.6916	8.8930	5.4478	0
GSM441902		1		1	0	0	1				13.24	0	11.2299	8.0933	3.8948	11.8372	9.9909	7.4453	8.8591	5.4066	0
GSM441903		1		1	1	0	0				12.05	0	11.5124	10.5218	3.8573	12.3342	10.2846	9.0389	9.0402	5.2817	0
GSM441904		1		0	1	0	0				14.22	0	9.8361	9.8279	3.9786	12.4841	10.3425	8.1824	9.6081	5.2138	0
GSM441905		1		0	1	0	0				14.57	0	10.3074	9.7010	4.4636	11.9296	11.1079	9.3525	8.6848	5.7950	1
GSM441906		1		0	0	0	0				14.49	0	9.7513	8.5808	4.2420	12.3592	10.4907	8.4511	8.6942	5.4788	0
GSM441907		1		0	0	0	1				15.63	0	9.7829	9.0887	4.2274	11.8253	9.6908	8.9511	8.9980	5.4174	0
GSM441908		1		0	1	0	0				3.83	1	9.6328	11.6039	4.0275	12.5164	9.8077	9.1032	8.5214	5.0715	0
GSM441909		1		0	1	0	0				10.84	0	9.9591	10.1638	4.1441	12.0989	9.5324	9.0027	9.2188	5.0908	0
GSM441910		1		0	1	0	0				10.13	0	9.5322	10.0701	4.6187	11.6445	9.5765	9.0451	9.0275	5.1486	0
GSM441911		1		0	1	0	0				13.99	0	10.4177	10.4678	4.1972	12.0790	9.9084	8.9953	8.3190	5.0701	0
GSM441912		1		1	1	0	1				10.28	0	13.4888	9.6910	4.2679	11.9451	10.1817	9.2829	8.5971	5.1788	0
GSM441913		1		0	0	1	1				13.28	0	9.8272	9.0072	5.1582	11.6744	10.3849	8.7623	8.7583	5.5897	1
GSM441914		1		0	1	0	1				14.19	0	10.3108	10.2042	4.0244	12.8998	10.4089	9.6800	8.8605	5.0860	1
GSM441915		1		0	0	0	0				10.29	0	10.0959	8.3168	4.0657	11.8911	9.8016	9.6260	8.7999	4.8362	0
GSM441916		1		0	1	1	1				13.03	0	9.9532	11.5723	7.2375	12.4581	10.9609	9.3771	8.8391	5.2968	1
GSM441917		1		0	1	0	0				13.83	0	10.5655	10.4851	4.2210	12.4493	10.7065	9.0535	9.1403	5.4071	0
GSM441918		1		0	1	0	0				13.65	0	10.4471	10.9041	3.7466	12.1490	9.9704	9.3972	8.6279	5.1723	0
GSM441919		1		0	1	0	0				9.89	0	10.0252	11.2864	4.6522	12.1180	10.4278	8.9118	8.9097	5.0172	0
GSM441920		1		0	1	1	0				14.43	1	10.0033	11.2122	7.6617	11.6674	9.9859	9.3154	8.4981	5.3517	0
GSM441921		1		0	1	0	1				16.27	0	9.3487	11.2698	4.1660	11.8775	10.4558	9.3422	8.3414	4.9529	0
GSM447197	1	0	0	1	0	0					1.10	1	11.6336	5.7697	3.0473	12.3288	10.7316	9.1531	8.3040	5.0283	0
GSM447198	1	0	0	1	0	0					0.15	0	13.3578	5.2167	2.9869	13.0514	9.6302	8.8983	8.3614	4.4147	1
GSM447199	1	1	1	1	1	1					2.13	1	12.4875	10.0671	5.7961	12.5681	10.1992	9.7169	8.8601	5.5289	0
GSM447200	1	0	0	1	0	0	1				4.74	0	13.0648	6.2434	4.4844	12.0944	9.7207	8.8501	8.5295	5.3619	0
GSM447201	1	0	0	1	0	0	0				0.70	1	11.6992	5.5101	3.1303	12.4861	10.0276	8.8149	8.7151	4.7973	0
GSM447202	1	1	1	1	0	0	1				2.74	0	12.5241	7.6495	3.7416	12.4470	10.2837	8.7368	8.8291	5.4938	0
GSM447203	1	0	0	1	0	0	1				1.39	0	12.7355	9.3764	4.1116	11.7099	9.6924	8.0645	8.2468	5.2925	0
GSM447204	1	0	0	0	0	0	1				1.01	1	10.8615	8.3563	3.0573	11.6417	10.2335	9.3143	8.7690	5.0865	0
GSM447205	1	1	1	1	0	1	0				3.48	0	11.8653	9.0901	5.0546	12.2627	10.4967	10.2105	8.2274	4.3974	0
GSM447206	1	0	0	1	0	0	1				1.36	1	12.8440	2.0929	2.8376	12.5106	8.8796	8.6851	8.0905	5.1775	0
GSM447207	0	1	1	0	1	1	1				4.98	0	8.9355	10.2755	8.6101	12.4437	9.9189	9.0029	9.0777	5.9677	1
GSM447208	0	1	1	1	1	0	1				7.19	0	11.8867	9.9968	3.7196	13.0993	10.6394	8.8541	8.6137	6.2892	1
GSM447209	1	0	0	1	0	0	1				4.85	1	12.8061	7.1762	3.6935	11.9319	9.9127	8.7614	8.6006	4.8147	0
GSM447210	1	0	0	0	0	0	0				1.21	1	10.7201	5.4085	3.3995	12.3699	10.1153	9.1236	8.9416	5.5409	0
GSM447211	NA	0	0	1	0	0	1				9.28	0	12.3412	4.2409	2.6555	12.5939	9.4476	8.2946	8.3306	5.3198	0
GSM447212	1	1	0	1	0	0	1				4.46	0	12.1576	8.6459	4.3659	12.3945	10.5114	8.7898	9.6917	5.5944	1
GSM447213	1	0	0	1	0	0	0				7.47	0	11.7311	7.6237	4.1970	12.6047	9.8579	10.0197	9.6760	4.8981	0
GSM447214	NA	0	0	1	0	0	0				3.37	1	11.9537	4.3576	2.6927	11.7097	10.0211	9.6028	8.5197	5.5620	0
GSM447215	1	0	0	1	0	0	1				4.08	0	12.6969	7.2777	4.0618	12.5097	10.0858	8.6362	8.4508	4.7742	0
GSM447216	1	0	0	1	0	0	1				5.24	1	12.0122	8.5309	4.1545	12.3667	10.7430	9.0465	8.3810	5.6205	1
GSM447217	NA	1	1	1	1	0	0				7.95	1	12.0998	10.1213	3.4036	12.2445	9.4258	8.1925	8.3186	5.5606	0
GSM447218	1	1	1	1	0	0	1				2.72	0	11.7958	9.4206	4.6740	12.5393	10.0784	8.5314	9.1217	5.3381	0
GSM447219	NA	0	0	1	0	0	1				0.93	0	12.1100	4.1703	3.1452	11.8042	9.9157	8.8276	8.7947	5.8999	1
GSM447220	NA			1	0	0	0				2.56	0	12.7024	4.0800	2.9803	12.5005	8.8935	9.0177	8.4524	6.3037	1
GSM447221	1	0	0	1	0	0	0				3.41	0	12.6701	7.3792	3.3651	12.3966	10.0429	9.1888	8.5793	5.3734	0
GSM447222	1	0	0	1	0	0	1				1.62	0	12.6675	6.0272	2.8814	12.1995	9.7230	9.7377	8.4967	5.9617	1
GSM447223	1	1	1	0	1	1	1						9.0854	9.8603	7.2271	11.3644	9.2797	8.5590	8.8869	5.6500	1
GSM447224	1	1	1	0	1	0	1						10.8961	11.8246	4.3576	11.9619	9.4039	9.6412	8.1609	5.4177	0
GSM447225	NA	0	0	1	0	0	0						12.6108	4.7631	3.1807	12.5717	9.7649	9.1898	9.0623	6.1612	1
GSM447226	1	0	0	1	0	0	1				0.96	1	12.7912	6.6200	3.4463	12.5642	9.8503	8.9961	8.8891	5.2895	0
GSM447227	0	1	0	1	1	0	1				2.00	1	12.1877	9.8486	3.5315	12.4820	10.4946	7.9797	8.2409	4.5491	0
GSM447228	0	0	0	1	0	0	1				0.82	1	12.4508	8.9460	4.0808	11.3623	9.7536	9.0206	9.0662	5.1934	0
GSM447229	1	0	0	1	0	0	0				4.28	0	12.3875	6.2334	3.9925	12.3372	9.6032	8.7879	8.5512	5.8428	1
GSM447230	1	0	0	1	0	0	1				2.64	0	11.6557	7.6820	3.2270	12.1147	10.1922	8.9783	8.3009	5.0079	0
GSM447231	1	1	1	1	0	0	0				8.14	0	13.4824	9.3611	4.4263	12.0992	9.7374	9.1588	8.7968	6.4678	1
GSM447232	1	0	0	1	0	0	1				1.02	1	12.0553	7.7161	3.9075	12.5684	10.1567	9.6962	8.8200	4.4859	0
GSM447233	1	1	1	1	1	0	1				6.71	0	12.7124	9.6510	3.9356	12.4412	9.8063	8.7481	8.5093	4.5053	0
GSM447234	1	1	1	1	1	0	1				4.55	0	11.8568	10.5421	3.5450	12.1051	10.4267	8.5749	9.2659	5.9809	1
GSM447235	NA			1	0	0							12.1010	6.9885	3.7983	12.5061	10.5586	9.0409	9.4248	4.6706	0
GSM447236	NA	1	1	1	0	0	1						12.6826	6.7022	3.7010	12.4466	11.1564	8.8794	8.8589	4.6201	1
GSM447237	NA			1	0	0							12.7345	6.7159	3.8129	11.6565	8.7256	8.4232	8.8973	4.4214	0
GSM447238	NA	1	1	1	0	0	0						12.2543	3.5632	3.2099	12.2779	10.7124	8.9298	9.2227	5.3195	0
GSM447239	NA	1	1	1	0	0	1						11.6024	6.2124	2.7641	11.8168	9.0728	8.8890	8.4756	4.2948	0
GSM447240	NA	1	1	1	0	0	1						12.0892	4.7724	3.5527	12.1554	9.9784	8.9937	8.4520	5.7279	1
GSM447241	NA			1	0	0							11.9954	5.0664	3.1894	12.4352	10.2033	8.9860	9.4759	5.2334	0
GSM447242	NA	1	1	1	0	0	0						11.9440	5.9262	3.6891	12.3737	10.9854	8.5388	8.7544	5.0261	1
GSM447243	1	1	1	1	1	0	0				0.78	0	11.4658	11.6317	3.4054	12.5392	10.3330	9.1138	8.7201	6.6296	1
GSM447244	1	1	1	0	1	1	1						10.7207	10.8275	6.4783	11.5964	9.7060	9.2500	8.2313	5.7312	1
GSM447245	1	1	0	1	1	0	1				0.83	1	11.2855	12.3156	3.6889	12.4739	10.0748	8.9620	8.7561	5.1949	0
GSM447246	1	0	0	1	0	0	1				0.86	1	12.3530	6.0938	3.6407	12.4144	9.4915	9.4027	9.3225	4.9705	0
GSM447247	1	0	0	1	0	0	1				3.00	1	12.1851	7.6160	4.0341	12.5013	9.0176	9.0229	8.9049	5.1268	0
GSM447248	NA			0	0	0							8.4714	8.0049	4.5231	12.7519	10.9059	9.3758	9.4770	5.3440	0
GSM447249	NA			0	0	0							8.5546	8.0926	3.5216	11.6691	10.6933	7.5382	9.1522	5.3107	0
GSM447250	NA			0	0	0							8.4342	8.8565	2.9688	11.3271	10.3632	8.5746	8.8019	5.4231	0
GSM447251	NA			0	0	0							7.8881	7.7574	3.2593	12.3692	10.3285	9.1915	8.7941	4.9247	0
GSM491175	1	0	0	1	0	0	1	3	2.3	53	2.00	1	12.9583	7.6804	3.6626	12.6828	10.7158	10.4980	8.7338	6.7500	1
GSM491176	0	0	0	0	0	0	0	3	0.9	60	3.17	0	8.9400	7.3701	4.0062	12.2779	10.0203	10.1544	8.8508	4.6048	0

GSM491177	0	0	0	0	0	0	0	3	3.5	65	5.00	0	8.2968	6.8591	4.4817	12.4723	10.7415	9.5324	9.0504	6.2171	1
GSM491178	1	0	0	1	0	0	1	3	1.8	48	0.75	1	13.0763	8.2349	4.6331	12.9957	10.0315	9.2144	8.5757	4.5646	1
GSM491179	0	0	0	0	0	0	1	3	2.1	58	5.17	0	7.5098	7.0410	4.2249	12.6219	10.8444	10.0504	9.8330	5.4237	0
GSM491180	1	0	0	1	0	0	1	3	2.5	45	1.33	1	13.3736	6.7062	3.8434	12.5271	10.2947	10.2896	9.0840	4.9253	0
GSM491181	0	0	0	0	0	0	1	3	1.2	49	5.33	0	8.3540	6.7774	3.8715	12.3036	11.2956	8.9490	8.9849	5.7224	1
GSM491182	1	0	0	1	0	0	0	3	2.1	56	5.58	0	13.0149	9.0956	4.7040	12.3684	10.6593	10.3447	9.1291	4.3580	0
GSM491183	1	0	0	0	0	0	1	2	2.5	85	2.25	1	9.7353	8.5473	4.2241	12.7470	10.1994	9.8337	8.9474	4.5571	0
GSM491184	0	0	0	0	0	0	0	3	2.5	66	5.17	0	8.4632	7.1820	4.3782	12.5777	10.4145	10.4962	10.1820	5.6307	1
GSM491185	0	0	0	0	0	0	0	2	1.8	58	4.33	0	8.3391	7.3472	4.0846	12.4586	9.6479	9.8549	8.2608	5.0292	0
GSM491186	0	0	0	0	0	0	0	3	5	79	0.08	1	10.0853	5.9449	3.7440	12.9656	10.4087	9.9925	9.2639	6.2609	1
GSM491187	0	0	0	0	1	1	0	3	2.1	58	4.42	0	9.6227	12.7162	5.3331	12.3293	10.1384	9.8070	9.4581	4.4989	0
GSM491188	0	0	0	0	0	0	0	3	1	48	4.50	0	9.0318	8.2347	4.3424	12.0869	9.6063	9.2190	10.1456	5.4970	0
GSM491189	0	0	0	0	0	0	0	3	0.9	50	4.25	0	8.2894	6.9899	4.4061	12.6210	9.1802	8.5042	8.2005	5.0057	0
GSM491190	0	0	0	0	0	0	0	3	2.2	40	4.00	0	8.4626	8.0337	4.0590	12.6702	9.5114	9.4152	8.8354	4.8301	0
GSM491191	0	0	0	0	0	0	0	3	1	58	4.33	0	8.3843	6.4823	3.7384	12.0295	9.2084	9.0222	8.4962	4.4848	0
GSM491192	1	0	0	1	0	0	1	3	3.5	63	4.17	0	12.6847	8.0611	4.4660	12.4572	9.8469	8.4066	8.6665	6.2067	1
GSM491193	0	0	0	0	0	0	0	3	2.7	41	4.17	0	8.3554	7.4109	4.6016	11.9405	9.1667	9.5052	7.4667	4.5927	0
GSM491194	0	0	0	0	1	0	0	2	2.5	74	3.33	0	9.5438	13.2367	4.1329	12.2238	10.2608	9.4957	8.7300	6.4334	1
GSM491195	0	0	0	0	0	0	0	3	3	48	4.08	0	8.4517	7.2963	4.3291	12.5913	10.5220	8.9379	9.0477	5.9870	1
GSM491196	0	0	0	0	0	0	0	3	1.3	78	4.42	0	9.7455	6.5003	3.7749	12.3401	9.7292	9.5652	8.7934	4.8931	0
GSM491197	0	0	0	0	0	0	0	3	1.4	55	4.17	0	7.8160	5.4263	3.7025	12.9962	9.7007	9.1554	8.8147	5.5798	1
GSM491198	0	0	0	0	0	0	0	3	1.7	43	2.92	1	8.5727	6.8987	3.9085	11.0337	10.1166	8.1506	8.5107	4.9458	0
GSM491199	0	0	0	0	0	0	0	3	2.3	72	4.17	0	7.4263	5.7704	4.4220	12.2510	11.0685	11.1632	8.7286	4.7086	1
GSM491200	1	0	0	1	0	0	1	3	3.4	49	3.00	0	13.3884	7.3191	4.0317	12.6518	9.9191	8.9615	8.7651	6.5461	1
GSM491201	0	0	0	0	0	0	0	3	2.8	43	3.00	0	9.2919	7.4310	4.1676	12.0778	10.1151	8.2206	9.9625	5.9991	1
GSM491202	0	0	0	0	0	0	0	3	2.2	56	3.25	0	8.3245	6.9377	3.7380	12.5819	11.3258	8.5573	8.6066	5.5044	1
GSM491203	1	1	1	0	1	0	1	3	1.1	41	5.42	0	9.0630	10.6348	3.8134	12.5538	10.0129	7.8619	8.4307	4.8983	0
GSM491204	0	1	1	0	1	1	0	1	3.5	62	6.67	0	8.7978	10.7808	6.4614	12.1860	9.2471	9.5178	8.6045	4.4307	0
GSM491205	1	1	1	0	1	1	0	3	1	43	6.50	0	11.1325	10.6987	4.9026	12.5671	10.4146	10.5242	8.5636	5.0541	0
GSM491206	1	1	1	0	1	0	0	1	1.7	54	6.50	0	10.2213	11.3325	3.6853	12.8274	10.5544	9.1307	9.4506	4.4281	1
GSM491207	1	1	1	1	0	0	1	3	1.4	55	4.25	0	12.4911	9.1347	3.8077	12.5547	9.2061	9.8849	8.5124	4.8967	0
GSM491208	0	1	1	0	1	0	1	2	2.6	46	0.50	1	10.3725	10.7349	4.6933	12.3758	10.0515	9.4377	9.2381	4.6619	0
GSM491209	0	1	1	0	1	1	0	2	0.9	46	7.08	0	9.7345	9.8728	6.5276	13.0089	10.6325	10.0868	8.6811	5.6389	1
GSM491210	0	1	1	0	0	0	0	1	0.8	44	6.08	0	10.0869	9.4714	4.3342	12.4318	9.8075	9.2765	8.4272	5.2774	0
GSM491211	1	1	1	1	1	0	0	2	2.2	54	6.83	0	11.8413	11.7977	4.1046	12.4149	10.1015	8.9521	8.8211	5.1940	0
GSM491212	1	0	0	0	0	0	0	3	1.8	49	6.25	0	8.2708	6.7130	3.9671	12.2600	10.5902	8.9876	8.5955	7.0170	1
GSM491213	0	1	1	0	1	1	1	2	2.2	84	6.58	0	9.1526	11.6439	6.4461	12.5053	10.2420	9.9233	9.3375	4.7047	0
GSM491214	1	1	0	0	1	0	0	3	4.2	43	4.33	0	9.3371	12.5579	4.3634	12.7691	9.7591	8.7303	9.0241	5.3896	0
GSM491215	1	0	0	0	0	0	0	2	2.5	54	6.75	0	10.6796	6.3824	3.8857	12.5643	10.1539	9.0301	8.7326	4.7916	0
GSM491216	0	1	1	0	1	1	0	2	2.3	45	4.92	0	9.4753	11.0548	4.8708	12.2474	9.6175	9.1783	8.6764	5.4389	0
GSM491217	1	1	1	0	1	0	1	2	1.7	45	6.92	0	10.7264	11.6276	3.6909	12.2716	10.2119	9.3519	9.1122	5.0345	0
GSM491218	0	1	0	0	1	0	1	2	1.1	35	7.08	0	9.4006	11.5596	4.1484	12.2849	9.9529	10.0409	9.2271	4.6458	0
GSM491219	0	0	0	0	0	0	0	3	3	53	1.50	1	10.1432	6.3409	3.9776	12.4333	11.1134	9.2045	9.4031	7.4559	1
GSM491220	0	1	1	0	1	0	0	1	2.3	60	5.83	0	10.2218	10.0984	3.8417	12.4293	10.1419	9.4556	9.0758	5.9989	1
GSM491221	0	1	1	0	1	0	1	2	1.5	32	6.92	0	9.7697	12.3400	4.1702	11.6133	10.0900	8.5503	8.6621	4.8120	0
GSM491222	0	1	0	0	1	0	1	1	4	40	6.17	0	10.4771	11.6894	4.7552	12.5366	10.5042	9.3394	8.8932	5.1741	0
GSM491223	1	0	0	1	0	0	0	3	1.5	45	7.25	0	12.6935	6.9838	3.9738	12.5240	10.3417	8.9004	8.9283	5.7528	1
GSM491224	0	1	1	0	1	1	0	1	1.1	46	7.17	0	9.7253	12.1085	5.4655	12.8602	10.0216	9.7880	9.5130	5.7648	1
GSM491225	0	1	1	0	1	1	1	1	3	41	7.17	0	10.0702	12.7275	7.7418	12.4402	10.1118	8.1977	9.3530	4.4792	0
GSM491226	1	1	1	0	1	0	1	3	2.3	46	6.67	0	9.5217	12.4228	4.4027	12.8221	9.9885	9.0784	9.1426	5.0591	1
GSM491227	0	1	1	0	1	0	0	2	1	36	6.50	0	10.1879	11.2687	4.5340	12.0783	9.9259	8.4593	9.3061	5.3408	0
GSM491228	0	0	0	0	0	0	0	3	1.1	68	6.08	0	8.6099	6.9437	3.9915	12.2742	10.2220	8.3912	8.5007	4.9115	0
GSM491229	0	0	0	0	0	0	0	3	1.1	54	4.67	0	8.5578	5.7233	3.5399	12.4885	9.9004	9.7360	8.9936	6.1122	1
GSM491230	0	1	1	0	1	0	1	1	2.5	81	5.83	0	9.6389	11.0980	3.2135	12.4123	10.0088	8.2923	8.7019	5.6430	1
GSM491231	1	0	0	0	0	0	1	3	3	45	6.92	0	10.1029	8.4914	4.6690	12.5903	9.8724	8.0521	9.3538	4.9504	0
GSM491232	0	0	0	0	0	0	1	3	5.2	53	1.17	1	8.4120	7.4338	3.8876	12.6974	8.9896	9.5161	8.0882	4.8150	0
GSM491233	0	0	0	0	0	0	0	3	2.5	48	6.25	0	7.7114	4.6681	3.7098	12.3876	9.9943	9.0002	9.3690	5.3030	0
GSM491234	0	1	0	0	1	0	0	2	2.5	53	6.42	0	9.1885	9.9665	3.2272	11.8871	9.0113	9.5261	8.2753	4.3458	0
GSM491235	1	1	1	0	1	0	1	3	1.7	57	6.08	0	10.1211	12.4583	3.9029	12.3519	10.4039	9.5240	8.8377	5.2587	0
GSM491236	0	1	1	0	1	1	0	2	2.1	57	4.92	0	9.7203	12.8543	6.2275	12.5599	10.3833	9.0510	9.5955	5.9877	1
GSM491237	0	1	1	0	1	0	1	3	1.5	46	7.08	0	9.6631	12.6111	3.9724	11.5574	9.6788	8.0639	9.3647	5.1391	0
GSM491238	1	0	0	0	0	0	0	3	1.9	50	6.42	0	9.9599	7.1497	4.3831	12.3422	10.0445	8.7629	9.8739	6.1307	1
GSM491239	0	1	1	0	1	1	1	2	3.5	58	6.75	0	9.1440	12.6324	6.9080	12.4513	10.6079	9.7414	8.8877	4.5926	0
GSM491240	0	1	1	0	1	0	0	1	1.5	62	6.75	0	9.7789	12.0361	3.6888	12.0443	9.8897	8.7604	8.4530	5.1876	0
GSM491241	0	0	0	0	0	0	0	3	2	44	4.75	0	8.4246	7.2584	3.1126	12.1118	8.9279	9.2496	8.6358	4.8947	0
GSM491242	1	1	1	1	1	1	1	2	1.3	37	4.00	0	13.2459	10.3593	5.1531	12.2460	10.0296	8.8043	8.2381	4.8377	0
GSM491243	1	0	0	0	0	0	1	3	2.5	55	4.83	0	9.0681	5.8298	3.5714	12.4184	10.2465	8.9711	8.5766	5.0214	0
GSM491244	0	0	0	0	0	0	0	3	1.7	64	1.75	1	8.0566	5.9422	3.5691	11.9107	9.6424	9.2144	8.2316	5.1346	0
GSM491245	1	0	0	1	0	0	0	3	3	53	6.42	0	13.2961	7.1929	4.0576	12.3437	9.7523	8.9761	8.6465	5.3366	0
GSM491246	0	1	1	0	1	1	1	1	1.1	66	6.83	0	9.0464	10.9752	5.3624	12.4068	10.4921	7.3100	9.1386	4.4522	0
GSM491247	1	1	1	0	1	1	0	3	1.2	63	5.92	0	10.9008	12.7818	5.3503	12.1668	10.0668	9.1626	8.8266	5.3405	0
GSM491248	0	1	1	0	1	1	1	1	1.2	66	4.58	0	9.6672	12.0255	5.1416	12.3194	10.2608	9.1459	9.1886	5.9818	1
GSM491249	0	1	1	0	1	0	1	3	4.2	38	0.17	1	10.6925	13.2792	4.4759	11.8284	10.4519	8.4707	8.8546	6.0932	1
GSM491250	0	1	1	0	1	1	1	3	3	46	6.33	0	9.0025	13.0145	8.0903	12.2060	9.9055	9.3251	8.8821	5.3609	0
GSM491251	0	1	1	0	1	1	1	3	2.2	60	7.33	0	9.7749	12.0095	6.7971	12.4657	10.2213	9.3285	8.8085	4.9620	0
GSM491252	0	1	1	0	1	0	1	1	1.3	50	3.92	0	10.0766	11.3582	3.4299	11.8867	9.6783	8.3353	8.6338	5.0462	0
GSM491253	0	1	1	0	1	1	1	1	1.2	37	6.92	0	9.4526	11.4161	5.0436	13.1220	10.0292	8.6997	8.5740	4.8439	1
GSM491254	1	1	1	1	1	0	0	3	2	53	4.50	0	11.7098	10.2149	3.5401	12.1789	9.7971	8.8782	8.8201	4.7653	0
GSM491255	0	1	0	0	1	0	0	1	1.1	57	6.58	0	9.8962	11.7796	3.2933	12.3561	10.1910	9.2119	8.4833	4.8727	0
GSM491256	1	0	0	0	0	0	1	2	2.9	57	1.25	1	10.2261	8.2604	4.2099	12.3233	9.8226	9.2275	8.4414	5.4146	0
GSM491257	0	1	1	0	1	0	1	3	2.5	84	1.50	1	9.4549	12.3876	3.6436	13.0895	11.4298	9.5670	8.9263	5.6134	1
GSM491258	0	1	1	0	1	0	0	2	1.2	46	6.33	0	9.4256	10.1489	4.1608	12.1521	9.7379	7.5843	9.9660	4.9217	0
GSM491259	0	1	1	0	1	1	1	1	1.5	45	6.75	0	10.1054	11.4300	6.0390	12.3270	10.1743	8.8232	9.3604	5.1395	0
GSM491260	0	1	1	0	1	0	0	1	5	62	6.92	0	9.4508	10.1693	3.3580	12.2880	10.2215	7.8294	9.1762	5.0547	0
GSM491261	0	1	1	0	1	0	1	1	1.5	58	4.50	0	8.8871	11.2093	4.1234	12.1948	9.5952	8.7853	8.0367	5.1743	0
GSM491262	0	1	1	0	1	0	0	1	1.4	50	6.58	0	10.0131	10.5629	3.6634	11.9126	9.6749	8.7171	8.2918	5.5471	0
GSM491263	0	1	1	0	1	0	0	1	1.1	74	6.17	0	10.2915	11.4726	3.8794	12.0530	9.5699	8.8116	8.7516	5.0643	0
GSM491264	0	1	1	0	1	1	1	3	1.1	57	6.17	0	9.1941	11.6211	5.7277	11.7885	9.1735	8.9167	8.5715	4.7766	0
GSM491265	0	1	0	0	1	0	0	1	0.8	58	6.75	0	10.3718	11.2603	3.6971	12.1420	9.8869	8.8911	8.7746	5.3040	0
GSM491266	0	1	1	0	1	1	0	1	1	59	6.75	0	9.4186	10.4412	5.7502	12.3150	9.1606	8.4913	8.7392	5.1330	0
GSM491267	0	1	1	0	1	1	0	2	2.1	46	6.33	0	9.2057	11.3239	7.2480	12.0326	10.2476	8.6251	8.7435	5.7191	1
GSM491268	1	1	1	0	1	0	0	2	1.5	54	5.42	0	10.4791	12.2385	3.8253	12.8423	10.8209	8.9310	9.2144	5.2222	1
GSM491269	0	1	1	0	1	0	0	1	1.3	64	6.92	0	9.4154	11.2457	4.3318	12.0306	9.8785	8.7252	8.4190	5.0150	0
GSM491270	1	1	1	0	1	0	0	3	2.1	64	4.00	0	9.2782	13.2669	4.2803	12.4271	10.3925	9.5619	8.4641	4.5500	0
GSM491271	1	0	0	0	0	0	1	3	5.5	68	4.25	0	9.7818	7.1232	3.8100	12.2552	10.3997	9.0382	8.4688	5.7624	1
GSM491272	0	1	1	0	1	1	1	1	2	49	4.67	0	9.4722	11.6633	7.4180	12.2982	9.9997	9.3073	8.9445	4.8698	0
GSM491273	0	1	1	0	1	0	1	2	6.5	37	4.92	0	9.9255	11.3742	3.7572	12.3775	11.6063	9.3161	8.5373	6.2065	1
GSM491274	0	1	1	0	1	1	1	3	4.5	63	5.75	0	8.1636	11.0858	5.9824	12.2827	10.5973	9.7628	8.0266	6.4593	1
GSM491275	1	1	1	1	1	1	1	3	2.3	71	5.92	0	11.8145	11.4783	6.5225	12.2630	9.8621	8.8165	8.1324	4.8572	0
GSM491276	0	1	1	0	1	1	1	2	4	45	4.42	0	9.2366	12.0096	5.3898	12.5489	10.3993	8.4464	9.1090	5.2237	0
GSM491277	0	1	1	0	1	0	1	3	2.3	78	4.42	0	10.2031	14.2877	4.6807	13.1683	10.8769	8.3740	8.8365	5.9265	1
GSM491278	0	1	1	0	1	1	1	3	5.6	49	4.42	0	7.7667	10.9805	5.3869	12.7297	10.3164	8.6616	8.6636	5.4964	0
GSM491279	1	1	0	1	0	0	1	3	4	42	4.50	0	13.2001	8.5409	3.6868	12.8526	10.4700	9.5191	8.7971	5.0431	1
GSM491280	0	1	1	0	1	1	0	2	2.5	54	5.92	0	9.7755	14.0762	8.4027	12.6767	10.5635	8.1985	9.2845	6.0150	1
GSM491281	0	1	1	0	1	1	1	2	2.5	82	4.00	0	9.6434	12.5327	5.3693	12.5199	9.7764	9.4118	8.5795	5.2332	0

GSM491282	0	1	1	0	1	1	1	3	5	48			5.92	0	9.7404	12.9326	6.7474	12.9001	9.7393	9.5520	8.9947	5.2422	1
GSM491283	1	1	1	1	1	1	0	3	3	49			5.92	0	12.8585	11.9598	5.1214	12.3735	9.7784	9.5056	9.0675	5.4044	0
GSM491284	0	0	1	0	0	0	1	2	1.2	45			6.17	0	7.6256	7.8252	4.1337	12.3178	9.3907	9.9064	8.3519	5.0842	0
GSM491285	1	1	1	1	1	0	1	3	4	47			3.00	0	12.3654	9.5429	4.1097	12.5592	9.5410	9.6803	8.7023	5.4057	0
GSM491286	0	1	1	0	1	0	0	2	1.6	56			1.50	1	10.2674	12.7813	4.0334	12.5424	10.9620	8.0983	9.1850	4.7380	1
GSM491287	1	1	1	1	1	0	0	3	1.9	45			6.08	0	12.5087	10.3704	4.2058	12.5174	10.6172	9.2829	9.7373	5.2189	0
GSM491288	0	1	1	0	1	0	1	3	4	41			5.58	0	9.3123	11.6084	3.9622	12.2205	10.7451	9.0035	8.7367	5.7373	1
GSM491289	1	1	1	1	1	1	1	2	4.5	32			4.58	0	12.5144	12.8439	6.0914	12.5158	10.1483	8.5476	9.1631	5.6852	1
GSM519117				1	0	0				33	7.00	0	7	0	12.4172	8.3520	3.6511	12.6030	10.4785	8.7903	8.7732	5.2696	0
GSM519118				0	0	0				40	7.40	0	7.4	0	9.5894	8.1113	3.8783	12.6196	10.8168	9.1237	9.1575	5.3428	0
GSM519119				0	1	1				35	7.20	0	7.2	0	8.8383	10.3897	7.8165	12.4459	10.2858	8.4771	8.6726	4.2734	0
GSM519120				0	1	1				47	6.30	0	6.3	0	10.2459	11.6117	6.0512	12.3909	9.2412	8.7535	8.3652	4.6063	0
GSM519121				0	0	0				41	7.00	0	7	0	9.0991	7.8004	4.7796	12.8074	11.1537	9.0892	9.3130	4.3255	1
GSM519122				1	0	0				44	7.30	0	7.3	0	12.6336	4.9079	3.4790	12.2547	10.7739	9.0760	8.9073	5.6311	1
GSM519123				0	1	0				62	6.80	0	6.8	0	9.0356	10.7764	4.1585	12.5414	10.5118	8.6135	9.6649	4.6698	0
GSM519124				0	0	0				43	7.10	0	7.1	0	8.9043	8.8323	4.0585	12.4974	9.8810	9.0739	8.7122	5.6506	1
GSM519125				0	0	0				40	6.50	0	6.5	0	8.3917	7.7783	3.7817	11.9585	9.8084	8.8969	8.3070	4.7330	0
GSM519126				0	0	0				48	5.40	1	5.4	0	8.5785	6.7265	3.5750	12.3937	10.5170	9.7063	9.7812	5.0857	0
GSM519127				1	0	0				33	7.10	0	7.1	0	12.6914	8.1257	3.7208	12.7192	9.9937	8.7331	8.6003	4.2536	0
GSM519128				0	1	1				51	6.90	0	6.9	0	9.6045	11.3929	6.3623	12.2931	9.8635	8.9710	9.3833	4.9407	0
GSM519129				0	0	0				57	7.00	0	7	0	8.9341	6.5804	3.2717	11.9185	9.9171	9.1246	8.7225	5.9304	1
GSM519130				0	0	0				43	6.40	0	6.4	0	8.0979	7.3799	4.3171	12.1772	9.7350	9.4691	9.0877	4.9722	0
GSM519131				0	1	1				38	7.00	0	7	0	8.9372	10.3234	5.5241	12.4563	9.9324	9.5288	9.0675	5.2790	0
GSM519132				0	1	1				48	8.00	0	8	0	9.7976	10.1924	5.9451	12.1880	10.0645	9.4289	8.6990	5.2784	0
GSM519133				1	0	0				41	1.80	1	1.6	1	13.3536	8.3554	3.6531	12.7758	10.5589	9.6923	8.5445	5.1621	0
GSM519134				0	1	0				39	6.10	0	6.1	0	9.2781	12.0363	3.5219	12.5769	9.9723	8.9966	8.7722	4.9258	0
GSM519135				1	0	0				63	3.50	1	1.7	1	12.7389	4.6537	3.2474	12.2891	9.9579	9.3955	8.3990	5.3478	0
GSM519136				0	1	0				43	7.00	0	5.1	1	9.2884	11.2568	4.6271	12.3555	10.5181	8.7846	9.4969	5.1440	0
GSM519137				1	0	0				55	7.20	0	7.2	0	12.9044	5.1648	3.4827	12.4480	10.3698	9.5038	8.2914	5.0260	0
GSM519138				0	1	1				25	5.20	1	0	1	9.8663	11.0102	5.1425	12.8984	11.3469	9.5282	8.8767	5.8634	1
GSM519139				0	1	1				45	7.10	0	7.1	0	9.4120	11.9738	7.2077	12.7654	10.4516	8.3381	8.7872	4.8632	0
GSM519140				1	1	0				45	6.80	0	6.8	0	12.5618	10.8349	3.8819	12.4763	10.5980	8.8880	8.6209	4.8481	0
GSM519141				0	1	1				62	1.90	1	1.4	1	10.0523	11.4399	6.4024	12.4002	10.2551	9.2413	9.3413	5.0251	0
GSM519142				0	0	0				43	6.90	0	6.9	0	9.0740	6.9829	3.6583	12.6373	10.7960	9.7964	8.1572	4.7216	0
GSM519143				0	1	1				66	5.40	0	5.4	0	10.4757	11.8889	7.5827	12.7856	11.3768	9.2796	9.6417	5.5592	1
GSM519144				0	0	0				42	1.20	1	0.8	1	8.1964	5.6238	3.2910	12.8582	10.6407	10.2962	8.5150	5.6016	1
GSM519145				0	1	1				44	5.80	1	5.7	1	10.9258	11.8770	6.5432	11.9161	10.2874	8.8046	8.9289	5.6702	1
GSM519146				0	1	0				39	6.90	0	4.1	1	10.7780	11.2718	4.0011	12.1132	11.0939	9.4317	8.9743	5.2226	1
GSM519147				0	1	1				52	6.50	0	6.5	0	10.8181	11.2821	5.6354	12.5456	10.7602	9.2459	9.4497	5.9576	1
GSM519148				0	1	1				63	6.90	0	6.9	0	9.5595	10.6236	5.5651	12.4412	10.4703	9.7826	9.1247	4.9483	0
GSM519149				0	0	0				35	4.00	1	2.6	1	8.6797	8.9389	4.3239	12.7535	10.7212	9.7814	9.0526	6.7776	1
GSM519150				0	1	1				43	6.70	0	6.7	0	9.3583	9.9915	5.2675	12.3006	10.7883	9.7699	8.7887	4.5940	0
GSM519151				0	0	0				47	6.00	0	6	0	8.9657	5.7255	3.5378	12.3224	10.1772	9.3285	8.3698	4.3136	0
GSM519152				0	0	0				31	6.00	0	6	0	8.0927	9.4150	3.5092	12.4857	10.5598	9.6640	8.2579	5.1130	0
GSM519153				0	1	0				29	3.70	1	3	1	9.3412	11.4674	3.8728	12.4436	11.1248	8.6335	9.6153	5.1506	1
GSM519154				1	0	0				58	0.80	1	0.7	1	12.1695	6.6910	3.8301	12.2784	10.5169	8.8061	8.7248	5.3487	0
GSM519155				0	0	1				46	6.70	0	6.7	0	9.5501	9.2286	5.1951	12.0446	10.1258	8.8252	8.7961	3.9392	0
GSM519156				0	1	1				67	5.80	0	5.8	0	9.8722	12.2817	9.5434	12.2636	10.3126	9.0546	8.7877	5.1244	0
GSM519157				0	1	1				43	6.00	0	6	0	10.5844	10.7028	6.6527	12.5461	10.4066	8.7516	9.2133	4.7399	0
GSM519158				1	1	1				46	6.30	0	6.3	0	12.9031	11.3436	5.6894	12.1306	10.2001	9.2966	8.6470	5.0298	0
GSM519159				0	1	1				33	6.40	0	6.4	0	10.6332	11.6660	5.9148	11.9305	10.3352	8.8507	9.4597	6.6761	1
GSM519160				0	1	1				38	5.10	0	5.1	0	9.0267	10.3538	6.4582	12.1793	9.7198	9.6678	8.8273	4.3981	0
GSM519161				0	0	0				48	6.40	0	6.4	0	8.1416	7.5617	3.7721	12.3886	10.2202	9.3047	8.6725	5.5468	0
GSM519162				0	1	1				49	5.90	0	5.9	0	7.9690	13.0348	5.9097	12.8075	10.5634	9.2107	9.4821	6.6436	1
GSM519163				0	1	0				52	5.40	0	5.4	0	9.9372	11.0356	3.7703	12.6666	10.6794	8.7838	8.9630	6.3733	1
GSM519164				0	0	0				66	5.90	0	5.9	0	8.3034	9.3255	3.6018	12.1901	10.1982	9.0507	8.5606	4.8460	0
GSM519165				0	0	0				49	6.40	0	6.4	0	9.7007	6.2092	3.9016	12.5806	10.2018	8.8504	8.9709	4.9314	0
GSM519166				1	1	0				53	2.30	1	1.2	1	12.4550	11.0658	3.9250	12.4132	10.2135	8.9421	9.2895	4.5656	0
GSM519167				1	0	0				41	7.00	0	7	0	13.5914	7.2546	3.7263	12.5625	10.5289	8.6531	8.7675	4.7485	0
GSM519168				0	1	1				40	6.50	0	6.5	0	9.6056	10.8822	5.4995	12.4959	11.3917	9.0117	9.0657	3.7792	1
GSM519169				0	1	1				67	6.00	0	6	0	9.8175	11.5465	5.8164	12.4095	10.3661	8.6579	9.8664	4.9982	0
GSM519170				1	1	1				60	6.50	0	6.5	0	12.9717	12.7882	5.3583	12.5296	10.6498	9.0091	9.4526	4.4272	0
GSM519171				0	1	0				48	6.90	0	6.9	0	11.1141	10.4892	3.7529	12.6552	10.7096	9.5737	9.0962	4.3075	0
GSM519172				1	0	0				55	1.80	1	1.6	1	13.0696	9.2840	3.9501	12.4341	10.3017	8.6983	8.6060	4.3736	0
GSM519173				0	1	1				68	6.80	0	6.8	0	9.5229	12.1884	5.2607	12.6772	10.6981	8.5335	9.2107	3.6936	0
GSM519174				0	1	1				68	6.80	0	6.8	0	9.3453	12.7249	5.5223	12.0105	10.6200	8.1613	9.1753	3.7962	0
GSM519175				0	0	0				61	5.30	0	5.3	0	9.6751	9.3074	3.7869	12.8748	11.3917	9.3442	9.5418	4.1948	1
GSM519176				0	1	0				61	5.70	0	5.7	0	9.2995	10.6143	4.7379	12.6841	11.8520	9.2470	9.2108	3.7849	1
GSM519177				1	0	0				24	6.60	0	6.6	0	12.4868	6.6766	4.4137	12.0841	10.9364	8.2346	9.1750	3.6536	0
GSM519178				0	1	1				45	5.20	0	5.2	0	9.5128	11.3305	7.0708	12.6178	11.4465	9.1866	9.1021	4.2558	1
GSM519179				0	1	1				49	4.90	0	4.9	0	10.0292	13.4037	6.0988	12.2262	10.9462	8.5712	9.6211	3.8557	1
GSM519180				1	0	1				34	5.10	0	5.1	0	12.1698	8.0311	4.9893	12.4649	11.1197	9.3114	8.9751	4.1827	1
GSM519181				0	0	0				61	6.10	0	6.1	0	9.3277	7.7307	3.7118	12.7389	11.0212	8.5804	9.5002	3.5111	1
GSM519182				0	1	1				45	9.20	0	9.2	0	9.1047	12.6597	8.4941	12.6394	11.0075	8.9828	9.7843	3.7407	1
GSM519183				0	1	0				54	6.50	0	6.5	0	9.8543	10.6274	3.2377	13.0014	11.1745	10.0653	8.9988	6.0873	1
GSM519184				0	0	0				62	5.00	0	5	0	8.8993	5.5528	4.0212	12.7611	10.6070	9.3465	10.0616	6.9605	1
GSM519185				1	1	1				47	6.10	0	6.1	0	11.1634	11.7300	6.4119	12.4728	10.9265	9.7283	9.0936	6.2076	1
GSM519186				1	0	0				43	6.60	0	6.6	0	13.0079	8.3152	3.6068	12.4083	10.6066	9.1747	8.9645	5.2349	0
GSM519187				0	1	1				70	6.50	0	6.5	0	10.7796	13.7513	5.3752	12.8747	11.4385	8.8733	9.4079	6.8774	1
GSM519188				0	0	0				42	6.80	0	6.8	0	6.2927	7.2576	3.1037	12.2600	9.6799	9.5976	8.5008	5.0757	0
GSM519189				0	1	1				48	6.40	0	6.4	0	9.1205	11.8276	7.4864	12.4722	10.1784	8.7019	9.7349	5.2531	0
GSM519190				0	0	0				60	0.90	1	0.9	0	10.4722	4.7521	3.1922	12.9741	10.6527	9.6849	10.5269	4.9261	1
GSM519191				0	0	0				59	6.40	0	6.4	0	8.6387	6.9895	4.7790	12.4175	10.2153	9.3338	8.5966	4.7779	0
GSM519192				0	1	1				50	6.50	0	6.5	0	9.7190	11.0020	6.2378	12.3411	10.0081	8.8127	9.1184	5.3660	0
GSM519193				1	0	0				46	4.00	1	2.1	1	13.4408	5.9579	3.6809	12.4849	9.9322	9.3838	8.4205	4.8307	0
GSM519194				0	0	0				38	10.60	0	10.6	0	9.2436	7.6003	4.0337	12.1914	9.6461	8.6840	9.1071	4.4275	0
GSM519195				0	0	0				58	14.10	1	14.1	0	8.0536	8.5245	3.4090	12.5028	10.2490	9.1208	8.7352	5.0390	0
GSM519196				1	0	0				42	2.30	1	2.3	0	12.4416	5.6092	3.4870	12.1918	9.7061	8.2133	8.5542	4.5445	0
GSM519197				1	0	0				51	13.80	0	8.2	1	12.6901	6.1201	3.4925	12.6496	10.0113	8.5683	8.6155	4.9641	0
GSM519198				0	1	1				53	13.80	0	13.8	0	8.0384	11.0095	5.1119	12.3731	9.8996	8.4176	8.7620	4.5096	0
GSM519199				0	1	1				40	14.10	0	14.1	0	9.9002	10.7740	5.3795	12.6139	10.3720	8.8188	9.2209	5.7507	1
GSM519200				1	1	1				52	4.30	1	2.9	1	13.0788	9.9166	5.5837	12.5568	9.8324	7.8786	8.8696	4.8041	0
GSM519201				1	0	0				57	13.20	0	13.2	0	12.4476	6.5741	3.8566	12.4217	9.4175	8.6892	8.2563	4.9546	0
GSM519202				0	1	1				40	4.70	1	2.4	1	10.0343	11.1798	5.3159	12.5816	10.5076	8.6443	9.0649	4.8328	0
GSM519203				0	1	0				39	9.00	1	3.5	1	10.0633	9.6576	3.3237	13.0719	11.1941	9.0717	9.4448	5.2096	1
GSM519204				1	0	0				44	2.90	1	1.6	1	12.6913	5.3702	3.4292	12.4281	9.3638	8.2128	8.5496	4.0748	0
GSM519205				0	0	1				39	11.60	0	11.6	0	9.2243	9.3087	6.9220	12.5137	10.4713	8.4349	9.2454	4.8456	0
GSM519206				0	0	1				27	11.50	0	11.5	0	9.3191	9.3813	5.3126	12.4199	10.6139	9.0491	8.7167	4.4950	0
GSM519207				1	1	0				54	13.00	0	13	0	11.3177	10.7220	3.5918	12.1616	9.8272	9.5614	8.3018	4.5849	0
GSM519208				0	1	1				34	13.10	0	13.1	0	9.8685	11.6111	8.7825	12.3674	9.4200	8.7194	9.0705	4.8609	0
GSM519209				1	1	0				48	12.40	0	12.4	0	11.8669	10.5360	4.0611	12.5000	10.4972	8.3797	8.7030	4.6782	0
GSM519210				0	0	0				53	0.80	1	0.8	0	11.1053	7.8844	3.3597	12.8349	11.0798	9.7331	8.9626	5.2933	1
GSM519211				0	1	1				41	11.60	0	11.6	0	10.5364	10.6362	5.1372	12.9845	11.0795	9.2459	9.6193	6.4610	1
GSM519212				0	1	1				40	6.30	1	4.8	1	9.2833	11.2178	5.9319	12.3436	10.6464	8.8530	9.1867	5.3374	0
GSM519213				0	1	1				38	4.90	1	3.8	1	9.5146	10.5622	5.2595	12.5127	9.9600	8.6082	8.7249	5.0525	0

GSM519214	0	1	1	71	8.30	1	8.3	0	9.5612	11.7716	7.2385	13.0513	10.9296	8.3167	9.4962	5.0683	1
GSM519215	1	0	0	37	13.30	0	13.3	0	13.1322	8.3889	3.9676	12.3194	9.4238	9.1044	8.4683	4.8286	0
GSM519216	0	1	0	43	12.20	0	12.2	0	8.1464	9.9410	2.9091	12.5263	10.4619	8.6960	9.0057	4.5629	0
GSM519217	1	0	0	51	1.30	1	0.9	1	13.0094	5.7555	3.6576	12.5204	10.0734	8.0973	9.0737	5.1160	0
GSM519218	0	0	0	39	12.20	0	12.2	0	8.0392	7.0161	4.0352	12.7784	10.0534	8.6465	8.4437	5.4716	0
GSM519219	0	1	0	77	8.10	0	8.1	0	9.4359	10.9615	3.2341	12.4916	10.2624	9.2674	9.0912	4.9403	0
GSM519220	0	0	0	43	11.00	0	11	0	9.4162	8.5995	4.1029	12.4603	10.4819	9.1900	8.8325	5.4242	0
GSM519221	0	1	1	38	12.20	0	12.2	0	9.6200	10.5496	6.6664	12.2497	10.0971	9.1652	9.7040	4.8305	0
GSM519222	1	1	0	36	5.00	1	3.6	1	12.7779	10.5341	3.3422	13.3206	11.0186	8.8615	9.4062	5.6463	1
GSM519223	0	1	1	62	1.40	1	0.5	1	9.6504	11.3559	6.8907	11.8153	10.4630	8.8327	9.1386	4.5208	0
GSM519224	0	0	1	43	6.50	1	5.9	1	8.4769	8.2098	5.5060	12.3035	10.5079	9.5113	8.7483	4.3497	0
GSM519225	1	1	0	61	5.70	1	4	1	11.8893	12.2346	4.0154	12.3546	9.1417	8.2387	8.6849	4.9037	0
GSM519226	0	1	1	63	9.80	0	9.8	0	9.0025	10.4781	4.8457	13.0380	10.2449	8.8383	9.1343	4.2949	1
GSM519227	0	1	1	52	11.60	0	11.6	0	8.8497	10.7341	6.1074	12.5392	10.4031	8.5890	9.5869	5.2555	0
GSM519228	0	1	1	47	12.10	0	12.1	0	9.3167	11.0045	7.2108	12.6616	10.2922	9.1704	9.0078	5.2555	0
GSM519229	0	1	1	50	9.20	1	2.7	1	10.6476	11.1694	6.7404	12.2477	10.0180	9.2246	9.1076	4.8972	0
GSM519230	0	1	0	48	12.90	0	12.9	0	11.0584	10.4191	4.1768	12.4685	9.7216	8.2210	9.3396	4.4302	0
GSM519231	1	0	0	32	12.70	0	12.7	0	12.9522	7.7002	3.3758	12.6371	10.5272	8.5699	8.7580	5.2749	0
GSM519232	1	1	1	43	12.10	0	12.1	0	13.1435	11.9214	8.4422	11.7591	10.2852	8.9743	9.3637	4.6598	0
GSM519233	0	0	0	51	12.80	0	12.8	0	9.7008	9.4388	4.8034	12.5639	10.0035	8.8620	8.6201	4.8076	0
GSM519234	0	1	1	59	10.90	0	10.9	0	10.3316	12.9328	9.1705	12.9904	11.1109	8.9189	10.1413	5.2901	1
GSM519235	1	0	0	40	2.50	1	2.1	1	12.7020	5.3304	3.4574	12.2194	9.3978	9.0815	8.8491	5.1447	0
GSM519236	0	0	0	43	2.00	1	1.7	1	8.4819	8.9049	3.9811	12.1616	9.7824	8.6049	8.5535	5.3008	0
GSM519237	0	0	0	39	1.20	1	0	1	7.9097	7.0266	3.1226	12.4855	10.6620	8.9185	9.7270	5.1040	0
GSM519238	0	1	0	43	11.40	1	0	1	9.6581	10.5366	3.5433	12.6647	10.5651	9.5067	8.2508	4.1300	0
GSM519239	1	1	0	38	4.60	1	3.2	1	12.5041	11.7830	4.6795	12.7346	10.2642	8.9500	8.8124	5.5562	0
GSM519240	0	1	1	54	12.30	0	12.3	0	8.6543	10.4291	6.4418	12.5340	10.0829	8.9664	8.8240	5.2294	0
GSM519241	1	1	1	42	11.00	0	11	0	11.3572	11.4292	7.1906	12.7013	10.4472	8.8698	8.7974	5.0943	0
GSM519242	0	1	1	45	11.10	0	11.1	0	8.1347	11.0605	5.3691	12.4578	10.5277	9.1476	8.5603	6.5392	1
GSM519243	1	0	0	38	10.80	0	10.8	0	13.4964	7.9584	4.0624	12.4701	10.2101	8.6039	8.8255	5.5613	0
GSM519244	1	1	0	53	11.00	0	11	0	12.8121	9.7801	3.4843	12.6378	10.4366	9.3647	9.2408	4.9787	0
GSM519245	1	1	0	46	7.00	1	3.9	1	13.2866	9.9335	3.7422	12.2182	9.9996	8.9342	8.9708	5.7419	1
GSM519246	0	1	0	50	8.90	1	6.8	1	8.9147	10.2800	4.3860	12.4544	10.3246	8.7831	9.0869	5.2843	0
GSM519247	0	1	0	37	12.60	0	12.6	0	9.1174	11.7350	3.9075	12.2375	9.9035	8.1767	9.3745	4.6046	0
GSM519248	0	1	1	39	5.90	1	5.3	1	8.9283	10.1050	5.5024	12.7891	10.6076	9.0985	9.4793	5.3765	0
GSM519249	0	0	0	58	11.20	0	11.2	0	11.0511	7.8061	3.5282	12.1178	10.2791	8.9390	8.9320	6.4751	1
GSM519250	0	1	0	61	11.00	0	11	0	9.4922	10.4544	3.5137	12.2053	10.1194	8.0593	8.7624	4.7816	0
GSM519251	0	0	0	30	11.30	0	11.3	0	9.0239	5.7219	3.8137	12.6546	10.9727	9.4377	8.7923	6.4977	1
GSM519252	0	1	1	62	11.60	0	11.6	0	9.7615	10.6093	6.4971	12.4300	10.4719	9.0509	9.0735	4.4609	0
GSM519253	0	1	0	62	2.40	1	0	1	9.0235	10.4904	3.9648	12.1710	10.5094	8.2714	8.7165	5.3284	0
GSM519254	0	0	1	43	11.90	0	11.9	0	9.3265	9.1978	5.3512	12.5601	10.6394	9.0541	9.1336	3.8590	0
GSM519255	0	1	1	41	12.70	0	12.7	0	8.8700	11.2573	7.1535	12.6194	10.1569	8.7198	9.4690	4.8371	0
GSM519256	0	0	1	43	12.80	0	10.6	1	9.0770	9.1197	6.2702	12.4710	10.8713	9.3699	9.2986	4.9300	0
GSM519257	0	1	0	27	4.60	1	1.7	1	9.3809	10.7725	4.2314	12.0276	9.8669	9.2189	8.6774	5.0482	0
GSM519258	1	1	0	58	10.90	0	10.9	0	11.2940	10.3590	2.9895	12.7918	9.8014	10.0375	9.0371	4.7276	0
GSM519259	0	1	0	43	11.40	1	0	1	9.2545	10.0403	3.5854	12.2270	9.8659	9.5684	8.2184	4.8320	0
GSM519260	0	1	1	42	12.50	0	12.5	0	9.0951	9.8082	6.0410	12.3815	10.1051	9.2154	9.1870	5.0397	0
GSM519261	0	1	0	41	12.50	0	12.5	0	9.9662	11.5029	4.4287	12.3475	10.8029	9.0653	9.3816	5.6586	1
GSM519262	0	0	0	60	9.90	0	9.9	0	9.8286	5.5232	3.1588	12.4553	10.5898	9.0439	9.0263	4.3558	0
GSM519263	1	0	0	46	2.90	1	2	1	11.4850	7.0024	3.5146	11.7288	9.5937	8.9506	8.4144	4.6206	0
GSM519264	1	1	1	57	11.10	0	11.1	0	11.6213	10.7956	5.4044	12.3886	10.5504	9.1872	8.9720	5.2800	0
GSM519265	1	0	0	51	5.70	1	5.1	1	12.7595	6.0020	3.3626	12.8482	10.9458	9.1663	7.9538	4.7843	1
GSM519266	1	0	0	58	1.00	1	0.9	1	13.1106	6.4195	3.7557	12.2495	10.0127	9.2089	8.7801	5.8201	1
GSM519267	1	0	0	48	13.10	0	8.8	1	13.0142	8.6598	3.4777	12.5351	10.2027	9.0006	8.9802	5.0987	0
GSM519268	0	0	0	36	11.10	0	11.1	0	8.1517	7.8785	3.2984	12.2753	10.3268	8.8367	9.0063	4.2870	0
GSM519269	0	0	0	42	5.50	1	2.9	1	8.9077	6.6067	2.9165	12.3278	10.3508	9.2111	8.6404	4.1768	0
GSM519270	0	1	0	46	12.10	0	12.1	0	9.5500	9.6362	3.2136	12.8613	10.7692	9.1531	10.0146	5.2873	1
GSM519271	0	1	0	51	9.40	0	9.4	0	9.5363	11.3293	4.2264	12.1217	11.0818	9.2581	8.7217	4.6464	1
GSM519272	1	0	0	71	11.10	0	11.1	0	11.2318	7.2655	3.8317	12.4294	10.8092	9.4870	8.7385	6.3715	1
GSM519273	0	1	1	51	12.10	0	12.1	0	8.7904	11.7156	7.8857	12.4705	10.2937	9.0788	10.1336	4.4624	0
GSM519274	0	0	0	31	10.80	0	10.8	0	7.5273	6.7147	3.5541	12.5241	10.1686	9.8814	9.7878	4.7934	0
GSM519275	0	1	1	34	10.20	0	10.2	0	9.8201	10.8789	6.7652	12.5307	11.0897	8.9011	9.0689	5.1876	1
GSM519276	0	0	1	51	12.40	0	12.4	0	9.7796	9.4063	4.9566	12.3501	10.1881	9.0510	9.4680	4.7519	0
GSM519277	0	0	0	33	7.40	0	7.4	0	8.4026	7.3626	3.5471	12.4400	10.8136	8.7844	9.2926	6.5233	1
GSM519278	0	1	1	43	11.60	0	11.6	0	8.5985	11.2744	8.6264	12.5760	10.3731	9.6836	8.7965	4.5122	0
GSM519279	0	0	0	48	7.50	1	4.8	1	9.2720	8.1683	3.5842	12.0369	9.9984	7.9543	9.2140	4.7716	0
GSM519280	0	1	1	56	2.20	1	1.3	1	9.3447	10.6480	5.6421	12.9356	11.6210	9.5351	9.3916	3.8906	1
GSM519281	0	0	0	41	7.10	0	7.1	0	7.9145	6.2850	3.7985	12.4511	10.1181	9.1671	9.5110	6.9250	1
GSM519282	1	0	0	58	10.20	0	10.2	0	11.6557	6.4848	3.6644	12.6674	9.6703	9.4464	8.6365	4.7402	0
GSM519283	0	1	1	53	10.30	0	10.3	0	8.9890	11.9309	7.6080	12.0324	9.8193	8.8097	8.7969	4.5444	0
GSM519284	0	0	0	44	11.80	0	11.8	0	8.9701	8.8994	3.5094	12.5955	10.6023	9.0165	8.7624	5.2078	0
GSM519285	1	1	1	57	11.50	1	11.5	0	13.0412	12.6029	5.4441	12.4363	10.3182	8.7245	8.8188	5.4551	0
GSM519286	0	0	1	45	10.50	0	10.5	0	9.2807	9.4324	5.1521	12.2992	10.0444	8.7429	8.9088	4.5972	0
GSM519287	0	1	1	41	11.70	0	11.7	0	9.5481	10.9807	5.9184	11.8678	10.5797	8.9005	9.3052	4.3042	0
GSM519288	0	1	1	54	11.70	0	11.7	0	8.1434	10.7508	5.4154	12.5625	10.3258	9.3711	9.0512	4.0696	0
GSM519289	1	0	0	64	1.60	1	1.6	0	12.3757	8.1058	3.7176	12.3819	9.9951	7.6598	8.2253	4.6407	0
GSM519290	0	1	1	56	10.00	0	10	0	9.5988	11.9511	5.7649	12.4423	10.4157	8.5037	9.4760	5.0886	0
GSM519291	0	1	0	58	11.70	0	7.3	1	9.1296	12.3575	3.9246	12.1253	9.9030	8.7551	8.8926	5.2976	0
GSM519292	0	0	0	38	12.50	0	2.4	1	10.3954	9.2582	4.7301	12.3765	10.2056	9.1649	9.6934	5.9367	1
GSM519293	1	0	0	42	1.30	1	0	1	13.2286	7.6316	3.9048	12.5323	10.3590	8.5512	9.2420	4.9759	0
GSM519294	0	0	0	69	10.20	0	10.2	0	9.3756	6.9604	3.6413	12.2641	10.1908	8.8758	8.5525	4.7929	0
GSM519295	0	1	1	79	10.40	0	10.4	0	9.9760	10.1743	6.2123	12.6695	10.6666	9.0599	8.7353	5.7978	1
GSM519296	1	1	0	37	11.20	0	11.2	0	11.7271	10.4491	4.3691	12.5649	10.4585	9.4480	8.8896	6.2007	1
GSM519297	0	1	0	44	9.80	0	9.8	0	8.3076	10.7802	4.5318	12.4912	10.0549	8.4000	9.3912	4.6779	0
GSM519298	0	0	0	48	1.20	1	1.2	1	9.7434	9.2509	4.8125	12.5106	9.9207	8.5861	9.3509	5.4660	0
GSM519299	0	1	0	43	9.40	0	9.4	0	8.5071	10.1127	3.9975	11.7771	9.8927	9.0201	9.3804	6.0344	1
GSM519300	1	0	0	50	11.30	0	11.3	0	12.7115	7.8852	3.5006	12.6409	10.7742	8.9573	8.3751	5.3720	0
GSM519301	0	0	0	30	10.00	0	10	0	8.3943	8.1729	4.0050	12.2034	10.3776	9.1154	9.5617	7.0251	1
GSM519302	0	0	0	36	11.50	0	11.5	0	9.2653	6.1241	3.6136	12.4595	10.1896	9.0264	8.0282	4.2969	0
GSM519303	0	0	0	57	7.10	1	6.1	1	9.0299	5.4223	3.7240	12.5175	9.6527	8.9908	8.4340	4.7500	0
GSM519304	0	0	0	49	2.10	1	1.5	1	8.5675	7.2401	3.8872	12.5094	10.7928	9.3578	8.4166	7.1121	1
GSM519305	1	0	0	41	1.80	1	1.1	1	12.9694	4.7719	2.9380	12.4894	10.6592	8.8816	8.8745	4.5701	0
GSM519306	0	1	0	52	5.80	1	2.4	1	9.3159	11.5016	3.7760	12.2186	10.2293	9.2092	9.2445	4.8398	0
GSM519307	0	1	1	47	11.30	0	11.3	0	8.4413	11.8385	7.6909	12.4057	10.7756	8.8205	8.8509	4.0757	0
GSM519308	0	1	0	63	11.10	0	11.1	0	9.1121	10.9276	3.3375	12.2264	9.9512	8.6942	9.0118	5.1221	0
GSM519309	0	0	1	31	1.60	1	1.1	1	9.5579	8.8089	5.3191	12.3587	10.7376	9.5151	8.7872	4.5052	0
GSM519310	0	1	0	34	3.10	1	3.1	0	9.7353	10.4278	4.2067	12.9396	11.3466	9.0734	9.5674	4.6702	1
GSM519311	0	0	0	30	9.50	0	9.5	0	9.1222	9.0354	4.6632	12.2149	10.4078	8.3577	8.6926	5.3606	0
GSM519312	0	0	0	39	11.10	0	11.1	0	9.7810	5.6162	3.5427	12.4311	10.1186	8.9339	9.2136	4.4088	0
GSM519313	0	1	0	38	11.40	0	11.4	0	9.7572	11.0012	3.6300	12.6702	10.6430	8.8788	9.4735	5.5125	0
GSM519314	0	1	0	43	9.40	0	9.4	0	10.0963	11.2076	4.5928	12.1418	10.8279	9.0912	9.1701	4.1813	0
GSM519315	0	0	0	43	10.40	0	10.4	0	10.0969	5.8366	3.3979	12.2744	10.4752	8.5418	9.4658	4.6055	0
GSM519316	0	1	1	49	11.00	0	11	0	9.5003	10.1800	4.8381	11.8149	9.7924	8.2209	8.8818	4.8457	0
GSM519317	1	1	0	67	11.10	0	11.1	0	12.8358	11.9369	3.7337	12.7610	10.0812	8.2607	8.4998	5.0724	0
GSM519318	0	1	1	75	6.00	0	6	0	9.3867	11.3529	6.2926	12.9094	10.8356	9.0546	9.0330	5.0281	1

GSM519319	0	1	1	41	9.70	0	9.7	0	9.7675	10.4546	6.6471	12.5916	10.4685	9.2875	8.9640	5.6432	1
GSM519320	0	1	1	70	11.00	0	11	0	9.3619	12.5440	5.6845	12.5802	10.6148	8.4481	8.9693	4.7979	0
GSM519321	0	1	0	46	11.20	0	11.2	0	11.0420	10.4668	3.8094	12.3213	10.1342	8.8381	8.8875	5.7313	1
GSM519322	0	0	1	46	3.40	1	1	1	10.9816	9.1563	5.2656	12.3856	10.0732	9.4444	8.4277	4.5180	0
GSM519323	0	1	1	37	10.30	1	10.3	0	8.8201	11.3940	8.9597	11.8043	9.7467	8.6327	8.7027	4.6211	0
GSM519324	0	0	0	38	4.70	1	1.6	1	8.2495	5.8020	3.5835	12.2847	10.1821	9.1235	8.5584	5.0205	0
GSM519325	0	0	0	55	11.00	0	11	0	8.5987	9.1534	3.8451	12.7027	10.1069	9.0951	8.8618	5.0279	0
GSM519326	0	1	1	32	11.20	0	11.2	0	11.0232	11.6523	5.9082	12.1370	10.6197	8.6226	8.6692	6.1708	1
GSM519327	0	1	0	46	6.20	1	3.1	1	9.6274	10.0453	3.4508	12.7129	11.0075	9.0487	9.0580	5.1307	1
GSM519328	0	1	0	72	7.00	0	7	0	9.4037	11.5799	3.9505	12.8809	10.9444	8.4922	9.3854	4.9556	1
GSM519329	0	1	0	33	9.10	0	9.1	0	9.9161	11.1922	3.7095	12.0044	11.3902	9.2784	9.1836	5.5887	1
GSM519330	0	1	1	40	10.20	0	5.7	1	9.7521	10.8075	5.3106	12.5318	10.4560	9.1501	9.5160	5.3297	0
GSM519331	0	1	1	45	11.10	0	11.1	0	9.3109	11.7451	6.2065	12.3346	9.6354	8.8497	9.0830	4.7013	0
GSM519332	0	1	1	39	11.00	0	11	0	9.7839	11.4164	6.2215	12.5049	10.6067	8.6167	9.5799	4.6504	0
GSM519333	0	1	1	47	7.50	1	3.2	1	8.1861	12.9733	7.5483	11.6799	9.8515	9.0952	9.2000	4.6488	0
GSM519334	0	1	1	54	9.50	0	9.5	0	10.4939	11.4730	6.6049	13.0665	10.8816	8.9287	9.6914	5.1737	1
GSM519335	0	1	1	58	11.00	0	11	0	8.7810	10.9030	5.1321	12.6050	10.6915	8.5892	9.1902	4.3990	0
GSM519336	0	1	1	31	11.00	0	11	0	9.2345	10.4527	5.3372	12.3784	11.0808	9.5214	9.1922	5.1317	1
GSM519337	0	1	0	81	2.70	1	2.6	1	8.9738	10.9904	3.9572	12.4526	10.6580	8.8005	9.3618	4.8123	0
GSM519338	0	1	1	43	10.00	0	10	0	9.5942	11.1311	7.4912	12.2795	10.3872	9.0796	8.9364	5.5680	0
GSM519339	0	1	1	66	11.00	0	11	0	10.1597	12.3841	7.8104	12.7995	10.5190	9.5403	9.0553	5.2342	0
GSM519340	0	1	1	48	11.00	0	11	0	9.5401	12.1046	7.3382	12.3169	9.7543	7.5348	9.0465	4.8343	0
GSM519341	0	0	0	62	10.80	0	10.8	0	9.1292	9.0918	3.7694	12.3113	9.9594	9.3345	9.0241	4.8123	0
GSM519342	0	1	0	36	5.50	1	2.4	1	8.4122	9.9695	3.3796	12.8091	10.6478	9.3848	9.2700	4.6314	0
GSM519343	0	0	1	55	10.00	0	10	0	8.5267	9.1775	4.9735	12.8150	10.5023	9.0264	9.1414	5.0534	0
GSM519344	1	1	1	43	9.90	0	9.9	0	12.6074	11.4500	5.4957	12.4053	10.0524	8.3339	8.9647	4.8725	0
GSM519345	0	1	0	40	9.20	0	9.2	0	9.7556	10.7654	4.3395	12.1310	10.7926	9.2059	8.9944	6.8333	1
GSM519346	0	0	0	54	10.60	0	10.6	0	8.2954	5.8060	3.6291	11.9191	9.4363	7.8992	8.6399	4.6282	0
GSM519347	0	1	0	60	10.10	1	0.2	1	8.7380	12.4301	4.0387	12.5060	9.9651	8.8668	8.9386	4.8674	0
GSM519348	1	0	0	47	10.50	0	10.5	0	11.4306	4.7799	3.1808	12.4544	10.0161	8.5615	8.9114	3.8802	0
GSM519349	1	1	1	35	1.90	1	1.8	1	11.3332	10.6192	7.1456	12.1319	9.8710	8.9627	8.8080	4.7572	0
GSM519350	0	1	0	52	9.20	0	9.2	0	10.0610	12.7954	3.8553	12.3156	10.1117	9.2500	8.5076	5.5684	0
GSM519351	0	1	1	44	7.90	0	7.9	0	9.8945	10.7738	6.2153	12.3563	10.2501	9.0821	8.9221	5.2671	0
GSM519352	1	0	0	46	8.10	1	4.7	1	13.1199	8.6810	3.6039	12.3796	9.8461	9.1775	8.3908	5.2117	0
GSM519353	0	1	0	82	7.50	0	7.5	0	9.5949	11.6189	4.0574	12.1777	9.2336	7.2876	9.3147	4.9911	0
GSM519354	0	1	1	38	9.40	0	9.4	0	8.9550	11.3622	6.6591	12.3787	10.1564	9.1105	9.2776	5.2712	0
GSM519355	0	1	0	38	10.60	0	10.6	0	9.5344	11.0591	3.7706	12.0852	11.0515	8.8469	9.1798	5.7592	1
GSM519356	0	1	1	45	10.20	0	10.2	0	10.0737	12.4642	6.2692	12.1639	10.3328	8.7436	8.9460	5.9431	1
GSM519357	0	1	1	41	5.30	1	3.9	1	9.5738	11.2519	6.7470	12.1415	9.7630	8.6967	8.7554	5.0016	0
GSM519358	1	1	0	47	8.90	0	8.9	0	11.2778	10.2695	4.7005	12.0197	9.7352	8.9519	8.5212	5.1323	0
GSM519359	1	0	0	84	1.30	1	1.3	1	12.4764	7.1593	3.1536	12.9977	10.6538	8.7873	9.1959	4.6381	1
GSM519360	1	1	1	54	9.00	0	9	0	11.7795	11.9951	4.9153	12.6186	10.2157	8.5156	8.4557	4.6698	0
GSM519361	0	1	0	66	8.40	0	8.4	0	8.5929	13.4355	3.5768	12.8525	9.4969	8.2351	8.7188	4.5437	1
GSM519362	0	0	0	63	9.10	0	9.1	0	8.6240	6.3825	3.8010	12.3778	9.5715	8.7680	8.5602	5.8521	1
GSM519363	0	1	0	55	8.70	0	8.7	0	9.1928	9.6855	4.3749	12.0758	9.7730	9.2388	8.5567	4.0262	0
GSM519364	0	1	0	47	10.30	0	10.3	0	8.4285	10.0510	3.0467	12.2987	10.2203	9.7761	8.9395	5.4790	0
GSM519365	1	0	0	45	9.60	0	9.6	0	12.9913	7.3938	2.7851	13.1582	11.6595	9.0680	8.6481	4.8277	1
GSM519366	0	1	1	56	10.10	0	10.1	0	10.2237	11.2865	6.4245	12.0863	9.8862	8.6731	9.0982	4.8329	0
GSM519367	1	0	0	38	3.70	1	2.7	1	12.2652	5.0070	3.6000	12.2781	9.9539	8.7807	8.6438	5.7086	1
GSM519368	0	0	0	49	3.40	1	1.7	1	8.9812	9.4096	3.6766	12.8653	11.0173	8.5946	8.5318	4.4302	1
GSM519369	0	1	0	53	10.40	0	10.4	0	10.7229	13.4541	4.0313	11.9191	10.5113	9.3006	9.3712	5.1069	0
GSM519370	0	0	0	34	5.00	1	3.4	1	9.4333	8.3938	4.4823	12.3651	10.8204	9.2092	9.5442	5.0312	0
GSM519371	0	1	0	38	7.50	1	4.2	1	8.9105	10.5554	3.4024	12.5910	10.0658	9.1903	9.0753	5.5523	0
GSM519372	1	0	0	41	10.10	0	10.1	0	11.8504	7.9649	4.1471	12.5344	10.5859	8.8217	9.1473	4.5685	0
GSM519373	0	1	0	52	7.20	0	7.2	0	9.0764	10.7145	4.0522	12.2651	10.0502	9.3479	8.7916	4.2345	0
GSM519374	0	1	1	40	10.10	0	10.1	0	8.9653	11.9791	9.1796	12.9172	10.1592	8.7687	9.2323	5.0101	1
GSM519375	1	1	1	43	8.90	0	8.9	0	12.5060	11.8488	6.1859	12.4305	10.0831	9.1874	9.4951	5.6304	1
GSM519376	0	1	0	62	9.40	0	9.4	0	10.1634	12.1513	2.9109	12.6464	9.9367	9.3394	8.6120	4.9882	0
GSM519377	0	1	0	62	10.20	0	10.2	0	8.5038	12.8078	4.3917	12.6091	9.1598	8.1335	8.5607	4.7759	0
GSM519378	0	1	1	44	9.40	0	9.4	0	9.1980	12.1335	5.7318	12.6179	10.7907	8.1429	9.6550	6.3928	1
GSM519379	0	0	1	41	9.20	0	9.2	0	9.8583	9.1542	5.2209	12.6128	10.6800	8.9943	9.0605	4.7857	0
GSM519380	0	1	0	38	9.20	0	9.2	0	8.7526	10.3081	3.5975	12.2190	9.5479	8.7667	9.1796	5.4285	0
GSM519381	1	0	0	43	9.90	0	9.9	0	12.7967	9.3730	4.5157	12.8373	11.2888	8.5453	9.1635	4.3602	1
GSM519382	0	0	1	48	3.10	1	2.9	1	8.9277	7.5103	6.9579	12.6831	10.7527	9.2622	9.8357	4.7372	0
GSM519383	0	1	1	39	9.80	0	9.8	0	10.4008	12.1018	7.6710	12.2669	10.2586	9.5649	8.8710	5.3835	0
GSM519384	0	0	0	44	8.30	0	8.3	0	8.1430	9.2323	3.7280	11.8205	9.7515	8.9671	8.3007	3.9647	0
GSM519385	0	1	1	37	9.80	0	9.8	0	10.6582	10.0974	6.0001	12.1473	9.9761	8.8249	8.6453	5.0448	0
GSM519386	0	1	1	41	10.40	0	0	1	8.9913	10.7202	5.6613	12.2157	10.0917	7.4010	8.9848	5.6395	1
GSM519387	0	0	0	53	8.90	0	8.9	0	8.2400	6.5924	3.3724	11.9441	9.8347	8.7164	8.5420	4.3312	0
GSM519388	0	1	0	36	9.50	0	9.5	0	8.8313	9.6040	4.7450	12.0603	10.1669	9.1356	8.8206	5.5845	1
GSM519389	0	0	0	55	9.50	0	9.5	0	8.1711	4.6275	3.5557	11.6503	10.6095	9.3447	8.7354	5.1787	0
GSM519390	0	1	1	52	9.60	0	9.6	0	9.2275	11.2473	7.1097	12.4874	10.0978	9.3261	9.4071	4.8762	0
GSM519391	0	1	1	63	9.60	0	9.6	0	9.2301	11.5657	8.0128	12.6578	9.9488	8.7624	8.9598	4.7303	0
GSM519392	0	0	0	47	8.30	0	8.3	0	11.0006	7.2982	3.0039	12.1694	10.4874	9.3976	8.8986	3.6982	0
GSM519393	1	0	0	49	9.40	0	9.4	0	12.0831	8.7149	3.8607	12.3317	9.8690	9.3338	8.5382	5.0402	0
GSM519394	0	1	1	48	9.40	0	9.4	0	9.7125	10.4323	4.8208	12.1358	9.9908	8.4599	8.4727	4.6381	0
GSM519395	0	1	1	47	8.60	0	8.6	0	9.5996	11.6267	7.2810	12.4088	10.4608	9.1041	9.7472	4.8171	0
GSM519396	1	1	0	41	6.60	1	5.1	1	12.9555	10.3333	4.6804	12.7694	10.5548	9.6200	9.2405	4.5698	0
GSM519397	0	1	1	53	9.30	0	9.3	0	9.1691	10.8573	6.8324	12.8987	9.9867	9.7107	8.9016	5.3072	1
GSM519398	0	1	1	46	7.00	1	5.7	1	9.4083	10.3292	4.9434	12.1103	9.9811	9.3549	8.5572	4.7528	0
GSM519399	1	0	0	57	9.10	0	9.1	0	12.9501	6.8967	3.3374	12.0199	10.1650	9.5485	8.3779	4.3297	0
GSM519400	0	1	1	43	3.30	1	0.9	1	9.1210	11.7001	5.9670	12.5577	10.1511	8.4119	9.2006	4.9393	0
GSM519401	0	1	0	47	9.00	0	9	0	8.3760	12.0585	4.3393	12.3389	10.4524	8.7179	9.2246	4.5005	0
GSM519402	0	1	0	48	8.10	0	8.1	0	9.1947	10.1197	4.0047	12.3001	10.6677	9.2668	9.1520	4.0490	0
GSM519403	0	1	1	39	0.40	1	0.4	0	8.0310	11.1584	5.3650	12.5709	10.1722	8.8863	8.6438	4.5928	0
GSM519404	1	0	0	58	9.10	0	9.1	0	12.6175	7.6364	3.4912	12.4559	10.1894	9.5001	8.3173	4.8003	0
GSM519405	0	1	0	42	8.90	0	8.9	0	8.5830	11.5809	4.1150	12.1420	10.0573	8.9554	8.6632	5.1996	0
GSM519406	0	1	0	57	8.90	0	8.9	0	9.1377	12.2996	3.1874	12.5166	10.3892	9.2590	8.4502	5.1149	0
GSM519407	0	1	1	40	8.20	0	8.2	0	8.8051	10.0603	7.4105	12.6997	10.5344	9.6783	8.9994	5.2606	0
GSM519408	0	1	1	48	7.70	0	7.7	0	9.9043	10.6158	7.2923	12.4309	10.2535	9.4697	8.8827	5.1390	0
GSM519409	1	0	0	46	8.70	0	8.7	0	11.3057	9.2733	3.4214	12.2873	10.2073	9.2756	8.6523	4.5214	0
GSM519410	1	1	0	49	8.10	0	8.1	0	12.1653	11.7113	4.6083	11.9947	9.9964	8.9902	8.6426	5.5363	0
GSM519411	0	1	0	53	8.60	0	8.6	0	10.0636	11.7548	3.4910	12.6518	9.8434	8.3711	9.1280	4.7596	0
GSM519412	1	0	0	39	9.00	0	9	0	12.8039	7.1508	3.4569	12.1728	10.1590	9.1383	8.6352	5.0450	0
GSM519413	1	0	1	50	7.80	0	7.8	0	11.5925	9.4961	6.1898	12.3638	10.4301	9.5825	9.0049	4.4376	0
GSM519414	1	0	0	34	8.70	0	8.7	0	12.2649	8.2083	3.8243	12.6142	10.4307	8.6451	8.6148	4.0673	0
GSM519415	0	1	1	53	8.40	0	8.4	0	8.6756	10.6229	7.2031	12.2423	10.4326	9.3401	8.7704	4.6033	0
GSM519416	1	0	0	59	8.30	0	8.3	0	11.3781	7.7152	4.1154	12.2625	9.4217	10.1297	8.0586	4.5945	0
GSM519417	0	0	0	62	2.40	1	1.5	1	10.2317	6.8114	3.3584	12.5001	10.0593	9.4864	8.4764	4.5114	0
GSM519418	1	1	0	41	8.50	0	8.5	0	12.4485	11.1311	3.8987	12.5443	10.0813	8.4117	8.6650	4.8785	0
GSM519419	0	1	0	28	2.90	1	2.1	1	9.3780	11.3191	3.4662	13.3300	11.2403	9.4797	9.4507	5.4553	1
GSM519420	0	0	0	29	8.10	0	8.1	0	9.9130	5.9724	3.6170	12.6281	9.7958	8.9967	8.6394	4.2525	0
GSM519421	1	0	0	46	8.10	0	8.1	0	12.5779	6.9012	3.0357	12.5680	10.5122	9.5905	9.1041	4.9217	0
GSM519422	0	0	0	48	8.00	0	8	0	7.8358	7.5281	3.9134	11.8079	9.3280	10.0259	7.9448	4.6119	0
GSM519423	1	1	0	64	8.00	0	8	0	11.4304	12.9501	4.4563	12.3848	10.1633	9.1361	8.4027	5.4783	0

GSM519424				1	1	0		48	8.40	0	8.4	0	11.7141	9.7493	3.1637	12.2729	10.3155	8.9968	8.8672	3.9840	0
GSM519425				0	1	1		39	3.50	1	0	1	9.0546	9.8344	5.3122	12.2715	10.3485	8.9082	9.0991	4.4563	0
GSM519426				0	1	1		40	8.10	0	8.1	0	9.5350	11.9989	7.9575	12.2037	9.7485	9.0316	9.2870	5.4977	0
GSM519427				0	1	1		55	8.50	0	3.7	1	9.7501	10.4330	4.8757	12.4463	10.6546	9.5935	9.3294	4.8923	0
GSM519428				0	1	1		37	8.10	0	8.1	0	9.5092	11.9397	7.5281	12.4527	10.5165	9.2474	8.8965	4.9848	0
GSM519429				0	1	1		41	6.40	0	5.8	1	9.5871	10.9883	8.1245	12.8841	11.6753	9.5496	9.1181	4.3041	1
GSM519430				0	1	1		72	1.50	0	1.5	0	9.5555	12.4743	6.7900	12.6030	10.7575	9.1506	9.1459	4.2055	0
GSM519431				0	1	0		51	6.80	1	6.7	1	11.0884	10.7489	4.0227	12.8298	11.2949	8.8728	9.0328	4.1516	1
GSM519432				0	1	1		59	6.70	0	6.7	0	9.7325	13.1538	5.5948	12.9921	11.4216	8.8310	9.5194	4.4026	1
GSM519433				0	1	1		57	6.50	0	6.5	0	9.6419	11.5038	6.8894	12.4974	10.7791	8.3467	9.6356	5.0024	0
GSM519434				0	1	1		46	4.00	1	2.1	1	9.3685	12.8832	6.8070	12.4258	10.6568	7.8757	9.2029	4.1966	0
GSM519435				0	1	0		38	5.80	0	5.8	0	10.4028	11.4849	4.3325	12.6448	11.1441	8.7044	8.4373	6.0950	1
GSM519436				1	1	0		56	5.00	0	5	0	11.8180	12.5830	3.8912	12.3201	10.4530	9.4808	8.9042	4.9206	0
GSM519437				0	1	0		50	5.30	0	5.3	0	10.0213	11.3923	4.4106	12.0846	10.2785	7.8365	9.0650	4.6433	0
GSM519438				0	1	0		49	4.90	0	4.9	0	8.9985	10.0327	4.5971	12.2597	10.4909	8.8676	8.8073	4.7552	0
GSM519439				0	0	0		53	4.70	0	4.7	0	9.7744	8.6379	4.1488	12.5153	10.9747	8.9916	9.1750	3.7173	1
GSM519440				0	1	1		44	4.10	0	4.1	0	9.0798	10.1393	6.6442	12.3957	10.2735	8.7688	8.6332	4.2880	0
GSM519441				0	1	0		57	3.80	0	3.8	0	9.1098	12.8467	4.1172	12.6372	11.0812	8.4701	9.3662	4.3354	1
GSM519442				0	0	0		52	3.80	0	3.8	0	10.1626	9.3233	3.3867	12.9098	10.8929	9.0690	9.1615	5.1797	1
GSM519443				0	1	1		34	8.10	0	8.1	0	8.5698	10.3858	6.3645	12.1608	9.9895	8.7343	9.0123	4.4899	0
GSM540108	0	1	0	1	1	0	2	67			2.00	1	12.5298	10.8380	4.0437	12.5357	9.7914	8.0470	8.3411	4.4834	0
GSM540109	1	0	0	1	0	0	3	47			1.36	1	13.1239	1.9725	3.2018	12.5830	9.0687	8.6299	8.0819	4.9146	0
GSM540110	1	1	0	0	0	0	3	41			1.64	1	9.1559	9.2403	3.4070	11.5111	8.8735	9.3464	7.7782	4.5653	0
GSM540111	1	0	0	1	0	0	3	62			4.85	1	13.1513	7.4419	3.9240	11.9696	10.1231	8.9318	8.7133	4.7450	0
GSM540112	0	1	1	0	1	0	2	60			2.39	1	10.6482	12.8723	3.4393	11.9620	10.2540	8.5779	8.3022	4.6647	0
GSM540113	0	0	0	1	0	0	2	51			0.82	1	12.9111	9.4209	4.4804	11.5961	9.9507	9.1086	9.1318	5.1808	0
GSM540114	0	1	0	0	1	0	3	61			0.62	1	10.2588	10.1457	3.9915	12.1567	10.1930	8.3583	9.0797	5.6568	1
GSM540115	0	0	0	0	0	0	1	60			15.76	0	10.3132	9.2279	3.3750	12.4506	9.8108	9.0170	8.6930	5.8301	1
GSM540116	0	1	1	0	1	1	3	49			3.47	0	9.7627	12.0620	8.1814	12.3554	10.7159	10.1476	8.6664	5.3847	0
GSM540117	0	0	0	0	0	0	2	45			3.04	1	7.8590	7.7290	4.2770	12.4622	9.6745	8.4239	9.6019	6.0646	1
GSM540118	0	0	0	0	0	0	2	58			8.84	0	8.4950	7.1873	3.9236	12.5402	10.6369	10.3503	9.1241	5.1651	0
GSM540119	0	0	0	0	0	0	3	62			0.99	1	9.5774	6.5047	4.3343	11.9138	9.8257	9.8660	9.2756	5.2687	0
GSM540120	0	1	1	0	1	0	3	62			11.88	0	9.1387	13.6396	4.6978	12.5799	10.4675	9.0466	8.9004	5.2504	0
GSM540121	0	1	1	0	0	0	3	47			10.87	0	8.8069	7.5392	4.5039	12.4635	10.7982	8.8853	8.9802	5.7892	1
GSM540122	0	1	1	0	1	1	3	54			1.23	1	10.0512	13.9872	6.2293	12.3031	9.7922	8.7911	9.3202	4.6072	0
GSM540123	0	0	0	0	0	0	3	85			0.00	1	9.8250	8.0396	3.5144	12.7292	10.4304	8.4765	8.9063	4.5200	0
GSM540124	1	0	0	1	0	0	3	64			1.21	1	11.2259	6.0235	3.7464	12.4623	10.3240	9.3195	9.0765	5.3597	0
GSM540125	0	0	0	0	0	0	3	68			3.27	0	8.2932	6.9883	4.0627	11.9797	9.8305	10.2320	9.1467	5.4033	0
GSM540126	0	1	0	0	1	1	3	82			2.59	0	9.8702	11.0160	6.8266	12.2906	10.3327	9.2316	9.2919	4.5970	0
GSM540127	0	0	0	0	0	0	3	33			1.82	1	9.1909	7.4853	4.2560	12.2607	9.6438	8.5732	8.9359	6.0497	1
GSM540128	0	1	1	0	1	0	3	35			12.52	0	10.0434	12.3494	4.1940	12.3887	11.0154	7.9573	9.3682	7.2039	1
GSM540129	0	1	0	0	1	0	2	47			12.66	0	8.4133	11.6803	4.3892	12.2210	10.4853	10.0027	8.5906	5.7806	1
GSM540130	0	0	0	0	0	0	3	71			9.71	0	5.3305	5.9382	4.1117	12.0165	9.6083	9.4969	8.2578	5.2179	0
GSM540131	0	1	0	0	1	0	2	68			13.84	0	9.9228	13.4736	3.8656	12.2879	10.7761	8.6435	9.2953	5.5035	0
GSM540132	0	0	0	0	0	0	3	59			3.06	0	8.8204	8.0184	4.1867	11.8344	9.6443	8.6170	9.1325	5.4341	0
GSM540133	0	1	1	0	1	1	1	42			12.57	0	9.9287	12.1083	5.4673	11.5135	9.9653	8.9125	8.9498	4.7542	0
GSM540134	0	1	0	0	1	0	1	56			13.40	0	9.5480	12.3748	3.7586	12.6602	10.1497	9.6103	9.3458	6.3549	1
GSM540135	0	1	1	0	1	1	1	30			13.12	0	10.3240	10.2101	6.5096	12.3486	10.1268	9.0636	8.8157	5.9997	1
GSM540136	0	1	1	0	1	1	1	45			8.97	0	9.7991	11.4428	7.3340	12.2730	10.6048	9.4212	8.6225	5.4356	0
GSM540137	0	1	1	0	1	0	3	51			11.05	0	10.3402	11.9555	4.7409	12.6305	10.1393	8.7375	9.4980	5.8180	1
GSM540138	0	0	0	0	0	0	3	53			0.94	1	6.8163	5.2535	4.1951	12.8222	10.6344	8.9473	8.9969	6.1154	1
GSM540139	NA	1	0	0	0	0	2	31			11.83	0	9.9303	8.3518	3.2434	10.1814	9.5119	8.9515	8.5348	4.6575	0
GSM540140	0	0	0	0	0	0	3	49			0.91	1	9.1475	7.8425	4.4606	12.0836	10.0613	8.1240	8.4277	6.4224	1
GSM540141	0	1	1	0	0	0	1	53			5.76	0	10.7186	9.3460	4.3009	11.2454	9.2881	6.7588	8.8546	4.9679	0
GSM540142	0	1	1	0	1	0	3	64			8.28	0	9.4723	12.6606	4.4862	11.7564	10.7611	8.5841	9.5346	5.2366	0
GSM540143	0	0	0	0	1	0	1	45			6.72	0	9.2154	11.3793	3.5011	12.6376	10.8452	9.3912	8.5467	4.9592	0
GSM540144	0	0	0	0	0	0	2	42			8.09	0	10.2497	8.6295	3.9814	12.8579	10.8107	9.2465	8.7438	5.4049	1
GSM540145	0	1	1	0	0	1	2	49			5.91	0	9.8700	9.0710	7.0142	12.5421	10.9068	9.5144	9.4635	5.2813	0
GSM540146	0	0	0	0	0	0	3	69			2.67	1	9.5381	3.8530	3.2585	12.4762	10.6721	8.5367	9.1071	6.5753	1
GSM540147	NA	0	0	1	0	0	3	35			0.93	0	12.5094	4.3866	3.5929	11.9057	10.1601	8.8924	8.8815	5.9052	1
GSM540148	0	1	1	0	1	0	3	55			6.46	1	10.6394	13.2461	4.6351	12.1517	11.5343	8.8647	9.2194	8.5329	1
GSM540149	0	1	1	0	1	0	2	49			11.70	0	9.9788	10.1267	4.7887	12.4642	10.6494	8.8435	9.6578	5.1718	0
GSM540150	0	1	1	0	1	1	2	44			11.25	0	9.7490	12.5581	7.1958	12.7446	10.5246	8.9718	9.7105	5.3812	0
GSM540151	0	0	0	0	0	0	2	59			7.88	0	8.7534	6.5477	4.5677	12.3291	10.2713	9.7056	9.3470	5.2708	0
GSM540152	0	1	1	0	1	0	2	80			4.36	1	9.3135	12.5673	4.2390	11.8656	10.4834	9.0266	8.5305	5.0521	0
GSM540153	0	1	1	0	1	1	1	39			5.50	0	9.5798	10.8679	5.4636	12.6543	10.3425	9.6777	8.9458	5.3417	0
GSM540154	0	1	0	0	1	0	1	61			11.27	0	10.6030	9.7980	4.2599	11.4887	10.4194	8.9912	9.5237	4.6890	0
GSM540155	1	0	1	0	0	0	2	38			3.50	1	9.1596	7.8382	4.5232	12.3359	11.4235	9.9087	9.4858	4.9719	1
GSM540156	0	0	1	0	0	1	2	60			1.73	1	8.3041	7.6672	5.0662	12.3873	9.6931	9.6969	9.7023	6.1693	1
GSM540157	0	0	0	0	0	0	1	74			3.08	1	8.7884	7.0716	3.8430	11.7907	9.2213	9.1809	8.7313	4.1636	0
GSM540158	0	0	0	0	0	0	1	51			10.48	0	8.7106	5.9096	4.0442	12.5393	10.2877	8.8046	10.0132	4.8176	0
GSM540159	0	0	0	0	0	0	1	57			10.78	0	9.4287	8.0579	3.7792	12.3497	9.8572	9.2987	9.1490	4.7043	0
GSM540160	0	0	0	0	0	0	3	68			10.98	0	8.9846	7.7012	4.4776	12.3629	9.3756	9.0601	8.8656	5.6296	1
GSM540161	0	1	1	0	1	0	3	41			2.18	1	10.2466	11.9998	3.8477	12.8254	10.9515	8.7518	9.4245	5.1483	1
GSM540162	1	0	0	1	0	0	2	26			2.15	1	11.9721	6.8558	4.3645	12.6576	9.7930	9.8024	8.4802	5.2383	0
GSM540163	0	1	1	0	1	0	3	33			7.89	0	8.8646	9.6512	2.5403	12.3368	10.2833	9.1217	8.7175	5.1044	0
GSM540164	0	0	0	0	0	0	3	75			10.05	0	8.2588	6.3425	4.1945	12.2330	9.8835	10.0663	9.0601	5.0960	0
GSM540165	0	1	1	0	1	0	3	24			2.63	1	8.9802	11.4835	4.5716	11.8097	10.0612	9.1893	9.0193	5.4797	0
GSM540166	0	1	1	0	1	1	2	51			7.51	0	10.3256	12.6655	4.9653	12.7835	10.9783	9.1910	9.2055	6.3377	1
GSM540167	0	0	0	0	0	0	3	37			4.60	1	8.2729	7.3891	4.4128	12.1005	9.9175	9.2034	8.1267	5.5367	0
GSM540168	0	1	1	0	1	0	3	71			2.60	1	10.1218	11.3983	3.8715	11.4906	10.0211	8.7627	8.8628	5.7783	1
GSM540169	0	1	0	0	1	0	1	64			5.52	1	9.9507	11.2290	3.9334	12.5768	11.0756	8.2484	10.0243	5.8093	1
GSM540170	0	1	1	0	1	1	2	66			0.90	1	8.7459	12.5754	5.9459	12.7695	10.1906	9.0847	8.9416	5.1742	0
GSM540171	0	1	1	1	1	0	2	61			7.19	0	12.3031	10.3022	3.9638	13.1532	10.8382	9.0527	8.7545	6.3680	1
GSM540172	0	0	1	0	1	0	3	73			2.33	1	10.6336	9.9651	3.9706	12.3147	10.8616	9.9481	9.5252	4.6159	0
GSM540173	0	1	1	0	1	1	1	73			10.12	0	9.2524	11.1111	5.2177	12.3039	10.3028	8.5567	8.7638	4.7654	0
GSM540174	NA	0	0	1	0	0	3	57			9.28	0	12.6395	4.2810	2.9268	12.6380	9.7719	8.5422	8.3953	5.5404	0
GSM540175	NA	1	1	0	1	1	1	55			10.06	0	10.0650	10.9940	5.9916	12.3780	10.4072	9.0989	9.2629	6.0810	1
GSM540176	0	0	0	0	0	0	3	63			1.86	1	9.2319	5.9803	4.2544	12.7416	10.8285	9.1321	8.5384	5.5959	1
GSM540177	0	1	1	0	1	1	3	52			3.66	1	9.4106	13.0089	5.7767	12.7044	10.8741	8.7433	9.5828	5.6375	1
GSM540178	0	0	0	0	1	0	3	80			3.54	0	11.1050	11.9525	3.8370	12.5787	10.5499	8.7962	7.8577	5.7924	1
GSM540179	0	0	0	0	1	0	2	45			7.09	0	9.5546	11.3158	4.4919	12.0364	9.8853	8.2321	8.9297	5.5933	1
GSM540180	NA	1	1	1	1	0	2	62			7.95	1	12.5175	10.6316	3.9335	12.3129	9.6972	8.2929	8.4524	5.4844	0
GSM540181	0	0	0	0	0	0	3	48			9.48	0	8.6368	5.7210	3.7493	11.9463	10.3423	8.6938	8.8734	5.7210	1
GSM540182	0	1	1	0	1	1	3	68			5.73	0	10.4815	12.1009	8.2341	12.8823	10.9076	9.4891	9.4394	7.1157	1
GSM540183	0	0	0	0	0	0	2	61			0.77	1	9.1503	7.6379	4.1160	12.4836	10.4311	8.8450	8.0504	5.2792	0
GSM540184	0	1	1	0	1	1	2	66			7.66	0	9.3037	10.1193	6.9198	11.7491	9.7137	8.2973	9.2366	4.8185	0
GSM540185	NA	1	1	0	1	1	2	53			4.62	1	10.4870	10.8190	5.0915	12.9108	10.5914	8.9434	8.8645	5.4405	1
GSM540186	0	1	0	0	0	0	3	63			2.03	0	9.6190	6.5435	4.1472	12.1737	10.4629	9.2512	8.6216	4.9650	0
GSM540187	NA	1	1	0	0	0	2	84			5.82	0	9.4782	9.3973	3.9361	11.9992	9.9120	8.9935	8.6641	5.8190	1
GSM540188	0	1	1	0	1	0	2	49			9.63	0	10.0598	11.8814	4.8080	12.6291	10.5133	10.2008	8.9379	5.6948	1
GSM540189	0	1	1	0	1	1	2	50			9.94	0	8.5713	11.1881	5.8725	11.8709	9.3018	9.0149	8.4086	5.1919	0
GSM540190	0	1	1	0	1	1	1	60			9.83	0	9.5246	10.9300	6.6873	11.6612	9.7865	9.8381	8.7475	5.6399	1
GSM540191	0	1	1	0	1	1	1	54			1.90	0	9.9516	9.6669	7.4023	12.0984	10.1307	9.2692	8.6324	5.5912	1
GSM540192	0	1	1	0	1	1	1	44			6.21	0	8.7605	11.0593	8.0742	12.9800	10.2506	8.7705	8.8354	4.5797	1

GSM540193	0	1	1	0	1	1	2	73	5.62	0	9.5777	13.0193	10.5584	12.4963	11.0623	9.3937	9.6795	6.5133	1
GSM540194	0	1	1	0	1	1	1	53	9.56	0	9.8446	10.2170	5.3003	12.1759	10.5011	8.4666	9.4556	6.1195	1
GSM540195	0	0	0	0	0	0	3	80	9.16	0	8.3856	6.3172	3.4049	10.8921	9.3973	10.1070	8.5702	4.7744	0
GSM540196	0	1	1	0	1	1	3	39	2.36	1	9.0214	11.7784	7.7385	12.2128	10.4179	8.7743	8.9661	6.3591	1
GSM540197	0	1	1	0	1	1	1	78	7.48	0	9.8114	10.9327	7.1062	12.2308	10.4947	9.0008	8.9497	5.6264	1
GSM540198	0	1	1	0	1	0	1	66	7.14	0	9.6670	11.6929	4.4608	11.4459	10.1625	9.2780	8.4765	5.5642	0
GSM540199	0	1	1	0	1	0	2	56	2.92	1	8.9344	13.4667	4.0587	12.7181	10.3184	9.5998	7.9715	5.5997	1
GSM540200	0	1	1	0	1	0	2	66	1.28	0	8.4824	10.6351	4.3616	12.3264	10.1231	8.9979	9.3793	4.6321	0
GSM540201	0	0	0	0	0	0	2	49	2.00	1	9.1261	4.6104	3.0639	12.9961	11.7922	8.8478	9.0844	5.3032	1
GSM540202	0	0	1	0	1	1	1	53	5.30	0	9.7089	11.5620	7.0767	12.1538	9.8195	9.6893	9.0433	5.4521	0
GSM540203	0	1	1	0	1	1	2	55	6.16	0	8.9698	9.7157	4.9946	11.5521	9.5453	8.9145	8.4788	5.5712	1
GSM540204	0	1	0	0	1	0	1	48	9.30	0	10.8683	10.0068	3.0561	12.4484	10.1941	8.1099	8.6060	5.6477	1
GSM540205	0	1	1	0	1	1	2	61	2.55	0	9.4325	12.1003	7.1087	12.3944	10.6831	9.1918	8.9839	6.9136	1
GSM540206	1	0	0	1	0	0	3	39	4.28	0	12.8654	6.4103	4.2159	12.3649	9.8666	8.8055	8.6670	5.6349	1
GSM540207	0	1	1	0	1	1	1	66	2.13	1	9.1669	10.9640	6.2562	11.3330	9.9128	8.6207	9.1133	5.3857	0
GSM540208	0	1	0	0	1	0	1	54	6.70	0	9.5285	10.5009	4.0133	12.3356	10.1937	9.3110	8.5050	4.8322	0
GSM540209	0	0	0	0	0	0	3	51	9.17	0	8.7489	8.7181	4.2602	12.0345	10.4153	9.5748	8.6731	5.4404	0
GSM540210	NA	1	1	0	1	0	1	60	8.71	0	10.9374	11.4591	3.7235	12.0586	10.1570	8.5870	9.0571	6.1752	1
GSM540211	NA	1	1	0	1	0	2	54	8.35	0	9.9898	10.7706	3.7095	11.7828	9.5926	8.6161	8.9720	5.5249	0
GSM540212	0	1	0	0	1	0	1	81	7.47	0	9.6308	10.2809	3.1083	11.5615	9.6837	9.2916	8.7098	5.2055	0
GSM540213	0	1	1	0	1	1	3	50	5.54	1	9.6191	9.7271	6.2797	12.0645	10.0507	8.4341	8.3253	5.5777	1
GSM540214	0	0	0	0	0	1	3	68	5.19	0	7.3084	8.9126	5.0983	12.4566	9.2016	9.2767	8.0100	4.9766	0
GSM540215	0	0	0	0	0	0	3	46	8.46	0	8.4722	7.8953	4.5945	12.8265	10.4946	9.7885	8.4129	5.5132	1
GSM540216	1	1	0	1	0	0	3	70	4.46	0	12.5240	9.2890	4.6855	12.4606	10.6670	8.8708	8.7756	5.7836	1
GSM540217	0	1	1	0	1	0	2	49	5.84	0	9.2251	9.6887	3.9927	12.2508	10.4433	8.0416	9.1549	6.6215	1
GSM540218	0	1	1	0	1	1	1	61	8.62	0	9.4637	10.7326	7.9987	11.4231	9.6122	8.6554	9.0569	5.1716	0
GSM540219	0	0	0	0	0	0	3	49	8.94	0	8.6523	6.9536	4.1433	12.3174	10.1224	10.0268	8.6047	4.8262	0
GSM540220	0	1	1	0	1	0	2	65	4.67	0	10.0746	12.0710	4.2786	12.4312	10.3646	9.2119	9.5485	6.2786	1
GSM540221	0	1	1	0	1	1	3	46	3.27	1	9.1858	11.7785	4.9733	12.8930	10.3534	6.3328	9.5354	5.8093	1
GSM540222	NA	1	1	0	1	1	1	79	6.42	0	10.0559	11.2814	7.1061	12.0097	10.2813	9.7921	9.6370	5.9639	1
GSM540223	0	1	1	0	1	0	1	55	8.94	0	8.8927	11.9340	4.7924	12.6895	11.0087	10.1126	10.2242	5.6066	1
GSM540224	0	1	1	0	1	0	3	53	8.56	0	10.9047	13.3543	4.3001	11.2369	10.3181	9.2943	8.6277	5.7921	1
GSM540225	0	1	1	0	1	1	2	75	7.70	0	9.3057	11.4116	5.1084	12.2252	10.8443	8.7445	9.4707	6.1022	1
GSM540226	0	1	1	0	1	0	2	60	6.14	0	9.4442	12.8845	3.9006	12.7042	10.6220	8.7601	9.2372	5.1308	0
GSM540227	NA	1	1	0	1	0	2	60	8.13	0	8.9533	10.5505	4.4415	11.8889	9.3517	9.2939	8.6581	4.9504	0
GSM540228	0	1	1	0	1	1	3	78	4.28	0	8.8363	12.2959	7.0912	12.5498	10.3473	9.0755	8.9591	5.1164	0
GSM540229	0	1	0	0	1	0	2	65	7.86	0	9.3240	13.1590	3.7456	13.1477	11.1901	8.8621	9.2080	5.4118	1
GSM540230	0	1	1	0	1	0	2	53	5.04	0	10.6899	10.0192	3.1474	12.7302	11.0597	8.9607	9.2455	5.8351	1
GSM540231	0	1	1	0	1	1	2	78	4.29	0	11.0145	10.4328	4.9411	9.8161	9.4317	8.0656	10.0665	5.1066	0
GSM540232	0	1	1	0	1	1	1	47	5.73	0	9.0185	11.1501	7.1458	12.0018	9.8530	9.1011	8.5984	4.9271	0
GSM540233	0	1	1	0	1	1	2	64	7.81	0	9.9179	11.1846	5.0538	11.9559	10.2202	8.5404	9.1667	5.7013	1
GSM540234	0	1	1	0	1	1	2	76	8.36	0	10.1740	13.3624	5.8321	12.3767	10.6173	8.8620	8.3334	5.0593	0
GSM540235	1	0	0	1	0	0	3	46	5.24	1	12.4371	9.2391	4.4385	12.4346	11.0838	9.1546	8.3673	5.4571	1
GSM540236	1	1	1	1	1	0	3	60	8.14	0	13.8931	9.8053	4.8164	12.1716	10.0145	9.2776	8.9077	6.4814	1
GSM540237	0	0	0	0	0	0	2	72	5.07	0	8.4544	7.6556	3.8802	12.0009	11.1698	10.1607	8.6233	5.7897	1
GSM540238	0	0	0	0	0	0	3	42	1.45	1	9.0128	6.8123	4.6783	11.7754	9.7214	9.4468	8.9385	5.7426	1
GSM540239	0	1	1	0	1	1	2	51	8.30	0	9.0069	12.6955	4.9488	12.2549	10.1846	8.6103	8.5436	5.5656	0
GSM540240	0	1	1	0	1	1	1	45	7.84	0	10.0752	10.4220	4.9589	12.4219	10.2053	9.6308	8.6540	5.4725	0
GSM540241	0	1	1	0	1	0	2	60	6.34	1	10.2516	11.3282	4.7457	11.5873	9.6592	8.4499	8.6248	5.4699	0
GSM540242	0	0	0	0	1	0	1	54	4.14	0	9.6600	9.9200	3.7156	12.4952	10.7670	9.7057	9.1161	6.1319	1
GSM540243	0	1	1	0	1	0	2	67	4.45	1	10.4723	12.4793	4.7706	12.6524	10.5428	8.6148	8.7180	5.6107	1
GSM540244	0	1	1	0	1	0	1	63	7.95	0	10.1295	10.2862	3.4839	12.1381	10.2894	9.0573	9.0619	5.8810	1
GSM540245	0	1	1	0	1	1	2	43	7.11	0	9.4327	14.0376	5.4948	12.9759	11.1395	10.4349	9.2040	5.7583	1
GSM540246	0	1	1	0	1	1	3	69	3.08	1	9.7594	11.8210	9.8280	12.2909	10.1797	8.4262	9.0948	6.1173	1
GSM540247	0	1	1	0	1	1	2	51	3.88	0	10.9028	12.0413	6.3840	12.4472	10.4808	9.8422	8.9751	5.7440	1
GSM540248	0	0	0	0	1	0	3	43	4.08	0	8.9030	9.9358	4.0435	12.1955	9.9620	9.3382	8.3499	6.1164	1
GSM540249	0	0	0	0	0	0	3	70	7.95	0	9.9141	7.3210	4.4263	11.7515	10.3654	8.0767	8.9037	5.9006	1
GSM540250	0	0	0	0	0	0	3	61	8.01	0	9.7821	7.0635	3.6710	11.8615	10.1041	9.1351	8.2070	6.6786	1
GSM540251	1	0	0	1	0	0	2	47	7.47	0	12.0357	8.4451	4.6074	12.6798	10.0846	10.1735	9.4618	4.9573	0
GSM540252	NA	0	0	1	0	0	2	59	3.37	1	12.3982	4.7702	2.8842	11.7677	10.2964	9.6964	8.6258	5.7112	1
GSM540253	0	1	1	0	1	1	3	43	2.34	1	9.3797	12.6042	8.0314	12.0642	10.3894	9.3573	9.7596	6.4378	1
GSM540254	0	0	0	0	0	0	3	44	3.76	0	9.4514	6.6645	3.8342	12.7008	10.6647	9.0209	9.4105	6.3332	1
GSM540255	0	0	0	0	0	0	2	61	0.46	0	8.9315	8.9291	4.0233	11.8423	10.1145	9.5584	9.9288	5.9081	1
GSM540256	0	0	0	0	0	0	3	40	5.66	0	9.7489	7.7386	4.6164	12.3655	9.6452	8.5730	8.4502	5.2251	0
GSM540257	1	1	1	1	1	0	3	52	6.71	0	13.1058	10.0432	4.2081	12.4917	10.0426	8.9596	8.4795	4.4994	0
GSM540258	0	1	1	0	1	1	2	71	5.58	0	9.6705	12.7777	6.2523	12.7025	10.9799	9.3613	9.5347	5.4054	1
GSM540259	0	1	1	0	1	0	2	63	6.84	0	8.4008	12.1527	4.0881	11.8610	9.8041	9.0540	8.9028	4.4512	0
GSM540260	0	0	0	0	0	0	3	75	7.00	0	10.0068	7.8455	4.2872	11.9736	10.1039	8.7853	9.3109	5.3366	0
GSM540261	0	0	0	0	0	0	3	74	5.17	0	9.2704	8.1014	4.7840	12.6970	10.2722	8.9505	9.2018	5.3062	0
GSM540262	0	1	1	0	1	0	2	66	4.50	0	9.6628	12.9812	3.7382	12.5387	10.2820	9.6477	8.7759	5.2218	0
GSM540263	0	0	0	0	0	1	2	37	4.86	0	9.7432	8.4856	5.9434	12.5070	10.4682	9.1546	8.6893	4.7616	0
GSM540264	0	0	0	0	0	0	3	59	3.93	0	9.1219	6.1690	4.0785	12.5482	10.6713	10.6218	8.8680	5.8367	1
GSM540265	1	0	0	1	0	0	3	61	4.87	0	11.2298	7.9024	4.2460	12.8264	10.5177	8.2638	9.3382	5.9284	1
GSM540266	0	1	1	0	1	1	2	60	4.57	0	10.0645	13.7161	6.6050	12.4893	10.6129	9.9504	9.4698	7.7925	1
GSM540267	0	1	1	0	0	0	1	49	1.32	1	9.7524	7.0748	4.1691	11.8272	9.5717	7.9126	9.3923	5.2833	0
GSM540268	1	0	0	1	0	0	3	61	4.08	0	13.0782	7.8173	4.4079	12.6375	10.2739	8.8222	8.5192	4.6064	0
GSM540269	0	1	1	0	1	1	3	34	3.64	1	9.0200	12.3712	5.3892	12.6754	11.0687	8.7196	10.1867	4.8873	1
GSM540270	1	1	1	0	1	1	3	69	4.25	0	10.7454	13.6141	4.9348	11.9918	10.1654	8.8329	9.1800	6.7107	1
GSM540271	0	0	0	0	0	1	3	47	5.51	0	8.8456	7.2288	4.8911	12.1688	10.4602	7.9416	9.9008	5.1195	0
GSM540272	1	0	0	1	0	0	3	53	4.31	0	12.1572	7.8378	4.0208	12.3517	9.8964	8.6770	8.5643	4.9569	0
GSM540273	0	0	0	0	0	0	3	60	0.00	1	8.4845	8.5727	4.1609	11.9464	9.2068	9.1098	8.5548	5.5079	0
GSM540274	1	0	0	1	0	0	3	72	0.96	1	13.2614	7.2100	4.0410	12.6390	10.1475	9.2211	9.0041	5.2471	0
GSM540275	0	1	1	0	1	0	2	48	4.96	0	9.5655	10.7250	4.5862	11.6906	10.2130	9.0036	9.2888	5.1947	0
GSM540276	0	0	0	0	1	0	3	63	1.12	1	9.3030	10.3076	4.4463	12.3710	10.3887	10.2758	9.7868	5.0832	0
GSM540277	0	1	1	0	1	0	3	66	4.93	0	9.3684	14.1251	4.7135	12.6007	9.7988	8.8763	8.8038	5.0987	0
GSM540278	0	1	1	0	1	1	3	69	1.89	0	8.8328	12.1973	9.5217	12.3983	10.9812	9.5938	9.0486	6.4706	1
GSM540279	0	1	1	0	1	1	2	40	4.98	0	9.3458	10.6374	8.9077	12.5164	10.1125	9.1269	9.2153	5.9978	1
GSM540280	1	1	1	1	1	1	3	51	4.89	0	12.1883	11.7763	5.1607	12.3425	10.5633	9.2610	9.1449	4.7694	0
GSM540281	0	0	0	0	0	0	3	50	4.37	0	9.3681	6.7794	4.0469	12.4691	10.3995	8.7419	8.6064	5.1860	0
GSM540282	1	0	0	1	0	0	3	50	4.79	0	11.2743	5.7661	4.0096	12.0575	9.9625	8.0491	9.7007	4.9451	0
GSM540283	0	1	1	1	1	1	3	54	4.76	0	11.2130	13.2658	6.7975	12.1223	10.0303	8.6226	9.2351	5.9976	1
GSM540284	0	1	1	0	1	0	3	42	4.56	0	9.0679	12.9802	4.3281	12.4690	10.4907	8.8522	9.5658	5.5756	1
GSM540285	0	0	0	0	0	0	3	38	4.78	0	9.3481	7.5036	4.2393	12.2931	9.9661	9.4330	8.7335	7.1145	1
GSM540286	0	1	1	0	1	1	2	31	3.58	0	10.4604	9.7412	5.3864	12.4681	10.4543	8.9385	8.6601	5.4969	0
GSM540287	0	1	1	0	0	1	2	32	2.95	0	10.5250	9.2353	5.0650	12.7106	11.0546	8.6168	9.0241	5.8031	1
GSM540288	0	0	0	0	0	0	3	45	4.32	0	8.6434	5.8070	4.1554	12.3439	8.6956	8.4104	8.9656	4.9697	0
GSM540289	NA			1	0	0		32	2.56	0	13.1579	4.5214	3.2459	12.4982	10.1862	9.0888	8.5936	6.2823	1
GSM540290	NA	0	0	0	0	0	3	31	3.14	1	7.1608	4.9586	3.1427	11.4004	10.1791	8.6499	8.8886	5.8428	1
GSM540291	1	0	0	1	0	0	3	34	3.41	0	13.0683	7.4197	3.5663	12.3999	10.3393	9.3273	8.6773	5.4366	0
GSM540292	0	0	0	0	0	0	3	35	2.98	0	8.0033	7.2521	3.4464	12.0077	10.3365	8.4866	8.3474	5.2043	0
GSM540293	0	0	0	0	0	0	3	31	2.80	1	9.3271	7.5909	4.2645	12.1687	10.2838	9.3059	8.8707	6.0815	1
GSM540294	0	0	0	0	0	0	3	44	2.37	0	7.8266	9.4787	4.5360	12.2774	10.4809	8.7686	9.3281	5.1117	0
GSM540295	1	1	1	0	1	0	2	33	2.73	0	10.6407	9.5248	3.8280	12.7166	11.0702	9.7793	8.5953	6.7319	1
GSM540296	0	1	1	0	0	1	2	34	2.69	0	9.8905	8.2592	4.9677	11.9158	10.4274	8.5081	8.8995	6.1648	1
GSM540297	1	1	1	1	1	1	3	32	2.72	0	12.2192	9.7575	5.1747	12.5580	10.2934	8.5725	9.2425	5.3827	0

GSM540298	0	1	1	0	1	1	3	74	0.00	1	8.8443	12.7702	6.7697	13.1801	10.7655	10.2798	9.2843	5.4236	1
GSM540299	0	1	1	0	1	1	2	35	1.63	0	10.2187	11.6128	5.1168	12.0502	10.9587	9.4455	9.1550	6.4963	1
GSM540300	1	0	0	1	0	0	2	31	1.62	0	13.0415	6.3462	3.2470	12.2749	10.0410	9.8250	8.4919	5.9936	1
GSM540301	1	1	1	0	1	1	3	33			9.6347	10.5272	7.6625	11.4703	9.6025	8.6546	8.8116	5.5039	0
GSM540302	0	0	0	0	0	0	3	54	1.62	1	8.4425	3.8451	3.1534	12.3278	10.2870	9.3506	9.6501	5.6670	1
GSM540303	0	1	1	0	1	0	3	29			7.6839	11.5263	3.7400	12.0983	10.5467	9.2083	8.0793	6.8349	1
GSM540304	0	1	0	0	0	0	3	33	0.68	0	8.1356	8.8865	3.1445	12.4100	9.7677	10.4359	10.0363	6.6365	1
GSM540305	0	1	1	0	1	1		32			9.1866	11.3341	5.9527	12.2744	10.4306	9.5650	8.4062	5.7076	1
GSM540306	0	1	1	0	1	0	2	29			9.2063	11.2489	3.9386	11.9805	10.2043	8.4100	8.9944	6.2629	1
GSM540307	0	0	1	0	0	0	3	29	0.83	0	9.4910	7.7003	3.7769	12.0970	9.6569	8.7928	8.8437	6.4727	1
GSM540308	0	0	0	0	0	0		28			8.4098	8.7500	3.9855	11.7028	10.4065	9.2640	8.1846	5.7071	1
GSM540309	NA	0	0	1	0	0	3	33			13.1028	5.4326	3.4552	12.6378	9.9685	9.3104	9.0201	6.2911	1
GSM540310	0	1	0	0	0	0	3	34	2.13	0	8.4248	8.1210	3.7419	11.8412	9.4038	9.6610	8.5824	5.5185	0
GSM540311	1	1	1	1	1	0	3	34			11.2241	12.4101	4.7550	12.0187	9.7129	9.7436	8.3119	5.3107	0
GSM540312	NA	0	1	0	0	0		34	0.00	1	8.5282	3.7919	3.1769	11.8019	9.8872	8.7496	8.7395	5.3566	0
GSM540313	0	1	1	0	1	0	2	32			10.0791	11.1479	4.6248	11.6054	9.4888	9.7767	8.7039	6.2559	1
GSM540314	0	0	0	0	0	0	2	56			8.4028	7.8008	3.6596	11.6652	10.0996	8.4275	8.2247	6.2713	1
GSM540315	1	1	1	1	1	1	1	34			11.1910	11.1830	6.7878	11.7086	10.0185	9.3398	8.3060	5.8130	1
GSM540316	0	1	1	0	1	0	2	35			9.4917	11.5868	4.1511	12.6978	10.5848	8.9761	8.9597	6.2450	1
GSM540317	0	1	1	0	1	1	1	64	0.59	0	10.6104	12.4160	6.7510	11.5297	10.0496	8.6275	8.7970	5.7326	1
GSM540318	0	1	1	0	1	1	1	58	0.61	0	9.3163	12.5277	4.9524	12.3588	10.9676	8.9679	8.8552	6.0520	1
GSM540319	0	1	1	0	1	1	1	54	0.71	0	10.0514	12.6281	6.0017	12.2532	10.5287	9.5440	8.6112	4.8949	0
GSM540320	0	1	1	0	1	0	2	59			8.9504	13.6057	3.9048	12.6099	10.8068	9.1420	8.9838	5.4258	0
GSM540321	0	1	1	0	1	1	1	79			10.0838	11.1290	5.0067	12.2628	9.9866	8.7131	8.3741	5.6487	1
GSM540322	0	1	1	0	1	0	2	58	0.71	0	9.3906	13.0047	4.7561	11.6671	10.0251	9.0462	8.7548	5.2384	0
GSM540323	NA			1	0	0					12.2679	6.8622	4.2095	12.5462	10.2729	9.1718	8.1811	5.0487	0
GSM540324	NA			0	1	1		49	18.52	0	10.1025	13.9275	8.7446	11.9352	10.3926	8.2110	8.9284	5.3330	0
GSM540325	0	1	0	0	0	0	3	71	1.73	0	9.9259	9.0318	4.1739	12.2541	10.0431	8.3970	8.3394	5.6240	1
GSM540326	NA	1	1	0	1	0	3	35	9.48	1	10.6479	10.8356	3.7600	12.1099	11.0693	8.8888	8.5103	5.7744	1
GSM540327	NA	0	0	1	1	0	2	63	14.29	1	12.6375	10.1849	3.9051	12.0282	10.1347	8.7616	8.6892	5.6425	1
GSM540328	0	1	0	0	1	0	2	60	2.74	1	9.3160	10.9092	4.0105	11.8079	10.6424	8.7479	9.3965	5.1711	0
GSM540329	0	1	1	0	1	1	2	66	2.34	1	9.7334	12.0350	8.5918	12.1652	10.2035	8.7716	7.7731	5.3445	0
GSM540330	0	1	1	0	1	0	2	53	7.66	1	9.6683	10.9568	3.6723	12.5926	10.8497	9.8037	9.1960	6.5686	1
GSM540331	1	1	1	0	0	0	2	52	0.52	1	9.7245	7.6979	4.0466	10.8206	9.6173	8.8626	8.7323	5.4346	0
GSM540332	0	1	1	0	0	1	3	39	3.13	1	9.0807	9.0964	6.1478	12.0311	9.4572	8.8568	8.5422	5.4353	0
GSM540333	0	1	1	0	1	1	2	74	8.95	1	10.1069	12.1124	7.0087	12.4450	10.3383	9.7294	9.0612	4.8990	0
GSM540334	0	1	1	0	1	1	1	74	5.02	1	9.4246	12.3964	8.3695	12.2646	10.2048	9.7098	9.3101	5.9723	1
GSM540335	NA	1	1	0	1	1	1	53	2.11	1	9.3737	11.8247	5.2986	11.7374	10.1379	9.2468	8.4812	5.1029	0
GSM540336	0	1	1	1	1	1	2	44	6.62	1	12.9298	12.0979	5.6586	12.4299	10.5690	9.5723	8.9636	6.3860	1
GSM540337	0	1	1	0	1	1	1	57	6.33	1	9.3512	12.6328	7.3275	12.2790	10.6693	9.3844	9.1774	5.7662	1
GSM540338	0	1	1	0	1	1	2	37	2.68	1	9.4014	10.5564	5.9606	11.3527	10.5024	9.8383	9.3870	5.6334	1
GSM540339	0	0	0	1	0	0	2	53	2.50	1	12.3520	5.2008	3.6726	12.4047	10.2664	10.3737	8.8151	5.7657	1
GSM540340	1	1	1	1	1	1		37	4.24	1	12.0628	11.2547	5.4784	12.2764	10.3669	9.2233	8.9284	5.5340	0
GSM540341	0	1	1	0	1	1	1	69	3.55	0	9.1673	13.7775	8.0876	12.0589	10.3277	9.7517	9.3863	5.9603	1
GSM540342	0	1	1	0	1	1	3	69	7.43	0	9.6538	12.7976	7.4207	12.5297	11.0784	9.8354	10.0336	6.8032	1
GSM540343	0	1	1	0	1	1	3	45	1.04	1	8.6871	11.9898	4.9563	13.3731	10.7144	9.6721	9.1141	5.7539	1
GSM540344	0	1	1	0	1	1	2	83	3.39	1	10.3630	11.9086	5.6831	12.0752	10.2698	9.7945	8.7219	6.1951	1
GSM540345	0	0	0	0	0	0	3	66	2.54	1	8.6360	7.0440	4.0673	12.3781	9.8557	8.9479	8.8064	5.4421	0
GSM540346	0	0	0	0	0	0	3	53	1.88	1	10.6061	7.7368	4.2579	12.2982	10.2658	8.4681	8.1763	5.9529	1
GSM540347	1	1	1	0	1	1	2	59	2.28	1	10.6542	13.0874	5.6802	12.0488	10.0087	8.9891	8.3676	5.3837	0
GSM540348	0	0	0	0	0	0	3	67	7.76	0	8.2033	5.8883	3.7280	12.1131	10.2558	9.1030	8.6842	5.8724	1
GSM540349	0	0	0	0	0	0	2	75	1.48	1	10.0140	6.5771	3.2006	12.0397	10.1924	8.5685	8.9422	5.3344	0
GSM540350	0	0	0	0	1	0	2	79	0.28	1	10.3209	9.7653	3.3298	11.6121	9.5695	7.3600	8.5843	4.6659	0
GSM540351	0	0	0	0	0	0	3	67	3.00	1	9.1976	3.5791	3.1984	12.4606	10.1876	8.1033	9.3340	5.7508	1
GSM540352	0	0	0	0	0	0	3	56	5.20	1	9.2982	5.3660	3.6446	12.4597	10.6253	8.5191	8.9137	4.9684	0
GSM540353	0	0	0	0	0	0	3	69	4.12	0	10.0270	4.9770	3.3335	12.0280	10.4709	9.0557	8.9554	5.3810	0
GSM540354	0	0	0	1	0	0	2	58	4.56	0	13.1112	8.8913	3.7656	11.8841	9.7175	8.1257	8.4625	5.2079	0
GSM540355	0	0	0	0	0	0	3	84	1.17	0	8.5687	6.5672	3.5156	11.8576	9.7554	8.6956	8.4798	4.8887	0
GSM540356	0	0	0	0	0	0	3	42	4.54	0	9.3181	6.6197	3.8027	11.6941	9.8884	7.6683	8.9860	6.4607	1
GSM540357	0	0	0	0	0	0	3	50	4.60	0	8.8668	7.0839	3.6931	11.8189	9.2752	9.2684	9.2348	5.8879	1
GSM540358	0	0	0	0	0	0	3	62	3.49	0	10.0177	7.2485	3.4503	12.8690	10.8625	9.2656	9.1988	5.7613	1
GSM540359	0	0	0	0	0	0	2	36	2.94	0	8.9020	8.3804	4.0269	12.6890	10.4554	8.8707	9.7329	6.2560	1
GSM540360	0	0	0	0	0	0	3	65	3.13	0	8.7341	6.3760	3.9643	12.2195	9.9355	9.3170	9.4138	5.5193	0
GSM540361	0	0	0	0	0	0	3	51	3.15	0	9.1286	7.3688	3.5065	12.1290	10.6138	9.0391	9.1671	5.6057	1
GSM540362	0	0	0	0	0	0	3	42	2.97	0	7.4937	5.7919	3.6273	11.9062	10.0303	8.1646	9.4995	4.9890	0
GSM540363	0	0	0	0	0	0	3	69	2.57	0	9.4140	9.1652	3.1756	11.5706	10.0916	9.1821	9.3722	5.3242	0
GSM540364	0	0	0	0	0	0	2	53	2.19	0	10.3645	7.6467	3.7650	12.4725	10.0664	8.7995	8.7880	5.0195	0
GSM540365	0	0	0	0	0	0	3	40	2.26	0	9.1778	8.9515	4.1718	11.7327	10.1904	9.0704	9.8803	4.8008	0
GSM540366	0	0	0	0	0	0	3	75	1.17	1	8.9308	4.3518	3.4321	12.2387	10.8868	8.9831	9.9217	6.3566	1
GSM540367	0	0	0	0	0	0	1	44	1.90	0	9.5890	4.4461	3.3256	12.1738	10.2725	9.0409	9.1962	5.3650	0
GSM540368	0	0	0	0	0	0	3	37	0.62	0	8.9286	4.3242	3.5027	12.2819	10.0049	9.6834	9.1408	5.9362	1
GSM540369	0	0	0	0	0	0	3	52	0.68	0	7.9434	3.9064	3.2089	12.3086	10.9609	9.8130	9.5964	5.2882	1
GSM540370	0	0	0	0	0	0	3	70	0.65	0	9.7225	6.4741	3.5554	12.2467	9.5695	9.1624	8.2772	5.6757	1
GSM540371	0	0	0	0	0	0	3	61	1.00	1	8.5807	6.2448	3.4108	12.4801	10.8285	10.1348	9.9606	6.6368	1
GSM540372	0	0	0	0	0	0	3	64	0.96	0	9.2302	5.2080	3.4052	11.9265	11.0358	9.2408	8.6887	5.1804	1
GSM540373	0	0	0	0	0	0	3	73	0.64	0	8.5260	8.3281	3.7526	12.3557	10.6638	8.9506	9.5990	6.2391	1
GSM547768		1		0	1	1		65			10.3731	12.1101	7.5984	11.8245	10.2493	8.3381	8.9697	4.7385	0
GSM547769		1		0	1	1		60			7.5666	10.6896	7.3678	11.7533	9.3650	7.5334	8.4273	5.1208	0
GSM547770		1		1	1	0		65			11.4117	11.6904	3.1470	11.8165	10.0337	7.7905	8.0953	5.3729	0
GSM547771		1		0	1	0		76			9.4121	10.6473	2.4353	11.6941	9.4567	7.9392	8.1761	4.6903	0
GSM547772		1		0	1	1		67			10.1274	10.7318	7.2744	11.8432	9.6940	8.2165	8.3742	4.7710	0
GSM547773		1		0	1	1		72			9.4503	11.1713	7.2670	12.1655	10.2961	8.2872	8.8885	4.9544	0
GSM547774		1		0	1	0		73			8.9835	10.1785	2.8807	11.8827	9.3548	7.8472	8.4037	4.7961	0
GSM547775		1		0	1	1		74			9.1828	10.1793	6.8569	11.9855	10.4612	8.3314	8.7734	5.0440	0
GSM547776		1		0	1	0		57			10.4158	10.7072	2.5252	12.2290	11.0913	9.5310	9.0793	5.5192	1
GSM547777		1		0	1	0		69			10.2318	10.5063	3.8125	12.0989	10.2267	8.5055	9.4516	4.7556	0
GSM547778		1		0	1	0		76			10.4671	10.8088	2.6087	11.6787	9.3091	9.1830	8.2058	4.7118	0
GSM547779		1		0	1	0		55			10.2800	11.4086	3.0107	12.2536	10.5172	8.6133	8.1113	5.0865	0
GSM547780		1		0	1	0		52			10.6670	11.1356	3.0372	12.4326	10.7408	8.6563	8.5071	5.1678	0
GSM547781		1		0	1	0		68			10.0230	11.6077	4.1497	12.2594	11.0678	8.4125	8.9705	6.0229	1
GSM547782		1		0	1	0		69			10.8913	11.4280	2.8197	12.2266	10.4189	8.1316	9.1709	4.6924	0
GSM547783		1		0	1	1		70			9.6041	10.9119	5.7383	11.8786	9.7603	8.2647	8.9632	5.1312	0
GSM547784		1		0	1	1		62			8.1848	11.1894	6.1076	12.4845	10.8420	8.8445	9.1232	5.3146	0
GSM547785		1		0	1	0		78			8.7367	11.8960	3.3069	11.8084	11.3477	8.3819	8.8228	5.1532	1
GSM547786		1		1	1	0		64			11.1979	11.2598	3.2185	11.8842	10.2029	7.2382	8.6362	5.2197	0
GSM547787		1		1	0	0		64			12.9852	8.2752	2.3359	12.4744	9.9213	9.3251	8.5153	4.9441	0
GSM547788		1		0	1	1		74			9.7162	10.4381	6.4909	12.0364	9.5996	8.2138	8.8232	5.0995	0
GSM547789		1		1	1	0		53			12.7704	9.6270	2.7246	12.0465	10.4528	9.0961	8.2223	5.0630	0
GSM547790		1		0	1	0		75			9.4590	9.9366	2.5063	11.6468	9.6274	8.5707	8.7555	4.8556	0
GSM547791		1		0	1	0		78			10.1458	11.5210	3.4317	11.9219	10.3236	8.4964	9.2271	5.4828	0
GSM547792		1		0	1	0		79			10.2263	11.4932	2.6073	12.6251	10.8632	9.9172	8.9326	5.6540	1
GSM547793		1		0	1	1		61			9.8554	11.3219	7.1219	11.9807	10.0373	8.4129	9.0840	5.1695	0
GSM547794		1		0	1	1		71			10.0012	10.3873	5.4498	11.9827	10.0909	9.0863	8.4716	5.0228	0
GSM547795		1		0	1	1		70			8.6313	12.7190	6.4526	11.9585	9.2222	8.2330	9.0584	4.3369	0
GSM547796		1		0	1	0		65			9.2101	11.5448	2.6963	11.7554	9.9173	8.7680	8.5608	5.6284	1

GSM547797		1	0	1	0		70	9.7985	10.6725	2.7293	12.6115	10.7478	9.1025	8.3783	5.1666	0
GSM547798		1	0	1	0		71	8.4579	11.7762	3.3049	12.2742	10.1333	8.3893	8.5393	4.7802	0
GSM547799		1	0	1	1		75	10.3771	11.5861	4.8363	11.8486	9.9793	7.8062	9.1797	4.9558	0
GSM547800		1	0	1	1		46	10.0053	9.5416	6.5145	11.9909	9.4088	8.0804	8.6741	5.3020	0
GSM547801		1	0	1	1		73	9.3758	9.5922	6.5718	11.9417	10.3582	9.3881	9.5114	5.6209	1
GSM547802		1	0	1	0		70	8.9665	9.9981	4.4368	12.1918	10.2785	8.9947	8.4433	4.9387	0
GSM547803		1	0	1	0		53	10.5427	10.7103	2.6303	12.3461	11.0944	9.0713	8.8640	4.9663	1
GSM547804		1	0	1	1		62	9.9083	10.6695	5.5933	12.1127	10.2866	7.7460	9.0516	4.8732	0
GSM547805		1	0	1	1		74	10.4388	12.5577	4.8357	10.9379	9.2483	7.8278	8.9050	4.7506	0
GSM547806		1	0	1	1		72	9.7407	11.8728	5.0225	12.2759	9.9582	9.0173	8.8957	5.3733	0
GSM547807		1	0	1	0		73	9.8066	10.9561	3.1899	11.8935	9.8279	7.4062	8.9669	5.1095	0
GSM547808		1	0	1	1		70	9.8688	10.3190	6.4057	12.4959	11.0949	8.9705	8.6218	5.0155	1
GSM547809		1	0	1	1		65	10.1458	11.1870	7.0749	12.1156	10.2472	8.4980	9.1448	5.3900	0
GSM547810		1	0	1	1		60	9.5234	10.9637	5.2569	11.1040	10.3626	8.2527	8.8883	5.1090	0
GSM549230		0	0	0	0	3	55	8.1152	6.0177	3.2669	11.9904	9.3409	8.3929	8.4927	5.0027	0
GSM549231		0	0	0	0	3	52	8.0389	6.6408	3.7787	11.7623	9.6611	9.4832	8.6809	5.3732	0
GSM549232		1	0	0	0	3	62	9.3011	8.4961	3.6855	12.3352	9.6388	9.2922	8.9004	5.4587	0
GSM549233		0	0	0	0	3	52	9.0706	7.7565	3.9291	11.6688	9.5007	9.1829	8.5892	5.3910	0
GSM549234		0	0	0	0	3	50	9.1465	6.3105	3.8778	12.3605	9.9303	8.3972	8.6458	5.3286	0
GSM549235		0	0	0	0	3	50	9.1819	7.9407	3.9614	12.2821	10.0846	9.4129	8.9710	5.3800	0
GSM549236		1	0	0	0	3	45	8.2411	8.0222	3.9346	11.7988	9.4115	8.4053	8.6038	5.2976	0
GSM549237		0	1	0	0	3	75	11.5880	5.8388	3.3720	12.4092	10.1725	8.9320	8.7716	5.1983	0
GSM549238		1	0	0	0	3	50	9.5614	6.7057	3.6774	12.3747	10.0756	8.8009	8.6793	5.4551	0
GSM549239		1	0	0	1	3	55	8.6397	9.2419	5.3215	12.1833	9.8817	9.1416	8.7023	5.0705	0
GSM549240		0	0	1	0	2	31	8.3739	10.3792	3.9237	12.4645	10.0643	8.7760	8.4353	5.3916	0
GSM549241		1	0	0	0	2	43	9.9144	9.3153	4.5018	12.7154	9.9739	9.4139	8.8302	4.9273	0
GSM549242		0	0	0	0	3	57	9.4238	5.0146	3.3236	12.6973	10.3935	9.7702	8.6667	5.2191	0
GSM549243		0	0	1	0	2	43	8.0700	9.6016	3.9977	12.3221	9.8073	9.1829	8.8957	5.3522	0
GSM549244		1	0	0	0	3	45	8.7895	8.2024	3.7973	12.3263	9.5560	8.7895	9.1493	5.3320	0
GSM549245		0	0	0	0	3	32	9.6025	6.9082	3.9027	12.2685	9.9206	8.8710	8.5669	5.1542	0
GSM549246		0	0	0	0	2	38	8.8398	7.4044	3.7690	12.2992	9.6048	9.5494	8.9463	4.7575	0
GSM549247		1	0	1	0	2	34	8.1224	10.7549	4.0893	12.6592	10.1401	8.6768	8.5021	5.2762	0
GSM549248		0	0	0	0	2	55	9.0203	7.8309	3.8880	12.3913	10.5027	9.8547	8.6453	5.0266	0
GSM549249		1	0	0	0	3	60	9.1131	8.2291	4.1273	12.7880	10.3105	9.0271	9.2429	5.4985	0
GSM549250		0	0	0	0	3	38	8.6744	6.6744	3.6123	12.3478	10.1004	9.9538	9.0125	5.6802	1
GSM549251		1	0	0	0	3	37	7.6033	5.8924	3.6186	12.0340	9.6113	9.5521	8.7121	5.1083	0
GSM549252		0	0	0	0	3	32	9.5462	7.2126	4.2026	12.2117	9.9464	8.1146	8.3435	5.8446	1
GSM549253		0	0	0	0	3	55	8.3668	7.2566	3.9641	11.9264	9.6388	8.6936	8.2271	5.1299	0
GSM549254		0	0	0	0	3	38	9.4200	5.0649	3.3897	12.4816	10.2611	9.8484	8.4519	5.1892	0
GSM549255		0	0	0	0	2	43	9.4401	8.2955	4.0852	12.9698	10.5103	9.1970	8.9676	5.3787	1
GSM549256		0	0	0	0	3	47	8.4486	7.3484	3.7836	11.8092	9.3406	9.2292	8.1358	4.8807	0
GSM549257		0	0	0	0	3	54	8.6891	6.5189	3.4852	12.3537	9.4485	8.5590	9.5788	4.9729	0
GSM549258		1	0	1	0	3	47	8.5938	11.1968	4.4668	12.2860	9.4577	8.9244	8.5327	4.8899	0
GSM549259		1	0	1	1	2	46	10.5512	10.7056	5.4183	12.5354	10.4779	8.8720	9.4275	5.6046	1
GSM549260		1	0	0	0	3	45	8.4077	5.9949	3.5621	12.0706	9.6809	9.1906	7.9785	4.9897	0
GSM549261		1	0	1	0	2	39	9.7931	9.9747	3.2238	12.9559	10.7046	9.5264	8.7058	5.3931	1
GSM549262		0	0	0	0	2	67	8.5666	7.5094	3.9411	11.8346	9.3507	9.3115	8.4984	5.0294	0
GSM549263		0	0	0	0	3	53	8.7451	7.4523	3.4476	12.2297	9.9569	9.0608	8.9084	5.1639	0
GSM549264		1	0	1	0	3	66	8.7948	12.2879	4.4388	11.8147	9.7074	9.0321	8.6107	5.3023	0
GSM549265		0	0	0	0	3	55	9.0335	7.6362	4.1756	12.5620	9.3939	8.7675	8.8922	5.4668	0
GSM549266		1	0	1	0	3	51	9.4670	11.3057	4.2680	12.7173	9.9965	9.3061	9.2626	5.0481	0
GSM549267		0	0	0	0	3	33	8.6327	7.6484	4.0490	11.8293	9.7813	8.8677	8.5104	5.2131	0
GSM549268		0	0	1	0	1	51	9.6168	10.3783	3.8320	12.0363	9.3428	8.2414	8.5234	4.8897	0
GSM549269		1	0	1	1	1	49	10.4307	10.7659	7.8980	13.2612	10.6499	8.8458	9.1114	6.0113	1
GSM549270		1	0	1	0	2	68	10.1670	10.6423	4.4311	13.3755	10.0898	7.7680	9.2533	5.1329	1
GSM549271		1	0	0	0	3	45	8.9208	6.6273	3.9164	11.8834	10.2276	7.5226	9.2394	5.0683	0
GSM549272		1	0	1	0		73	10.0134	11.3674	4.3231	12.1978	9.7436	8.2037	8.8438	5.1791	0
GSM549273		0	0	0	0		46	10.6070	2.8217	3.2371	9.5746	7.8941	6.7991	9.7290	6.2911	1
GSM549274		1	0	0	1	3	78	10.9849	8.7726	4.8472	13.0001	10.0023	8.6382	9.3200	6.1559	1
GSM549275		1	0	1	0		40	9.4634	10.0772	4.7342	13.2664	10.0378	8.7969	9.2225	5.0498	1
GSM549276		1	0	1	0	1	45	10.6049	10.4920	3.7260	11.2776	10.4543	8.4558	9.3805	5.4983	0
GSM549277		1	0	1	0	2	40	9.6285	10.7652	3.9063	11.6812	8.8959	7.8838	8.6645	5.0503	0
GSM549278		0	0	0	0	2	52	9.8741	7.2481	4.2041	12.6631	9.9651	9.4339	8.4526	4.8563	0
GSM549279		1	0	1	0	2	50	10.3606	9.8480	4.1037	12.5024	9.9803	9.4614	9.2129	5.1647	0
GSM549280		1	0	1	0	2	50	9.3776	10.8667	4.0145	12.4843	10.2201	8.6033	8.6978	4.7425	0
GSM549281		1	0	1	0	2	55	9.7888	9.6260	4.0997	12.0084	9.2468	8.6466	8.6669	5.1846	0
GSM549282		0	0	0	0	3	66	7.9280	7.1204	3.7397	12.1375	9.5179	7.2774	8.7776	4.7846	0
GSM549283		0	0	0	0	2	65	9.9858	9.3120	3.7596	11.9725	9.9798	8.8352	8.9868	4.9675	0
GSM549284		1	0	1	0	3	44	9.5421	11.7060	4.4363	12.7638	10.0237	8.9719	9.1094	5.3045	0
GSM549285		1	0	1	0	2	64	8.6957	9.5754	3.2460	11.9059	9.1713	8.4131	8.5204	4.8415	0
GSM549286		0	0	0	0	2	54	10.5402	9.0870	4.3658	12.3712	10.3024	9.2048	8.3790	5.1052	0
GSM549287		0	0	0	0	3	31	8.2866	5.7451	3.6910	12.1958	9.8828	8.2128	8.4543	4.7894	0
GSM549288		1	0	0	0	2	43	9.3033	9.4641	3.1668	12.7349	10.2901	8.2337	8.4288	5.0897	0
GSM549289		0	0	0	0	3	46	8.9819	8.2666	4.4117	12.5880	9.8275	9.2743	8.2327	5.7276	1
GSM549290		0	0	0	0		32	9.3831	6.2118	3.4538	12.3220	10.3019	9.4748	8.3440	5.3785	0
GSM549291		0	0	0	0	3	58	7.9083	7.3835	3.8648	12.4880	10.0841	9.7620	9.0683	4.9176	0
GSM549292		1	0	1	1	2	62	10.1173	12.3988	8.0354	12.7045	10.5866	8.9811	9.2290	5.7069	1
GSM549293		1	1	1	0	3	32	11.1921	11.1925	4.0867	12.2524	10.6723	9.2424	9.2202	5.3863	0
GSM549294		1	0	1	0	2	53	9.9742	9.7551	4.0760	12.6539	11.0388	9.4290	9.0924	5.0847	1
GSM549295		1	0	1	0	2	34	9.3926	10.1955	4.4311	13.7152	11.0557	9.0072	9.0438	5.5737	1
GSM549296		0	0	0	0	3	38	8.8836	6.5480	3.8100	12.3465	11.1651	9.3337	9.0495	5.3862	1
GSM549297		1	0	1	1	2	49	9.3305	11.8812	6.1202	12.3847	9.2043	8.4846	8.6633	5.2176	0
GSM549298		0	0	0	0	2	57	10.2039	5.7199	3.3237	12.3797	9.7061	6.9168	8.9261	5.1507	0
GSM549299		0	0	1	1		42	9.8887	10.1787	6.8309	12.8966	10.2548	8.7122	9.2223	5.0239	1
GSM549300		0	1	1	0		52	11.9726	10.2932	4.2405	12.8956	10.5183	8.4106	9.0356	5.0518	1
GSM549301		0	0	0	0		57	9.4179	9.1835	3.6053	12.1058	8.9024	8.0314	9.1683	4.9717	0
GSM549302		1	0	1	0		36	10.5281	11.0811	4.1842	12.7643	11.0970	9.4189	9.0211	5.3161	1
GSM549303		0	0	0	0		34	8.8281	6.8259	3.7864	11.6089	9.1888	8.1661	8.3037	4.3907	0
GSM549304		1	0	1	0		43	10.0148	10.9099	4.1261	9.8485	10.8874	9.7064	9.0074	5.8642	1
GSM549305		1	0	1	1		62	10.6450	13.2364	5.9927	13.2268	9.3551	8.7549	9.4778	4.7125	1
GSM549306		1	0	0	0		43	9.8601	9.2857	3.7802	12.9807	9.8434	9.4204	9.2602	5.6129	1
GSM549307		1	1	1	0		52	11.2073	10.9958	4.2914	12.4363	9.7733	8.9512	8.9067	4.9146	0
GSM549308		1	0	0	0		46	8.9766	8.4565	3.8117	11.5865	9.4315	8.1845	8.9407	4.5715	0
GSM549309		0	0	0	0		40	9.2939	7.6587	3.6979	11.6187	9.0230	7.9949	8.4816	4.6274	0
GSM549310		0	0	0	0		36	9.5771	5.7947	3.6298	12.4909	11.0271	8.4982	9.1944	5.7029	1
GSM549311		0	0	0	0		35	8.2153	5.9838	3.3614	11.5061	8.9311	8.3260	7.9609	4.6619	0
GSM570498	0	1	0	1	1	3		8.6688	13.3833	6.9056	12.4959	10.9588	9.7963	8.8976	5.3691	1
GSM570499	0	1	1	1	0	3		12.3166	13.6519	3.8610	11.9028	11.1033	8.9247	8.7101	5.7251	1
GSM570500	0	1	0	1	1	3		11.0269	12.4696	8.5542	12.7836	11.1177	10.2266	8.9670	5.5455	1
GSM570501	0	1	0	1	1	3		10.2048	13.8867	5.0849	13.0295	11.5090	9.3503	9.9564	4.9282	1
GSM570502	0	1	0	1	0	3		9.7984	13.9616	3.5109	12.7568	11.0607	9.3620	8.9521	4.9355	1
GSM570503	0	1	0	1	1	3		10.4077	12.3798	6.8981	12.7213	10.4683	9.3324	9.2229	4.6990	0
GSM570504	0	1	0	1	0	3		10.9846	12.0476	3.1083	12.5872	11.5415	9.2893	9.2316	5.5694	1
GSM570505	0	1	0	1	1	3		10.1104	13.5990	6.1206	12.5039	11.0951	9.1802	9.3745	5.9910	1
GSM570506	0	1	1	1	0	3		11.2354	13.8275	3.7012	12.3325	10.6502	8.8801	9.2361	5.1920	0

GSM570507	0	1	0	1	1	3	10.6902	12.6553	5.8920	12.6674	10.8815	8.8325	9.0781	4.8441	0
GSM570508	0	1	0	1	1	3	9.7773	13.4874	5.8988	12.7141	10.9525	9.5983	8.5421	5.2269	1
GSM570509	0	1	0	1	1	3	10.3879	12.4077	6.3231	12.0413	10.6909	8.5975	9.1439	4.9217	0
GSM570510	0	1	0	1	0	3	8.4264	10.5231	3.7524	12.5628	10.4336	9.7746	9.4803	4.5384	0
GSM570511	0	1	0	1	1	3	10.4179	12.6123	5.5438	12.7620	11.1149	8.9625	9.5933	4.6938	1
GSM570512	0	1	0	1	1	3	8.2511	12.6771	7.7338	12.2862	10.7472	8.9863	8.8447	4.6474	0
GSM570513	0	1	0	1	0	3	10.4306	13.3813	3.4500	12.7658	11.4089	9.6050	9.0090	5.4639	1
GSM570514	0	1	0	1	0	3	8.9806	13.1416	3.1415	13.0186	11.7817	8.8998	9.4414	4.5873	1
GSM570515	0	1	0	1	0	3	10.6565	13.1728	3.5611	12.6202	10.9332	9.9702	9.0792	5.7250	1
GSM570516	0	1	0	1	0	3	10.1254	13.3262	3.3296	11.8356	10.8761	8.9084	8.1687	5.5122	0
GSM570517	0	1	0	1	1	3	10.3175	13.2691	7.0018	12.5093	10.6500	9.1897	9.6544	4.9980	0
GSM570518	0	1	0	1	1	3	9.6379	12.0632	9.0912	12.6895	10.3444	9.2139	9.3616	5.9850	1
GSM570519	0	1	0	1	1	3	9.7440	12.3442	5.9787	12.3707	10.5563	8.6015	8.9504	5.6072	1
GSM570520	0	1	0	1	1	3	9.7773	11.2290	4.8445	12.3597	10.5648	8.8756	8.7560	5.7108	1
GSM570521	0	1	0	1	0	3	9.5058	12.4657	4.3479	12.8559	10.8279	9.7615	9.0925	6.6738	1
GSM570522	0	1	0	1	0	3	9.5240	12.3993	2.3190	12.8754	10.2333	9.1974	9.0590	5.1337	1
GSM570523	0	1	0	1	1	3	9.6560	10.8554	9.5069	12.8302	10.4650	8.9060	8.8644	5.2648	1
GSM570524	0	1	0	1	0	3	8.7840	10.3791	2.7438	12.0471	10.1736	9.1082	8.4323	4.6063	0
GSM570525	0	1	0	1	1	3	9.3685	10.2229	5.9302	12.9370	11.2574	10.5704	9.6050	5.8260	1
GSM570526	0	1	0	1	1	3	9.7337	12.9714	7.0168	12.5875	10.8403	8.8374	8.9775	5.2556	0
GSM570527	0	1	0	1	0	3	10.6242	11.7175	3.7920	12.9445	11.0551	9.4175	8.8495	6.2562	1
GSM570528	0	1	0	1	0	3	9.8754	11.1071	1.8407	12.3076	10.7661	9.4996	9.1469	5.6446	1
GSM570529	0	1	0	1	1	3	9.8022	11.8236	7.3521	12.3296	9.7098	8.7630	8.9363	5.0903	0
GSM570530	0	1	1	1	0	3	12.0900	11.7087	4.3569	11.5734	10.0200	9.2880	8.9205	5.6179	1
GSM570531	0	1	0	1	1	3	10.3536	11.7984	5.0748	12.9821	11.0181	9.1381	9.7310	5.5764	1
GSM570532	0	1	0	1	0	3	10.5632	10.9222	2.3180	13.1186	11.0946	9.3824	9.2484	6.7032	1
GSM570533	0	1	0	1	1	3	8.9361	11.7450	5.1974	12.0748	9.6021	9.1073	8.8959	4.4984	0
GSM570534	0	1	0	1	1	3	10.0167	11.8664	6.9152	12.1090	10.6718	8.3201	8.8291	4.6756	0
GSM570535	0	1	0	1	0	3	9.7938	10.3269	2.9098	12.4174	10.2467	9.2853	8.3590	5.0474	0
GSM570536	0	1	0	1	1	3	9.8981	11.0453	6.7497	13.2274	11.0829	9.6117	9.6315	5.8531	1
GSM570537	0	1	0	1	1	3	9.1938	13.3731	5.6956	12.3135	10.0069	8.9502	8.9225	5.3391	0
GSM570538	0	1	0	1	0	3	10.2931	12.3658	4.7337	12.6952	11.0103	9.5497	9.3567	6.1185	1
GSM570539	0	1	0	0	0	3	8.7780	6.0178	2.9000	12.7660	10.4182	9.5960	9.2810	5.3208	0
GSM570540	0	1	0	1	1	3	8.9981	11.5440	8.0452	12.7237	10.0202	9.1938	8.5920	5.3264	0
GSM570541	0	1	1	1	1	3	11.6644	10.9246	6.4237	13.0361	10.6821	9.3832	8.6260	5.1277	1
GSM570542	0	1	0	1	0	3	8.7618	12.0365	3.0013	12.6159	10.5726	9.1936	8.3773	5.4178	0
GSM570543	0	1	0	1	0	3	9.3012	11.3831	2.1588	12.9171	10.8802	10.1344	8.0708	5.6950	1
GSM570544	0	1	0	1	1	3	7.8520	12.6099	4.9123	12.5266	10.4151	8.9498	9.3694	6.0273	1
GSM570545	0	1	0	1	1	3	10.0929	11.9412	5.6362	12.9409	11.3083	8.9999	8.5960	5.7705	1
GSM570546	0	1	0	1	0	3	9.5786	10.9718	2.0916	13.1029	10.6965	10.0903	9.6256	6.1946	1
GSM570547	0	1	0	1	1	3	10.2475	12.0498	9.9290	12.6877	10.2084	8.8807	9.0205	5.3240	0
GSM570548	0	1	0	1	1	3	9.9370	12.0632	6.2030	12.4945	10.4259	8.8127	8.8505	5.4305	0
GSM570549	0	1	0	1	0	3	11.1347	12.1051	2.3330	13.0644	10.9990	8.6718	8.9561	5.6365	1
GSM570550	0	1	0	1	0	3	10.6052	11.5607	3.3184	12.3967	9.9844	8.9292	8.6962	4.5121	0
GSM570551	0	1	0	1	1	3	9.9174	11.1465	7.8764	12.7645	10.4109	9.1085	9.1275	5.1813	0
GSM570552	0	1	0	0	1	3	9.7904	9.4643	4.9759	12.4442	10.1790	9.3227	8.3729	4.5943	0
GSM570553	0	1	0	1	1	3	9.5075	11.7517	6.6543	12.2655	10.3375	8.9905	8.7650	5.4845	0
GSM570554	0	1	0	1	0	3	8.1536	12.3327	2.4973	12.8081	10.7181	9.5675	9.0804	5.9488	1
GSM570555	0	1	0	1	0	3	10.0516	12.0206	3.6039	12.6272	10.4462	8.8354	8.7772	5.6698	1
GSM570556	0	1	0	1	0	3	10.2747	12.6376	3.1025	12.4392	10.5222	9.1616	9.3820	5.5358	0
GSM570557	0	1	0	1	0	3	8.9785	10.3909	3.1236	12.4972	10.1791	8.9748	8.1186	4.8148	0
GSM570558	0	1	0	1	0	3	8.9368	10.4749	3.2501	12.1628	10.1293	8.4435	8.9493	5.3356	0
GSM570559	0	1	0	1	1	3	9.3800	11.5473	5.2460	12.8976	10.5092	9.0503	8.9417	5.2331	1
GSM570560	0	1	0	1	0	3	9.2729	11.2334	4.5514	12.8904	10.7454	8.8340	9.3635	4.4204	1
GSM570561	0	1	0	1	1	3	10.3026	12.2112	9.4429	12.8723	10.7047	8.6532	9.6682	5.3265	1
GSM570562	0	1	1	1	1	3	11.2537	11.1521	8.8842	12.4600	10.4464	9.9322	8.7408	5.7923	1
GSM570563	0	1	0	1	1	3	9.4489	11.8951	6.8739	12.9740	10.5555	8.6413	8.6447	6.0016	1
GSM570564	0	1	0	1	1	3	11.1500	10.0284	5.0128	12.8541	11.0983	9.5537	8.8625	5.5880	1
GSM570565	0	1	0	1	1	3	9.9049	10.4181	7.2447	12.4975	10.6529	8.9315	8.2186	5.0625	0
GSM570566	0	1	0	1	1	3	9.8586	12.0586	8.0405	12.7374	10.7492	9.1191	9.1843	4.7645	0
GSM570567	0	1	0	1	1	3	9.1129	11.0508	7.1895	12.1031	10.5679	9.4714	8.8602	5.8748	1
GSM570568	0	1	0	1	0	3	10.9157	11.6261	2.1166	12.7089	10.1771	9.6455	9.2749	4.8596	0
GSM570569	0	1	0	1	1	3	9.9641	11.3513	6.5037	12.3001	10.0126	8.7849	9.0239	4.8445	0
GSM570570	0	1	0	1	1	3	9.0391	11.5471	6.6708	12.3613	10.4590	9.7814	8.3663	5.5595	0
GSM570571	0	1	0	1	1	3	8.7193	11.6718	5.3089	12.4312	11.5529	10.1762	8.8441	5.2602	1
GSM570572	0	1	0	1	1	3	9.0546	10.0248	6.0277	12.9895	10.6258	9.4757	9.5369	5.3528	1
GSM570573	0	1	0	1	1	3	10.4707	11.5465	6.9096	12.4364	10.0795	8.9708	9.1758	5.3391	0
GSM570574	0	1	0	1	1	3	9.3651	11.7711	5.6734	12.1367	10.0149	8.5750	9.1976	5.1265	0
GSM570575	0	1	0	1	1	3	9.6891	11.8906	7.8177	12.4226	10.4882	8.8932	9.3423	5.4833	0
GSM570576	0	1	0	1	1	3	8.9081	11.3258	10.1115	12.6680	10.3455	9.1598	9.2491	5.4227	0
GSM570577	0	1	0	1	1	3	10.5478	11.8205	5.8080	12.2543	10.5081	8.9471	9.1919	5.6224	1
GSM570578	0	1	0	1	1	3	8.5499	10.2748	8.0678	12.6479	10.6872	9.1733	9.1821	5.6611	1
GSM570579	0	1	0	1	1	3	9.6920	11.2641	5.9539	12.7116	10.4208	9.2578	8.4906	5.2840	0
GSM570580	0	1	0	1	0	3	9.0988	11.9247	3.2346	12.4833	10.4088	9.2177	9.2912	6.2677	1
GSM570581	0	1	0	1	1	3	10.1168	11.8748	7.4696	12.6607	10.6061	8.6863	9.2964	5.9001	1
GSM570582	0	1	0	1	1	3	8.5433	11.1101	8.0200	12.4694	10.2148	9.5079	9.1848	5.6831	1
GSM570583	0	1	0	1	1	3	9.3012	11.2575	9.5209	12.8842	10.6935	9.6022	8.9071	6.0197	1
GSM570584	0	1	0	1	1	3	10.5163	11.9409	7.6664	12.5044	11.0584	9.3982	8.9197	4.7722	1
GSM570585	0	1	0	1	1	3	9.7022	11.8401	7.3627	12.7538	10.8127	9.4747	9.9144	5.7877	1
GSM570586	0	1	0	1	0	3	9.0897	11.3587	3.9532	12.5397	10.2367	9.4658	8.5774	5.2404	0
GSM570587	0	1	0	1	0	3	9.8947	10.9813	2.9608	12.6691	9.7086	9.6542	8.8476	5.0768	0
GSM570588	0	1	0	1	1	3	9.2582	11.4296	6.2477	12.7171	10.5126	8.9178	9.0242	6.4866	1
GSM570589	0	1	0	1	0	3	10.4743	11.3673	4.6901	13.0109	11.0209	9.0445	8.9372	5.8741	1
GSM570590	0	1	0	1	0	3	8.6897	11.4776	2.4089	12.6185	10.3456	9.6159	7.9101	6.2421	1
GSM570591	0	1	0	1	1	3	10.4806	10.9774	6.5323	12.8596	10.6597	8.9028	9.2630	5.9812	1
GSM570592	0	1	0	1	1	3	9.7515	11.7089	7.5158	12.7057	11.0233	9.4262	8.8026	6.5094	1
GSM570593	0	1	0	1	1	3	10.1287	11.7898	6.7393	12.2482	10.2895	9.3896	8.8700	4.3694	0
GSM570594	0	1	0	1	1	3	10.1336	11.8494	9.1849	12.3896	10.0036	9.6270	8.6934	4.7800	0
GSM570595	0	1	0	1	1	3	10.3188	11.8334	7.9102	12.9732	11.2798	9.1158	9.3018	5.7148	1
GSM570596	0	1	0	1	1	3	9.2089	11.5220	5.9788	12.7603	10.7265	9.5461	9.4077	6.9380	1
GSM570597	0	1	0	1	1	3	10.5863	11.6087	4.9632	12.1467	10.3072	10.4286	8.5324	5.5771	1
GSM570598	0	1	0	1	0	3	8.9796	11.9823	3.2113	12.6924	10.0836	8.8757	8.7048	4.9647	0
GSM570599	0	1	0	1	1	3	9.7972	12.4485	6.8122	12.5632	10.2724	8.8849	8.2014	5.3081	0
GSM570600	0	1	0	1	1	3	9.2402	12.4893	6.7116	12.5299	10.5460	8.3936	8.7228	5.1434	0
GSM570601	0	1	0	1	1	3	10.1875	12.6890	8.5914	12.5705	10.1213	8.6793	9.3286	5.3904	0
GSM570602	0	1	0	1	1	3	9.1324	12.0965	6.9821	12.8868	10.3088	9.2917	9.2383	5.4672	1
GSM570603	0	1	0	1	1	3	10.4398	11.2974	10.0512	12.6468	10.3445	8.9284	9.4946	5.9809	1
GSM570604	0	1	0	1	1	3	9.6514	10.8839	6.7000	12.8615	10.4998	9.6599	9.6579	5.7926	1
GSM570605	0	1	0	1	1	3	10.6317	12.0699	8.3312	12.6569	10.5366	8.8104	9.1407	5.5402	0
GSM570606	0	1	0	1	0	3	10.1491	11.0145	4.8150	12.9248	10.9303	9.4968	9.3528	6.1508	1
GSM570607	0	1	0	1	0	3	9.2992	11.4464	2.2932	12.3957	10.4315	9.8169	8.5306	4.9928	0
GSM570608	0	1	0	1	1	3	9.8213	12.2118	4.9789	12.3718	10.3763	9.4643	9.1433	5.6960	1
GSM570609	0	1	0	1	0	3	8.9475	10.6914	2.4863	12.8698	10.5181	8.7469	8.7607	6.0182	1
GSM570610	0	1	0	1	1	3	9.6703	12.2055	8.8057	12.7330	10.3622	9.1442	8.8065	5.4685	0
GSM570611	0	1	0	1	1	3	9.9545	11.4524	8.1157	13.3652	11.1493	9.9103	10.1037	5.5009	1

GSM570612	0	1		0	1	0	3		7.9178	12.8581	4.1875	12.1689	10.5955	8.9587	9.1107	6.0606	1
GSM570613	0	1		0	1	1	3		10.7388	11.4081	6.4760	12.7174	9.4854	8.7102	8.4508	5.9159	1
GSM585300		1	1	0	1	1		43	9.3728	13.2910	5.1756	12.9123	10.9492	10.4005	9.3690	5.7076	1
GSM585301		1	1	0	1	0		55	8.8239	11.3240	4.5580	12.5039	10.8348	9.9022	10.0950	5.4416	0
GSM585302		1	0	0	1	0		47	8.3140	10.8782	4.0056	12.0947	10.2731	9.9387	8.8131	5.8828	1
GSM585303		1	1	0	1	1		53	9.7601	9.6551	4.9166	12.1871	10.3120	8.4477	9.6101	6.0908	1
GSM585304		1	1	0	1	0		56	8.8631	12.7987	3.7632	12.6439	10.1957	9.4874	8.1175	5.5674	0
GSM585305		1	1	0	1	1		39	9.4525	10.2481	5.0720	12.5304	10.1484	9.4670	8.9400	5.3121	0
GSM585306		1	1	0	1	0		51	10.1758	12.1740	4.7005	12.7099	10.8408	9.1128	9.4237	6.2928	1
GSM585307		1	1	0	1	1		66	8.6561	12.0059	5.6229	12.7093	9.9089	9.0151	8.9009	5.1305	0
GSM585308		1	1	0	1	0		65	9.9696	11.3889	4.0065	12.2797	10.2488	9.0802	9.3387	6.3895	1
GSM585309		1	0	0	1	0		64	9.7921	10.7028	3.6924	12.4528	10.8762	8.1925	9.9604	5.8125	1
GSM585310		1	1	0	1	0		32	9.9869	10.6561	4.4189	11.5295	9.3649	9.6913	8.6287	6.1534	1
GSM585311		1	1	0	1	1		61	9.3023	11.5788	6.7765	12.3206	10.5109	9.1784	8.9035	6.7958	1
GSM585312		1	1	0	1	1		45	9.6916	10.8738	7.0250	12.1551	10.4025	9.3448	8.6370	5.3765	0
GSM585313		1	1	0	1	0		60	10.1118	10.8977	4.3921	11.4385	9.4405	8.4156	8.7110	5.3748	0
GSM585314		1	1	0	1	1		64	9.7775	10.8708	4.8443	11.8489	10.0385	8.5449	9.0808	5.6844	1
GSM585315		1	1	0	0	0		55	8.8387	9.1724	4.8140	11.4071	9.3854	8.8094	8.4257	5.4888	0
GSM585316		1	1	0	0	0		84	9.3204	8.9285	3.7090	11.9173	9.7183	8.9177	8.6357	5.5858	1
GSM585317		1	0	0	1	0		54	9.3587	8.8974	3.7758	12.2170	10.0424	9.3241	8.4841	4.7887	0
GSM585318		1	1	0	1	1		60	9.3501	10.5670	6.4751	11.5483	9.6191	9.8093	8.6769	5.6049	1
GSM585319		1	1	0	1	1		50	8.4528	10.7415	5.6101	11.7519	9.1276	8.8744	8.3641	5.1555	0
GSM585320		1	1	0	1	0		45	9.9538	9.8160	4.6505	12.3284	10.0531	9.5888	8.5900	5.4323	0
GSM585321		0	1	0	1	1		53	9.5906	11.1326	6.8437	12.0351	9.7070	9.7434	9.9604	5.2195	0
GSM585322		1	1	0	1	1		51	10.7840	11.5584	5.9740	12.2855	10.2408	9.7373	8.9059	5.7048	1
GSM585323		1	1	0	1	0		54	9.9337	10.4427	3.6231	11.7687	9.4090	8.6243	8.9759	5.5357	0
GSM585324		1	1	1	1	0		61	12.1901	9.8350	3.8082	13.0635	10.7520	8.9450	8.6610	6.2983	1
GSM585325		1	1	0	1	1		78	9.7095	10.7504	6.8994	12.1258	10.3400	8.9703	8.9079	5.6158	1
GSM585326		1	1	0	1	0		66	9.5195	11.3104	4.2927	11.3350	9.9538	9.2511	8.4850	5.5047	0
GSM585327		1	1	0	0	0		33	8.7186	9.2729	2.3010	12.2339	10.0595	8.9836	8.6118	5.0624	0
GSM585328		1	1	0	0	1		32	10.3508	8.7134	4.8871	12.6104	10.8775	8.5698	8.9355	5.7559	1
GSM585329		1	1	0	0	0		49	9.1652	9.1958	3.8064	12.1502	10.3081	7.8595	9.1133	6.5297	1
GSM585330		1	1	0	0	1		34	9.8404	7.9045	4.8507	11.8112	10.2863	8.4385	8.8173	6.2817	1
GSM585331		1	1	0	1	1		75	9.1595	10.9760	4.9684	12.1308	10.7556	8.6714	9.5070	6.0703	1
GSM585332		1	1	0	1	0		49	9.9515	11.5061	4.5832	12.5362	10.3206	10.1651	8.9990	5.6466	1
GSM585333		1	1	0	1	1		55	9.9208	10.6019	5.7601	12.2580	10.1903	8.9784	9.2348	6.1216	1
GSM585334		1	1	0	0	1		54	9.7982	9.1907	7.1531	11.9833	10.1319	9.1345	8.6089	5.5467	0
GSM585335		1	1	0	1	1		30	10.2084	9.7924	6.2752	12.2487	9.9161	8.9352	8.7564	5.9679	1
GSM585336		1	1	0	0	1		31	10.3930	9.2595	5.0893	12.3830	10.2504	8.7500	8.6549	5.3828	0
GSM585337		1	1	0	0	0		33	10.5558	9.1673	3.7049	12.6839	10.9426	9.6924	8.5281	6.6084	1
GSM585338		1	1	0	1	1		53	10.3396	10.3300	4.8760	12.7864	10.3519	8.9212	8.8063	5.3901	0
GSM585339		1	1	0	1	0		61	9.9705	12.4878	3.2996	12.9767	10.6043	8.7150	9.0915	5.3453	1
GSM585340		1	1	0	1	0		47	9.4939	12.3921	4.1360	12.2912	11.4490	8.8233	9.1354	6.2778	1
GSM585341		1	0	0	1	0		81	9.5108	9.9042	2.8900	11.4296	9.5193	9.1993	8.5913	5.2360	0
GSM585342		1	1	0	1	0		67	10.3276	12.0507	4.6047	12.5178	10.3819	8.4476	8.6210	5.5558	0
GSM585343		0	0	0	1	1		41	9.7216	10.3454	5.1432	11.5397	10.5936	9.1480	8.8028	5.2667	0
GSM585344		1	0	0	0	0		36	9.9216	8.7442	2.9332	12.9192	10.9280	9.3462	8.6546	5.4593	1
GSM585345		1	0	0	1	0		65	9.2447	12.7377	3.5222	13.0774	11.0133	8.8895	9.1168	5.2942	1
GSM585346		1	1	0	1	1		73	9.1392	10.6342	5.0726	12.2620	10.1072	8.5173	8.7715	4.8372	0
GSM585347		1	1	0	1	0		54	10.0693	10.3623	3.5162	11.7017	10.9110	9.0532	9.1179	5.4743	0
GSM585348		1	1	0	1	0		39	10.0500	10.4957	3.1463	12.4250	10.4515	9.2865	9.3739	5.5459	0
GSM585349		1	1	0	1	0		35	10.0886	10.9885	4.6923	11.9611	10.8726	9.3271	9.0399	6.5437	1
GSM585350		1	0	0	0	0		48	10.7888	9.3365	2.7236	12.3348	10.0563	8.0729	8.4834	5.6107	1
GSM585351		1	1	0	1	1		66	9.1661	9.5906	6.5625	11.5661	9.5528	8.2172	9.2489	4.7524	0
GSM585352		1	1	0	1	1		63	9.4212	10.4156	7.8241	11.3145	9.4234	8.6535	9.0093	5.4003	0
GSM585353		1	1	0	1	0		61	9.9520	11.3987	4.6612	12.0085	9.8108	8.6715	8.4743	5.1734	0
GSM585354		1	1	0	1	0		60	8.8470	10.0407	4.0305	11.8351	9.1924	9.2861	8.5881	4.8457	0
GSM585355		1	1	0	1	0		53	10.5434	9.6381	2.8508	12.6665	10.8410	8.9410	9.1902	5.8576	1
GSM585356		1	1	0	1	1		55	8.7673	11.0924	6.6560	12.5186	10.1010	8.5431	9.3178	5.5680	0
GSM585357		1	1	0	1	1		66	9.0641	10.3676	5.9469	11.1908	9.6744	8.5695	9.0196	5.3588	0
GSM585358		1	1	0	1	0		57	8.5816	11.9861	4.3241	12.4244	10.5858	10.5307	8.7975	4.3332	0
GSM585359		1	1	0	1	0		78	10.9907	9.8495	4.6349	9.7762	9.2711	8.1084	10.0113	5.1174	0
GSM585360		1	0	0	0	0		31	9.8539	7.7536	3.0320	10.0653	9.3828	8.7469	8.4748	4.6025	0
GSM585361		1	1	0	1	1		42	9.8090	11.5883	5.1789	11.3718	9.8389	8.8653	8.8664	4.6044	0
GSM585362		1	1	0	1	1		58	10.4040	12.9776	5.2941	11.8432	10.1378	8.2971	8.7779	5.8968	1
GSM585363		1	1	0	1	1		34	11.0519	10.6493	6.5199	11.5985	9.7732	9.3435	8.2979	5.6625	1
GSM585364		1	1	0	1	0		66	8.3922	10.2700	4.1483	12.2207	9.9816	8.9728	9.2744	4.5344	0
GSM585365		1	1	0	1	0		60	9.2894	12.2140	3.5983	12.5758	10.4312	8.6967	9.1601	5.1444	0
GSM585366		1	1	0	1	0		80	9.1785	12.0143	3.9746	11.7770	10.2300	8.9779	8.4609	4.9867	0
GSM585367		1	1	0	1	0		43	9.0655	9.9104	3.8451	12.2647	11.0703	8.9736	8.7772	5.2048	1
GSM585368		1	1	0	1	0		64	9.3680	11.8303	4.1612	11.6579	10.6433	8.5352	9.4684	5.0849	0
GSM585369		1	1	0	1	1		82	9.4224	12.3310	6.1758	12.0994	10.0821	8.5231	9.1944	5.5542	0
GSM585370		1	1	0	1	1		52	8.7112	11.2643	5.4014	12.7486	10.4884	9.2277	8.8008	5.8223	1
GSM585371		1	1	0	1	1		52	9.7390	13.2530	5.2198	12.0404	10.7082	8.6103	9.2447	5.6323	1
GSM585372		1	1	0	1	0		43	8.5829	10.2939	3.6331	11.9840	10.3598	8.5253	8.8611	4.7521	0
GSM585373		1	1	0	1	1		32	9.0838	10.7990	5.6688	12.1895	10.3070	9.5135	8.2489	5.7118	1
GSM585374		1	1	0	1	0		48	9.4687	10.2609	4.2855	11.5974	9.9955	8.9043	9.2024	5.1362	0
GSM585375		1	1	0	1	1		44	8.0296	11.2023	5.0743	12.8810	10.9518	8.9449	8.8697	5.9975	1
GSM585376		1	1	0	1	0		24	8.9908	11.4099	3.5348	12.1007	11.2053	9.7276	9.2034	6.3798	1
GSM585377		1	1	0	1	1		54	11.1121	12.9310	6.5657	12.0073	9.8580	8.5977	9.2133	5.9696	1
GSM585378		1	1	1	1	1		80	12.7755	10.0305	5.5817	12.4554	10.2497	9.7350	8.8955	5.5308	0
GSM585379		1	1	0	1	1		40	9.2678	10.2205	8.5897	12.4347	9.9951	9.0634	9.1111	5.9879	1
GSM585380		1	1	0	1	0		69	10.5638	13.0734	4.6087	11.8644	9.9568	8.7067	9.0877	6.6668	1
GSM585381		1	0	0	1	0		68	9.7758	12.8733	3.5599	12.1587	10.5694	8.5733	9.1855	5.5047	0
GSM585382		1	1	0	1	0		35	9.9336	11.6733	3.9209	12.2776	10.8814	7.9122	9.3067	7.3374	1
GSM585383		1	1	0	1	0		62	9.0226	12.9973	4.4383	12.3416	10.2307	8.9667	8.8893	5.1837	0
GSM585384		1	1	0	1	1		76	10.0895	13.0193	5.5813	12.2669	10.4615	8.7844	8.2476	5.1392	0
GSM585385		1	1	0	1	1		71	9.5623	12.3552	5.9747	12.6172	10.7805	9.2072	9.4162	5.3910	0
GSM585386		1	1	0	1	1		78	8.7026	12.0423	6.6977	12.4568	10.1337	9.0631	8.7894	5.1347	0
GSM585387		1	1	0	1	1		79	9.9220	10.7889	6.8265	11.8756	10.1050	9.7361	9.6582	5.8771	1
GSM585388		1	1	0	1	1		60	9.9431	13.2085	6.3234	12.3728	10.4396	9.8522	9.3912	7.6911	1
GSM585389		1	1	0	1	1		73	9.4480	12.5521	10.3464	12.3964	10.9213	9.3385	9.5397	6.3602	1
GSM585390		1	1	0	1	0		60	10.5173	12.3007	3.1173	11.8609	10.0874	8.5311	8.2774	4.6347	0
GSM585391		1	1	0	1	0		60	10.8313	10.7739	3.4378	11.9199	9.9296	8.4812	9.0702	6.2798	1
GSM585392		1	0	0	1	1		82	9.7922	10.7396	6.6736	12.2227	10.1809	9.1077	9.2932	4.4680	0
GSM585393		1	1	0	1	1		78	10.2156	13.2831	5.0412	12.7536	10.5449	8.6364	8.5291	5.1054	0
GSM585394		0	0	0	1	0		49	8.3141	13.0211	3.5415	12.1094	9.2132	9.3434	8.4056	5.1236	0
GSM585395		1	1	0	1	0		66	9.2371	13.5689	4.5242	12.5100	9.5912	8.8129	8.7254	5.0993	0
GSM585396		1	0	0	1	0		56	9.3910	11.8609	3.3432	12.4996	9.9670	9.4967	9.2461	6.2472	1
GSM585397		1	1	1	1	0		55	12.1227	10.4229	3.8308	12.0718	10.4379	8.5677	9.1432	5.9251	1
GSM585398		1	1	0	1	1		54	9.8777	13.4067	5.9437	12.1768	9.6038	8.7392	9.2525	4.6439	0
GSM585399		1	1	1	1	0		40	12.8095	10.5378	3.2726	12.5883	10.3282	8.0970	8.8041	4.5633	0
GSM585400		0	1	0	0	0		73	10.5524	9.5133	3.8123	12.2244	10.7513	9.8657	9.3780	4.5644	0
GSM585401		1	1	0	1	1		39	9.0200	11.3414	7.2935	12.1095	10.3082	8.7297	9.0860	6.1887	1
GSM585402		1	1	1	1	0		52	13.0412	9.6488	4.0199	12.3691	9.8915	8.7702	8.4998	4.4467	0

GSM585403	1	1	1	0	1				43					12.2095	9.0068	5.0955	12.2098	10.5639	10.1515	8.2384	4.3682	0
GSM585404	1	1	0	1	0				50					8.5997	10.9187	3.6723	12.8412	11.2904	9.2404	8.9068	4.7704	1
GSM585405	1	1	1	0	0				35					12.7485	7.6656	3.9155	12.4222	10.3563	8.8210	8.7632	5.5283	0
GSM585406	1	1	0	0	0				78					8.4820	8.3183	3.3174	12.2417	10.0642	8.4322	7.8222	5.1650	0
GSM585407	1	1	0	1	0				52					9.3870	10.2069	3.7399	12.3558	10.0627	8.9057	8.9097	4.8858	0
GSM585408	1	0	1	1	0				58					11.5604	12.4352	3.6735	12.4330	10.2155	9.0661	8.7725	5.0766	0
GSM585409	1	1	0	1	0				29					9.1025	10.8196	3.6407	11.8732	10.0824	8.3767	8.9438	6.1686	1
GSM585410	1	1	0	1	1				43					9.2879	12.1983	7.6356	11.9538	10.2621	9.1793	9.6129	6.3928	1
GSM585411	1	1	1	1	0				62					12.3949	10.0572	3.5574	12.1868	9.5110	8.2611	8.3280	5.5693	0
GSM585412	1	0	0	0	0				61					10.6927	7.1039	3.5516	11.7704	9.9099	8.9760	8.5616	4.7489	0
GSM585413	0	0	1	0	0				59					12.3000	4.3594	2.5861	11.7022	10.0886	9.6205	8.5924	5.6438	1
GSM585414	0	0	1	0	0				72					13.1401	6.7381	3.8085	12.5671	9.9655	9.0940	8.8953	5.2125	0
GSM585415	0	0	0	0	0				64					11.1150	5.6195	3.6194	12.3541	10.1542	9.2421	8.9482	5.3439	0
GSM585416	0	0	0	0	0				49					8.6289	5.8767	3.6951	12.0053	10.1661	7.8647	9.1463	7.3660	1
GSM585417	0	0	0	0	1				68					7.2902	8.2371	4.8298	12.3446	8.9508	9.2385	7.9676	5.0625	0
GSM585418	1	1	1	0	0				60					13.7455	9.3625	4.6258	12.0839	9.8370	9.1610	8.8097	6.3374	1
GSM585419	0	0	0	0	0				48					9.2782	5.6771	3.5528	11.7669	10.1786	9.2970	8.9079	5.1265	0
GSM585420	0	0	0	0	0				59					8.6152	6.0062	4.3110	12.2078	10.0464	9.5900	9.2071	5.2571	0
GSM585421	0	0	0	0	0				42					8.8848	5.9800	4.1539	11.6217	9.4178	9.2768	8.8764	5.6563	1
GSM585422	0	0	0	0	0				57					8.6135	8.2287	4.1554	12.2243	9.3801	9.5961	9.0408	5.7205	1
GSM585423	0	0	1	0	0				39					12.7326	5.9579	3.8705	12.2968	9.6868	8.8034	8.5406	5.6733	1
GSM585424	1	0	0	0	0				63					9.5370	6.1516	3.7535	11.9896	10.2748	9.1386	8.5971	4.8391	0
GSM585425	0	0	0	0	0				69					9.3993	3.2497	2.9292	12.3995	10.5042	8.5240	9.0835	6.5427	1
GSM585426	0	0	0	0	0				63					9.0955	5.5844	4.0567	12.6030	10.7068	9.1143	8.4315	5.6400	1
GSM585427	0	0	0	0	0				41					8.6232	5.1484	3.8784	12.1056	9.7015	8.6722	8.9019	4.4512	0
GSM585428	1	0	0	0	0				26					7.9594	7.8037	3.6082	12.1632	10.4312	9.1898	8.7241	5.4721	0
GSM585429	0	0	0	0	0				32					8.3593	5.3146	3.4071	12.3425	10.0324	10.0620	9.1441	4.5340	0
GSM585430	0	0	0	0	0				32					8.4611	4.6331	3.6522	12.5107	10.4620	10.0947	9.4935	4.7343	0
GSM585431	0	0	0	0	0				58					8.4136	6.8487	3.6398	12.4132	10.3947	10.2934	8.9284	5.0750	0
GSM585432	0	0	0	0	0				59					9.0919	5.4660	3.7872	12.4403	10.5243	10.5509	8.9076	5.7530	1
GSM585433	0	0	0	0	0				27					9.5266	7.6006	4.0402	12.6752	10.5242	8.8348	9.2498	5.7255	1
GSM585434	0	0	0	0	0				52					8.3783	9.1162	4.2335	12.4674	9.8009	8.9118	8.2409	5.8053	1
GSM585435	0	0	0	0	0				45					7.7874	7.4298	4.0541	12.3753	9.4968	8.3976	9.5901	5.9240	1
GSM585436	0	0	0	0	0				44					9.3641	6.2288	3.6037	12.5728	10.4800	8.8618	9.3979	6.2836	1
GSM585437	0	0	0	0	0				70					9.7950	7.1980	4.2680	11.7152	10.1695	8.0574	8.8444	5.8417	1
GSM585438	0	0	0	0	0				40					9.6024	7.1985	4.3938	12.2657	9.5634	8.5427	8.3754	5.1874	0
GSM585439	0	0	0	0	0				43					8.7882	9.4740	3.8089	12.0910	9.7295	9.3276	8.3587	5.9494	1
GSM585440	0	0	0	0	0				40					9.4070	4.5301	3.1843	12.4320	10.1249	8.2507	8.9921	6.2077	1
GSM585441	0	1	0	0	0				60					8.2363	7.0101	4.8048	12.2710	9.5283	9.5700	9.5358	6.1142	1
GSM585442	0	0	0	0	0				61					8.7956	8.5434	3.8095	11.7270	9.9734	9.4739	8.9428	5.9215	1
GSM585443	0	0	0	0	0				51					8.5666	8.2645	3.9998	11.9190	10.2039	9.5832	8.5812	5.4934	0
GSM585444	0	0	0	0	0				61					9.0343	7.0721	3.8690	12.3494	10.2779	8.6934	8.0869	5.2199	0
GSM585445	0	0	0	0	0				40					8.3208	6.4433	4.1799	11.5508	9.5088	8.5729	8.9953	4.9638	0
GSM585446	0	0	0	0	0				37					8.1373	6.9840	4.3194	11.9432	9.7424	9.1350	8.0806	5.4849	0
GSM585447	0	0	0	0	0				72					8.3126	7.2826	3.6984	11.9116	11.1533	10.1955	8.5817	5.7956	1
GSM585448	0	0	1	0	0				40					12.2360	7.6181	3.9739	12.5380	10.1800	9.7200	8.8575	4.5725	0
GSM585449	0	0	1	0	0				36					11.9187	5.9456	3.1327	12.3331	10.8070	9.2791	8.3416	4.9749	0
GSM585450	0	0	1	0	0				47					11.9623	7.8830	4.4456	12.5752	9.9441	10.0032	9.6089	4.8035	0
GSM585451	0	0	1	0	0				57					12.6988	6.0716	3.6651	12.3968	9.5360	9.5071	9.3269	4.8476	0
GSM585452	0	0	1	0	0				57					12.5910	3.8984	2.8137	12.5505	9.5614	8.3713	8.3252	5.4026	0
GSM585453	0	0	1	0	0				62					13.1127	7.1195	3.7713	11.8508	9.9475	8.8544	8.5564	4.6943	0
GSM585454	0	0	1	0	0				46					12.3912	7.7229	4.1418	12.4482	9.1285	9.0582	8.9403	5.0998	0
GSM585455	0	0	1	0	0				38					13.4162	6.3587	4.5348	12.0221	9.7957	8.7989	8.4708	5.1189	0
GSM585456	0	0	1	0	0				61					12.9685	7.4658	4.2333	12.5079	10.1906	8.6804	8.4604	4.5415	0
GSM585457	0	0	1	0	0				34					12.9970	7.1697	3.4061	12.3228	10.1142	9.2779	8.6038	5.3736	0
GSM585458	0	0	0	0	0				62					9.4406	6.1254	3.9551	11.8065	9.6253	9.9165	9.1584	5.2007	0
GSM585459	0	0	0	0	0				28					8.3930	5.5746	3.5500	13.1997	11.1447	9.6038	8.7468	4.9745	1
GSM585460	0	0	1	0	0				68					13.6482	5.2486	3.0141	13.0264	9.7198	8.9218	8.3884	4.3900	1
GSM585461	0	0	1	0	0				70					11.9967	7.4879	3.3585	12.0793	10.2309	9.0403	8.2843	5.1316	0
GSM585462	1	0	1	0	0				70					12.3870	8.9769	4.3261	12.3370	10.6662	8.8987	9.6529	5.6050	1
GSM585463	1	1	0	0	1				50					9.5006	9.2864	6.0420	11.9745	9.8337	8.3341	8.4463	5.5713	1
GSM585464	1	1	0	0	1				49					9.8000	8.6574	6.5506	12.4451	10.7775	9.3737	9.5316	5.2714	0
GSM585465	0	1	0	0	0				38					9.0691	7.5105	4.1929	12.2504	11.3404	9.9459	9.4897	4.9559	1
GSM585466	1	1	0	1	1				47					8.9398	10.5745	6.8445	11.9000	9.6526	9.1184	8.4677	4.7889	0
GSM585467	1	1	0	1	0				63					8.2859	11.9490	3.8331	11.7840	9.6686	9.0008	8.7364	4.3416	0
GSM585468	1	1	0	1	1				44					8.6208	10.5952	7.9652	12.9145	10.1394	8.6972	8.7102	4.6075	1
GSM585469	1	1	0	1	1				44					9.6025	11.8747	6.8027	12.6401	10.3507	8.8908	9.5664	5.2786	0
GSM585470	1	1	0	1	0				51					8.8621	12.1867	4.6527	12.0999	10.0738	8.5693	8.5372	5.5518	0
GSM585471	0	0	0	1	0				34					9.5054	12.5866	3.9940	12.4470	10.1075	8.6156	8.6130	5.5900	1
GSM585472	1	1	0	1	0				46					8.5286	11.8926	4.0216	12.1433	10.0662	9.1281	8.1488	4.7584	0
GSM585473	0	0	1	0	0				31					12.9818	5.8555	3.0117	12.1565	9.8314	9.7546	8.5469	6.0803	1
GSM585474	0	0	0	0	0				74					8.6535	6.6794	3.6563	11.7130	9.0792	9.2330	8.6829	3.9480	0
GSM585475	0	0	1	0	0				51					12.7913	8.9842	4.3018	11.4196	9.7857	9.1077	9.0454	5.1410	0
GSM585476	1	1	0	1	0				38					8.4213	10.7233	3.9856	11.8478	9.7826	8.7096	8.9943	5.5597	0
GSM585477	0	0	1	0	0				75					13.0396	9.3330	4.4145	11.7290	9.7880	8.1811	8.3020	5.2320	0
GSM585478	1	1	0	1	0				61					9.9428	9.8570	3.3912	11.9993	10.0887	9.0409	8.9124	5.8509	1
GSM585479	0	0	0	0	1				37					9.6152	7.9392	5.5476	12.4188	10.2326	9.0016	8.6802	4.7501	0
GSM585480	1	1	0	1	1				45					9.3055	9.5694	5.0661	12.3872	10.7421	9.7275	9.3314	5.2269	0
GSM585481	0	0	0	1	0				54					9.5164	9.5611	3.5173	12.4051	10.6992	9.7181	9.1305	6.0285	1
GSM585482	1	1	0	0	1				43					8.8031	9.4472	7.2710	12.1333	10.1771	8.7503	9.0956	5.0575	0
GSM585483	0	0	0	0	0				37					11.0034	8.4971	3.2027	11.6470	10.2608	9.3815	8.7732	5.0221	0
GSM585484	1	1	0	0	0				51					8.6184	8.0763	3.4374	12.2351	10.2186	9.2510	8.6082	5.0710	0
GSM585485	1	1	0	0	0				53					8.4295	7.8222	3.7720	12.0950	10.1279	9.7712	8.3033	4.5917	0
GSM585486	0	0	0	0	0				28					8.3528	8.1583	3.7482	11.6188	10.3070	9.2317	8.1874	5.7185	1
GSM585487	1	0	0	0	0				61					10.4323	9.2256	4.0197	11.4359	10.2246	9.0254	9.4703	4.6076	0
GSM585488	1	1	1	1	0				71					11.6764	11.5486	3.3352	12.5197	10.4340	9.2941	8.7120	6.6064	1
GSM585489	0	0	0	0	0				32					8.6279	6.5903	3.7777	12.1719	9.1145	9.9303	8.1355	4.7810	0
GSM585490	0	0	0	0	0				57					9.3527	7.8515	3.5903	12.2905	9.7429	9.2252	9.0262	4.7988	0
GSM585491	0	0	0	0	0				51					8.5822	5.4140	3.8182	12.4529	10.1005	8.6925	9.8918	4.7563	0
GSM585492	1	1	0	0	0				34					9.5104	9.4873	3.9159	12.2888	10.7861	8.8172	8.7355	5.1441	0
GSM585493	0	0	0	0	0				49					9.5105	9.0577	3.4994	12.2111	9.9998	8.3885	9.1333	4.9353	0
GSM585494	1	1	0	1	1				52					8.3694	10.7202	5.1895	11.7156	9.4043	8.8078	8.2741	5.0394	0
GSM585495	0	0	1	0	0				49					12.0262	5.6300	3.1926	12.4717	10.0821	8.9462	8.8045	4.8383	0
GSM585496	0	0	1	0	0				35					12.4813	4.0156	3.3632	11.7837	10.0908	8.9512	8.8733	5.8564	1
GSM65316	1	1	0	1	0	1	3	2.7	72	2.58	0	2.58	0	10.3017	11.9026	3.8700	12.7060	10.1312	9.1243	9.0934	4.6087	0
GSM65317	1	1	0	1	0	1	1	2.6	71	0.58	1	0.58	1	10.4386	10.3079	3.3009	12.6122	10.5981	9.3102	8.7597	5.0793	0
GSM65318	1	1	0	1	0	0	1	1.7	58	9.92	0	9.92	0	10.0828	10.1500	3.8800	12.2744	8.6689	8.9662	8.3648	4.6900	0
GSM65319	1	1	0	1	0	1	1	2	52	8.42	0	8.42	0	10.0287	9.8712	4.4664	12.2592	9.4199	9.0823	9.3604	4.7477	0
GSM65320	1	1	0	1	1	1	1	1.7	52	9.08	0	9.08	0	9.9330	10.5074	5.8219	12.2312	10.0609	8.9512	8.9493	4.5617	0
GSM65321	1	1	0	1	0		3	2.4	80	3.58	0	3.58	0	8.7182	12.1898	4.5417	12.1713	9.7629	9.1511	8.8141	4.8789	0
GSM65322	1	0	0	1	1	1	3	2.8	65	9.33	0	9.33	0	7.9933	12.5423	5.1749	12.5833	9.7080	9.6086	8.7619	5.2558	0
GSM65323	1	1	0	1	0		1	3.8	78	1.83	0	1.83	0	9.3911	9.8276	3.6864	12.1064	9.3214	9.6265	8.8240	5.0583	0
GSM65324	1	1	0	1	0	1	3	4.1	62	3.25	0	3.25	0	10.4190	11.2670	4.2482	13.3356	10.4110	10.0047	8.5653	4.7619	1
GSM65325	1	0	1	0	0	1	3	3.2	70	0.58	1	0.58	0	13.4083	8.3141	3.6355	13.1964	10.6457	9.7838	8.9169	5.4092	1
GSM65326	1	1	0	0	0	1	3	5	62	11.75	0	11.75	0	9.1253	9.2479	3.9823	12.2477	9.6963	9.4962	8.4947	4.5418	0

GSM65327				1	1	1									11.2248	11.6179	6.5994	13.0414	11.0495	9.3777	9.0906	4.8972	1
GSM65328				0	1	1									10.1184	11.3445	7.5148	12.1118	9.5296	9.5689	8.5965	4.7771	0
GSM65329		1	1	0	1	0	0	1	1.6	55	8.42	0	8.42	0	9.5392	10.4339	4.6127	12.1813	10.2463	9.9756	8.9307	5.0768	0
GSM65330		1	1	0	1	1	1	1	1.2	67	10.50	0	10.50	0	10.2803	11.6900	5.1486	12.4405	9.4162	9.1115	8.7668	4.5191	0
GSM65331		1	1	0	1	0	1	1	2.3	65	9.50	0	9.50	0	10.2242	10.8738	3.8431	12.2043	9.7088	8.6386	9.0320	4.5161	0
GSM65332		1	1	0	1	1	1	1	1.6	72	10.83	0	10.83	0	10.1284	11.2696	6.6367	12.1580	9.9051	8.7397	8.7815	4.7042	0
GSM65333		1	0	0	0	0	1	3	2.2	77	3.42	0	3.42	0	9.6003	8.1463	4.2613	12.4586	9.3280	9.4545	8.3834	4.5815	0
GSM65334		1	1	0	1	1	1	1	1	59	9.33	0	9.33	0	10.0520	11.1213	5.3949	12.5839	10.0204	8.4524	9.1506	4.7913	0
GSM65335		1	1	0	1	0	1	3	2.6	59	1.75	1	1.75	1	9.6530	11.9254	4.2494	12.1703	11.7422	9.0346	9.0547	5.8283	1
GSM65336		1	1	0	1	1	0	1	2.6	64	9.50	1	9.50	1	9.9203	10.8096	8.0002	12.3584	9.7843	9.8183	9.1544	5.2636	0
GSM65337		1	1	0	1	0	0		2.8	84	0.33	1			9.7480	10.4448	3.2561	12.4933	10.8049	8.4335	9.2324	5.1356	0
GSM65338		1	1	0	1	1	1	3	3.3	73	8.92	1	8.92	1	9.5141	11.6237	5.0798	12.3081	10.4032	9.0377	9.4830	4.9296	0
GSM65339		1	1	0	0	0	0	3	2.53	77	3.08	1	3.08	1	9.7089	7.9297	4.4632	12.4065	9.9217	7.8438	8.9807	4.6346	0
GSM65340		1		0	1	0		1	1.1	79	0.02	0	2.33	0	10.3946	11.6103	4.5148	12.6522	10.7471	9.0415	9.7983	4.9188	0
GSM65341		1		0	1	1	0		2	82	0.04	0	2.97	0	9.7566	11.8896	8.4693	12.5469	10.4644	9.3007	9.1110	5.4347	0
GSM65342		1		0	1	1	1		8.2	75	0.18	0			10.1882	10.8234	6.8993	12.4854	10.4979	10.2638	9.0876	5.5725	1
GSM65343		1		0	1	0	1	3	5.5	76	0.62	0			11.0741	11.2520	4.2078	12.7978	11.0031	9.2008	9.1052	5.2538	1
GSM65344		1		0	1	0	1	3	2.5	66	0.71	0	0.71	0	9.2954	12.8706	4.3025	12.7465	10.8622	9.1881	9.0983	5.2511	0
GSM65345		1		0	1	1	1	1	3.5	86	1.35	0	1.35	0	10.4242	11.5522	5.1197	12.8839	10.1037	9.3764	9.4000	5.4129	1
GSM65346		1		0	0	1	0	1	1.1	47	1.55	0	1.55	0	10.2370	9.1770	5.0170	12.3309	10.3298	8.6977	9.0133	5.4075	0
GSM65347		1		0	1	0	1	1	2.2	74	2.53	0			9.8387	10.6869	4.4952	12.8534	10.1128	9.1503	8.8572	5.2361	1
GSM65348		1		0	1	0	1	1	1.7	69	2.60	0	2.60	0	10.3114	13.3906	4.8186	12.2482	10.7028	8.6702	10.4970	5.1631	0
GSM65349		1		0	1	1	0	1	1.5	58	2.74	0			10.0739	9.8305	6.5031	12.2497	10.3813	8.6728	9.0770	5.2949	0
GSM65350		1		0	1	1	0	3	2.5	62	3.73	0			10.6329	10.0481	4.8466	12.5814	10.6402	10.4147	8.8175	5.2237	0
GSM65351		1		0	1	0	0	1	1.8	80	3.88	0	3.88	0	10.5214	10.8369	3.8001	12.9472	10.8833	9.3821	9.0654	5.4374	1
GSM65352		1		0	1	0	1	3	1.4	63	4.17	0	4.17	0	9.2351	10.7111	3.6784	13.0709	10.1751	9.3010	9.1255	5.1079	1
GSM65353		1		0	1	1	1	1	3.3	76	4.49	0	4.49	0	10.4973	12.2679	7.1791	12.8522	10.5616	9.6184	8.6585	5.5786	1
GSM65354		1		0	1	0	0	3	2.4	61	4.95	0	4.95	0	10.7677	12.9171	4.5564	12.7816	10.5744	9.0075	9.1479	5.9586	1
GSM65355		1		0	1	1	0	1	1.7	62	5.16	0	5.16	0	10.2799	10.2619	5.0606	12.5235	10.6509	9.0264	9.1033	5.4255	0
GSM65356		1		0	1	0	0	3	3.5	65	5.32	0	5.32	0	9.2813	12.3937	4.7514	13.1522	10.3608	8.9023	9.3468	4.8404	1
GSM65357		1		0	1	0	0	3	1.4	63	5.48	0	5.48	0	9.3371	11.1457	3.9451	12.5222	10.8617	8.6443	9.1394	5.3128	0
GSM65358		1		0	1	0	0	1	2	70	5.58	0	5.58	0	9.5447	11.0240	3.6268	12.2436	10.2960	9.7361	9.6342	5.1566	0
GSM65359		1		0	1	1	0	3	3.2	78	5.74	0	5.74	0	9.8103	11.9129	4.8836	12.9631	10.9508	9.1425	9.6093	5.1829	1
GSM65360		1		0	1	1	0	1	2	71	7.01	0	7.01	0	10.0228	12.2357	5.7093	12.5640	10.5031	9.7526	9.5236	5.1121	0
GSM65361		1		0	1	1	0	1	1.6	68	7.36	0	7.36	0	9.9862	11.6748	7.7452	12.7957	10.8709	8.9315	9.4998	5.3073	0
GSM65362		1		0	1	1	0	1	0.2	45	7.58	0	7.58	0	9.9627	10.3909	5.1848	12.8861	10.8660	9.0148	9.2001	5.4822	1
GSM65363		1		0	1	1	0	1	3.5	57	8.59	0	8.59	0	9.3117	12.2813	7.0552	12.5379	10.9140	9.9046	9.0598	5.1151	0
GSM65364		1		0	1	1	1	3	1.7	67	8.81	0	8.81	0	10.6379	11.6287	5.8905	12.4937	10.5007	8.6134	8.8410	5.4078	0
GSM65365		1		0	1	1	1	1	1.8	74	8.88	0	8.88	0	10.3204	10.9341	4.8730	12.5286	10.2910	9.0141	9.1611	5.7701	1
GSM65366		1		0	1	1	0	3	4	60	9.41	0	9.41	0	9.9915	12.4969	5.6599	12.3289	10.7171	8.9569	9.5924	5.0725	0
GSM65367		1		1	0	0	0	3	1.1	60	9.88	0	9.88	0	11.4113	8.6303	4.0127	12.6711	10.3959	9.2138	8.5072	5.1061	0
GSM65368		1		0	1	1	1	1	2.3	63	10.29	0	10.29	0	10.2696	12.4665	5.2040	12.5328	11.1714	9.0479	9.2843	5.6415	1
GSM65369		1		0	1	1	0	3	3.3	69	0.50	1			8.6795	12.2903	5.0867	12.4255	10.3078	9.2959	9.0882	5.5894	1
GSM65370		1		1	1	0	1	3	2.3	66	0.90	1	1.74	1	12.2358	12.1420	4.2793	12.3760	10.7038	8.9672	9.1891	5.4106	0
GSM65371		1		0	1	0	0	3	3	66	1.55	1	1.55	1	10.9304	12.1269	4.2769	13.1527	11.4852	9.6816	9.3359	6.1733	1
GSM65372		1		1	1	0	0	3	1.9	49	2.57	1	2.57	1	12.2944	11.9286	4.6543	12.7155	10.7926	9.3784	8.8473	5.4069	0
GSM65373		1		0	1	0	1	1	4.5	61	3.94	1	6.50	1	10.2137	12.3399	4.2712	12.7035	11.2437	9.8784	9.0888	5.5680	1
GSM65374		1		0	1	1	0	3	2.1	45	4.53	1	4.53	1	9.3613	10.5068	5.5202	12.3472	9.6748	8.2829	9.5849	4.9044	0
GSM65375		1		0	1	0		1	2	78	4.89	1			9.5875	10.6717	3.8157	12.4751	11.1972	9.6852	8.9365	5.4181	1
GSM65376		1		0	1	1	0	1	2	40	5.00	1	5.00	0	9.8190	10.8884	6.4932	12.5046	10.4345	8.4996	8.8841	5.2787	0
GSM65377		1		0	1	0	1	1	5	69	5.67	1	5.67	1	10.7961	12.5989	4.5213	12.4920	10.5544	8.8890	9.3034	5.1713	0
GSM65378		1		0	1	1	1	3	5.5	64	7.12	1	7.12	1	9.6296	11.4957	7.7448	12.4867	11.1530	9.6387	9.3033	5.0794	1
GSM65379		1		0	1	1	0	1	1.4	48	8.10	1	8.10	0	9.8568	11.0638	5.1609	12.5014	10.6782	9.6279	9.3287	5.7034	1
GSM655624	0	1	0	0	1	0		2							9.7337	12.6069	4.3773	12.6171	10.2824	7.8230	9.0651	4.9738	0
GSM655625	1	1	0	1	1	0		3							11.2268	10.2745	3.4306	11.4908	9.9759	6.3237	8.4582	4.3193	0
GSM655626	0	1	0	0	1	0		3							8.1334	10.4618	4.6327	12.4078	9.9724	7.8731	9.0627	5.0075	0
GSM655627	1	0	0	1	0	0		3							11.8770	4.4391	3.5745	12.5357	10.6431	8.4251	8.4205	4.5993	0
GSM655628	0	1	1	0	1	1		3							7.9651	12.1705	7.8128	12.5489	10.9890	8.9012	8.7716	5.4874	1
GSM655629	0	1	1	0	1	1		2							8.8894	10.8520	5.0989	12.3380	9.8744	9.1202	8.9976	4.6622	0
GSM655630	0	0	0	0	0	0		3							8.0705	5.8882	3.6927	12.0710	10.1662	8.6422	9.4273	4.8258	0
GSM655631	1	1	1	1	1	1		3							11.6245	11.9334	8.3361	12.0086	9.6791	8.8403	9.1871	4.7284	0
GSM655632	0	1	1	0	1	1		1							10.7949	11.7216	8.2612	12.6836	10.4456	9.0457	9.0070	5.2753	0
GSM655633	0	0	0	0	0	0		3							8.0123	8.3497	3.7618	12.3787	9.5131	7.8389	8.5550	4.9311	0
GSM655634	1	1	0	1	1	1		2							12.6717	11.6919	7.3231	12.7785	10.3955	8.9801	8.8954	4.7177	0
GSM655635	0	1	1	0	1	1		2							8.9368	10.6943	6.1376	12.4402	10.1456	8.8869	8.5865	4.8680	0
GSM655636	0	1	1	0	1	0		3							9.9991	12.2304	3.5336	12.3138	10.2406	7.5293	9.7831	6.5249	1
GSM655637	0	1	1	0	1	1		2							9.7509	12.7828	4.8475	12.5951	10.8668	9.6667	8.7404	5.4621	0
GSM655638	0	1	1	0	1	1		2							9.1453	11.8378	4.9904	12.5233	10.4660	8.5272	9.8435	5.0008	0
GSM655639	1	1	1	0	1	1		3							10.6627	11.2210	5.0864	12.3545	9.7866	5.9576	8.3982	5.3951	0
GSM655640	0	1	0	0	1	0		2							9.2915	11.1765	4.3473	12.7169	10.1061	7.8897	8.4870	5.4581	0
GSM655641	1	0	0	0	0	1		2							10.7992	7.2430	5.2312	12.3475	11.9520	10.4214	8.9430	5.2301	1
GSM655642	0	1	0	0	1	0		3							8.2172	11.7958	4.8193	12.5400	10.6499	9.1130	8.1626	4.6421	0
GSM655643	0	1	1	0	1	1		2							7.9187	12.0144	7.7106	12.4691	9.8203	7.9163	8.4224	4.9746	0
GSM655644	0	0	0	0	0	0		3							9.3986	6.3507	4.1109	12.2539	10.2081	9.3342	8.4838	5.3692	0
GSM655645	1	1	1	1	1	0		2							12.0236	10.5951	4.4049	12.8942	10.4438	8.4815	8.0351	5.2621	1
GSM655646	1	0	1	1	0	0		2							12.6333	7.5393	3.7704	12.5042	9.4149	8.7906	8.2502	4.7196	0
GSM655647	0	0	0	0	0	0		3							8.7290	6.8560	4.2637	12.2107	8.9404	7.4393	9.1024	5.1402	0
GSM655648	0	1	0	0	1	0		1							9.7695	10.3893	4.0060	12.1990	9.6707	9.3967	8.0132	4.6297	0
GSM655649	0	1	0	0	1	0		2							8.6757	12.5537	3.7089	12.9026	10.3229	8.6006	9.1781	4.4619	1
GSM655650	0	1	0	0	1	0		2							9.0612	10.9160	3.5497	12.2960	9.7009	8.7188	8.5777	4.8109	0
GSM655651	1	1	1	1	1	1		3							12.1515	10.8728	8.4369	12.6853	10.4488	9.3017	8.5208	5.1589	0
GSM655652	1	1	0	1	0	0		3							12.0248	8.8795	3.4206	12.7500	9.8560	8.2591	8.6082	4.5406	0
GSM655653	0	1	1	0	1	1									9.5759	10.7528	5.2064	12.5051	10.4748	8.9186	9.3992	4.8025	0
GSM655654	0	1	1	0	1	0		2							8.9403	10.7088	4.7976	12.5132	10.4219	9.6387	8.9786	4.7993	0
GSM655655	0	0	0	0	0	0									8.6579	5.4855	3.4739	12.1627	10.0531	8.6883	9.1636	4.9386	0
GSM655656	1	1	1	1	0	0		2							11.8427	8.6581	3.2894	11.5442	8.7672	7.4820	8.6807	4.4484	0
GSM655657	1	0	0	1	0	0		2							12.3893	6.7289	3.5653	12.1958	9.8499	9.1385	8.8388	4.8365	0
GSM655658	1	1	1	1	1	0		3							13.1933	10.3372	4.6393	12.4570	10.2176	8.4824	8.5231	4.3643	0
GSM655659	0	1	0	0	1	0		3							8.6260	10.1883	3.7370	12.6549	11.0271	9.1792	8.7778	4.5624	1
GSM655660	0	0	0	0	0	0		3							9.1177	6.2633	3.2839	12.0148	9.8719	8.8070	8.5468	6.0921	1
GSM655661	0	1	0	0	1	0		3							7.8257	12.2297	3.5067	12.8503	10.7270	9.5862	8.4657	5.5271	1
GSM655662	0	0	0	0	0	0		3							8.0492	5.8598	3.8878	12.0672	9.9219	9.3173	9.7201	4.8240	0
GSM655663	0	1	1	0	1	0		3							9.6463	11.8187	3.7698	12.7754	11.0692	8.8557	9.5682	5.4949	1
GSM655664	1	0	0	1	0	0		3							12.6708	5.6645	3.7383	12.4549	10.4669	9.6821	8.3318	4.9341	0
GSM655665	1	1	1	0	1	1		2							9.1848	10.4840	6.5336	11.2064	9.6052	7.3219	9.1631	5.0430	0
GSM655666	0	1	0	0	1	0		2							9.0163	10.9466	3.1161	12.6544	9.9725	9.2578	9.0976	4.9440	0
GSM655667	0	0	0	0	1	1		2							9.2700	11.6808	4.9264	11.4915	9.0154	7.0121	8.5737	4.9339	0
GSM655668	0	1	0	0	1	0		2							9.8033	10.6552	2.8284	12.8500	10.6900	9.0667	8.8975	4.9351	1
GSM655669	1	1	0	1	1	0		3							12.8901	10.8228	3.5783	12.6988	10.4292	9.0795	8.9555	4.6621	0
GSM655670	0	1	0	0	1	0		2							9.5058	10.1123	3.8050	12.4278	10.2416	8.8758	8.4651	5.3067	0
GSM655671	1	0	0	1	0	0		2							12.3868	6.4975	3.2397	12.7852	10.1786	8.9553	8.3093	4.7449	0
GSM655672	0	0	0	0	0	0		2							8.0388	6.6112	3.4979	12.4152	9.6346	8.5802	9.3903	5.0682	0
GSM655673	1	1	0	1	1	0		3							11.9834	13.0054	3.7616	12.2564	10.3969	8.0093	8.4429	4.8926	0
GSM655674	0	0	0	0	0	0		3							7.8227	5.6558	3.6072	12.0927	10.4544	9.3349	8.5028	4.7722	0
GSM655675	0	1	0	0	1	0		2							10.0476	12.1458	2.9140	11.1287	9.7537	8.1663	8.9022	5.8219	1

GSM655676	0	0	0	0	0	0		7.8047	8.8824	3.7581	12.0522	9.9430	9.6375	9.1588	4.8503	0
GSM655677	0	1	1	0	1	1	2	8.6880	11.8847	5.5436	12.0205	8.7540	7.7484	8.5998	5.2673	0
GSM655678	0	1	1	0	1	1	2	8.5299	10.8171	5.7862	12.2642	9.2540	8.7057	8.5686	4.6801	0
GSM655679	0	1	1	0	1	0	2	9.1916	12.1891	3.3051	12.3979	10.8223	8.8854	8.9351	4.9625	0
GSM655680	1	1	0	1	1	0	2	11.2455	10.4911	3.7679	12.4371	10.4633	9.6807	9.1568	4.6620	0
GSM655681	1	0	0	1	0	0	3	12.4164	8.9902	3.3031	12.7078	11.1320	9.4122	8.7489	5.8241	1
GSM655682	1	0	0	1	0	0	3	12.9052	6.8079	4.0720	11.0670	9.8546	6.6873	9.5980	5.1803	0
GSM655683	1	0	0	1	0	0	2	12.4930	6.0157	3.1277	12.4473	10.0578	8.2109	8.2599	5.2458	0
GSM655684	0	1	1	0	1	0	1	8.7899	10.1958	4.3191	12.6405	10.6303	9.4862	9.5071	4.9638	0
GSM655685	1	1	0	1	1	0	3	11.7601	10.2678	3.9137	12.2033	9.9552	9.0160	9.0546	4.7130	0
GSM655686	1	0	1	1	1	1	3	12.7115	10.4756	5.2538	12.3926	10.0446	9.2396	9.2335	4.9299	0
GSM655687	0	1	0	0	0	0	3	7.7152	8.8092	3.5619	12.1572	10.0571	9.7182	9.8578	4.4604	0
GSM655688	1	0	0	1	0	0	3	12.3249	4.8237	3.7565	12.4790	9.2204	9.0644	8.5644	4.5355	0
GSM655689	1	0	0	1	0	0	2	12.4753	7.5393	3.9940	12.0390	9.6142	8.5187	8.2985	4.4577	0
GSM655690	0	1	1	0	1	0	3	8.1145	10.7711	4.1261	12.2627	9.2846	9.0357	8.3050	4.9235	0
GSM655691	1	0	0	1	1	0	3	12.4894	11.1248	3.6118	12.6273	9.5483	9.2136	8.8210	4.7162	0
GSM655692	0	0	0	0	0	0	3	8.4626	7.5348	4.3476	11.9074	9.4087	8.4826	8.0418	4.6390	0
GSM655693	0	1	1	0	1	1	3	8.1850	10.4147	5.3321	12.3988	10.2778	8.4726	8.8845	5.2585	0
GSM655694	1	0	0	1	1	0	2	11.1831	10.9278	3.7624	12.4109	10.3376	8.4872	7.7937	5.0783	0
GSM655695	0	1	1	0	1	0	3	8.4027	10.9943	4.1600	12.1226	10.0045	8.8064	8.4057	5.2219	0
GSM655696	0	1	1	0	1	0	1	8.6826	11.5134	4.7956	12.2485	10.0925	9.0639	8.9349	4.9081	0
GSM655697	1	1	1	1	1	0	2	12.2381	11.3636	3.7399	12.8839	10.7391	9.4310	8.7937	5.1019	1
GSM655698	1	1	1	0	0	0		10.4338	9.4131	4.2028	12.3306	10.1699	9.5623	8.5391	4.7640	0
GSM655699	0	0	0	0	0	0	2	8.1949	8.9261	4.2073	12.2421	10.1708	9.3390	8.4221	4.7782	0
GSM655700	1	1	1	0	1	0	2	9.3094	12.6411	3.2798	12.4035	10.6331	9.3776	8.3492	5.8883	1
GSM655701	1	0	1	1	1	0	2	12.1241	10.3093	4.5126	12.3831	9.8610	8.5591	8.0449	5.0186	0
GSM655702	1	0	0	1	0	0	3	12.3191	7.3243	3.6750	12.4088	9.6305	9.2374	8.0349	5.2024	0
GSM655703	0	0	0	0	1	0	2	8.5943	12.2063	3.6766	12.4007	10.7089	8.2088	8.8207	5.6277	1
GSM655704	0	0	1	0	1	1	3	9.0493	10.4527	7.8537	12.5831	10.5109	9.4763	9.1971	5.2945	0
GSM655705	0	1	1	0	1	1	2	8.5750	11.8067	6.5204	12.3980	9.0725	9.3478	8.5504	5.3235	0
GSM655706	0	1	1	0	1	1	2	8.5658	10.5474	7.9488	12.2152	9.9272	9.7629	8.6732	4.9929	0
GSM655707	0	0	0	0	0	0	3	7.1188	7.6224	3.6287	12.9234	9.7726	8.4973	8.1937	5.3127	1
GSM655708	0	1	0	0	1	0	3	9.7456	10.1479	4.0909	11.3998	9.4525	7.1204	8.6518	5.6233	1
GSM655709	0	1	1	0	1	1	1	8.5066	10.9858	7.4178	12.4452	10.0525	8.6982	9.1879	4.9069	0
GSM655710	1	1	1	1	1	1	3	11.5304	11.8690	5.3641	12.5535	10.3620	8.6193	8.5872	5.2173	0
GSM655711	0	0	0	0	0	0	3	6.0522	6.2102	3.7266	12.0265	9.4338	9.5859	7.8047	4.8771	0
GSM655712	0	1	1	0	1	1	1	9.2482	10.2917	7.8661	12.3566	10.5476	9.4551	8.8553	5.2939	0
GSM655713	0	0	0	0	0	0	3	7.9003	9.4366	3.7271	11.9263	9.4933	8.3145	8.7492	4.6073	0
GSM655714	0	1	1	0	1	1	2	8.4088	13.8575	7.3659	12.9697	10.2375	9.1472	9.2872	5.1357	1
GSM655715	1	0	0	1	0	0	3	11.7374	6.5471	3.5939	12.0107	9.8448	8.7428	8.0644	4.7706	0
GSM655716	0	0	0	0	1	1	3	8.5591	10.7283	5.8856	12.6972	10.3478	8.7944	8.7098	5.2897	0
GSM655717	0	1	1	0	1	0	2	8.3126	11.5596	4.8055	12.2446	10.4879	9.4941	8.5347	4.8652	0
GSM655718	0	1		0	1	1	3	9.1842	12.0603	6.1108	12.2695	10.6800	8.6698	8.4097	5.3766	0
GSM655719	0	1	1	0	1	1	2	8.7966	11.8020	4.8417	13.0244	10.5121	9.6433	8.7524	5.1950	1
GSM655720	0	0	0	0	0	0	3	7.6854	4.2538	3.4715	12.5871	10.1283	8.7158	8.8396	6.4514	1
GSM655721	0	1	1	0	1	0	1	9.1534	10.7870	4.6866	12.6461	10.6069	8.5861	9.2882	4.9541	0
GSM655722	1	1	1	0	1	1	3	10.2269	11.4996	5.7142	12.5866	10.2497	8.3376	9.1436	5.0305	0
GSM655723	0	1	1	0	1	1	3	8.8187	12.4232	5.6678	12.4335	10.5406	7.8578	8.9265	4.4789	0
GSM655724	0	1	1	0	1	1	3	8.4761	13.5487	6.9775	11.9127	9.6461	9.5288	8.0087	4.2076	0
GSM655725	0	1	0	0	1	0	2	8.5063	12.3443	3.7056	12.2490	10.4519	8.8264	8.5945	4.3015	0
GSM655726	0	0	0	0	0	0	3	7.4047	8.0874	3.5770	12.2464	10.3606	8.6335	9.9289	4.9027	0
GSM655727	0	0	0	0	0	0	3	7.5878	3.5940	3.2568	12.5841	10.4274	9.8803	9.3887	5.5886	1
GSM655728	0	0	0	0	0	0	3	8.3705	8.1452	4.0961	12.6283	10.1062	8.4145	9.6751	5.1195	0
GSM655729	0	1	1	0	1	1	2	9.5975	12.0317	6.7791	11.8566	10.2277	8.2026	8.6226	4.1553	0
GSM655730	0	0	0	0	0	0	3	8.6971	6.8974	3.4565	13.0298	10.2862	8.9019	8.4695	4.2090	1
GSM655731	0	0	0	0	0	0	3	7.9463	5.1055	3.5335	13.0340	9.9437	8.6062	9.4473	4.5515	1
GSM655732	0	0	0	0	1	0	3	7.7435	9.7339	3.9810	12.2443	10.3903	8.7783	9.6546	5.2115	0
GSM655733	0	1	1	0	1	1	3	9.4418	10.9058	6.4297	12.0069	10.0330	8.6736	8.8736	4.5693	0
GSM655734	0	1	1	0	1	1	2	8.6708	9.9344	6.7996	12.4630	10.3363	9.1485	9.1688	4.4911	0
GSM655735	0	0	0	0	0	0	3	7.1363	7.0720	4.1160	12.5791	9.9258	8.6077	8.5524	4.3538	0
GSM655736	0	1	1	0	1	1	1	9.0973	11.4701	5.0081	12.6297	10.2188	8.8624	8.6150	4.2593	0
GSM655737	0	1	1	0	1	0	1	9.1775	10.9691	3.4842	12.6994	10.4537	9.0305	8.5956	4.4352	0
GSM655738	0	0	0	0	0	0		8.3537	5.2103	3.5216	12.6343	10.1547	9.7881	8.1395	4.4873	0
GSM655739	0	1	1	0	1	1	2	9.1466	11.5934	5.7352	11.9156	9.9278	8.1744	8.5426	4.5376	0
GSM655740	0	1	1	0	1	1	1	9.1698	11.0952	6.8574	12.1890	9.9121	8.6438	8.4811	4.5107	0
GSM655741	0	0	0	0	0	0	3	7.3973	6.1370	3.9209	12.0714	10.1490	8.5891	9.2195	4.8418	0
GSM655742	0	0	0	0	1	1	3	9.9291	11.9983	4.9463	13.1507	11.2285	9.4206	8.8218	4.7414	1
GSM655743	0	0	0	0	0	0	3	7.6654	7.2956	4.1829	11.6693	9.4144	8.5040	8.7470	3.4761	0
GSM655744	0	0	0	0	0	0	3	7.4743	4.5076	2.8623	11.7255	9.4439	8.8757	7.4246	3.5637	0
GSM655745	0	0	0	0	0	0	3	6.9123	7.7221	3.9492	12.5239	9.2611	8.2268	8.2418	3.8792	0
GSM655746	0	1	1	0	1	1	3	9.4003	12.1640	7.8624	12.5736	9.1328	8.5447	8.7332	3.8011	0
GSM655747	0	1		0	1	0	2	9.1729	11.6935	3.7730	13.1497	10.4007	9.6341	9.0421	5.9164	1
GSM655748	0	0	0	0	1	1	3	8.2675	11.0074	5.5760	12.9770	10.8224	8.7238	9.4050	4.1174	1
GSM655749	0	1	1	0	1	1	3	7.9552	12.2056	8.4181	12.4875	9.6086	8.1198	8.7803	4.3400	0
GSM655750	0	0	0	0	1	1	2	9.9725	10.7172	8.4366	12.9526	10.8352	9.6499	8.9472	4.4417	1
GSM655751	0	1	1	0	1	1	3	9.6092	11.8214	4.9186	12.5648	10.5816	8.5743	8.4244	4.6757	0
GSM655752	0	1	1	0	1	1	2	8.4207	13.2818	7.7806	13.0499	10.4980	8.8779	8.7927	4.1775	1
GSM655753	0	1	0	0	1	0	3	8.2202	11.3692	3.7168	12.9120	10.1625	9.0771	9.0720	4.6269	1
GSM655754	0	0	0	0	0	0	3	7.6994	8.3599	3.8143	12.2019	9.8159	9.6194	8.2174	4.2102	0
GSM655755	0	1	1	0	1	1	3	7.7483	11.7785	6.8278	12.3698	9.8309	8.9364	8.5676	4.3851	0
GSM655756	0	1	0	0	1	0	2	8.8081	11.2714	3.2221	12.4111	9.9660	9.0226	8.0501	4.5770	0
GSM655757	0	1	0	0	1	0	3	9.7001	12.1588	3.5802	12.1505	9.5635	8.5279	8.4477	4.0178	0
GSM655758	0	1	1	0	1	0	3	8.5795	10.9040	3.8360	12.2703	10.3125	8.5998	8.7679	4.3671	0
GSM655759	0	0	0	0	0	0	3	8.4661	6.2168	3.9317	12.3873	10.1759	8.6404	8.6932	4.1175	0
GSM655760	0	0	0	0	0	0	3	7.8432	8.8408	4.1821	12.4044	10.1901	8.3542	8.7719	4.2090	0
GSM655761	0	1	0	0	1	0	3	9.7926	12.7087	3.6549	13.1639	10.8543	9.3256	8.4432	4.9128	1
GSM655762	0	0	0	0	0	0	3	7.5871	4.8586	4.2413	12.3953	9.9300	8.8499	8.5133	5.0076	0
GSM655763	0	1	1	0	1	1	3	8.4538	10.3114	5.8914	12.2855	9.7785	9.1471	8.9799	4.2502	0
GSM655764	0	1	1	0	1	0	2	8.6436	11.2160	3.6994	12.6007	9.9631	8.9686	8.6964	4.3573	0
GSM655765	0	1	1	0	1	1	2	9.5453	11.9793	6.6502	12.2685	9.7191	9.7537	8.7575	4.1218	0
GSM655766	0	1	1	0	1	0	2	9.6810	12.0229	4.5395	12.3000	10.7617	8.8241	8.7656	5.2211	0
GSM655767	1	0	0	1	0	0	3	12.9547	8.1891	3.4171	12.1243	10.0431	9.1628	8.6517	4.3269	0
GSM655768	1	0	0	1	1	0	2	12.8561	9.9187	2.9869	12.7630	10.0078	9.4159	8.2933	4.3362	0
GSM655769	1	0	0	1	0	0	3	12.1801	8.8566	3.9484	12.4224	9.6444	9.6658	8.2064	4.0258	0
GSM655770	1	1	1	1	1	1	3	11.6663	11.2271	7.9826	12.5060	10.2089	8.6184	8.4158	4.1241	0
GSM655771	1	0	0	1	0	0	3	11.9039	6.3976	3.5274	12.6145	10.0691	9.1507	8.8789	3.8281	0
GSM655772	1	1	1	1	1	0	2	12.1497	10.6455	4.4875	12.7020	10.3648	9.5557	8.5425	4.3249	0
GSM655773	1	1	1	1	1	1	3	13.0141	11.5816	5.4603	12.2556	9.4419	8.7879	8.3919	4.3244	0
GSM655774	1	1	1	0	1	1	3	10.9967	10.9716	5.2705	12.0847	10.6282	8.8651	8.4024	4.0849	0
GSM655775	1	0	0	1	0	0	3	12.0508	8.7748	3.5704	12.6925	10.3727	9.1040	7.9491	4.0105	0
GSM655776	1	0	0	1	0	0	2	12.9399	5.5227	2.8173	12.8696	10.2029	9.6507	8.8600	4.3582	1
GSM655777	1	0	0	1	0	0	3	12.3255	7.6778	3.6934	12.2164	9.9562	8.6996	8.4380	4.0927	0
GSM655778	1	1	1	0	1	1	2	8.5621	10.5050	6.1865	12.3331	9.9728	9.2898	8.9473	5.1006	0
GSM655779	1	0	0	1	0	0	3	12.6773	8.1159	3.6290	12.7572	9.5898	8.3758	9.4139	3.9508	0
GSM655780	1	1	1	1	1	1	3	12.1844	11.9161	5.3367	12.4447	10.3787	8.4136	8.9615	3.6457	0

GSM655781	1	0	0	1	0	0		3							12.8955	7.6735	3.7136	12.0304	10.0383	9.3781	8.1083	4.1464	0
GSM655782	1	1	1	1	1	1		3							11.2671	11.3094	4.8611	12.8883	10.9244	9.1891	8.7858	4.4909	1
GSM655783	1	0	0	1	1	0		3							13.1254	10.8139	3.8791	12.6217	10.3695	8.8886	9.1903	3.9508	0
GSM655784	1	1	0	1	0	0		3							11.8928	9.3094	3.3345	12.1978	10.1906	8.9676	8.4090	4.2986	0
GSM655785	1	1	1	0	1	1		3							9.2617	9.8563	5.2959	12.1989	10.0149	9.2703	9.4476	4.8868	0
GSM655786	1	0	0	1	0	0		3							12.8575	6.9585	3.5461	12.6294	10.0912	9.1743	8.7978	4.8161	0
GSM655787	1	0	0	0	0	0		3							10.1711	6.6070	3.2696	11.8986	9.7158	9.5381	8.4449	3.6356	0
GSM655788	1	0	0	1	0	0		2							13.0116	6.9309	3.2607	12.3149	10.4557	8.7771	8.4055	4.5539	0
GSM655789	1	1	0	1	1	0		3							11.3415	12.5576	3.3356	12.5705	10.5751	8.3391	8.3056	4.6644	0
GSM655790	0	1	0	0	1	0		2							9.2709	12.3108	3.6573	12.8181	10.4769	9.2959	8.6888	5.6101	1
GSM655791	0	1	1	0	1	1		2							9.1075	11.3364	7.1872	12.2521	10.1144	9.0464	9.5061	4.3664	0
GSM655792	0	1	0	0	1	0		2							9.5985	10.7330	3.3760	12.5571	10.4817	9.2764	8.1829	4.5019	0
GSM655793	0	1	1	0	1	1		2							8.9118	11.8807	6.1670	12.3933	9.7942	8.7872	9.2942	4.8360	0
GSM655794	0	1	0	0	1	0		2							9.7790	12.1697	3.5711	12.7549	10.6824	9.1840	8.2783	4.6197	0
GSM655795	0	1	0	0	1	0		2							8.9151	11.7594	4.7594	12.3997	10.2840	8.9293	8.6129	4.5482	0
GSM655796	0	1	0	0	1	0		2							9.2259	11.3275	3.4040	12.2836	9.9351	8.7760	7.8843	4.0915	0
GSM655797	0	1	1	0	1	0		3							8.5339	11.8100	3.8401	12.3348	10.5548	8.9168	8.8237	6.2480	1
GSM655798	0	1	1	0	1	1		1							9.7619	12.3291	9.1432	12.6994	10.2753	8.6523	9.0164	4.7141	0
GSM655799	0	1	1	0	1	0		3							8.0870	10.7061	4.4840	12.6172	10.2251	8.0881	8.8320	4.3553	0
GSM655800	0	1	1	0	1	0		3							8.5126	12.2216	4.2753	12.9324	10.4582	9.5338	8.8017	4.6249	1
GSM655801	0	1	1	0	1	1		1							8.1806	10.9890	6.9467	12.1520	9.8278	8.0741	8.9354	4.1869	0
GSM655802	0	0	0	0	0	0									9.0401	8.0559	4.1160	12.1643	9.5112	9.0022	8.8773	4.4100	0
GSM655803	1	1	1	1	1	1									13.0626	10.7280	5.6005	12.6582	10.5876	8.7131	9.0516	4.6601	0
GSM655804	1	1	1	0	0	0		2							7.4264	6.6145	3.7511	11.8878	8.9974	10.5331	7.7674	4.3563	0
GSM655805	1	1	1	1	1	1		2							11.1703	11.5067	5.8834	13.3209	10.7682	9.5767	9.3044	4.5779	1
GSM655806	1	1	0	1	1	0		2							13.5913	11.4173	3.9303	12.2913	9.7594	8.4249	8.2531	4.0945	0
GSM655807	1	1	0	0	1	0		2							11.0427	11.1571	3.5360	12.9959	10.7953	9.0820	8.3545	3.6667	1
GSM655808	1	1	0	1	1	0		2							12.0576	12.8005	3.7436	12.2515	9.5210	8.0452	8.3158	4.0251	0
GSM655809	1	0	0	1	0	0		3							12.3212	7.7168	4.3197	12.4881	9.4838	8.1148	8.4649	5.1481	0
GSM655810	1	1	0	0	1	0		2							9.9274	10.3147	3.6844	12.4495	10.1046	9.0000	8.3880	4.3384	0
GSM655811	1	1	1	1	1	1		2							12.0160	10.8354	7.4716	12.5701	10.0661	8.7533	8.6332	4.1826	0
GSM655812	1	1	1	1	1	0		2							12.0448	10.5227	4.0511	12.6793	10.6115	9.2402	8.8331	4.7995	0
GSM655813	1	1	0	1	1	0		2							13.0544	10.2224	4.2535	12.4197	10.2149	9.5911	8.7009	4.2522	0
GSM655814	0	0	0	0	0	0		3							7.9244	8.9962	3.7348	12.1785	9.7284	8.9337	7.6254	3.6579	0
GSM655815	0	1	1	0	1	1		2							9.5173	10.3767	5.8409	12.2988	10.0972	9.6911	8.6219	4.5049	0
GSM655816	0	0	0	0	0	0		3							8.6935	9.3840	3.5535	12.5441	10.6725	9.3068	8.8785	4.0993	0
GSM655817	0	0	0	0	0	0		3							8.8888	7.5748	3.9581	12.9782	11.1654	8.4741	8.3940	4.4928	1
GSM655818	0	0	0	0	0	0		3							8.4813	7.4071	3.6762	12.4409	10.1357	9.0352	8.8700	4.8752	0
GSM655819	0	1	1	0	1	1		3							9.1204	11.7440	7.7085	12.4780	10.4866	8.7957	9.8809	4.3231	0
GSM655820	1	0	0	1	0	0		3							12.4400	6.5998	3.6845	11.9797	9.8850	8.6775	8.6895	4.2025	0
GSM655821	1	0	0	1	0	0		3							12.7510	6.5422	3.7277	12.2696	10.1580	9.1372	7.7531	3.8582	0
GSM655822	1	0	0	1	0	0		3							12.9130	7.2506	3.3329	12.7410	10.3238	9.0491	8.3408	4.3206	0
GSM655823	1	0	0	1	0	0		2							12.2073	7.8199	4.0362	12.6338	10.0141	9.9775	8.2332	4.2208	0
GSM655824	1	1	1	1	1	0		3							12.3640	9.8002	3.8258	12.6319	10.6542	9.4491	8.7937	4.3049	0
GSM655825	1	1	1	1	1	1		3							11.8280	10.8217	7.0819	12.3900	9.6730	9.1114	9.3265	3.9782	0
GSM655826	0	0	0	0	1	0		2							10.1319	9.6049	3.7095	11.7590	8.6878	7.8893	8.2389	5.1327	0
GSM655827	0	0	0	1	0	0		2							12.5142	6.4239	3.5180	10.7476	8.4939	6.5852	8.3344	5.1727	0
GSM655828	0	0	0	0	0	0		3							8.2130	5.8254	3.5138	11.1265	8.8391	5.6751	8.4126	5.7329	1
GSM655829	0	0	0	0	0	0		3							8.6714	8.5602	3.8463	12.1348	9.5722	8.8590	9.0023	4.4477	0
GSM655830	0	1	1	0	1	1		2							8.9944	11.1824	5.1837	12.3982	10.2648	8.1638	9.3163	4.2843	0
GSM655831	0	1	0	0	1	0		3							8.6536	9.8572	3.8709	11.9678	9.0298	8.3633	8.3581	4.8660	0
GSM655832	0	1		0	1	0		2							10.3014	13.6041	3.6711	12.1944	10.4708	9.6451	8.4295	4.8814	0
GSM655833	0	0	0	0	0	0		3							7.9614	7.8517	3.4432	12.2381	10.6925	8.5449	9.4150	4.9285	0
GSM655834	0	0	0	0	0	0		3							7.7405	6.7159	3.9241	12.3459	10.8659	8.8503	8.8691	4.3427	0
GSM655835	0	1	1	0	1	1		2							9.4171	11.5314	6.7363	11.9169	9.8274	7.8117	9.1298	5.1984	0
GSM655836	0	1	1	0	1	1		1							8.1231	12.1962	7.4458	12.8669	10.4033	9.3834	8.9592	4.0306	1
GSM655837	0	1	0	0	1	0		2							8.9706	11.1764	3.8063	12.4283	10.1254	8.7754	8.1200	4.3138	0
GSM655838	0	1	1	0	0	0		1							9.4327	9.2396	4.2976	12.4378	10.8317	8.1880	10.4035	4.3503	0
GSM655839	0	1	0	0	0	0		2							10.1415	9.2480	3.3276	12.6773	9.7390	8.3243	8.4583	4.2758	0
GSM655840	0	1	1	0	1	1		3							8.7407	12.0092	5.8811	12.4660	10.1000	8.1480	8.7921	3.9605	0
GSM655841	0	0	0	0	0	0		2							8.4952	8.0832	3.8782	12.6260	10.0131	8.7918	8.3025	4.7686	0
GSM655842	0	1	1	0	1	1		1							10.3716	11.1789	6.3573	11.9784	9.4803	8.2351	9.5696	5.1319	0
GSM655843	0	1	1	0	1	1		3							10.3997	12.7709	5.5943	11.5080	9.8801	6.3306	10.1183	5.9940	1
GSM655844	0	0	0	0	0	0		3							8.1726	7.7939	3.6265	11.9716	10.1595	8.8051	9.3381	4.1190	0
GSM655845	1	1	1	1	1	0		3							11.8240	11.4333	4.7654	11.0694	9.1985	4.6476	8.7382	6.3634	1
GSM655846	1	1	0	1	0	0		3							12.6286	9.0151	3.9594	11.7539	9.0667	7.9585	8.4214	5.7328	1
GSM655847	1	0	0	0	0	0		3							10.8387	6.4129	3.3235	11.8599	9.8499	8.0286	9.0341	6.0869	1
GSM655848	1	1	1	1	1	1		3							12.3293	11.2087	6.2513	12.6286	9.9419	8.7063	8.9791	4.2172	0
GSM655849	1	1	0	1	1	0		3							11.9646	12.6187	3.3889	12.3761	11.9810	7.9738	8.9361	4.7915	1
GSM65752		0	0	0	0	0	0	3	1.2	40	6.25	0	6.25	0	9.4300	5.2867	3.9577	12.5848	8.6017	9.3182	8.4929	4.7987	0
GSM65753		1	1	0	1	0	0	1	1.3	46	7.33	0	7.33	0	10.3491	10.1797	4.5199	12.3354	9.7427	8.2202	8.8719	4.7014	0
GSM65754		1	1	0	0	0	0	1	6	37	1.17	1	1.17	0	10.0949	9.0657	4.1541	12.7012	10.1230	9.2285	8.4128	4.6932	0
GSM65755		1	1	0	1	0	0	3	3.3	41	0.50	1	0.50	1	9.8590	10.8259	4.1312	12.9475	9.6824	8.4628	8.5186	4.7409	1
GSM65756		1	1	1	1	0	0	3	3.2	38	3.08	1	3.08	1	12.6135	9.8272	4.1828	13.1107	10.2292	9.1723	9.0335	4.7592	1
GSM65757		1	1	1	0	0	0	2	1.6	34	10.83	0	10.83	0	13.1623	7.9918	3.6666	12.0506	10.0124	9.6756	8.6211	5.1666	0
GSM65758		1	1	0	1	0	0	2	2.1	46	5.00	1	5.00	1	9.9487	10.3520	4.3482	12.0287	9.3169	9.7205	8.4723	4.7568	0
GSM65760		1	1	1	1	1	0	3	2.2	57	1.92	1	1.92	1	12.0516	10.6117	5.3122	12.3786	10.1626	9.5638	9.0335	5.1522	0
GSM65761		1	1	0	1	0	0	2	2.8	63	2.00	1	2.00	1	9.2068	11.5577	4.6646	12.2006	10.3057	10.6118	8.3766	5.0455	0
GSM65762		1	1	1	1	0	0	2	1.7	54	2.42	1	2.42	0	13.1470	10.0514	3.9810	12.8582	10.2951	9.4810	8.6530	4.6095	1
GSM65763		1	1	0	1	0	0	2	1.2	61	7.75	0	7.75	0	10.0117	9.9301	4.2115	12.0497	10.0397	9.0069	8.7700	4.6739	0
GSM65764		1	1	0	0	0	0	2	2.1	40	5.50	0	5.50	0	9.1066	8.4280	3.7591	12.3261	10.1616	8.6317	8.5363	5.1071	0
GSM65765		1	1	0	1	1	0	2	1.5	57	6.91	0	6.91	0	10.2183	11.4379	5.2322	12.5094	9.6273	8.8622	8.8866	4.7572	0
GSM65766		0	1	0	0	0	0	1	1.3	57	9.99	0	9.99	0	8.9933	8.8597	4.0785	12.1555	10.0344	9.6932	8.5208	4.6927	0
GSM65767		1	1	0	1	1	0	1	0.8	49	6.16	0	6.16	0	9.7712	9.8685	5.5873	12.0170	9.8792	9.1436	8.6417	4.7156	0
GSM65768		0	0	0	0	0	0	2	2.3	62	9.83	0	9.83	0	9.5878	5.8242	3.8185	11.7414	9.9514	9.2108	8.5567	5.0727	0
GSM65769		1	1	1	1	1	0	3	2.3	42	8.58	1	8.58	0	11.3513	10.1220	5.5861	12.3762	9.7368	9.3436	8.7672	4.7883	0
GSM65770		1	1	0	0	0	0	1	1.8	41	6.83	1	6.83	1	9.8157	8.5757	4.4079	12.2745	9.9618	9.0149	8.8954	4.8451	0
GSM65771		1	1	0	1	0	0	1	1	63	8.66	0	8.66	0	9.9284	10.1448	4.3555	12.0109	8.7879	9.4867	8.7685	4.6798	0
GSM65772		0	0	1	0	0	0	2	1.4	60	9.58	0	9.58	0	12.6638	7.3191	4.1364	11.9917	9.6759	9.4253	8.6693	4.5905	0
GSM65773		1	1	0	1	0	0	1	1.4	65	5.91	0	5.91	0	10.6661	11.2920	4.0048	12.1064	10.7666	8.3351	9.1166	4.9840	0
GSM65774		1	1	0	1	0	0	2	1.6	44	5.16	0	5.16	0	8.1565	10.0336	4.0932	12.9156	11.1571	9.2578	9.4602	5.2118	1
GSM65775		1	1	0	0	1	0	2	2.3	54	10.08	0	10.08	0	8.9449	9.4095	5.3089	12.5342	9.8253	9.2504	8.9178	4.7469	0
GSM65776		1	1	0	1	1	0	1	2.2	59	4.41	1	4.41	1	9.8889	10.9824	7.0310	12.0067	10.1318	8.6242	8.9709	4.4912	0
GSM65779		1		0	1	0	0	1	2	42	7.66	0	7.66	0	9.6084	9.9781	4.2904	12.0773	9.6747	8.6352	8.4168	4.3673	0
GSM65780		1	0	0	1	0	0	1	1.2	57	9.08	1	9.08	0	10.2741	10.5843	3.5518	12.1995	10.0765	8.1546	8.9024	4.5705	0
GSM65781		1	1	0	1	0	0	1	1.3	52	9.50	0	9.50	0	10.2379	9.7500	3.3583	11.7210	10.1333	8.1546	8.6185	4.8779	0
GSM65782		1	1	0	1	0	0	1	2.2	56	6.41	1	6.41	1	10.1353	10.4336	3.7179	12.1574	9.7827	9.7180	8.2941	5.0359	0
GSM65783		1	1	0	0	1	0	1	1.1	49	9.16	0	9.16	0	9.4637	9.5124	5.6546	12.1793	9.0680	9.1753	8.6898	4.7289	0
GSM65784		1	1	0	1	1	0	2	1.6	64	2.16	1	2.16	0	8.6078	12.2873	8.0256	12.4318	10.2630	9.1625	8.4938	5.3615	0
GSM65785		1	1	0	0	0	0	1	1.8	50	6.00	0	6.00	0	9.1802	9.1221	3.3515	12.2150	9.6690	8.9594	8.6952	4.9406	0
GSM65786		1	1	0	1	1	0	2	1	56	4.16	0	4.16	0	9.3698	10.4617	6.3306	12.3235	9.9183	8.9437	9.1205	5.5308	0
GSM65787		1	1	0	1	0	0	1	2.5	57	2.92	0	2.92	0	10.4311	9.7930	3.6965	11.9879	10.1759	9.2276	8.7148	4.9748	0
GSM65788		1	1	0	1	1	0	2	2.3	55	6.00	0	6.00	0	9.9568	9.6384	6.1988	12.1347	10.0428	10.1331	9.1354	4.4017	0
GSM65789		1	1	0	0	0	0	1	1	65	6.75	0	6.75	0	9.6005	8.0290	3.7204	12.4841	9.7566	9.3844	8.9816	4.6722	0
GSM65790		1	1	0	1	0	0	2	2.1	58	4.50	0	4.50	0	9.1252	11.7387	4.4878	11.7830	9.6610	8.2157	8.8793	4.5263	0

GSM65791	1	1	0	1	0	0	1	2.2	58				8.83	0	8.83	0	9.6958	10.9323	3.9095	12.0317	8.8381	8.1886	8.7861	4.7525	0
GSM65792	1	1	0	0	0	0	2	1.5	32				8.91	0	8.91	0	8.4832	6.0626	3.5028	12.0161	9.7967	8.6182	9.3343	5.2326	0
GSM65793	1	1	0	0	1	0	2	1.7	45				3.67	0	3.67	0	10.1223	8.9947	6.3447	12.0369	9.7676	10.4640	8.8059	4.6403	0
GSM65794	1	1	1	0	0	0	2	1.1	56				8.66	0	8.66	0	12.2629	6.3219	3.8988	12.6617	9.5371	10.7220	8.7220	5.2485	0
GSM65795	1	1	0	0	0	0	3	1.9	50				8.75	0	8.75	0	9.3401	5.9556	3.6248	12.3133	10.4622	10.1628	9.0379	4.9313	0
GSM65796	1	1	0	0	0	0	2	0.9	45				1.00	1	1.00	0	9.7442	9.1605	4.7690	12.3542	9.8369	9.6447	8.8754	4.7569	0
GSM65797	1	1	0	0	0	0	1	1.1	55				2.33	0	2.33	0	9.9788	9.3928	4.3239	12.2864	10.0096	9.8958	8.4278	4.8762	0
GSM65798	1	1	0	1	1	0	1	1	48				9.41	0	9.41	0	10.0258	9.9231	5.5797	12.2894	9.4855	10.0400	8.7925	4.8825	0
GSM65799	1	1	0	1	0	0	2	1.8	62				7.33	0	7.33	0	9.8296	10.6055	4.7432	11.9949	9.8574	9.8016	8.9446	4.8787	0
GSM65800	1	1	0	1	1	0	1	2	56				6.25	0	6.25	0	9.7768	11.1382	6.0956	12.3128	9.7894	9.4112	8.7293	4.8893	0
GSM65801	1	1	0	1	1	0	2	2	51				8.99	0	8.99	0	9.2268	10.1614	6.0176	12.2130	10.2170	9.9126	9.0967	4.8438	0
GSM65802	0	1	0	0	0	0	3	2.3	37				6.75	0	6.75	0	8.5164	6.2312	3.8828	12.7445	9.8047	10.0117	9.3267	4.6893	0
GSM65803	0	1	0	1	1	0	3	1.5	54				2.58	1	2.58	1	9.8035	9.6242	4.8600	12.3659	9.9554	10.5211	8.6291	4.8116	0
GSM65804	0	1	0	0	0	0	2	2.1	38				2.25	1	2.25	1	9.3799	7.1021	4.4663	12.2255	9.6929	10.0121	8.6397	4.9898	0
GSM65805	1	1	0	0	0	0	1	1.2	44				8.41	0	8.41	0	9.0627	5.4761	4.0521	11.7626	9.5158	9.7626	8.9351	4.7588	0
GSM65806	0	0	0	0	0	0	3	3	38				0.17	1	0.17	0	8.1669	5.7929	3.8381	12.5669	9.2061	8.7639	8.5522	4.7012	0
GSM65807	1	1	0	0	0	0	1	1.3	51				9.24	0	9.24	0	9.1397	8.1028	3.1748	12.2834	9.7132	8.7849	8.7798	4.7996	0
GSM65808	1	1	0	0	0	0	2	2.2	46				8.25	0	8.25	0	10.2675	9.4063	4.4885	12.3420	9.6411	9.2002	8.3660	4.8397	0
GSM65810			0	1	1	0	1	2.6	59				8.83	0	8.83	0	9.4022	9.8424	5.8601	11.9917	9.3841	9.5823	8.8062	4.8772	0
GSM65811	0	1	0	0	1	0	2	1.2	46				0.58	0	0.58	0	10.1064	9.3881	5.3072	12.1629	10.0584	9.5277	9.0109	5.2483	0
GSM65812	1	1	0	1	1	0	1	2.6	53				11.91	0	11.91	0	9.9391	10.0812	6.4218	12.0873	10.0085	8.5064	8.8992	4.6309	0
GSM65813	1	1	1	0	0	0	2	2.4	37				0.75	1	0.75	0	12.8528	8.5030	4.0057	12.3143	9.5785	8.7637	9.0386	4.8944	0
GSM65814	1	1	0	0	0	0	1	1.8	49				3.50	0	3.50	0	9.9867	7.4914	4.3829	12.5947	10.3688	10.0030	8.8776	5.2510	0
GSM65815	1	1	0	1	0	0	1	2.2	56				5.91	1	5.91	1	9.8916	9.9785	4.7716	12.0563	10.0580	9.3698	8.1884	4.9974	0
GSM65816	0	0	0	0	0	0	2	2.4	61				9.99	0	9.99	0	9.6270	6.7399	3.7026	12.2441	10.6258	9.1270	9.1844	5.1999	0
GSM65817	1	1	0	0	0	0	2	1.2	36				10.16	0	10.16	0	9.2477	9.3617	3.9237	12.6177	10.3775	8.6806	9.0313	4.7112	0
GSM65818	1	1	0	1	0	0	2	2.4	63				4.25	1	4.25	0	10.0367	10.8996	4.0420	12.5865	9.9836	8.9027	9.0330	4.8209	0
GSM65819	1	1	0	1	0	0	3	2.3	60				11.41	0	11.41	0	9.1191	10.4145	4.0326	12.7792	9.6322	8.9069	9.1616	4.7505	0
GSM65820	1		1	1	1	0		0	44				14.53	0	14.53	0	11.3106	10.7967	5.0464	12.5241	10.0062	9.6009	9.1563	5.2409	0
GSM65821	1		0	1	1	0	2	2.6	46				11.18	0	11.18	0	10.5578	9.7499	6.4148	12.4077	9.6455	9.2960	9.1480	5.3111	0
GSM65822	0		0	0	1	0	2	1.8	38				8.48	1	8.48	0	9.0275	9.5062	5.9330	12.3930	9.8958	8.8521	8.7826	5.0200	0
GSM65823	1		0	1	1	0		0.3	61				14.19	0	14.19	0	10.1397	10.5387	5.1806	12.1995	10.1011	10.2314	8.9605	6.1870	1
GSM65824	0		1	0	0	0	2	2	43				12.05	1	12.05	1	12.8195	7.2070	3.5408	12.4964	10.2803	9.4703	8.8866	5.4824	0
GSM65825			1	0	0	0	3	2.6	65				13.96	0	13.96	0	13.6391	5.7108	3.8707	12.5357	9.5519	8.7377	9.2460	5.0992	0
GSM65826	1		0	0	0	0		1.4	61				5.82	1	5.82	1	10.2266	9.4880	3.8253	12.2111	9.5942	8.3137	8.8027	5.5946	1
GSM65827	1		0	0	1	0		0.9	58				13.78	0	13.78	0	10.3538	8.9495	5.2931	12.6450	10.2939	8.5468	8.7158	5.4386	0
GSM65828	1		0	1	0	0		3.1	60				1.78	1	1.78	1	10.3450	11.2827	4.5173	13.1210	10.5445	8.9873	9.1876	5.1298	1
GSM65829	0		0	0	1	0		1	45				11.41	1	11.41	0	10.6226	9.3940	7.4257	12.0811	9.6760	9.0236	8.8598	4.7003	0
GSM65830	1		0	0	0	0	1	1.9	42				13.34	1	13.34	0	10.1963	8.1992	4.4476	12.7511	10.2838	8.9228	8.6448	5.2020	0
GSM65831	1		0	1	0	0	3	3	55				13.78	0	13.78	0	10.2156	11.0757	4.0110	11.1861	10.6347	7.8631	8.9080	5.8831	1
GSM65832	0		0	1	1	0	2	2.5	45				12.92	0	12.92	0	10.7489	10.4593	5.5724	12.2129	9.9076	8.3374	9.3249	5.0086	0
GSM65833	0		1	0	0	0	3	2	42				13.44	1	13.44	0	12.6293	9.3704	3.6745	12.0942	9.8419	8.3259	8.7894	5.0266	0
GSM65834	1		0	1	0	0	2	1.5	64				12.55	1	12.55	1	10.0864	11.3166	3.9263	11.6509	9.8840	9.3304	9.0121	6.0940	1
GSM65835	0		1	0	0	0	2	1	53				1.54	1	1.54	1	13.7075	5.8731	3.2896	12.0388	9.9466	8.6958	9.0935	5.0062	0
GSM65836	0		0	0	0	0	2	1	47				12.62	0	12.62	0	10.8045	7.5468	3.5230	12.5467	10.1338	9.6698	9.1064	5.3650	0
GSM65837	1		0	1	0	0	3	2.1	64				5.14	1	5.14	1	10.5578	10.0648	4.7730	12.4952	10.0101	7.9425	9.3079	5.0290	0
GSM65838			1	0	0	0		1	54				13.31	0	13.31	0	12.7053	6.3279	4.1117	12.5016	9.8255	9.3446	8.5538	5.0421	0
GSM65839	0		0	0	0	0	3	4.5	73				0.69	1	1.54	0	10.8097	5.2146	3.4583	12.5803	9.4279	9.0947	8.5475	5.4712	0
GSM65840	1		0	1	1	0		2	48				12.64	1	12.64	0	10.4740	9.8654	4.9441	12.4779	9.7543	8.4565	9.0416	5.2456	0
GSM65841	0		0	0	0	0	2	1.6	64				10.56	1	10.56	0	9.9520	6.3433	3.4748	12.5010	9.9960	9.4228	8.7949	5.4305	0
GSM65842	0		0	0	0	0	3	2.2	47				12.83	0	12.83	0	8.7163	5.8697	3.3997	12.0309	10.0837	9.4651	9.1224	5.3497	0
GSM65843	0		0	0	0	0	3	5	39				7.02	1	7.02	0	9.0068	6.3845	3.6319	12.6525	10.4429	9.4033	9.2681	5.8424	1
GSM65844	1		0	1	0	0	2	4.5	65				0.61	1	0.61	1	9.4971	10.3407	4.4315	12.3904	10.0541	9.0220	9.0479	5.7291	1
GSM65845	0		0	0	0	0	3	3	32				12.60	0	12.60	0	9.0636	5.8145	3.5902	12.8882	10.4641	10.4824	8.3506	5.3902	1
GSM65846	0		1	0	0	0	3	2.5	57				2.84	1	3.05	1	13.5473	7.3343	3.7186	12.4284	9.8402	9.0214	8.9080	5.3798	0
GSM65847	1		0	0	1	0		1.8	64				2.92	1	2.92	1	10.8064	9.4804	5.2473	11.6286	9.8268	9.0711	8.8137	5.4159	0
GSM65848	1		0	0	0	0	3	3	60				12.79	0	12.79	0	10.3851	8.8580	4.1818	12.1865	9.9029	9.6213	8.6264	5.2814	0
GSM65849	1		0	0	0	0		2	71				2.89	1	2.89	1	10.0976	8.6755	3.4329	12.2380	9.5292	9.2582	8.8795	4.8319	0
GSM65850	0		1	0	0	0	3	0.7	42				10.45	0	12.42	0	13.1996	6.5851	4.0253	12.2699	10.0095	9.2610	9.2733	5.0207	0
GSM65851	1		1	1	0	0		3	57				12.47	0	12.47	0	11.5767	10.2803	3.1300	12.8496	10.3228	9.4221	9.2525	4.9516	1
GSM65852			0	0	0	0		2.5	53				3.47	1	3.47	1	8.8717	4.9666	3.8041	12.6610	10.9373	9.7757	9.4970	5.1275	0
GSM65853	1		0	1	1	0	2	1.3	64				10.72	0	10.72	0	10.1993	10.4358	5.0420	11.8574	9.5406	9.1852	8.3966	5.2425	0
GSM65854	1		0	1	1	0		2	66				12.27	0	12.27	0	9.8024	10.4351	4.9729	12.8941	10.6447	9.5729	9.3686	5.5028	1
GSM65855	0		0	0	0	0	2	3.8	47				3.11	1	3.11	0	10.7088	7.3652	3.3977	12.4805	10.1957	9.3392	9.0004	5.0588	0
GSM65856	1		0	1	0	0		2.4	63				9.82	0	9.82	0	10.2030	10.0360	3.3035	12.2277	10.6348	9.4824	8.8240	6.2490	1
GSM65857	1		0	1	1	0	2	1.8	54				2.73	1	4.74	1	10.1689	11.0580	6.4253	12.4132	10.0935	8.8552	9.0552	5.2261	0
GSM65858			0	0	1	0	2	1.6	42				12.04	0	12.04	0	10.3096	9.0226	5.9804	12.3367	10.0993	9.7901	8.7247	5.0451	0
GSM65859	1		0	1	0	0	3	2.6	53				12.32	0	12.32	0	10.0631	10.6903	3.5207	12.3880	9.9777	9.4551	9.0172	5.0387	0
GSM65860	1		0	1	0	0	3	1.5	59				12.25	0	12.25	0	10.1916	9.9320	3.1472	11.7643	9.7152	8.5851	9.3339	5.0081	0
GSM65861	0		0	0	0	0	2	1.9	71				2.64	1	2.64	1	9.3977	6.6937	3.6304	12.4021	9.6992	8.7356	8.2715	5.5687	0
GSM65862	1		0	0	1	0	2	1.8	54				11.86	0	11.86	0	11.0932	9.1609	5.3846	12.1802	10.4480	9.0786	9.0214	5.2528	0
GSM65863	0		1	0	0	0	3	4	45				9.26	0	11.93	0	11.9817	4.4364	3.3306	12.5078	10.2303	9.6240	9.1647	5.5837	1
GSM65864	1		1	1	0	0		1	64				10.04	1	10.04	0	11.2336	9.5977	3.4475	12.4329	10.4830	9.4095	9.0887	5.2673	0
GSM65865	0		0	0	0	0	3	3	43				12.06	0	12.06	0	8.8992	6.9956	3.4207	12.2046	9.9870	9.1172	8.9326	5.0911	0
GSM65866	0		1	0	0	0	3	1.3	68				11.17	0	11.17	0	13.5560	6.3019	3.7086	12.4989	9.4425	8.4561	8.6312	4.7192	0
GSM65867	1		0	1	0	0	3	2	63				11.56	0	11.56	0	9.7955	10.2878	3.4644	12.1411	9.2464	8.3693	8.6279	5.1090	0
GSM65868	1		0	1	0	0	2	2	69				4.08	1	3.01	1	10.5560	10.7479	3.5165	12.8395	10.3531	8.5228	9.3528	5.5016	1
GSM65869	1		0	1	0	0	2	3	62				2.63	1	2.63	1	10.4336	11.6882	3.6996	12.5600	10.4014	8.8391	8.5981	5.1231	0
GSM65870	0		1	0	0	0	3	2.2	43				5.71	1	11.46	0	13.3295	7.9190	3.7111	12.6469	10.1470	8.7679	8.7115	5.4853	0
GSM65871	1		0	1	0	0	1	1	48				8.74	0	8.74	0	10.0206	9.7430	4.1216	12.5888	10.3313	9.5288	8.8379	5.3326	0
GSM65872	0		0	0	0	0	2	2	43				2.00	1	2.00	1	9.7452	7.9871	4.3946	12.3692	10.0247	9.3146	8.8953	4.9101	0
GSM65873	1		0	0	0	0	1	0.8	50				4.36	0	4.36	0	10.2946	9.2390	4.7602	12.5826	9.6775	9.5602	10.0118	4.9510	0
GSM65874	1		0	0	0	0	1	0.8	43				5.26	0	5.26	0	10.5513	6.4896	3.6576	12.4757	10.5514	9.3952	8.8939	5.3486	0
GSM65875	1		0	1	1	0	1	2	70				8.94	0	8.94	0	10.6093	10.9848	5.8873	11.9596	10.5137	8.9726	8.3335	5.2415	0
GSM65876			0	0	0	0	1	2	52				5.24	0	5.24	0	7.4489	6.5065	3.5056	11.6565	8.4818	8.1369	8.3137	5.0249	0
GSM65877	1		0	0	1	0		1.3	49				7.25	0	7.25	0	10.7939	9.3378	5.8300	12.2282	9.5255	8.9903	9.2292	4.9611	0
GSM65878	0		0	0	0	0	2	2	38				0.73	1	0.73	1	9.5143	4.4338	3.3892	12.4757	9.8855	8.7109	9.6575	4.8183	0
GSM65879	0		0	0	0	0		0.9	47				3.53	0	3.53	0	10.2209	6.0789	3.4703	11.9177	10.6031	8.5436	8.9547	5.3564	0
GSM65880	0		0	0	1	0	2	1	39				6.29	1	7.70	0	10.9682	9.1704	5.5328	12.1967	9.7944	9.4426	9.0093	5.4692	0
GSM79114	1	0	0	1	0	0	1	9	68	0	11.83	0					9.9066	11.0156	4.0407	12.0467	9.5555	8.4293	8.6711	5.0784	0
GSM79115	0	0	0	0	0	0	3	12	40	0	11.83	0					9.2895	5.8298	4.1866	12.7602	9.1473	9.3361	8.5117	5.2996	0
GSM79116	1	1	1	0	1	0	3	26	51	0	11.83	0					13.2218	8.8497	4.9149	12.2709	9.5550	8.7495	8.8967	5.3491	0
GSM79117	0	0	0	0	0	1	3	22	52								8.6902	6.5629	4.2871	12.3872	10.9595	10.0627	8.1856	5.0860	1
GSM79118	1	1	0	1	1		3	24	80	0	3.58	0					8.7206	12.5850	4.8837	12.1328	10.1736	9.1349	8.8720	5.2906	0
GSM79119	1	1	0	1	0	0	1	13	46	0	11.75	0					10.1094	10.6605	4.8099	12.5493	9.9924	8.4400	9.0799	5.1380	0
GSM79120	1	1	0	1	0	0	1	60	37	1	6.92	1					9.9772	9.9079	4.7581	12.7756	10.2689	9.5049	8.6198	5.2669	0
GSM79121	1	1	0	1	1	0	1	50	70	0	11.33	0					9.7302	12.1208	5.7302	12.3278	10.4239	9.7188	9.1245	5.2107	0
GSM79122	1	1	0	0	0	0	2	20	74	0	11.67	0					10.5764	6.4411	4.3222	12.4632	10.1407	8.7128	9.1346	5.5911	1
GSM79123	1	1	0	1	0	0	3	33	41	1	7.17	1					9.7557	11.1841	4.2332	13.0173	10.0598	8.6568	8.6317	5.2043	1
GSM79124	1	1	0	1	0	1	2	24	61	1	1.50	1					9.2561	11.6612	4.6236	12.1720	10.4320	9.7132	8.8684	5.4012	0
GSM79125	1	1	1	1	0	0	3	32	38	0	5.50	0					12.3227	10.3881	4.5229	13.2135	10.5562	9.4210	9.2794	5.3894	1
GSM79126	1	1	0	1	1	1	1	12	67	0	11.17	0					10.2315	11.8884	5.5289	12.4730	9.5271	9.0179	8.7910	5.0894	0
GSM79127	0	0	0	1	1	0	2	18	69	0	7.42	0					10.5174	10.8123	4.9838	12.3147	10.2170	9.5131	8.9733	5.5727	1
GSM79128	1	1	1	0	0	0	2	16	34	0	11.58	0					12.8326	8.6930	4.0438	12.2765	10.4317	9.6111	8.7145	5.7091	1
GSM79129	1	1	0	1	0	0	2	19	79	0	11.17	0					9.1154	11.9224	4.5516	13.0845	11.1694	10.3202	8.9666	5.1960	1

GSM79130	1	1	0	1	1	0	2	21	46	1	9.00	1	9.8802	10.7452	4.9166	12.1252	9.7405	9.6795	8.6118	5.2349	0
GSM79131	1	1	1	1	1	0	3	22	57	1	4.67	1	11.9253	10.8980	5.4882	12.5197	10.1766	9.5138	9.0996	5.6068	1
GSM79132		0	0	0	1		1	12	71	0	4.58	0	9.9215	9.4961	5.3471	12.4233	10.3184	9.4579	9.0400	5.3801	0
GSM79133	1	1	0	1	1	1	2	18	73	1	5.58	1	10.3861	10.9406	6.1327	12.3886	10.2692	10.4084	8.9424	5.4019	0
GSM79134	1	1	0	1	0	1	3	26	59	1	5.83	1	9.5710	12.1508	4.5875	12.3364	11.9628	8.9916	9.3013	6.1823	1
GSM79135	1	1	0	1	1	0	2	28	63	1	8.25	1	9.1496	11.8807	5.0701	12.3329	10.6944	10.5544	8.4125	5.4276	0
GSM79136	1	1	0	1	1	0	1	26	64	0	11.50	0	9.7712	10.7822	7.9173	12.3978	10.1387	9.6021	9.2844	5.5898	1
GSM79137	1	1	1	1	0	0	2	17	54	0	11.50	0	12.8549	10.2994	4.1772	13.0252	10.5995	9.3957	8.9096	5.2013	1
GSM79138	0	0	1	0	0	0	2	6	68	0	11.50	0	12.8333	7.7713	4.2799	12.3751	10.1374	9.0227	8.6114	5.2949	0
GSM79139	1	1	0	1	0	1	1	23	65	0	11.50	0	10.0954	11.3591	4.1919	12.3892	10.1886	8.6642	9.0640	4.9428	0
GSM79140	1	0	0	1	0	0	3	28	84	1	0.33	1	9.6495	10.9142	3.6731	12.7572	11.1160	8.5586	9.4426	5.7365	1
GSM79141	1	1	0	1	1	0	2	25	67	0	10.83	0	9.6509	9.7107	6.2587	12.1436	10.4108	9.8519	9.8506	5.3575	0
GSM79142	1	1	0	1	0	0	2	12	61				9.9009	10.4466	4.6075	12.2735	10.3462	9.2070	9.0421	5.1195	0
GSM79143	0	0	1	0	0	0	3	27	67	0	11.50	0	12.6690	7.2765	4.5828	12.7643	9.6936	9.4859	8.5665	5.3428	0
GSM79144	0	0	1	0	0		3	29	93	0	2.17	0	12.0175	6.0009	4.0885	12.1189	9.5525	9.6665	8.4695	5.3691	0
GSM79145	1	1	0	0	0	0	2	21	40	0	11.50	0	9.0468	8.7673	4.1739	12.3849	10.3693	8.6351	8.5695	5.5616	0
GSM79146	1	1	0	1	0	0	2	18	73	0	11.42	0	9.2998	11.2581	4.0956	12.1213	10.5058	8.9511	8.7635	5.6571	1
GSM79147	0	0	0	0	0	1	3	50	34				8.4265	8.5772	4.3922	12.6772	10.5733	9.6623	8.6694	5.4741	0
GSM79148	0	0	1	0	0	1	3	24	71	0	5.08	0	12.8124	6.5418	4.3759	12.7279	10.0565	10.3632	8.9928	5.1673	0
GSM79149	1	1	0	1	0	1	?	26	71	1	2.92	1	9.8038	10.6443	4.8068	12.2708	10.4059	9.3833	8.8522	5.1681	0
GSM79150	1	1	0	1	0	1	2	20	61	0	10.83	0	9.4179	10.6272	4.1635	12.3724	10.4810	9.3365	9.0633	5.3977	0
GSM79151	1	1	0	1	1	1	2	35	55	0	11.42	0	9.7870	11.9018	5.2414	12.7334	9.4861	9.2106	8.6540	5.1858	0
GSM79152	1	1	0	1	1	0	2	15	57	0	11.42	0	10.1169	11.7811	5.7085	12.7116	10.0984	9.0577	9.0123	4.9914	0
GSM79153	1	1	0	1	0	0	2	18	83	0	2.08	0	9.0763	11.3450	3.8798	12.2513	10.5414	9.3206	8.9759	5.5342	0
GSM79154	0	0	0	0	0	0	3	18	71	0	11.42	0	8.9319	6.7244	4.3872	12.5035	10.6344	9.2324	8.9298	5.2311	0
GSM79155	1	1	0	1	0	1	2	9	52	1	4.58	1	10.6211	12.6041	4.6578	12.8717	10.3410	9.0849	9.0623	5.6939	1
GSM79156	0	0	0	0	0	0	1	13	57	0	11.42	0	8.8706	9.4007	4.2416	12.4039	10.3470	9.7320	8.8321	5.0484	0
GSM79157	1	1	0	1	0	0	1	12	70	0	11.42	0	9.9737	10.1213	4.4541	12.3835	10.1474	8.9166	8.5221	5.1896	0
GSM79158	1	1	0	1	1	0	2	7	69	0	8.50	0	9.2396	11.0501	4.8598	12.5268	10.1606	8.3440	9.0970	4.9195	0
GSM79159	1	1	0	1	1	1	2	14	57	0	11.42	0	9.7944	11.6069	5.1237	12.2476	10.0089	8.7061	9.2091	5.5356	0
GSM79160	1	1	0	0	1	0	2	50	83	0	4.42	0	9.5963	9.4959	5.1463	11.9556	10.0895	9.0314	9.2501	5.3211	0
GSM79161	1	1	0	1	0	0	1	14	73	0	5.50	0	9.5952	11.0220	4.5576	12.2237	9.7610	8.5164	8.9009	5.3240	0
GSM79162	1	1	0	1	1	0	2	13	69	0	11.42	0	9.9469	10.4678	6.1705	12.5536	10.3870	8.6215	9.3687	5.1708	0
GSM79163	1	0	0	1	1	1	3	33	73	0	10.75	0	9.3534	11.9627	5.3106	12.4405	10.6923	9.1514	9.7546	5.4783	0
GSM79164	1	1	0	1	1	0	1	8	49	0	11.33	0	9.6769	10.2329	5.6744	12.1843	10.2328	9.3398	8.8098	5.3421	0
GSM79165	0	0	0	0	0	0	2	23	62	0	11.33	0	9.5817	6.5394	4.0501	12.0483	10.3334	9.3360	8.7986	5.7885	1
GSM79166	1	1	0	1	1	1	2	23	74	1	2.33	1	8.2292	11.7123	5.3274	12.4780	10.3636	9.5209	9.1427	5.5355	0
GSM79167	1	1	0	1	1	1	2	22	65	0	11.33	0	10.1758	12.4463	5.3195	12.2398	9.6451	9.5112	8.7383	5.3634	0
GSM79168	1	1	0	1	1	0	2	45	90	0	5.33	0	9.3768	11.7485	6.3502	12.1911	10.0219	10.0452	8.5012	5.3743	0
GSM79169	1	1	0	1	0	0	2	14.1	57	0	11.33	0	9.0099	10.2472	4.3926	12.4753	10.0811	8.9843	8.8771	5.0070	0
GSM79170	1	1	0	1	1	0	2	15	80	1	4.33	1	9.4453	10.3390	4.8250	12.2602	10.2066	9.7567	8.7188	5.4675	0
GSM79171	1	1	0	1	1	1	2	25	70	1	4.08	1	9.1840	10.6188	4.8731	12.4379	11.1165	8.9512	8.6761	5.3551	1
GSM79172	1	1	0	1	1	0	2	18	62	1	7.00	1	9.8459	12.1896	5.3995	12.3379	10.9964	9.2161	9.1892	5.2456	1
GSM79173	1	1	1	1	1	0	3	23	42	0	11.25	0	11.1735	10.4797	5.6661	12.4944	10.0245	9.4787	8.9265	5.2721	0
GSM79174	1	1	0	1	1	1	1	16	72	0	11.25	0	10.0786	11.7043	6.7375	12.2961	10.1720	8.7262	8.8585	5.0539	0
GSM79175	1	1	0	1	0	0	1	11	74	0	5.75	0	9.1963	10.6469	4.3988	12.3237	10.0600	9.3224	9.4292	4.7242	0
GSM79176	1	1	0	1	1	1	2	60	46	1	0.42	1	9.5682	10.4407	4.8899	12.6462	10.0142	9.3063	8.7388	5.5648	0
GSM79177	1	1	0	1	0	0	2	25	77	0	6.00	0	9.8173	10.5907	3.7646	12.3994	10.3136	8.9435	9.4897	5.6017	1
GSM79178	1	1	0	0	1	0	1	18	41	0	11.17	0	9.6710	9.0423	4.9756	12.4728	10.2687	9.1622	8.8134	5.5156	0
GSM79179	1	1	0	1	0	0	2	16	82	0	7.42	0	9.9751	10.6376	4.1213	12.8072	10.6067	9.6619	9.1176	5.0108	0
GSM79180	1	1	1	1	0	0	2	8	66	1	4.00	1	12.2955	10.4629	4.0657	12.4700	9.6702	9.2511	8.4797	5.3652	0
GSM79181	1	1	1	0	0		2	25	83	0	1.92	0	13.0082	9.4741	4.5513	12.4072	10.6935	9.2797	8.9335	5.3415	0
GSM79182	1	1	0	0	1	0	1	2	70	0	7.25	0	9.5811	9.3937	4.8329	12.4172	10.7077	9.7487	8.9569	5.4418	0
GSM79183	1	1	0	0	0	1	1	20	34	0	11.08	0	9.6921	8.2962	4.7635	12.4997	10.6430	9.5273	9.3766	5.4720	0
GSM79184	0	0	0	0	0	1	3	28	51	0	11.08	0	9.8916	8.7994	4.6644	12.7925	9.8714	9.4287	9.5763	5.2169	0
GSM79185	1	1	0	1	0	1	2	24	60	1	10.83	1	10.9833	12.0897	4.4328	12.7844	11.1891	9.8244	9.1907	5.1340	1
GSM79186	1	1	0	1	0	0	1	10	63	0	11.00	0	9.8080	10.5086	4.7399	12.1720	9.2172	9.7257	8.9381	5.1891	0
GSM79187	1	1	0	1	0	0	1	8	68				9.2471	10.2296	4.7644	12.4552	10.0299	9.7922	9.1439	5.2879	0
GSM79188	1	1	0	1	1	1	2	46	86	1	1.42	1	9.5278	12.2223	5.4221	12.2715	9.9614	9.4457	8.5677	5.0941	0
GSM79189	1	1	1	1	1	1	2	23	29	1	0.00	1	12.9581	11.9869	5.6473	12.5366	10.9293	9.1275	8.7299	5.2820	0
GSM79190	0	0	1	0	0	0	2	14	60	0	11.00	0	12.3647	7.4042	4.0077	12.0758	9.9766	9.2523	8.6245	5.2426	0
GSM79191	0	0	0	0	0	0	2	38	58				9.6243	6.6825	4.5444	12.6418	10.6082	9.2365	9.4202	5.3279	0
GSM79192	1	1	0	1	1	0	2	19	68	0	10.75	0	9.6663	11.9211	6.5289	12.0533	10.3284	9.2857	9.1499	4.9287	0
GSM79193	1	1	1	1	0	0	2	27	59	0	11.00	0	12.1331	11.8716	3.9189	12.8372	10.3902	8.6126	9.0578	5.7081	1
GSM79194	1	1	0	1	0	0	1	8	73	0	7.92	0	9.1744	11.2664	4.2198	12.5383	8.9671	8.6961	8.6275	5.3543	0
GSM79195	0	0	1	0	0	1	2	16	68	0	10.92	0	11.3000	6.8927	3.9717	12.2626	9.8913	8.4405	8.8019	5.2798	0
GSM79196	1	0	0	0	0	1	3	22	77	0	3.58	0	9.5715	8.7126	4.5066	12.5204	9.5748	9.4226	8.5831	5.3013	0
GSM79197	1	1	0	1	0	0	1	18	87	0	3.00	0	9.4802	11.3314	4.4029	12.5679	10.3174	9.3576	8.9689	5.1433	0
GSM79198	1	1	0	1	0	0	1	14	65	0	10.92	0	10.4940	11.6892	4.3540	12.1334	11.1060	8.7250	9.3018	5.4404	1
GSM79199	1	1	0	1	0	0	2	16	44	0	10.92	0	8.0718	10.9557	4.7631	13.0625	11.5825	9.3459	9.5979	5.6972	1
GSM79200	1	1	0	0	1	0	2	20	74	0	10.92	0	9.4688	9.0523	5.7924	12.4580	10.4129	9.5037	9.3887	5.4533	0
GSM79201	1	1	0	1	1	0	2	23	54	0	10.92	0	8.8502	9.8447	5.7297	12.6157	10.1497	9.3259	9.0243	5.0648	0
GSM79202	1	1	0	0	1	1	1	31	61	1	4.17	1	9.3465	8.6878	4.8496	12.3742	9.4314	8.4315	8.5531	5.0376	0
GSM79203	1	1	0	1	1	1	3	20	47	0	10.92	0	9.8800	9.9864	7.5940	12.5353	10.1754	10.0656	8.9585	5.3096	0
GSM79204	1	1	0	1	0	1	2	24	53	0	10.92	0	10.6514	10.8459	3.6067	12.5885	9.8439	9.4336	8.8289	5.4858	0
GSM79205	1	1	0	1	0	0	2	10	72	0	10.83	0	9.6534	10.2606	4.2369	12.4079	10.4145	8.8441	9.0907	4.9894	0
GSM79206	1	1	0	1	0	1	2	40	51	0	10.83	0	9.9274	10.2045	4.0109	12.3493	10.5203	8.4645	8.8631	5.1800	0
GSM79207	1	1	0	1	1	0	1	12	70	0	10.58	0	9.6384	10.1101	5.3175	12.4285	10.2075	8.9552	8.7943	5.3007	0
GSM79208	1	1	0	1	1	0	1	22	59	1	4.42	1	9.6553	11.2233	7.3226	12.0659	10.3335	8.8591	9.8206	5.0983	0
GSM79209	1	1	0	1	1	0	2	18	80	0	6.42	0	9.3122	11.5635	6.3184	12.7930	10.3120	10.3206	9.0550	5.4825	0
GSM79210	1	1	0	1	1	1	1	10	59	0	10.83	0	9.8660	11.4640	5.9625	12.6802	10.4042	8.4940	9.3347	5.2680	0
GSM79211	1	1	0	1	1		2	22	67	0	0.08	0	9.4207	11.8980	5.7188	12.7933	10.3728	8.1681	8.9630	5.3810	0
GSM79212	1	1	0	1	1	0	1	14	80	0	10.83	0	10.3027	11.4358	8.1097	12.3513	10.2442	9.3358	9.2432	5.2478	0
GSM79213	1	1	0	1	1	0	2	42	45	0	10.83	0	10.7458	10.6859	6.1645	12.6908	10.1787	9.4555	9.4841	5.2311	0
GSM79214	1	1	0	1	1		2	18	85	0	1.50	0	9.8864	11.8417	6.7309	12.4515	10.5956	9.6074	9.1044	5.5922	1
GSM79215		0	0	1	1	0	2	24.1	74	0	10.83	0	9.7236	11.1990	6.1190	12.2425	10.5356	9.8808	9.2577	5.4394	0
GSM79216	1	1	0	1	1	0	2	18	70	0	10.08	0	9.2837	12.4654	5.0812	12.0015	9.4412	7.7571	8.5570	5.1808	0
GSM79217	1	1	0	1	0	1	2	22	71	0	10.75	0	8.8323	9.7570	4.8049	12.5270	10.2411	9.5163	9.0492	5.3448	0
GSM79218	1	1	0	1	1	0	2	19	74	0	10.75	0	10.2409	10.8401	5.3901	12.3118	10.2696	9.0807	8.7091	5.5321	0
GSM79219	1	1	0	1	1	0	2	14	69	0	10.75	0	10.1144	11.4263	6.7062	12.3296	10.3407	9.3257	8.8855	5.5358	0
GSM79220	1	1	0	1	0	0	1	10	68	0	10.67	0	9.0414	11.1325	4.3100	12.2111	9.9258	10.3843	8.8236	5.1756	0
GSM79221	1	1	0	1	0	0	1	20	42	0	10.75	0	9.4135	10.5713	4.6722	12.2909	9.9997	8.7258	8.5679	5.0559	0
GSM79222	1	1	0	1	1	0	2	17	68	0	10.67	0	8.8251	11.5346	6.0336	12.3722	9.7592	9.7388	9.3151	5.4463	0
GSM79223	1	1	0	1	1	1	3	65	50	1	1.17	1	9.9574	11.1812	5.2044	13.4723	11.8299	9.8998	9.2926	5.6409	1
GSM79224	1	1	0	0	0	1	2	32	44				10.8617	9.1481	4.0607	12.5788	10.6323	9.1722	8.6978	5.3133	0
GSM79225	0	0	0	0	0	0	1	11	70	0	7.92	0	9.2327	6.1599	3.9526	12.3734	10.1323	9.6128	8.9387	5.2943	0
GSM79226	1	1	0	1	0	1	2	27	72	0	10.75	0	9.3154	11.1602	4.5583	12.3569	10.8403	9.3464	9.7354	5.8225	1
GSM79227	1	1	0	0	1	1	3	45	28	0	10.75	0	9.4262	8.0504	4.8569	12.2718	10.5109	9.3634	9.3076	5.0650	0
GSM79228	1	0	0	1	0	0	1	12	57				10.1791	11.0551	3.8483	12.3556	10.3275	8.3735	9.0153	5.0215	0
GSM79229	1	1	0	1	0	0	1	13	52	0	10.67	0	10.1974	10.2571	3.6872	11.9544	10.4082	8.3048	8.6638	5.4006	0
GSM79230	1	1	0	1	0	0	2	12	83	0	4.83	0	9.9407	11.4535	4.1780	12.7299	11.0290	8.8144	9.5543	5.7195	1
GSM79231	1	1	0	0	0	1	2	22	77	1	0.58	1	10.2456	7.8829	3.7156	12.4567	10.3677	9.1543	9.2408	4.9131	0
GSM79232	1	1	0	1	0	0	1	22	56	1	10.00	1	10.0297	10.8773	3.8848	12.2139	10.1702	9.6567	8.4797	5.5407	0
GSM79233	1	1	1	1	1	1	3	24	44	1	2.92	1	11.7554	10.2996	5.2291	12.3981	10.2273	9.2133	8.4101	5.5108	0
GSM79234	1	1	0	1	1	0	1	11	49	0	10.67	0	9.3101	9.9160	6.0901	12.3945	9.4450	9.2080	8.7588	5.2628	0

GSM79235	1	0	0	1	0	1	3	11	29	0	10.67	0	8.9330	10.2290	4.6022	12.4620	10.2921	9.9644	9.6571	5.1251	0
GSM79236		0	0	1	1	0	1	21	71	0	10.67	0	9.7903	12.5088	4.9043	12.6879	10.8379	9.2284	9.0034	5.2262	0
GSM79237	1	1	0	1	0	0	1	18	72	0	10.67	0	9.8317	10.8577	3.9298	12.6701	10.6527	10.0716	8.8062	5.4260	0
GSM79238	1	1	1	1	1	1	2	19	50	0	10.67	0	12.2831	10.3871	5.7227	12.4247	10.2067	8.9019	8.9527	5.5276	0
GSM79239	0	0	1	0	0	0	3	24	54	0	10.67	0	12.7822	6.8386	4.1362	11.9977	9.7021	9.1067	8.8525	5.0681	0
GSM79240	1	1	0	1	0	1	2	17	65	0	10.58	0	10.3072	10.3023	3.7974	12.7498	11.8151	8.4012	9.0237	5.1553	1
GSM79241	1	1	0	1	0	0	2	22	68	0	10.67	0	9.5499	11.6524	4.4648	12.5938	10.7674	10.0358	9.1884	5.0590	0
GSM79242	1	1	0	1	0	1	2	35	61	1	3.58	1	8.6493	11.2558	4.5427	12.1214	10.0697	8.7318	9.2688	5.2040	0
GSM79243	1	0	1	1	0	0	3	23	66	1	6.75	1	11.2948	12.8481	4.3500	12.0711	9.6145	8.6872	8.5907	5.3843	0
GSM79244	1	1	0	1	1	0	2	18	64	0	10.58	0	8.4761	12.2838	7.7958	12.5539	10.6005	9.2657	8.5011	5.6295	1
GSM79245	1	1	0	1	0	0	1	10	50	0	10.58	0	9.1990	9.9195	4.1533	12.3123	10.0076	9.0880	8.9424	5.3190	0
GSM79246	1	1	0	1	0	1	2	33	51	0	10.58	0	9.3623	9.8481	4.1917	12.9089	10.5337	9.8838	8.9147	5.7275	1
GSM79247	1	1	0	1	0	1	2	45	76	1	1.08	1	9.2651	10.1876	4.4704	12.4039	10.2624	9.2886	9.3443	5.1527	0
GSM79248	1	0	0	1	0	0	1	11	73	0	10.17	0	9.9507	10.8848	3.5785	12.5042	10.8416	8.4262	9.8268	5.6674	1
GSM79249	1	1	0	1	1	0	2	25	56	0	10.50	0	9.2458	10.9791	6.5049	12.3785	10.3448	8.8562	9.2381	5.7322	1
GSM79250	1	1	0	1	0	0	1	23	57	0	10.50	0	10.3688	10.2760	4.0571	12.1729	10.4562	9.3654	8.8344	5.5259	0
GSM79251	0	0	0	0	0		3	25	88	0	0.17	0	9.2068	6.2129	3.7571	12.5819	9.3380	9.7082	8.6344	5.7263	1
GSM79252	1	1	0	1	1	0	2	16	55	0	10.67	0	9.7779	9.9871	6.3100	12.3106	10.2559	10.1008	9.2434	5.0616	0
GSM79253	0	0	0	0	0	0	3	18	75	0	2.67	0	8.7212	9.1753	4.0965	12.5763	10.6126	9.3909	9.2231	5.2771	0
GSM79254	1	1	0	1	1	0	2	8	66	0	10.50	0	9.2045	11.1697	5.7091	12.4857	10.7711	9.6772	8.7187	5.4848	0
GSM79255	1	0	0	0	0	0	3	25.3	77	1	3.58	1	9.6104	8.6178	4.6224	12.5184	10.2269	8.1772	8.9882	5.1217	0
GSM79256	1	1	0	1	1	0	1	9	75	0	8.00	0	10.0384	10.6738	4.9616	12.5140	10.5947	9.2882	8.9620	5.4888	0
GSM79257	1	1	0	1	1	0	2	22	67	0	10.50	0	9.7350	10.9287	5.5029	12.6286	9.8660	9.5993	9.2670	5.1439	0
GSM79258	1	1	0	1	1	0	2	22	72	0	10.50	0	10.0021	10.5874	6.4005	12.3361	10.4764	9.0263	9.3923	5.4766	0
GSM79259	0	0	1	0	0	1	2	32	57	1	2.75	1	13.1264	7.0454	3.9814	12.2318	10.0224	9.5446	8.6373	5.3319	0
GSM79260	1	1	0	1	1	1	2	20	46	0	10.50	0	9.8504	11.0876	5.6663	12.5115	10.1020	9.1998	8.8568	5.5781	1
GSM79261	1	1	0	0	1	0	2	8	71	0	10.33	0	10.1350	9.2700	5.5614	12.2142	10.4314	9.0879	9.1729	5.2563	0
GSM79262	1	1	0	0	0	0	1	10	65	0	10.42	0	9.3808	8.6090	4.0256	12.6122	10.1630	9.4705	9.0553	5.1551	0
GSM79263	1	1	0	1	0	0	2	21	58	0	10.42	0	9.0519	11.9958	4.8179	12.0140	9.9940	8.2615	8.9593	5.1112	0
GSM79264	1	1	0	1	0	0	1	14	76	0	10.42	0	9.4665	10.6206	4.4160	12.3306	10.0079	10.8591	8.5949	5.1513	0
GSM79265	1	1	0	1	0	0	1	20	71				9.6917	11.2487	4.1036	12.4506	10.2577	9.4758	8.3893	5.1978	0
GSM79266	1	1	0	1	1	0	1	20	71	1	8.25	1	9.4818	10.7771	5.1283	12.3304	11.0883	10.6117	8.9544	5.1606	1
GSM79267	1	1	0	1	1	0	2	18	76	0	10.42	0	9.9110	12.3320	7.4158	12.4448	12.3961	10.0347	8.7688	5.3402	1
GSM79268	1	1	0	1	0	0	1	22	58	0	10.42	0	9.6674	11.1749	4.1138	12.1730	9.3965	8.4082	8.9120	5.3258	0
GSM79269	1	1	0	1	1	0	1	28	84	0	10.33	0	10.3833	12.6139	7.1911	12.5524	10.7554	9.1630	9.0638	5.4266	0
GSM79270	1	1	0	0	0	0	2	15	32	0	10.33	0	8.5301	6.5265	3.7325	12.0898	10.1195	8.4187	9.3871	5.7895	1
GSM79271	0	0	0	0	0	1	3	25	46	0	0.00	0	8.4807	6.3362	4.0387	12.6022	9.6948	9.4156	9.1431	5.4659	0
GSM79272	1	0	1	0	0	1	3	19	39	1	1.42	1	12.2158	7.5446	4.3239	11.9861	10.6328	9.5058	8.8141	5.3105	0
GSM79273	0	0	1	0	0	1	3	33	40	0	10.33	0	12.7007	7.7982	4.4366	12.8138	10.7092	10.5980	8.4267	5.2987	0
GSM79274	0	0	1	0	0	1	3	29	66	1	1.83	1	13.0296	6.3762	3.9808	12.4366	10.6740	9.9424	8.7427	4.9860	0
GSM79275	1	1	0	0	1	0	2	17	45	0	10.33	0	10.0138	9.4505	6.6604	12.2381	10.1098	10.2945	8.8481	5.1189	0
GSM79276	1	1	0	1	1	0	2	16	66	0	10.33	0	9.4194	11.7745	6.9740	12.2507	9.6060	10.3213	8.9292	4.9747	0
GSM79277	1	1	0	1	1	0	2	17	69	0	10.25	0	9.4512	11.3654	7.1421	12.8752	11.0992	9.4262	9.1469	5.4959	1
GSM79278	1	1	0	1	0	0	2	20	79	0	8.58	0	9.9180	10.2963	4.3174	13.1685	10.8737	9.6044	9.4088	5.3274	1
GSM79279	1	1	1	0	0	0	2	11	56	0	10.25	0	12.1044	6.8877	4.2814	12.7490	9.8540	10.6275	9.0012	5.7745	1
GSM79280	1	1	0	0	0	0	3	19	50	0	10.17	0	9.3183	6.5482	3.8666	12.4992	10.8315	10.1889	9.1791	5.3660	0
GSM79281	1	1	0	1	1	1	2	13	67	0	6.83	0	9.9046	10.6711	6.3776	12.8720	10.5561	11.4608	8.9928	5.3834	1
GSM79282	1	1	0	1	1	0	2	11	45	0	10.08	0	9.5618	9.8941	5.1894	12.4727	10.3060	9.7514	8.9124	5.3151	0
GSM79283	1	1	0	1	0	0	1	10	55				9.8745	9.9191	4.6334	12.4627	10.2488	9.9416	8.4451	5.2968	0
GSM79284	1	1	0	1	0	1	2	32	48	1	6.83	1	8.8779	10.0535	4.6755	12.4584	10.0008	10.0703	8.7630	5.2102	0
GSM79285	1	1	0	1	1	0	1	18	48	0	10.08	0	10.0942	10.1874	5.8739	12.3431	9.6984	9.7814	8.8325	5.3332	0
GSM79286	1	1	0	1	1	0	2	20	62	0	10.00	0	9.6881	11.2378	5.3599	12.2614	10.3337	9.8921	9.0567	5.4594	0
GSM79287	1	1	0	0	0	1	3	20	37	0	10.00	0	9.1271	6.4686	4.0517	12.1663	10.4880	9.5688	8.6078	4.9261	0
GSM79288	1	1	0	1	0	1	2	38	88	0	10.00	0	9.8275	11.3489	4.2827	12.0954	9.6554	9.4719	9.0462	5.0708	0
GSM79289	1	1	0	1	1	1	1	20	52	0	10.00	0	9.8792	10.3829	4.8932	12.4097	9.7778	9.3116	9.4818	5.2195	0
GSM79290	1	1	0	1	1	0	1	9	56	0	10.50	0	9.7268	11.3153	6.4790	12.4629	10.0758	9.4104	8.7257	5.4335	0
GSM79291	0	0	0	1	0	0	2	18	75	0	10.00	0	9.6376	11.0267	4.7765	12.3803	10.2386	9.4600	8.7386	4.9820	0
GSM79292	0	0	0	0	0	0	3	42	84	0	0.92	0	9.7460	6.9864	4.3393	12.7496	10.0895	10.0045	8.6294	5.1186	0
GSM79293	1	1	0	1	1	0	2	20	51	0	10.00	0	9.2122	10.6912	6.6798	12.4267	10.7847	9.8499	9.0974	5.4064	0
GSM79294	1	1	1	0	0	0	1	25	43	0	10.00	0	12.6234	7.9461	4.2011	12.7902	10.6702	9.8014	9.0084	5.1013	0
GSM79295	1	1	0	1	1	0	2	13	73	0	9.92	0	9.6419	10.9783	6.4673	12.3464	9.9698	9.3013	8.8754	5.2131	0
GSM79296	1	1	0	1	0	1	2	30	51	0	9.92	0	10.1710	10.0712	4.0981	12.6516	10.1618	9.9664	8.9270	5.2763	0
GSM79297	1	1	0	1	1	0	2	24	62	0	9.92	0	9.9979	11.6401	7.5555	12.5616	9.3424	9.2023	8.8963	5.0700	0
GSM79298	1	1	1	0	0	0	2	32	74	0	9.92	0	13.0461	6.6871	3.8250	12.4519	10.4411	9.4234	9.0432	5.0966	0
GSM79299	0	0	0	0	0	0	3	23	37	0	9.92	0	8.2852	6.5299	4.0895	12.8012	10.1702	10.0303	9.4636	5.2013	0
GSM79300	1	0	0	1	1	0	3	15	54	1	2.92	1	9.7771	10.0723	5.1514	12.4758	10.2941	10.5468	8.8794	5.2245	0
GSM79301	1	1	0	1	0	0	2	24	66	0	9.83	0	10.1141	11.3135	3.7834	12.6867	10.8051	10.2602	9.2548	5.5196	0
GSM79302	0	0	0	0	0	0	2	21	38	1	5.25	1	9.3600	7.4551	4.5097	12.3883	10.2136	9.9900	8.7521	5.2836	0
GSM79303	1	1	0	0	0	0	1	12	44	0	9.83	0	8.9804	6.6709	4.3531	11.9715	9.9597	9.8186	8.9677	5.2139	0
GSM79304	1	0	1	0	0	1	3	32	70	1	1.75	1	13.1903	8.2073	3.7128	13.1038	10.9329	9.7749	8.9297	6.1043	1
GSM79305	1	0	0	1	1	1	3	28	65	0	10.17	0	7.9241	12.7910	5.3977	12.5556	9.9793	9.4134	8.7743	5.8052	1
GSM79306	0	0	0	0	0	0	3	30	38	1	9.58	1	8.0002	6.1781	4.1327	12.6922	9.5585	8.8456	8.6317	5.3640	0
GSM79307	1	1	0	1	0	0	?	12	66	0	8.00	0	10.7369	10.1981	4.4941	12.0594	10.4820	9.3315	8.8382	5.2142	0
GSM79308	1	1	0	0	0	0	1	13	51	0	10.75	0	9.1674	8.4929	3.6044	12.3993	10.2882	9.0823	9.0643	5.1978	0
GSM79309	1	1	0	1	1	1	1	18	49				9.1880	10.2809	6.2612	12.1709	10.0332	8.5933	8.8666	5.2208	0
GSM79310	1	1	0	1	1	0	2	20	67				9.7085	10.7239	5.0757	12.0086	10.3436	9.1506	9.3919	5.4453	0
GSM79311	1	1	0	0	1	0	2	23	45	0	6.08	0	9.6632	9.2220	5.5847	12.2933	10.6665	9.3335	8.5524	5.0755	0
GSM79312	1	1	0	1	1	0	2	22	46	0	10.08	0	10.0780	9.7505	4.8394	12.4530	9.8490	9.2160	8.3883	5.4045	0
GSM79313	1	0	1	0	0	0	3	15	73	0	10.00	0	11.9295	7.5464	4.6832	12.6665	9.5641	10.1073	8.9807	5.4064	0
GSM79314	1	1	0	1	1	1	1	17	52	0	10.00	0	9.7945	10.4563	5.8541	12.4405	10.2497	9.0794	9.0318	4.9164	0
GSM79315	1	1	0	1	0	1	2	22	56	0	10.00	0	10.1541	10.8424	4.0702	12.9479	10.6223	9.1079	8.8637	5.7346	1
GSM79316	1	1	0	1	0	1	2	22	61	0	9.92	0	9.1982	9.9555	4.0019	12.2865	9.8138	9.2122	8.9124	4.8683	0
GSM79317		0	0	1	1	0	1	26	59	0	9.92	0	9.4545	10.2026	5.8841	12.0966	9.6919	9.5294	8.8890	5.4482	0
GSM79318	1	1	0	1	1	0	2	12	46				10.0721	9.9909	5.9872	12.2996	10.4138	9.5538	9.1992	5.7390	1
GSM79319	1	1	0	0	1	1	2	19	41	0	12.75	0	10.0889	9.2673	5.7651	12.8786	10.8090	9.9352	9.9548	5.8084	1
GSM79320	1	1	0	1	1	0	1	18	75	0	12.75	0	10.2298	11.4332	5.0666	12.7643	11.0229	9.8365	8.8367	5.7070	1
GSM79321	1	1	1	1	1	1	3	22	74	1	0.08	1	11.1863	11.8619	7.0809	13.1409	11.4530	9.4538	9.1522	5.3554	1
GSM79322	0	0	0	0	0	1	2	53	61	0	10.58	0	9.2921	7.3527	4.2044	12.4888	10.0769	8.8513	8.5721	5.1301	0
GSM79323	1	1	0	1	0		1	38	78	0	1.83	0	9.3356	10.1699	3.8821	12.3368	9.6874	9.5750	8.8555	5.5846	1
GSM79324	1	1	0	1	0	0	1	14	80	1	6.67	1	9.4582	10.6770	4.7464	12.2505	10.0326	8.7588	9.0266	5.2221	0
GSM79325	1	0	0	1	0	1	2	22	47	1	9.58	1	9.4656	10.1360	4.1543	12.8971	10.3249	10.2898	9.3717	5.8513	1
GSM79326	1	1	0	1	1	1	2	32	59				9.5072	12.3888	5.0269	12.5446	9.8223	8.2986	9.1237	5.3424	0
GSM79327	1	1	0	1	1	0	1	24	53	0	12.67	0	9.8001	10.4223	6.4337	12.1810	10.2857	8.4670	8.9290	5.2771	0
GSM79328	1	1	0	1	0	1	2	21	82	1	3.17	1	9.8531	11.6425	4.3871	12.9743	11.7582	9.6635	9.1834	5.6265	1
GSM79329	0	0	0	0	0	0	3	14	68	0	12.58	0	8.4288	6.8792	4.0006	12.1361	8.9343	9.6137	9.4062	5.2530	0
GSM79330	1	0	0	1	0	1	3	41	62	0	3.25	0	10.1545	11.5437	4.4211	13.3951	10.7773	10.0007	8.6763	5.2959	1
GSM79331	1	1	0	1	0	1	2	45	52	1	2.25	1	9.3019	9.9716	4.1008	12.5333	10.2376	7.8900	8.9944	4.7075	0
GSM79332	1	0	1	0	0	1	2	8	47	1	1.33	1	12.9441	7.9470	4.6608	12.8936	10.1323	9.7565	9.2671	5.3948	1
GSM79333	1	1	1	0	0	0	2	26	37	1	5.50	1	12.6695	9.1364	4.2679	12.3991	9.8437	8.7176	9.1327	5.3133	0
GSM79334	1	1	0	1	1	0	2	36	77	1	0.92	1	9.9925	9.6297	5.3144	12.7850	10.0871	9.7801	8.6775	5.2444	0
GSM79335	1	1	0	1	1	0	1	14	68	0	12.50	0	10.0049	11.6367	5.1580	12.2974	9.9315	9.0709	9.0529	5.2792	0
GSM79336	0	0	0	0	0	0	3	24	77	1	0.33	1	9.5080	6.3790	4.3112	12.4011	10.7213	8.6940	9.0523	5.4911	0
GSM79337	1	1	0	1	0	1	2	24	55	1	5.00	1	9.2993	10.7761	4.4037	12.2968	10.2762	7.5666	8.4248	5.2010	0
GSM79338	1	0	0	1	0	0	2	28	61	0	12.42	0	10.4546	11.5083	4.3260	12.3924	9.6353	8.8523	9.0875	5.4770	0
GSM79339	1	1	0	0	0	0	1	18	49	0	12.42	0	9.9558	8.0657	4.7083	12.7679	10.8141	9.9980	9.0353	5.7339	1

GSM79340	1	0	1	1	1	1	3	35	48	1	1.42	1	12.7670	9.8958	5.6823	12.5686	10.6210	9.6137	8.8711	5.8744	1
GSM79341	0	0	1	0	0	1	3	28	79	1	1.17	1	11.5834	5.0850	4.2681	12.6415	10.4352	8.9568	8.6016	5.8815	1
GSM79342	1	1	0	0	0	1	3	50	62	0	12.42	0	8.9635	9.0631	3.9781	12.3937	10.0520	9.4650	8.6058	5.0118	0
GSM79343	1	1	0	1	0	1	1	26	71	1	1.67	1	10.1584	11.0726	3.6622	12.7845	10.9853	9.5595	8.6733	5.4823	1
GSM79344	1	0	0	0	0		3	15	87	0	12.00	0	9.3170	7.2032	4.0991	12.4173	9.8424	10.6455	8.7772	5.0687	0
GSM79345	1	1	0	1	1	0	1	16	55	0	12.33	0	9.5041	10.7850	4.9821	12.2657	10.5483	9.8656	8.9231	5.4424	0
GSM79346	1	1	1	0	0	1	3	130	44				12.3834	6.8545	3.9027	12.5188	10.4028	9.1771	9.0154	5.6595	1
GSM79347	1	1	0	1	1	0	1	22	56	0	12.33	0	9.7121	10.4073	5.3436	12.2692	10.5134	9.3923	8.5192	5.4867	0
GSM79348	1	1	0	1	0	0	2	19	69	1	11.92	1	9.4510	11.3041	4.0263	12.4597	10.3485	8.9686	8.9668	5.4373	0
GSM79349	1	0	0	1	1	1	3	65	81	0	2.08	0	9.7584	11.5852	7.7927	12.2703	9.7220	9.6129	8.6248	5.3969	0
GSM79350	1	0	0	1	1	1	2	20	75	0	1.17	0	10.9551	11.5045	5.5180	12.4987	10.6011	9.3608	8.8077	5.2676	0
GSM79351	0	0	1	1	0	1	3	25	65	0	0.83	0	12.1848	11.6453	4.4286	12.9783	10.2670	9.2071	8.8634	5.1060	1
GSM79352	1	1	0	0	0	1	2	19	52	1	4.58	1	9.3111	8.8822	4.6802	12.2514	10.2912	9.7009	8.8693	5.0913	0
GSM79353	1	1	0	1	0	1	3	27	72	0	2.58	0	10.3102	12.1985	4.2416	12.7653	10.5009	9.1750	9.2044	5.0584	0
GSM79354	1	1	0	1	0	0	2	28	84	0	12.17	0	9.6456	9.7642	4.1400	12.2496	10.6299	9.3836	9.6504	5.4708	0
GSM79355	1	1	0	1	0	1	2	15	86	0	2.08	0	10.7879	10.6034	4.1294	13.0296	10.5298	9.4308	9.0616	5.5953	1
GSM79356	0	0	0	0	0	0	2	24	61	0	12.17	0	9.4638	7.4727	4.2191	12.3898	10.9990	9.1993	9.2086	5.8415	1
GSM79357	1	1	0	1	0	0	2	12	36	0	12.08	0	9.1324	9.8175	4.2708	12.7364	10.7603	8.6669	9.0581	5.1406	0
GSM79358	1	1	0	1	0	0	2	24	63	0	12.08	0	9.8400	11.1933	4.4437	12.6807	10.4094	8.9437	9.1178	5.5321	0
GSM79359	1	1	0	1	0	0	1	17	58	0	11.33	0	9.9099	10.5456	4.1328	12.5747	9.0944	8.8798	8.4789	5.2167	0
GSM79360	1	0	1	1	0	0	3	26	76	0	2.67	0	12.5684	12.3806	4.2505	12.7635	10.3042	9.1242	9.3060	5.4848	0
GSM79361	1	1	0	1	1	0	2	6	73				9.7334	10.9233	5.2859	12.2519	10.1445	9.4186	8.6015	5.6998	1
GSM79362	1	1	1	1	1	1	2	38	63	1	0.25	1	11.2090	11.9392	5.5224	12.8367	10.4894	10.6523	8.9259	5.4864	1
GSM79363	1	1	0	1	0	0	1	19	82	0	10.50	0	9.6042	10.2773	4.3702	12.2282	10.0824	8.2437	8.8643	5.2360	0
GSM79364	1	1	0	1	0	0	2	12	71	0	11.33	0	10.1073	11.7563	4.6896	12.3164	10.6548	9.2427	8.6687	5.2788	0
