# Supplementary figures and images for: Amplification and high-level expression of heat shock protein 90 marks aggressive phenotypes of human epidermal growth factor receptor 2 negative breast cancer
Source: Breast Cancer Res. 2012 Apr 17;14(2):R62. doi: 10.1186/bcr3168 (PMC3446397; doi:10.1186/bcr3168)

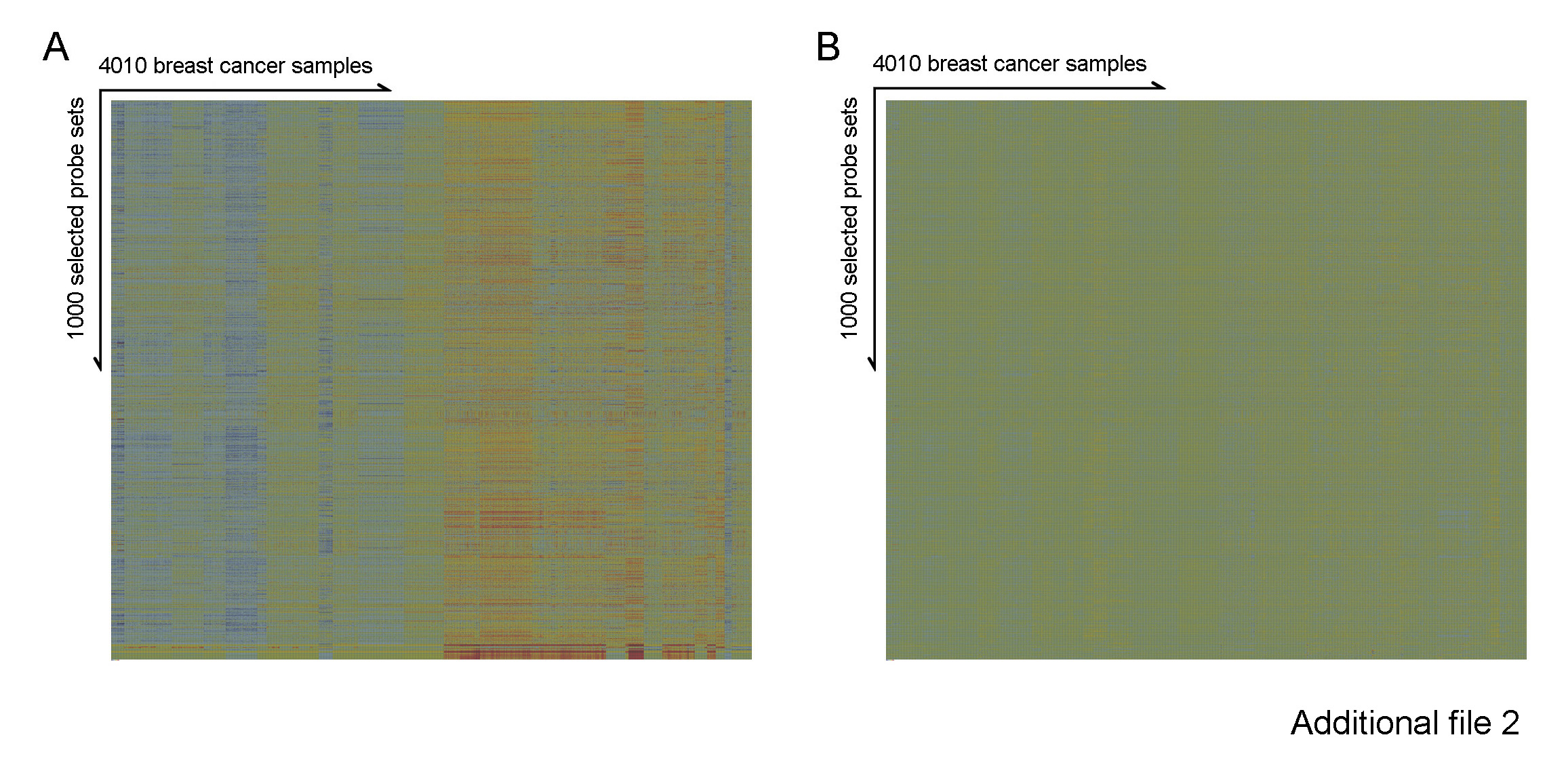

Supplement: Additional file 2 — Heatmaps. These heatmaps show the expression patterns in the data before (A) and after (B) normalization. The rows contain the 1,000 genes that exhibit the highest variance in gene expression profile across the original data set. The columns contain the samples in the data sets provided. The genes and samples are in the same order in both heatmaps. Warm colors indicate high expression of the gene and cool colors indicate low expression. [file bcr3168-S2.JPEG]

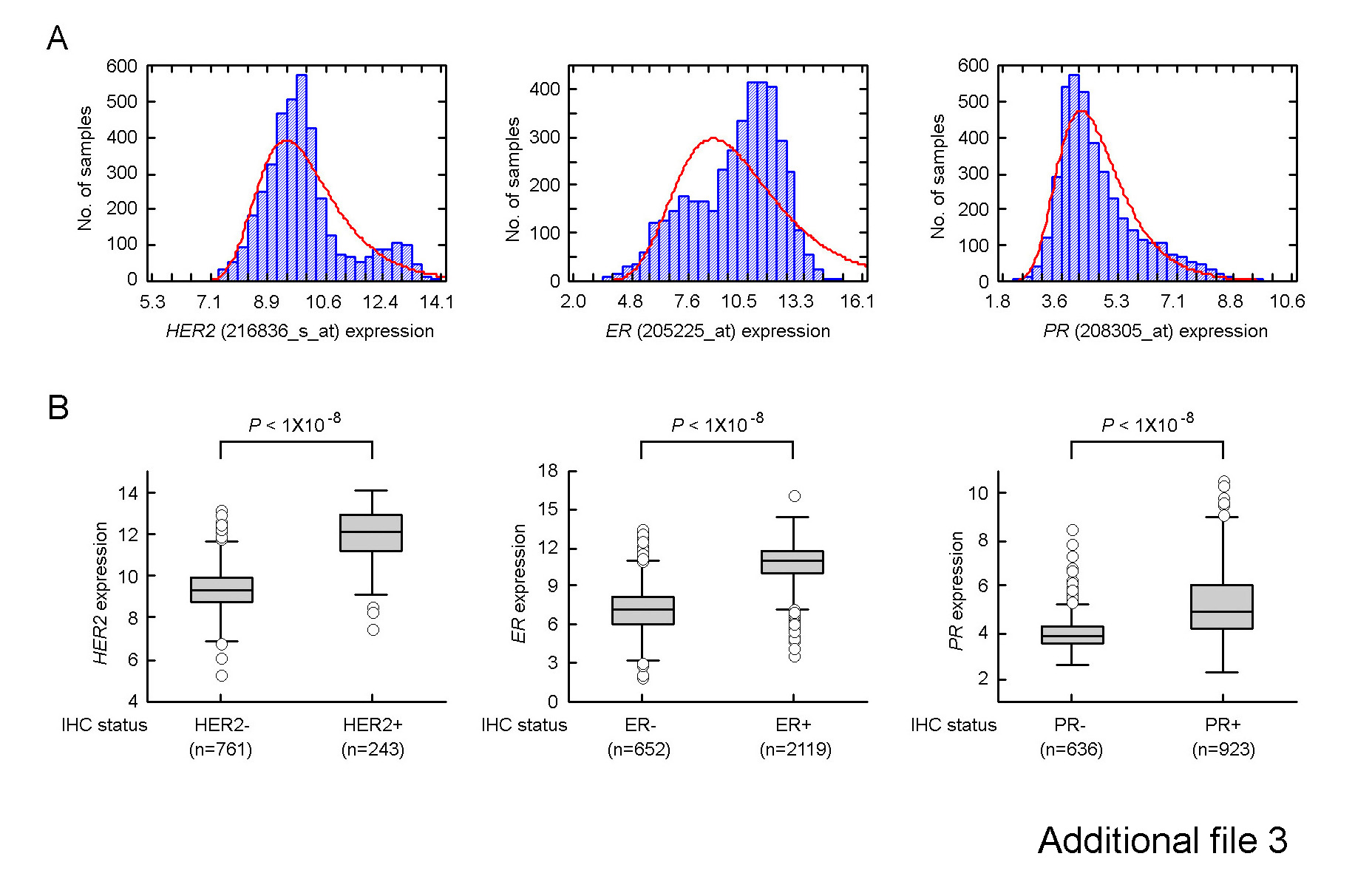

Supplement: Additional file 3 — Distribution of HER2, ER and PR mRNA expression and its correlation with IHC measure molecular status. This figure shows (A) histograms of HER2, ER and PR mRNA expression in 4,010 breast cancer samples and (B) the correlation between mRNA expression and IHC status. Differences between positive and negative groups were assessed using the exact Mann-Whitney U test. Boxes represent the 25% to 75% quartiles, lines in the boxes represent the median level, whiskers represent the non-outlier range, and circles represent the outliers. [file bcr3168-S3.JPEG]

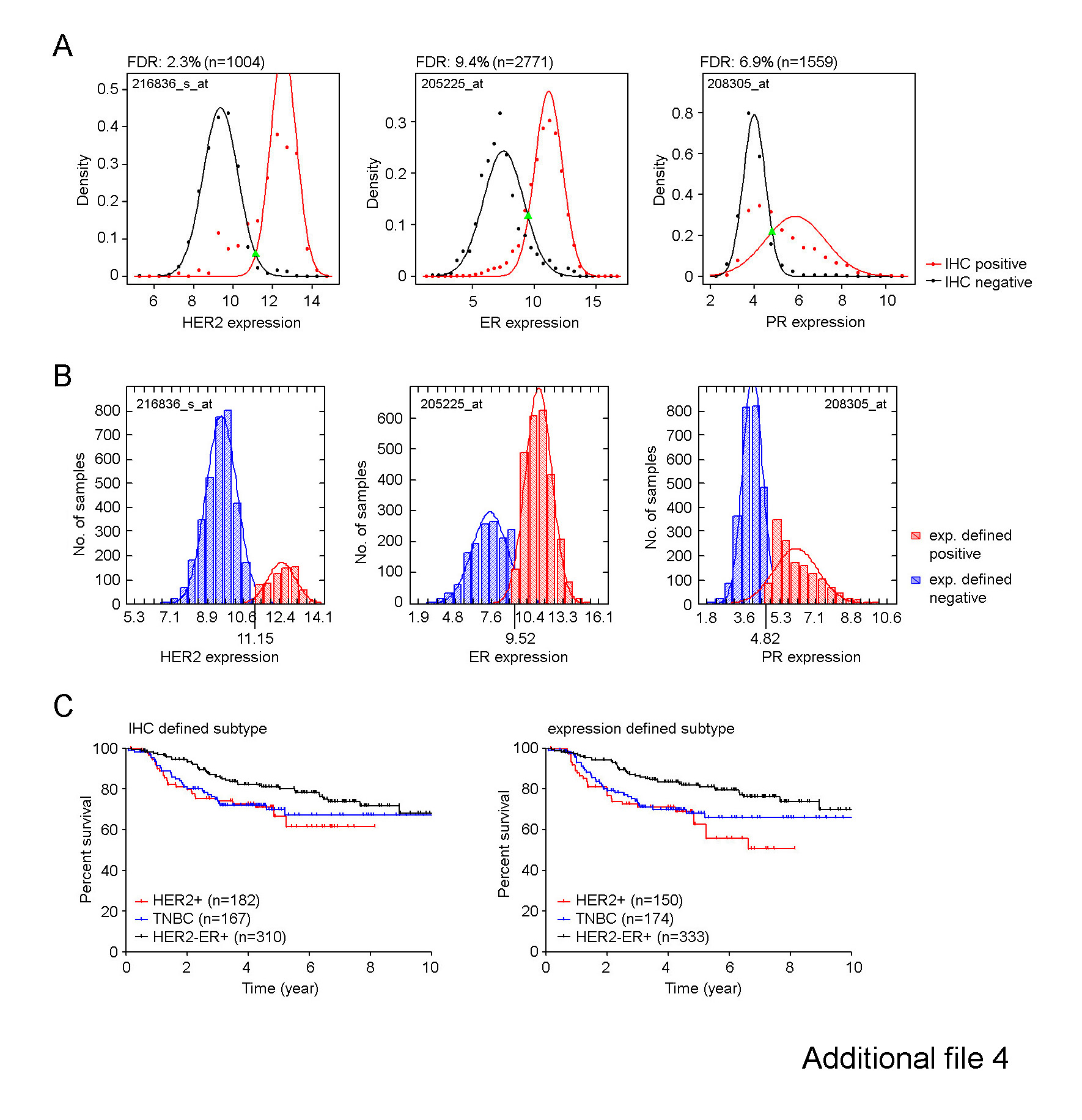

Supplement: Additional file 4 — Expression defined breast cancer subtypes. This figure shows (A) Bimodal selection for HER2, ER and PR cutoff according to the distribution of expression values stratified by IHC/biochemical status. (B) Distribution of HER2, ER and PR mRNA expression in combined dataset. (C) Distant metastasis-free survival analyses were stratified according to IHC/biochemical status or expression derived status using samples with available IHC/biochemical status and outcome data. Tick marks in Kaplan-Meier Estimates distant-metastasis free survival indicate patients whose data were censored by the time of last follow-up or owing to death. P values were calculated using log-rank Mantel-cox test. [file bcr3168-S4.JPEG]

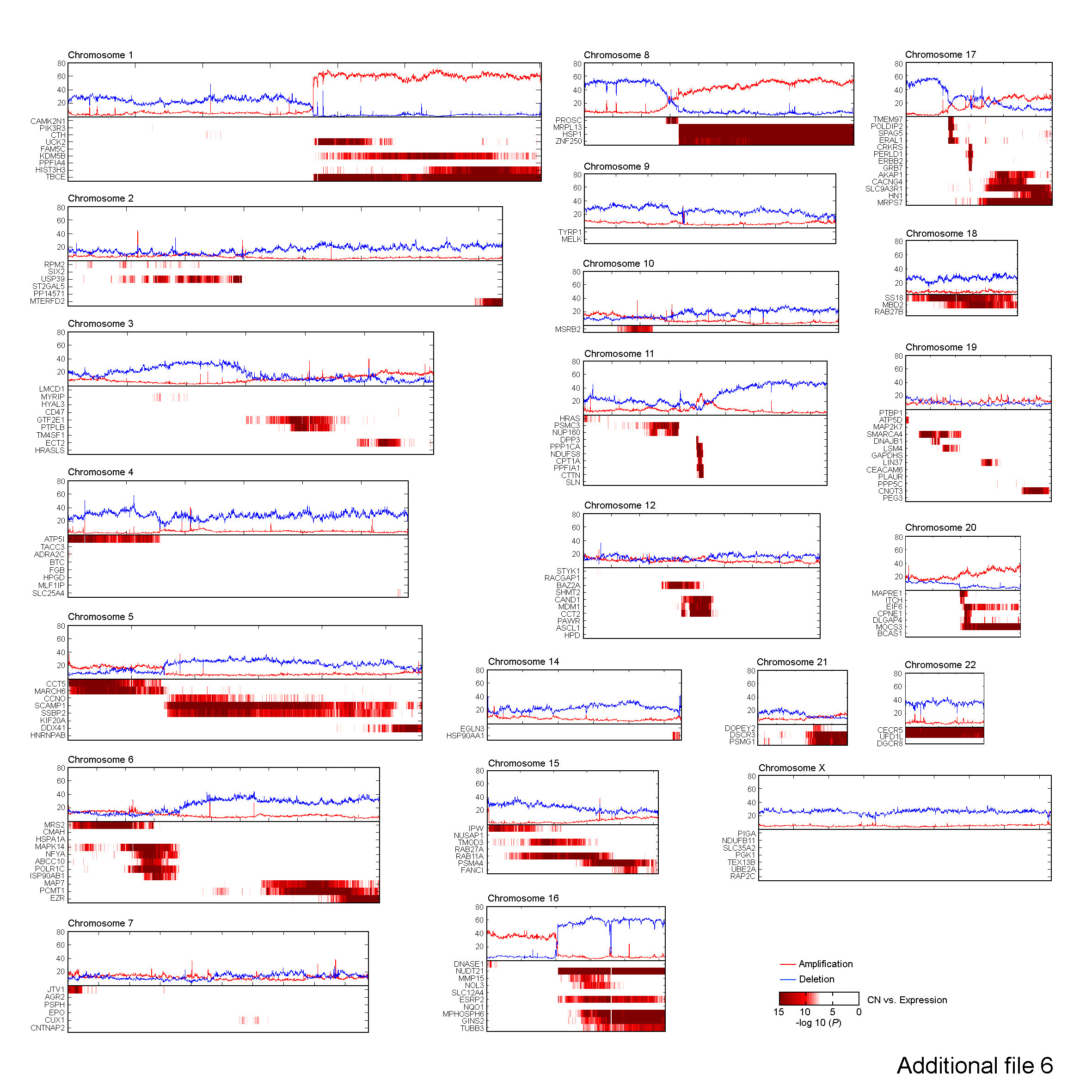

Supplement: Additional file 6 — Genome scans for poor prognosis associated gene. This figure shows the correlation between copy number aberrations and gene expression of identified genes that were associated with breast cancer poor prognosis. Upper panel shows percentage of amplification (low-level and high-level amplification) and deletion (homozygous and hemizygous deletion) at each detected chromosome region in a group of 481 breast cancer patients. Bottom panel shows correlation between CNA and mRNA expression of poor prognosis associated genes that were identified from each chromosome. Analysis of variance (ANOVA) was performed to test for association between copy numbers and gene expression. [file bcr3168-S6.JPEG]

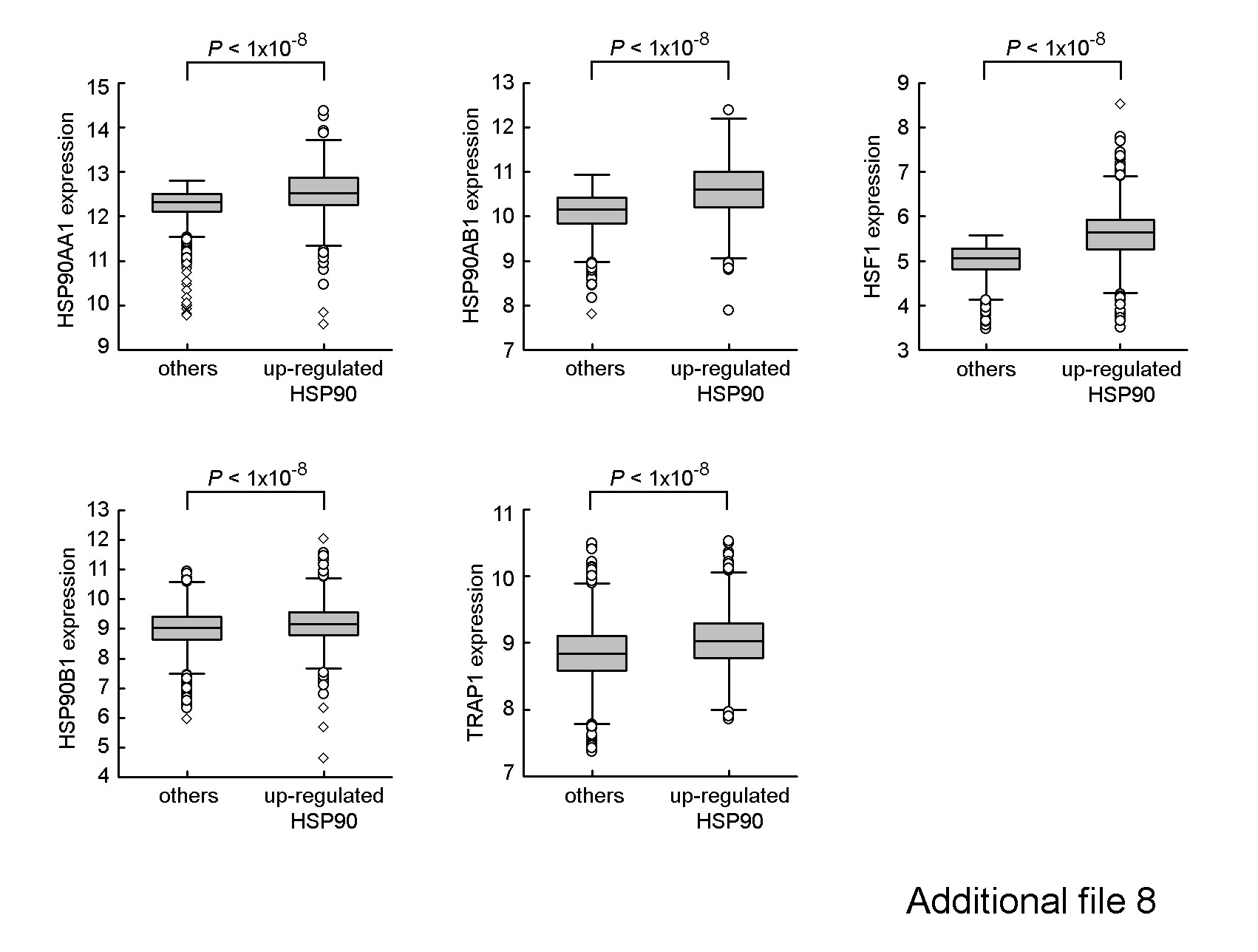

Supplement: Additional file 8 — Correlation between HSP90 and HSF1 mRNA expression and up-regulated HSP90. This figure shows HSP90 and HSF1 expression difference between samples defined as up-regulated HSP90 and not up-regulated HSP90. Differences for each pairwise comparison were assessed by the Mann-Whitney U test. Boxes represent the 25% to 75% quartiles, lines in the boxes represent the median level, whiskers represent the non-outlier range, and circles represent the outliers. [file bcr3168-S8.JPEG]

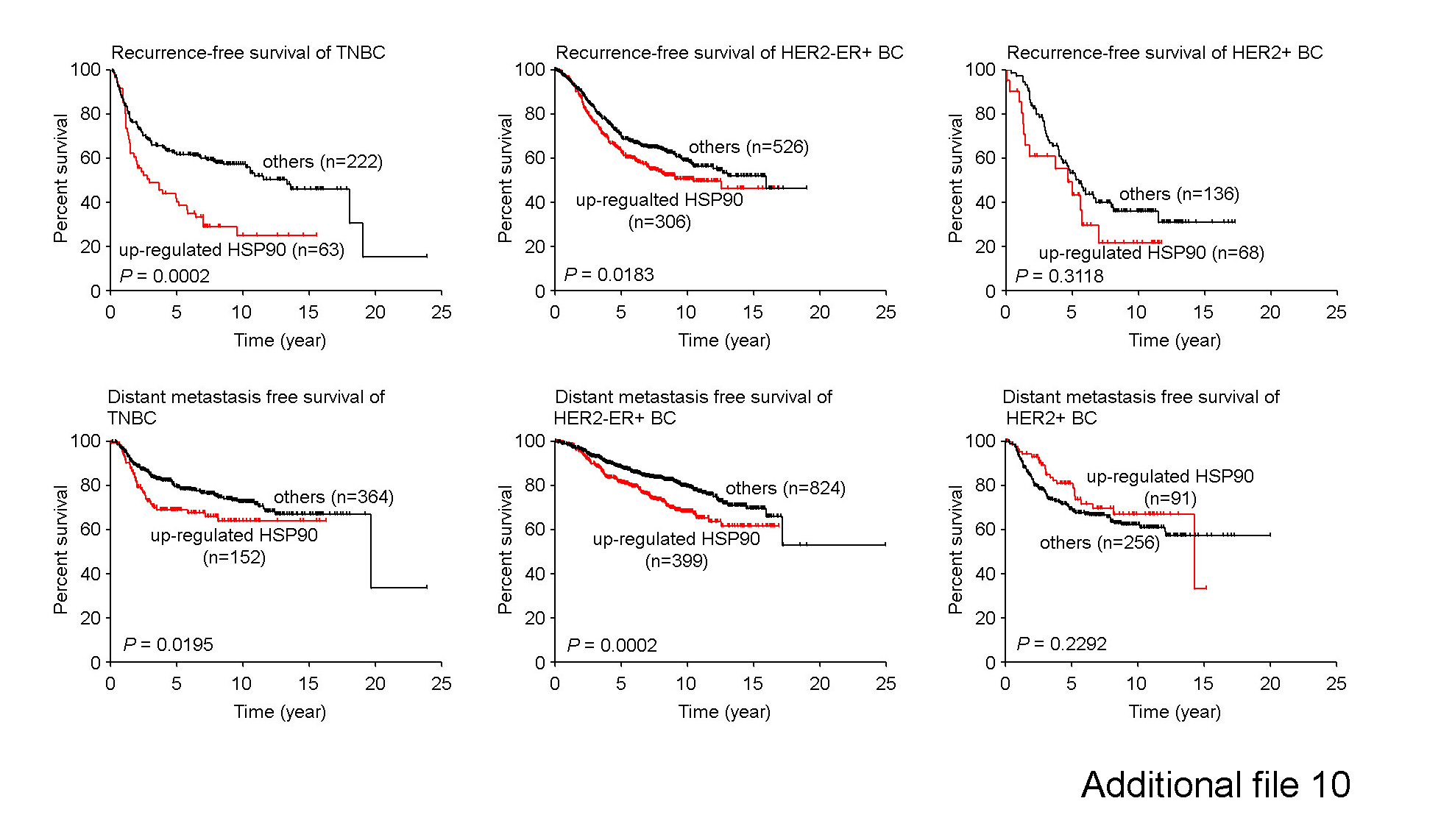

Supplement: Additional file 10 — Prognosis of up-regulated HSP90 in different breast cancer subtypes. This figure shows Kaplan-Meier estimates curve of up-regulated HSP90 in different breast cancer subtypes. Number of recurrence events: TNBC, n = 142; HER2-/ER+, n = 331; HER2+, n = 112. Number of distant metastasis events: TNBC, n = 133; HER2-/ER+, n = 260; HER2+, n = 111. Tick marks in Kaplan-Meier estimates of recurrence-free survival and distant-metastasis free survival indicate patients whose data were censored by the time of last follow-up or owing to death. P values were calculated using log-rank Mantel-cox test. [file bcr3168-S10.JPEG]
