# Supplementary material for: Amplification and high-level expression of heat shock protein 90 marks aggressive phenotypes of human epidermal growth factor receptor 2 negative breast cancer
Source: Breast Cancer Res. 2012 Apr 17;14(2):R62. doi: 10.1186/bcr3168 (PMC3446397; doi:10.1186/bcr3168)
Supplement: Additional file 5 — Breast cancer poor prognosis associated gene. This table lists breast cancer poor prognosis -ssociated genes. Cox-regression survival analyses were performed using 395 samples in which event of death from breast cancer was available. Analysis of variance (ANOVA) was performed to test for an association between copy numbers and gene expression using 481 TCGA breast cancer samples. [file bcr3168-S5.PDF]

Additional file 5. Breast cancer poor prognosis associated gene

Gene	cutoff at 10%		3 quartiles	expression signal	CN_exp (*P*<1.00E-8)	final26
	p-value	FDR	p-value	p-value		
PPFIA1	2.96E-06	0.01850	0.00734	4.51E-06	yes	1
DOPEY2	3.63E-05	0.03582	0.02727	0.00183	yes	1
EZR	0.00014	0.06782	0.00037	5.46E-05	yes	1
GINS2	0.00032	0.09231	0.00147	8.50E-05	yes	1
LSM4	0.00053	0.11166	0.02904	0.04332	yes	1
TMEM97	0.00055	0.11209	0.42282	0.00500	yes	1
HSP90AA1	0.00062	0.11697	0.00858	0.00205	yes	1
CUX1	0.00064	0.11697	0.04488	0.04119	yes	1
PSMG1	0.00068	0.11697	0.33329	0.04704	yes	1
ESRP2	0.00090	0.13184	0.00028	6.90E-05	yes	1
TUBB3	0.00103	0.13995	0.02017	0.00929	yes	1
ATP5I	0.00220	0.18415	0.03546	0.01899	yes	1
ERBB2	0.00254	0.19439	0.02275	0.00021	yes	1
EIF6	0.00307	0.20856	0.09761	0.00525	yes	1
GRB7	0.00406	0.22435	0.05537	0.00031	yes	1
SPAG5	0.00482	0.23353	0.04192	1.42E-05	yes	1
MMP15	0.00565	0.24637	0.00597	4.18E-05	yes	1
MAPRE1	0.00699	0.26303	0.06952	0.02565	yes	1
DDX41	0.00705	0.26332	0.19317	0.01149	yes	1
CCT5	0.00783	0.27282	0.06623	0.02836	yes	1
HSP90AB1	0.00802	0.27509	0.02521	0.01363	yes	1
SMARCA4	0.00843	0.28108	0.00353	0.00070	yes	1
PTPLB	0.00887	0.28576	0.00563	0.00416	yes	1
NOL3	0.00915	0.28880	0.01244	0.00065	yes	1
CTTN	0.00968	0.29460	0.00282	0.00041	yes	1
MAP7	0.00987	0.29657	0.08342	0.02132	yes	1
NQO1	1.46E-05	0.02738	3.98E-05	2.43E-05	no	
MAR_6	3.28E-05	0.03531	0.13370	0.03225	no	
SLC35A2	5.14E-05	0.04190	0.04639	0.01272	no	
EPO	0.00013	0.06782	0.52147	0.02072	no	
SSBP2	0.00020	0.07500	0.38644	0.10132	yes	
ITCH	0.00024	0.07684	0.11081	0.00541	yes	
PPP5C	0.00025	0.07684	0.13046	0.01183	no	
GAPDHS	0.00029	0.08631	0.80272	0.03260	no	
RACGAP1	0.00035	0.09651	0.00088	2.24E-05	no	
MELK	0.00044	0.10855	0.00023	0.00062	no	
CEACAM6	0.00044	0.10855	0.05266	0.00466	no	
PPP1CA	0.00046	0.11119	0.01408	0.00078	no	
DNASE1	0.00049	0.11119	0.60626	0.03239	yes	
SLC9A3R1	0.00053	0.11166	0.04712	0.05989	yes	
CCNO	0.00054	0.11209	0.03839	0.01765	no	
PP14571	0.00064	0.11697	0.00968	0.00603	no	
NUSAP1	0.00067	0.11697	0.00011	7.22E-05	no	

Additional file 5. Breast cancer poor prognosis associated gene (continued)

Gene	cutoff at 10%		3 quartiles	expression signal	CN_exp	final26
	p-value	FDR	p-value	p-value	(P <1.00E-8)	
MDM1	0.00073	0.12279	0.92171	0.08061	yes	
TMOD3	0.00074	0.12279	0.77064	0.14664	yes	
CPT1A	0.00081	0.12450	0.07126	0.00215	yes	
CNTNAP2	0.00083	0.12450	0.00844	0.00100	no	
CMAH	0.00083	0.12450	0.17239	0.10010	no	
RRM2	0.00114	0.14741	0.00064	0.00010	no	
FGB	0.00119	0.14854	0.80223	0.04962	no	
UCK2	0.00120	0.14854	0.56060	0.12613	yes	
TEX13B	0.00129	0.15212	0.25818	0.16043	no	
CNOT3	0.00138	0.15777	0.10709	0.07660	yes	
SCAMP1	0.00144	0.16086	0.42638	0.32082	yes	
PGK1	0.00172	0.16500	0.01429	0.00061	no	
KIF20A	0.00176	0.16500	0.19171	0.02012	no	
DNAJB1	0.00184	0.16995	0.51458	0.44942	yes	
BTC	0.00188	0.17112	0.38684	0.86564	no	
STYK1	0.00208	0.17890	0.02825	0.10623	no	
CAND1	0.00222	0.18418	0.49038	0.09595	yes	
MTERFD2	0.00225	0.18422	0.62876	0.19672	yes	
MLF1IP	0.00231	0.18510	0.00804	0.00017	no	
MAPK14	0.00243	0.19141	0.41152	0.09141	yes	
CRKRS	0.00245	0.19141	0.45909	0.00187	yes	
POLDIP2	0.00250	0.19370	0.25525	0.02326	yes	
MRPS7	0.00256	0.19512	0.65967	0.43767	yes	
ZNF250	0.00270	0.19931	0.39193	0.89372	yes	
CCT2	0.00274	0.19985	0.07371	0.00185	yes	
AGR2	0.00275	0.19985	0.01879	0.00791	no	
USP39	0.00283	0.20487	0.30007	0.06641	yes	
ADRA2C	0.00287	0.20618	0.56691	0.04979	no	
CTH	0.00291	0.20739	0.76561	0.57249	no	
NUP160	0.00296	0.20779	0.96171	0.66695	yes	
PERLD1	0.00301	0.20810	0.34358	0.00479	no	
DGCR8	0.00303	0.20810	0.16186	0.12687	no	
PSMA4	0.00303	0.20810	0.35513	0.12832	yes	
PPFIA4	0.00309	0.20916	0.84857	0.27832	no	
HSPA1A	0.00311	0.20976	0.12537	0.05091	no	
DPP3	0.00320	0.21277	0.06080	0.02007	yes	
PROSC	0.00342	0.21730	0.16502	0.01982	yes	
PSPH	0.00347	0.21730	0.93605	0.59812	no	
JTV1	0.00349	0.21730	0.69405	0.25142	yes	
FANCI	0.00350	0.21730	0.08315	0.00341	yes	
IPW	0.00360	0.21963	0.50066	0.76186	yes	
HPD	0.00361	0.21963	0.09823	0.00469	no	
CACNG4	0.00366	0.21963	0.31723	0.05013	yes	

Additional file 5. Breast cancer poor prognosis associated gene (continued)

Gene	cutoff at 10%		3 quartiles	expression signal	CN_exp	final26
	p-value	FDR	p-value	p-value	(*P* <1.00E-8)	
RAB27A	0.00370	0.21963	0.09235	0.00938	no	
LIN37	0.00372	0.21963	0.73097	0.27656	yes	
UBE2A	0.00373	0.21963	0.16073	0.00281	no	
EGLN3	0.00374	0.21963	0.83615	0.22964	no	
PAWR	0.00376	0.21963	0.13286	0.03089	yes	
ASCL1	0.00384	0.22086	0.14919	0.06555	no	
BAZ2A	0.00387	0.22092	0.39988	0.24626	yes	
RAB11A	0.00389	0.22092	0.96471	0.21964	yes	
CPNE1	0.00396	0.22231	0.20979	0.00544	yes	
SIX2	0.00411	0.22435	0.38775	0.03452	no	
RAP2C	0.00413	0.22435	0.09158	0.03201	no	
HYAL3	0.00420	0.22479	0.36955	0.90754	no	
CAMK2N1	0.00429	0.22563	0.49946	0.06845	no	
MOCS3	0.00431	0.22563	0.41897	0.72247	yes	
DLGAP4	0.00432	0.22563	0.05177	0.00845	yes	
TYRP1	0.00441	0.22842	0.66953	0.00078	no	
MBD2	0.00456	0.22878	0.98310	0.82771	yes	
ECT2	0.00470	0.22987	0.12794	0.03090	yes	
FAM5C	0.00491	0.23689	0.00462	0.00014	no	
ERAL1	0.00496	0.23724	0.42064	0.01561	yes	
HSF1	0.00522	0.24081	0.21850	0.15177	yes	
MSRB2	0.00527	0.24088	0.81042	0.43712	yes	
UFD1L	0.00537	0.24321	0.10212	0.03430	yes	
SHMT2	0.00537	0.24321	0.08252	0.03811	no	
SS18	0.00562	0.24637	0.91467	0.07435	yes	
MYRIP	0.00580	0.25043	0.78826	0.06625	yes	
HIST3H3	0.00584	0.25043	0.16401	0.06623	yes	
NDUFB11	0.00599	0.25378	0.70862	0.54079	no	
HN1	0.00604	0.25378	0.27540	0.01375	yes	
SLN	0.00605	0.25378	0.12564	0.47382	no	
HPGD	0.00610	0.25417	0.97168	0.07435	no	
NFYA	0.00619	0.25549	0.13254	0.22820	yes	
MAP2K7	0.00643	0.25717	0.29562	0.16413	no	
PCMT1	0.00643	0.25717	0.11839	0.04963	yes	
HRAS	0.00643	0.25717	0.02611	0.01798	no	
TM4SF1	0.00650	0.25820	0.69038	0.45573	no	
NDUFS8	0.00655	0.25820	0.01827	0.01258	yes	
TACC3	0.00661	0.25820	0.00608	0.00090	no	
TBCE	0.00683	0.26029	0.48004	0.09217	yes	
POLR1C	0.00685	0.26052	0.67920	0.19831	yes	
MPHOSPH6	0.00698	0.26303	0.48488	0.12805	yes	
SLC12A4	0.00712	0.26488	0.06335	0.10913	no	
PTBP1	0.00773	0.27282	0.82706	0.42243	yes	

Additional file 5. Breast cancer poor prognosis associated gene (continued)

Gene	cutoff at 10%		3 quartiles	expression signal	CN_exp (*P* <1.00E-8)	final26
	p-value	FDR	p-value	p-value		
SLC25A4	0.00777	0.27282	0.01183	0.02405	no	
BCAS1	0.00779	0.27282	0.00334	0.00072	no	
MRPL13	0.00788	0.27282	0.44896	0.14543	yes	
PSMC3	0.00803	0.27509	0.16340	0.09408	yes	
RAB27B	0.00819	0.27753	0.16183	0.00431	no	
ATP5D	0.00841	0.28108	0.84626	0.48348	yes	
CECR5	0.00844	0.28108	0.86668	0.11980	yes	
HRASLS	0.00848	0.28142	0.78346	0.87587	no	
MRS2	0.00855	0.28272	0.07122	0.16094	yes	
LMCD1	0.00862	0.28272	0.53523	0.82581	no	
CD47	0.00863	0.28272	0.39891	0.12334	no	
ABCC10	0.00864	0.28272	0.21282	0.08210	yes	
HNRNPAB	0.00870	0.28370	0.10186	0.01815	no	
AKAP1	0.00899	0.28716	0.07922	0.37147	yes	
KDM5B	0.00908	0.28790	0.22701	0.02908	yes	
PLAUR	0.00924	0.29020	0.96471	0.50016	no	
GTF2E1	0.00928	0.29073	0.79237	0.38880	yes	
PIK3R3	0.00950	0.29280	0.54915	0.36886	no	
ST3GAL5	0.00968	0.29460	0.64881	0.33519	no	
NUDT21	0.00971	0.29460	0.72690	0.04831	yes	
DSCR3	0.00978	0.29577	0.21662	0.08187	yes	
PIGA	0.00980	0.29589	0.09175	0.18544	no	
PEG3	0.00986	0.29657	0.84134	0.53121	no	
