# Supplementary material for: Amplification and high-level expression of heat shock protein 90 marks aggressive phenotypes of human epidermal growth factor receptor 2 negative breast cancer
Source: Breast Cancer Res. 2012 Apr 17;14(2):R62. doi: 10.1186/bcr3168 (PMC3446397; doi:10.1186/bcr3168)
Supplement: Additional file 7 — Prognosis of HSP90 and HSF1 in different breast cancer subtypes. This table lists the results of survival analyses. Breast cancer subtype specific disease-specific survival (dss, event of death from breast cancer), over-all survival (os), recurrence-free survival (rfs), and distant metastasis-free survival (dmfs) were assessed using Cox-regression survival analysis, Kaplan-Meier Estimates survival analysis and Cox Proportional-Hazards (COXPH) Regression survival analysis. [file bcr3168-S7.PDF]

Additional file 7. Prognosis of HSP90 and HSF1 in different breast cancer subtypes

subtype	phenotype	gene	Cox-regression analysis		Kaplan-Meier survival analysis				COXPH survival analysis		
					Hing 25% vs. others		Hing 10% vs. others		phenotype	p-value	p_adjusted
			p-value	Coef	p-value	HR (95%CI)	p-value	HR (95%CI)			
All samples	dss	HSP90AA1	0.0020	1.0475	0.0499	1.75 (1.00-3.06)	0.0241	2.81 (1.15-6.90)	dss	0.0193	0.3320
	(n=395)	HSP90AB1	0.0136	0.5079	0.0404	1.72 (1.02-2.90)	0.0011	3.69 (1.68-8.07)	(n=225)	0.0022	0.0008
		HSP90B1	0.5979	0.0836	0.2429	1.33 (0.82-2.15)	0.8838	0.96 (0.52-1.75)		0.9469	0.8447
		TRAP1	0.3435	-0.3231	0.7019	1.10 (0.67-1.80)	0.8557	0.93 (0.44-1.98)		0.0592	0.9955
		HSF1	0.1518	0.4842	0.5832	1.22 (0.60-2.49)	0.0015	8.49 (2.27-31.77)		0.0001	0.0540
	os	HSP90AA1	0.0081	0.5074	0.0384	1.39 (1.02-1.89)	0.0002	2.55 (1.55-4.21)	os	0.0048	0.1069
	(n=1072)	HSP90AB1	0.0175	0.3077	0.0401	1.36 (1.01-1.83)	0.0024	2.06 (1.29-3.28)	(n=421)	0.0010	0.0022
		HSP90B1	0.5204	-0.0709	0.8472	1.03 (0.76-1.40)	0.4348	0.83 (0.53-1.31)		0.9893	0.3978
		TRAP1	0.5454	-0.0980	0.5325	1.10 (0.82-1.46)	0.4658	0.86 (0.58-1.29)		0.0576	0.0521
		HSF1	0.8446	0.0259	0.692	1.09 (0.72-1.66)	0.3261	1.32 (0.76-2.28)		0.0006	0.0073
HER2+	os	HSP90AA1	0.7459	0.1499	0.694	0.89 (0.50-1.60)	0.1523	2.07 (0.76-5.61)	os	0.4364	0.2703
	(n=194)	HSP90AB1	0.5693	-0.1765	0.6728	1.15 (0.59-2.24)	0.3733	1.76 (0.51-6.13)	(n=63)	0.0000	0.1839
		HSP90B1	0.5517	-0.1402	0.6199	0.86 (0.47-1.58)	0.4223	0.68 (0.26-1.75)		0.7232	0.2433
		TRAP1	0.6860	-0.1484	0.9693	1.02 (0.48-2.16)	0.4499	0.58 (0.14-2.36)		NA	NA
		HSF1	0.7863	0.0739	0.6647	1.22 (0.49-3.05)	0.4755	1.67 (0.41-6.86)		1.01E-05	0.1076
	rfs	HSP90AA1	0.0228	0.6978	0.0034	1.89 (1.23-2.88)	0.0525	1.86 (0.99-3.48)	rfs	0.1901	0.3523
	(n=204)	HSP90AB1	0.3514	-0.1869	0.4572	0.85 (0.56-1.30)	0.0556	0.56 (0.31-1.01)	(n=72)	0.5250	0.5411
		HSP90B1	0.0005	0.5361	0.0004	2.20 (1.42-3.42)	0.1722	1.65 (0.81-3.36)		0.8785	0.7860
		TRAP1	0.8973	-0.0322	0.2828	0.75 (0.45-1.27)	0.3785	1.74 (0.51-6.01)		NA	NA
		HSF1	0.6658	0.0981	0.1852	1.49 (0.83-2.69)	0.0093	2.87 (1.30-6.35)		0.4260	0.9968
	dmfs	HSP90AA1	0.4948	-0.2118	0.6939	0.92 (0.61-1.39)	0.4697	0.80 (0.44-1.45)	dmfs	0.7322	0.9935
	(n=347)	HSP90AB1	0.0488	-0.4080	0.886	1.04 (0.63-1.70)	0.9503	0.97 (0.40-2.36)	(n=90)	0.2891	0.6220
		HSP90B1	0.5186	0.1075	0.9586	0.99 (0.63-1.54)	0.9862	1.01 (0.49-2.08)		0.1640	0.2461
		TRAP1	0.0903	-0.4207	0.1023	0.63 (0.37-1.10)	0.0366	0.41 (0.18-0.95)		0.5307	0.9977
		HSF1	0.8684	-0.0328	0.4123	0.77 (0.40-1.45)	0.0969	0.53 (0.25-1.12)		0.9446	0.5903
HER2-ER+	os	HSP90AA1	0.1057	0.4143	0.0706	1.52 (0.97-2.39)	0.0563	1.92 (0.98-3.75)	os	0.1593	0.5829
	(n=506)	HSP90AB1	0.0015	0.5751	0.0918	1.44 (0.94-2.20)	0.0005	3.04 (1.63-5.68)	(n=228)	1.53E-05	0.0004
		HSP90B1	0.6534	-0.0745	0.5028	1.18 (0.73-1.89)	0.9867	0.99 (0.50-1.97)		0.4439	0.9294
		TRAP1	0.4514	-0.1998	0.818	1.05 (0.70-1.57)	0.311	0.75 (0.43-1.31)		0.2038	0.1686

Additional file 7. Prognosis of HSP90 and HSF1 in different breast cancer subtypes (continued)

subtype	phenotype	gene	Cox-regression analysis		Kaplan-Meier survival analysis				COXPH survival analysis		
					Hing 25% vs. others		Hing 10% vs. others		phenotype	p-value	p_adjusted
			p-value	Coef	p-value	HR (95%CI)	p-value	HR (95%CI)			
HER2-ER+	os	HSF1	0.7365	0.0720	0.8236	1.08 (0.57-2.05)	0.6418	1.24 (0.50-3.05)		0.0011	0.0107
	rfs	HSP90AA1	0.0012	0.4703	0.0186	1.35 (1.05-1.73)	0.2164	1.25 (0.88-1.79)	rfs	0.7668	0.7119
	(n=832)	HSP90AB1	0.2024	0.1294	0.6729	0.95 (0.76-1.19)	0.9413	0.99 (0.73-1.34)	(n=361)	0.2505	0.6151
		HSP90B1	0.0116	0.2065	0.0052	1.44 (1.11-1.85)	0.0708	1.39 (0.97-1.98)		0.8112	0.4392
		TRAP1	0.2899	-0.1492	0.7255	0.96 (0.76-1.21)	0.7909	0.96 (0.70-1.31)		0.0885	0.0840
		HSF1	0.0015	0.4019	0.111	1.30 (0.94-1.78)	0.0004	2.05 (1.38-3.05)		0.1101	0.4995
	dmfs	HSP90AA1	0.0217	0.3615	0.0064	1.46 (1.11-1.92)	0.0004	2.00 (1.36-2.94)	dmfs	0.2070	0.2112
	(n=1223)	HSP90AB1	0.0014	0.3730	0.0215	1.38 (1.05-1.80)	0.0062	1.71 (1.17-2.52)	(n=415)	0.0052	0.2059
		HSP90B1	0.9847	-0.0020	0.5807	0.92 (0.69-1.23)	0.4976	0.86 (0.56-1.32)		0.4544	0.1169
		TRAP1	0.8107	-0.0383	0.5753	1.08 (0.83-1.41)	0.715	0.93 (0.64-1.35)		0.5220	0.4827
		HSF1	0.0526	0.2427	0.4406	1.16 (0.80-1.68)	0.8467	1.05 (0.66-1.67)		0.0561	0.6533
TNBC	os	HSP90AA1	0.0049	1.0961	0.0302	2.07 (1.07-3.98)	< 0.0001	16.86 (4.66-60.91)	os	0.0079	0.0394
	(n=282)	HSP90AB1	0.1328	0.3875	0.0483	1.82 (1.00-3.30)	0.2936	1.83 (0.59-5.66)	(n=105)	0.4344	0.9968
		HSP90B1	0.5032	-0.1351	0.7241	0.90 (0.51-1.59)	0.5289	0.77 (0.34-1.73)		0.7345	0.9549
		TRAP1	0.1082	0.4093	0.0391	1.79 (1.03-3.11)	0.3111	1.48 (0.69-3.19)		0.2130	0.9967
		HSF1	0.9479	0.0139	0.8351	0.93 (0.45-1.90)	0.8427	1.09 (0.48-2.44)		0.7478	0.6372
	rfs	HSP90AA1	0.0003	0.8877	0.0001	2.58 (1.58-4.20)	0.0513	2.14 (1.00-4.59)	rfs	0.3702	0.7225
	(n=285)	HSP90AB1	0.0066	0.4432	0.0686	1.52 (0.97-2.38)	0.5822	1.25 (0.57-2.73)	(n=122)	0.0492	0.0930
		HSP90B1	0.0021	0.3790	0.0939	1.40 (0.94-2.08)	0.6856	1.11 (0.66-1.87)		0.4342	0.4292
		TRAP1	1.34E-05	0.8023	< 0.0001	2.28 (1.51-3.44)	< 0.0001	4.44 (2.20-8.97)		0.7630	0.5131
		HSF1	0.0076	0.5436	< 0.0001	3.90 (2.13-7.14)	< 0.0001	7.19 (3.14-16.47)		0.0008	0.0267
	dmfs	HSP90AA1	0.2762	0.2343	0.097	1.48 (0.93-2.33)	0.097	1.48 (0.93-2.33)	dmfs	0.0268	0.0526
	(n=516)	HSP90AB1	0.2787	0.1784	0.1118	1.44 (0.92-2.26)	0.9931	1.00 (0.47-2.15)	(n=158)	0.8232	0.8114
		HSP90B1	0.5273	-0.0926	0.215	0.78 (0.52-1.16)	0.8896	0.96 (0.55-1.69)		0.7293	0.7273
		TRAP1	0.4780	0.1342	0.2689	1.28 (0.83-1.96)	0.7443	1.10 (0.61-2.00)		0.3978	0.5291
		HSF1	0.0923	0.2573	0.045	1.65 (1.01-2.68)	0.0132	2.05 (1.16-3.61)		0.8116	0.8797

*dss: disease specific survival, using death from breast cancer as event; *os: over-all survival;

*rfs: recurrence-free survival; *dmfs: distant metastasis-free survival

*Cox-regression analysis was performed using expression as continuous variable

*COXPH: Cox multivariate analyses using size, grade, nodal status, age, ER, HER2 and PR as co-variants
