# Supplementary material for: Amplification and high-level expression of heat shock protein 90 marks aggressive phenotypes of human epidermal growth factor receptor 2 negative breast cancer
Source: Breast Cancer Res. 2012 Apr 17;14(2):R62. doi: 10.1186/bcr3168 (PMC3446397; doi:10.1186/bcr3168)
Supplement: Additional file 9 — Cox univariate and multivariate analyses of up-regulated HSP90. This table lists the results of Cox Proportional-Hazards (COXPH) Regression survival analyses of up-regulated HSP90 using samples where the entire set of clinical data was available. [file bcr3168-S9.PDF]

Additiona file 9. Cox univariate and multivariate analyses of up-regulated HSP90

Subtype	Event phenotype	n	*P* -value	co-variants							*P* -adjusted
				HER2	ER	PR	nodal	grade	size	age	
All samples	Death	421	0.0007	0.0219	0.5080	0.2856	0.0020	0.0522	0.2120	0.8804	0.0062
	Recurrence	581	0.1063	0.1301	0.9762	0.0043	0.9176	0.9671	2.35E-05	0.2520	0.1564
	Distant metastasis	689	0.1001	0.1651	0.6205	0.0002	0.3387	0.3172	1.33E-05	0.2618	0.4964
HER2+	Death	63	0.2564	NA	0.5271	0.2271	0.0008	0.3110	0.3628	0.2994	0.1405
	Recurrence	72	0.9528	NA	0.5585	0.2029	0.3216	0.5601	0.4956	0.6317	0.6705
	Distant metastasis	90	0.5383	NA	0.7348	0.3466	0.0313	0.8292	0.4205	0.2162	0.2330
HER2-/ER+	Death	228	0.0003	NA	NA	0.0659	0.2514	0.5738	0.0009	0.6943	0.0042
	Recurrence	361	0.1790	NA	NA	7.16E-05	0.7106	0.4540	2.61E-05	0.4344	0.3054
	Distant metastasis	415	0.0098	NA	NA	8.18E-06	0.7223	0.2024	7.45E-06	0.3987	0.0705
TNBC	Death	105	0.5693	NA	NA	NA	0.8438	0.0857	0.8803	0.7979	0.5869
	Recurrence	122	0.0008	NA	NA	NA	0.5923	0.2635	0.0154	0.8120	0.0101
	Distant metastasis	158	0.6722	NA	NA	NA	0.3205	0.8909	0.0083	0.9014	0.9390
